# Spatial distribution patterns and formation of global spermatophytes

**DOI:** 10.1111/jipb.13923

**Published:** 2025-05-23

**Authors:** Xian‐Han Huang, Tao Deng, Jun‐Tong Chen, Quan‐Sheng Fu, Xin‐Jian Zhang, Nan Lin, Peng‐Rui Luo, Qun Liu, Xin‐Yuan Kuai, Jing‐Yi Peng, Jacob‐B. Landis, Yan‐Tao Wei, Heng‐Chang Wang, Hang Sun

**Affiliations:** ^1^ State Key Laboratory of Plant Diversity and Specialty Crops Kunming Institute of Botany, Chinese Academy of Sciences Kunming 650201 China; ^2^ Yunnan International Joint Laboratory for Biodiversity of Central Asia Kunming Institute of Botany, Chinese Academy of Sciences Kunming 650201 China; ^3^ College of Life Science Henan Agricultural University Zhengzhou 450002 China; ^4^ School of Integrative Plant Science Section of Plant Biology and the L.H. Bailey Hortorium, Cornell University Ithaca 14853 New York USA; ^5^ Faculty of Artificial Intelligence in Education, Central China Normal University Wuhan 430079 China; ^6^ CAS Key Laboratory of Plant Germplasm Enhancement and Specialty Agriculture Wuhan Botanical Garden, Chinese Academy of Sciences Wuhan 430074 China

**Keywords:** distribution center, formation mechanism, formation time, K‐means clustering, origin location, Spermatophyte Spatial Evolutionary System

## Abstract

The evolution of spermatophytes (seed plants) is relatively well known in their evolutionary relationships over temporal changes, but their spatial evolution is another critical yet often neglected lens, especially using a taxon‐based approach. Here, by integrating geographic distributions and origin locations across 429 spermatophyte families worldwide with unsupervised machine learning approaches, we constructed a Spermatophyte Spatial Evolutionary System (SSES) that classifies global spermatophytes into 18 distribution types and six distribution supertypes within three primary floristic elements: cosmopolitan, tropical, and temperate. We found that the three elements all primarily originated from Gondwana, with the cosmopolitan element being the youngest and the temperate element being the oldest in terms of origin. They primarily formed during the Tertiary, particularly between the Eocene and Miocene, driven mainly by climate, long‐distance dispersal, and tectonic movement, while each exhibited distinct migration routes and formation models. Our results provide novel insights into the spatial evolution of global spermatophytes and highlight that similar distribution patterns of spermatophytes were driven by their comparable formation processes and mechanisms at the levels of floristic element, distribution supertype, and type.

## Graphical abstract

The global Spermatophyte Spatial Evolutionary System defines 18 distribution types and six supertypes across three floristic elements, primarily formed between the Eocene and Miocene and shaped by climate, long‐distance dispersal, and tectonic movement, revealing that similar distribution patterns of spermatophytes were driven by comparable formation processes and mechanisms.

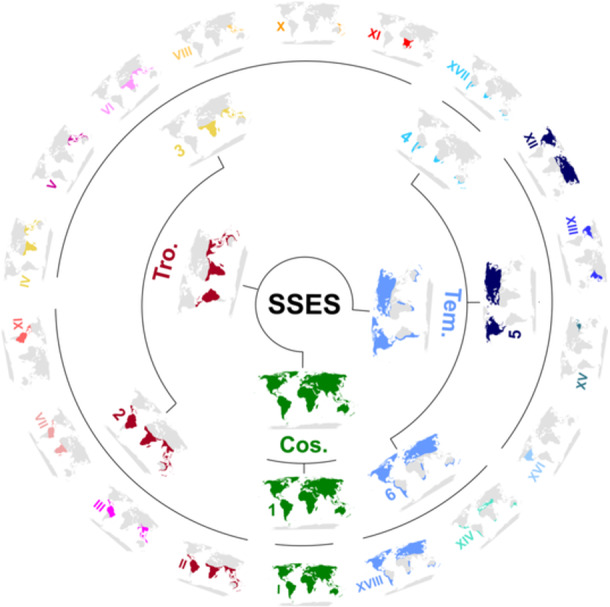

## INTRODUCTION

The evolution of life on Earth is not only reflected through a temporal lens, answering such questions as when an organism originated, who its ancestors were, and how many descendants it produced, but also through a spatial lens including questions of where it originated, how it spread, and how the current distribution pattern was formed. Multiple disciplines such as molecular phylogenetics, genomics, and paleobotany, are advancing rapidly, facilitating interdisciplinary projects such as the completion of the tree of life for spermatophytes (Smith and Brown, 2018). These advancements also include the continuous improvement and deepening of the Angiosperm Phylogeny Group (APG) classification system (APG, 1998; APG II, 2003; APG III, 2009; APG IV, 2016), as well as the recent gymnosperm classification (Christenhusz et al., 2017, 2011; Yang et al., 2022b), which have provided the study of plant evolution significant progress toward the classification and refinement of lower taxonomic levels. However, the study of plant evolution in terms of geographic scale is still in its infancy. Such study requires a complete phylogeny based on the temporal data of species, as well as relevant studies involving geological history and modern environment for support (Wu et al., 2003b, 2006). Therefore, there have been relatively few systematic studies of the spatial evolution of global spermatophytes corresponding to molecular phylogenetics to date (Carta et al., 2022; Liu et al., 2023). Exploring the spatial evolution and constructing a mostly complete Spermatophyte Spatial Evolutionary System (SSES) are key to understanding the formation of plants, along with constructing the tree of life, which is a significant endeavor for botanists.

The spatial evolution of plants is primarily a comprehensive reflection of the formation of distribution patterns, and the processes of dispersal and changes in those distribution patterns over time. This can be categorized into two key aspects using taxon‐based and flora‐based approaches. The former emphasizes the locations and time of origin, the dispersal pathway and modern distribution patterns of each taxon on Earth, namely, the plant distribution types (Wulff, 1943; Wu, 1991; Wu et al., 2003b), while the latter focuses on the composition, origin and spatial patterns of each flora on Earth, namely the floristic regionalization, and has attracted tremendous attention (Takhtajan, 1986; Carta et al., 2022; Liu et al., 2023). As for the plant distribution type, it classifies the approximate geographic distribution pattern, indicating that similar distribution patterns always have similar formation histories (Wu, 1991; Wu et al., 2003b). For example, the disjunct distribution pattern between East Asia and North America was associated with warm‐thriving plant taxa during the Tertiary, which used the Bering Land Bridge (BLB) as a crucial migration route, while taxa with the disjunct distribution pattern between tropical Asia and tropical America were always linked to the North Atlantic Land Bridges (NALBs) that existed in the early Tertiary (Donoghue, 2008; Wen et al., 2010; Huang et al., 2021). Undoubtedly, analyzing the origin and formation of different distribution types is essential for revealing the spatial evolution of plants. Nevertheless, these kinds of studies are constrained by recent progress in fields such as plant taxonomy, phylogenetics, and biogeography and are thus relatively limited to date.

Vester (1940) classified the distribution of global plant families into two distribution types and provided the distribution maps for these families. Good (1974) divided the global distribution of plants, again at the family level, into six distribution types: world and subworld, tropical, temperate, disjunct, endemic, and irregular. Wu (2003) and Wu et al. (2003b) conducted a comprehensive classification of global seed plant families, consisting of 18 distribution types and 63 subtypes based on available spatial distribution data of these families. At the genus level, van Balgooy (1969) and Hikcey (1977) classified noncultivated angiosperm genera of the Pacific Islands and plants of the Golden Valley Formation into nine distribution types, respectively. Wu, (1965, 1991) and Wu et al. (2010) classified 3,156 Chinese seed plant genera into 15 types and 35 subtypes and then analyzed their origins and relationships in depth. Additionally, Raven (1972) and Thorne (1972) preliminarily analyzed the major disjunct distribution patterns and possible formation mechanisms of global plants at different levels spanning families, genera, and even species. Nonetheless, the above‐mentioned studies of plant distribution types remain preliminary, mainly confined to a morphological‐geographic scale and lacking the integration of multiple disciplines. Accordingly, little progress has been made in the comprehensive study of global spermatophyte distributions over the past 20 years.

With the advent of newer applications of molecular biology techniques for constructing large molecular phylogenies, such as the APG system (APG, 1998; APG II, 2003; APG III, 2009; APG IV, 2016), monophyletic categories at various taxonomic levels, especially at the family level, have been clearly defined, enabling large‐scale studies of plant distribution types to be feasible. Combined with the increasing availability of highly accurate data on plant geographic distributions, the rapid development of molecular biogeography has also been greatly promoted, which reveals the origin, dispersal mechanisms, and modern distribution pattern formation of high‐level taxonomic groups. Recently, Pillon et al. (2021) constructed a high‐resolution genus‐level phylogeny of the Cunoniaceae using Angiosperms353 and inferred an Australasia origination during the Cretaceous, followed by long‐distance dispersal (LDD) and related Australasia‐America vicariance events leading to the currently observed tropical distribution. Currently, with the continuous accumulation of numerous types of data and the application of new analytical approaches arising from machine learning (Bergen et al., 2019; Hueffel et al., 2021; Capo et al., 2022), especially the widely used K‐means clustering (Macqueen, 1967) based on unsupervised machine learning, classifying more accurate distribution types across broad taxa is becoming more reliable, facilitating the study of global spermatophyte distributions at the family level.

**Figure 1:**
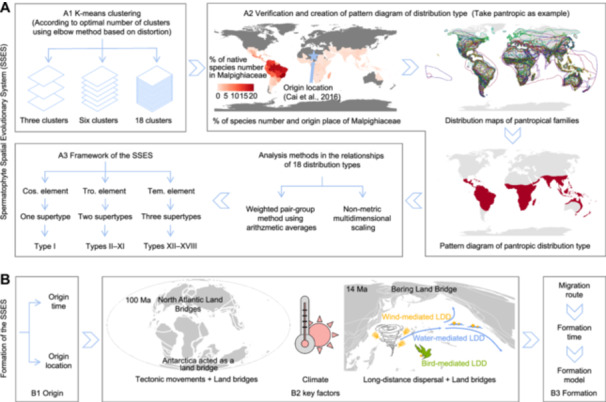
Analytical roadmap for the construction and formation of the Spermatophyte Spatial Evolutionary System (SSES) **(A)** The SSES was reconstructed by three steps. (A1) Preliminary classification of distribution type of 429 spermatophyte families through unsupervised machine learning approaches. (A2) Verification of corresponding distribution types of each family and creation of pattern diagram of each distribution type. The origin location of Malpighiaceae (Cai et al., 2016) is marked by a pentagram. In the distribution maps of pantropical families, the closed lines of each color represent the distribution range of one family. Pattern diagram of pantropic distribution type is drawn based on the main distribution range of its families. (A3) Analysis of the relationships of 18 distribution types. The Cos., Tro., and Tem. represent the cosmopolitan, tropical and temperate, respectively. **(B)** Formation of this SSES involves its origin (B1), key factors (B2), and formation (B3). In B2, base maps for the Cretaceous (100 Ma) and Neogene (14 Ma) were obtained from Ocean Drilling Stratigraphic Network plate tectonic reconstruction service (http://www.odsn.de/odsn/services/paleomap/paleomap.html).

Attempting to understand the SSES using the taxon‐based approach, this study integrates the latest family‐level classification system, geographic distributions, origin information, relevant geological history, and ecological environment to reveal the spatial distribution patterns and formation of global spermatophytes (Figure 1). Specifically, we address the following issues: (i) classifying the distribution types of the 429 global spermatophyte families and reconstructing their spatial evolutionary framework; (ii) determining the origin of the distribution types, distribution supertypes, and floristic elements and then clarifying their migration routes, formation time, and models through formation factors.

## RESULTS AND DISCUSSION

### Comprehensive framework of global spermatophyte spatial evolution

**Figure 2:**
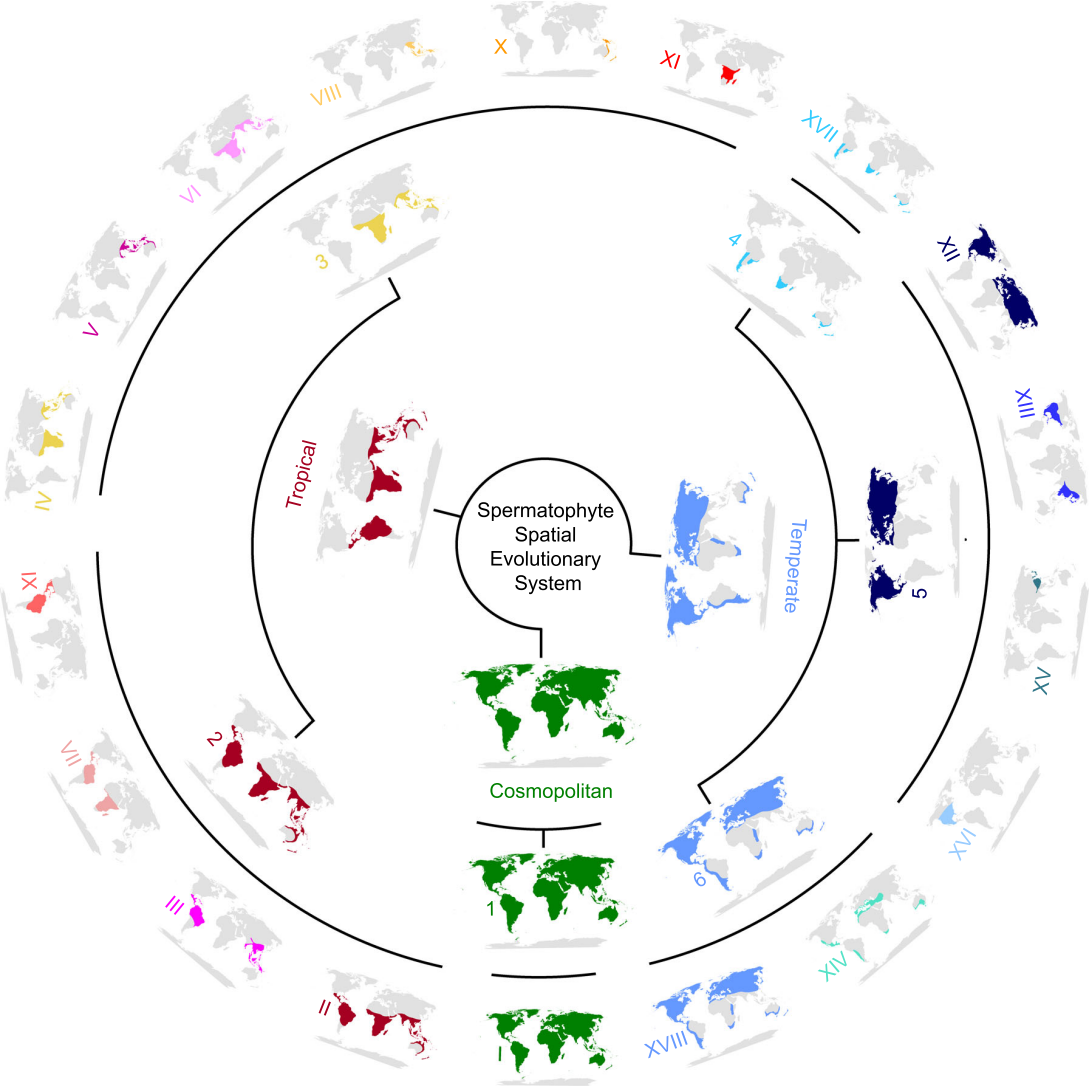
The Spermatophyte Spatial Evolutionary System (SSES) frame and its pattern diagrams The black line framework represents the SSES frame, which consists of three floristic elements, six distribution supertypes, and 18 distribution types (Table 1). A pattern diagram is provided for each type at the three hierarchical levels. Among them, “Cosmopolitan,” “Tropical,” and “Temperate” represent the cosmopolitan, tropical, and temperate elements, respectively; numbers 1–6 denote the six distribution supertypes, respectively; and I–XVIII represent the 18 distribution types, respectively.

Combined with the distribution characteristics of the family and the previous divisions of distribution types (Wu, 2003; Wu et al., 2003b), the optimized numbers of clusters K were set as three, six, and 18 for the K‐means clustering of the distribution of global spermatophyte families based on the optimal number of clusters determined using the elbow method considering distortion (Figure S1). *K*‐means clustering showed when *K* = 3, 77.65% of families of Cluster 1 were widespread, 97.93% of families of Cluster 2 were with a predominant distribution in tropical zones, and 100% of families of Cluster 3 were with a main distribution in temperate zones; these three clusters were defined as cosmopolitan, tropical, and temperate elements, respectively (Figures 2, S2A; Table S1). With *K* = 6, more than or approximately 75% of families in each of the six clusters had similar distribution patterns, and these six clusters were defined as six distribution supertypes (Figures 2, S2B; Table S1). With *K* = 18, more than or approximately half of the families in each of the 13 clusters had similar distributions, excluding the third, seventh to eighth, 12th, and 14th clusters (Figures 2, S2C; Table S1). Focusing on the distribution center of each cluster, the first to second, fourth to sixth, ninth to 11th, 13th, and 15th to 18th clusters were defined as 13 distribution types (Figures 2, S2C, S3; Table S1). Subsequently, the K‐means clustering results were verified and/or adjusted one by one on the basis of the classification principles of distribution type. For example, Africa was inferred as the origin for Malpighiaceae (Cai et al., 2016), which supported the family belonging to Type II (pantropic). The third, seventh to eighth, 12th, and 14th clusters were defined as tropical Asia and tropical America disjunct distribution type (i.e., Type III), South Atlantic disjunct distribution type (i.e., Type VII), tropical Asia distribution type (i.e., Type VIII), north temperate distribution type (i.e., Type XII) and pan‐Mediterranean diffused distribution type (i.e., Type XIV; Figures 2, S2C, S3; Table S1), respectively, on the basis of previous studies (Wu et al., 2003b, 2006). With the addition of 10 subtypes among the six distribution types based on characteristics of endemic and disjunct distributions of families, the distribution of 429 global spermatophyte families can be classified into three floristic elements, six distribution supertypes, 18 distribution types, and 10 distribution subtypes (Table 1), which partly supported the results of previous studies (Wu, 2003; Wu et al., 2003b) and was involved with less human interference, further supporting its reliability and scientific basis.

**Table 1:** The Spermatophyte Spatial Evolutionary System (SSES) and its relevant information

Number	Distribution type	Floristic element	Distribution supertype	Distribution subtype	Distribution	Number of family	Typical families
1	Ⅰ. Cosmopolitan	Cosmopolitan	1. Cosmopolitan	‐	Almost on all continents of the world, but without special distribution center, or although some families have one or several distribution centers, there are global distribution taxa in these families	66	Ceratophyllaceae, Orchidaceae, etc.
2	Ⅱ. Pantropic	Tropical	2. Tropics involving New World	Ⅱ.Ⅰ Tropical Asia‐Australasia and tropical America; Ⅱ.Ⅱ Tropical Asia‐tropical Africa‐tropical America	Widely in the tropics of the world	99	Myristicaceae, Escalloniaceae, Achariaceae, etc.
3	Ⅲ. Tropical Asia and tropical America disjuncted	Tropical	2. Tropics involving New World	‐	Mainly and disjunctly between tropical and subtropical Asia and tropical and subtropical America (Central and South America)	13	Bonnetiaceae, Sabiaceae, etc.
4	Ⅳ. Old World tropics	Tropical	3. Old World tropics	‐	Widely in tropical Asia, tropical Australasia and tropical Africa	7	Musaceae, Pandanaceae, etc.
5	Ⅴ. Tropical Asia to tropical Australasia	Tropical	3. Old World tropics	‐	Continuously or disjunctly between tropical Asia to tropical Australasia	14	Hanguanaceae, Philydraceae, etc.
6	Ⅵ. Tropical Asia to tropical Africa	Tropical	3. Old World tropics	‐	Continuously or disjunctly between tropical Asia to tropical Africa	16	Ancistrocladaceae, Pandaceae, etc.
7	Ⅶ. South Atlantic disjuncted	Tropical	2. Tropics involving New World	‐	Mainly and disjunctly between tropical Africa and tropical America	11	Canellaceae, Strelitziaceae, etc.
8	Ⅷ. Tropical Asia	Tropical	3. Old World tropics	‐	Widely in tropical Southeast Asia, Indo‐Malaya and tropical southern and southwestern Pacific Islands	8	Lowiaceae, Rafflesiaceae, etc.
9	Ⅸ. New World tropics	Tropical	2. Tropics involving New World	‐	Widely in tropical America	35	Cannaceae, Goupiaceae, etc.
10	Ⅹ. Tropical Australasia	Tropical	3. Old World tropics	Ⅹ.Ⅰ Endemic to New Caledonia; Ⅹ.Ⅱ Endemic to Fiji	Widely in tropical Australasia	17	Byblidaceae, Oncothecaceae, Degeneriaceae, etc.
11	Ⅺ. Tropical Africa	Tropical	3. Old World tropics	Ⅺ.Ⅰ Endemic to Madagascar	Widely in tropical Africa	18	Hydrostachyaceae, Barbeuiaceae, etc.
12	Ⅻ. North temperate	Temperate	5. North temperate	‐	Widely in temperate regions of Asia, Europe and North America	20	Liliaceae, Pinaceae, etc.
13	XIII. East Asia and North America disjuncted	Temperate	5. North temperate	‐	Mainly and disjunctly between temperate and subtropical regions of East Asia and North America	12	Penthoraceae, Saururaceae, etc.
14	XIV. Pan‐Mediterranean diffused	Temperate	6. North and south temperate	XIV.Ⅰ Mediterranea, West Asia to Central Asia	Widely in Mediterranea to North Africa, Central Asia, South Africa, southern Australia, southwestern South and North America	14	Frankeniaceae, Drosophyllaceae, etc.
15	XV. East Asia	Temperate	5. North temperate	XV.Ⅰ Endemic to Japan	Mainly from the Himalayas to Japan	11	Eupteleaceae, Sciadopityaceae, etc.
16	XVI. North America	Temperate	5. North temperate	‐	Widely in temperate North America	9	Crossosomataceae, Limnanthaceae, etc.
17	XVII. South temperate	Temperate	4. South temperate	XVII.Ⅰ Temperate South America; XVII.Ⅱ temperate Australasia; XVII.Ⅲ temperate Africa	Widely in temperate regions of South America, Africa and Australasia	43	Griseliniaceae, Misodendraceae, Dasypogonaceae, Grubbiaceae, etc.
18	XVIII. North and south temperate disjuncted	Temperate	6. North and south temperate	‐	Disjunctly between south temperate and north temperate	16	Ephedraceae, Zosteraceae, etc.

The three floristic elements were supported by the three categories (*r* ^2^ = 0.99, stress = 0.09; Figure S5) clustered by 18 distribution types based on non‐metric multidimensional scaling (NMDS). Furthermore, the clustering results also revealed three superclades based on the weighted pair‐group method using arithmetic averages (WPGMA; Figure S5; Table S2). However, the first superclade included Type I (Clade 1, namely supertype 1 with cosmopolitan distribution; Figures 2, S5; Table 1) and Clade 6 (namely the distribution supertype 6 of north and south temperate regions) including Types XIV and XVIII (north and south temperate disjuncted), which was likely associated with the fact that these three distribution types all cover several of the same parts of the north and south temperate region but the latter two distribution types are not distributed in the tropics and were still included in the temperate element. For the remaining 15 distribution types, Types II, Ⅲ (tropical Asia and tropical America disjuncted), and Ⅸ (New World tropics) clustered into Clade 2, corresponding to the distribution supertype 2 associated with the New World tropics, and Types IV (Old World tropics) to Ⅺ (tropical Africa) except for Type Ⅸ, clustered into Clade 3 corresponding to the distribution supertype 3 associated with the Old World tropics. These 10 distribution types formed a second superclade, representing the tropical floristic element, which then formed a sister clade with the remaining five distribution types (Clade 5, namely the distribution supertype 5 associated with north temperate distribution, and Clade 4, namely the distribution supertype 4 associated with south temperate distribution) of the temperate floristic element. Specifically, Type VII with the South Atlantic disjunct distribution formed a sister clade with Type Ⅺ in Clade 3 with the primary Old World tropics distribution (Figure S5), which appears to be related to the fact that these two distribution types both involve an African distribution, and Type VII likely acts as a link between the distribution types of the Old and New World tropics. However, to ensure that Clade 3 is exclusively an Old World tropical clade, our subsequent analyses assigned Type VII to Clade 2 involving the New World tropics. Combined with the perspective of Wu et al. (2010), the SSES was constructed, with the cosmopolitan element including supertype 1 and Type I, the tropical element containing supertypes 2–3 and Types II–XI, and the temperate element comprising supertypes 4–6 and Types XII–XVIII (Figure 2; Tables 1, S1). Excluding Cephalotaxaceae and Tiganophytaceae, the three floristic elements (Blomberg's *K* = 0.37, Pagel's *λ* = 0.34, *P* < 0.05 for both), six distribution supertypes (Blomberg's *K* = 0.40, Pagel's *λ* = 0.42, *P* < 0.05 for both), and 18 distribution types (Blomberg's *K* = 0.36, *P* < 0.07; Pagel's *λ* = 0.38, *P* < 0.05) showed significant phylogenetic signals, indicating that families with closer phylogenetic relationships have more similar floristic elements or distribution supertypes or distribution types.

### Young cosmopolitan element predominantly driven by LDD and tectonic movement

**Figure 3:**
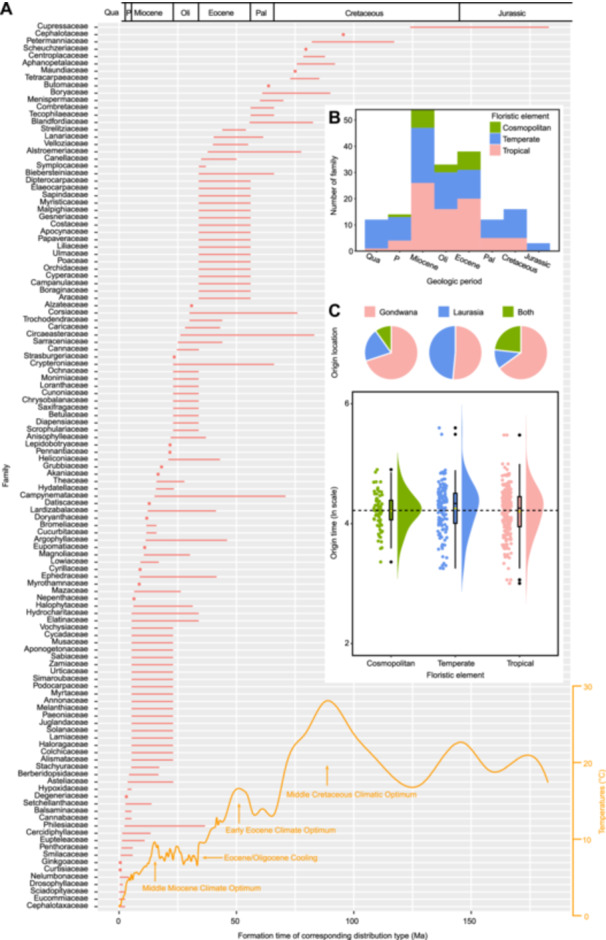
Formation of the corresponding distribution types and origin of the three floristic elements of spermatophyte families **(A)** Formation time of corresponding distribution types of 121 spermatophyte families is shown by points or intervals. A trend line of paleotemperatures (Zachos et al., 2008) was drawn using the smooth.spline function in R and significant climate events were labeled during the corresponding formation time. **(B)** Distribution of the formation time for corresponding distribution types of 121 spermatophyte families. Origin time for three floristic elements based on ln origin time for 429 families of spermatophytes, except for Cephalotaxaceae and Tiganophytaceae (the lower portion of **(C)**), and percentage of origin location for three floristic elements based on origin locations of 96 spermatophyte families (the upper portion of **(C)**).

**Figure 4:**
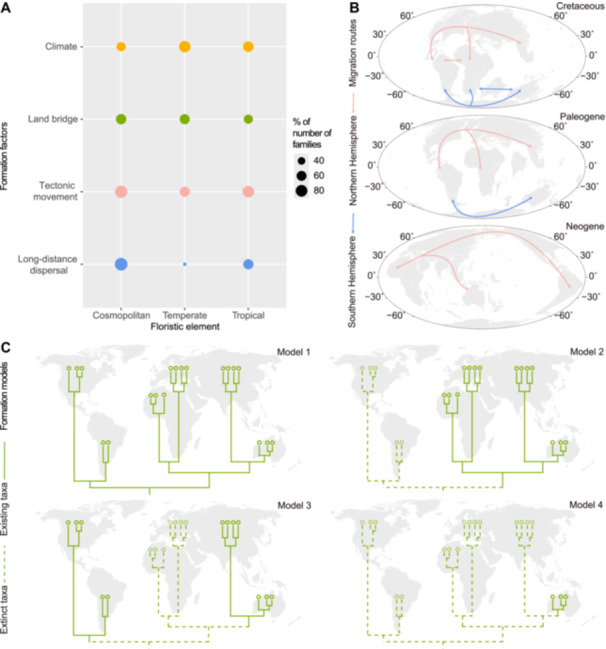
Formation factors, migration routes and formation models of the Spermatophyte Spatial Evolutionary System (SSES) **(A)** Dot‐plot of formation factor statistics for the three floristic elements based on 89 spermatophyte families. The size of the circle represents the percentage of the number of families involving the mentioned factor within each formation factor of a floristic element. **(B)** Major migration routes of spermatophyte families during the Cretaceous, Paleogene and Neogene. Base maps for the Cretaceous (100 Ma, Upper), Paleogene (50 Ma, Middle) and Neogene (14 Ma, Lower) were obtained from Ocean Drilling Stratigraphic Network plate tectonic reconstruction service. **(C)** Formation models of distribution types. Assuming the Americas as the origin place, Model 1 represents a family originating and then dispersing to other regions or globally without reducing its distribution area; Model 2 represents a family originating and dispersing, but then becoming extinct in its origin place and retaining a wide distribution area; Model 3 represents a family originating and dispersing, but then becoming extinct in regions other than its origin place, forming ultimately a modern disjunct distribution; Model 4 represents a family originating and dispersing, but then becoming extinct in its wide distribution area, ultimately surviving finally in a localized area.

The LDD pattern was initially proposed by Darwin and Wallace to explain the transoceanic distribution of organisms (Wallace, 1876; Darwin, 1991), mainly mediated by birds, ocean currents, and wind (Nathan, 2006; Gillespie et al., 2012). Regarding the cosmopolitan element (the youngest element among the three floristic elements in origin time; Figure 3C; Table S3), which is only Type I, widely distributed around the world, 94.12% of the investigated families were inferred to be associated with this pattern (Figure 4A; Table S4). The relatively consistent and stable aquatic environments appear to facilitate the dispersal/migration and evolution of some taxa in this element (Schenck, 1886), especially those in Alismataceae (Li et al., 2022), Araceae (Nauheimer et al., 2012), Elatinaceae (Cai et al., 2016), and Haloragaceae (Chen et al., 2014). For example, the biogeographical study of Araceae showed that DNA substitution rates are especially high in free‐floating Araceae (Nauheimer et al., 2012). Meanwhile, the dry to fleshy fruits of the Solanaceae were inferred to be adapted to a variety of LDD events by birds and ocean currents (Dupin et al., 2017), and *Empetrum* of Ericaceae was also associated with a single Mid‐Pleistocene bird‐mediated LDD (Popp et al., 2011). Additionally, the bristles of *Eriophorum* in Cyperaceae (Spalink et al., 2016) have adapted to wind‐mediated LDD to some extent. Statistically, the cosmopolitan element is also the floristic element with the largest percentage of families involved in the LDD mechanism among the three floristic elements (Figure 4A).

In terms of the formation of the cosmopolitan element, geological history is undoubtedly another significant factor, and 88.24% of the investigated families were inferred to be correlated with the tectonic movements (Figure 4A; Table S4), particularly during the final stage of the splitting of Pangaea, such as Alismataceae (Li et al., 2022), Boraginaceae (Luebert et al., 2017), Orchidaceae (Givnish et al., 2016a), and Scrophulariaceae (Villaverde et al., 2023). Land bridges appear to facilitate or enhance plant migration (Graham, 2018), which were also inferred to play a crucial role in the acquisition of a widespread distribution, such as in Cannabaceae (Jin et al., 2020) and Colchicaceae (Chacón and Renner, 2014). Additionally, global climate change (Zachos et al., 2008; Ruddiman, 2014) was considered as the other factor in the formation of this element, such as in Campanulaceae (Crowl et al., 2016), Scrophulariaceae (Villaverde et al., 2023), and Ulmaceae (Zhang et al., 2022a). Herbaceous plants, such as the Gnaphalieae tribe of Asteraceae (Nie et al., 2016) and the Rubieae tribe of Rubiaceae (Yang et al., 2022a), were both inferred to have rapidly spread worldwide following global cooling and drying, the gradual fragmentation of continental forests, and the expansion of open habitats, such as grasslands, since the late Miocene. However, the cosmopolitan element had the smallest percentage of families involving climate factors (climate‐mentioned: 52.94% of families; Table S4) among the three floristic elements.

Statistically, the cosmopolitan element comprising 66 families was the youngest among the three floristic elements in origin time (Figure 3C; Table S3). This element primarily originated from Gondwana (14 families, 70.00% of cosmopolitan families; Figures 3C, S6, S7; Table S5), with its formation occurring in the Eocene and Miocene (Figure 3A, B; Table S6). Combined with the LDD, this element also had the highest proportion of animal‐, water‐, and wind‐dispersed families (72.73%, 68.18%, and 59.09% of families, respectively; Table S3), indicating the strongest dispersal ability among the three floristic elements, even across the 18 distribution types. The cosmopolitan element was inferred to involve the migration routes of the Paleogene and Neogene (Figure 4B) and the first formation model (Figure 4C). Together with the cosmopolitan element occupying the largest geographic distribution area and more climate types, this element has the most families of mixed herbaceous and woody plants (Figure S8) and mixed annual and perennial herbaceous plants (Table S7) among the 18 distribution types, indicating that it also seems to occupy the most ecological niches.

### Ancient temperate element largely influenced by climate, land bridge, and tectonic movement

#### Distribution supertype 6 of north and south temperate regions

Climate change can alter the distribution of plants (Parmesan and Yohe, 2003; Kelly and Goulden, 2008), promoting expansion in favorable regions and contraction or even extinction in unfavorable regions, especially with the dramatic climate events over 100 million years ago (Ma) (Zachos et al., 2008; Ruddiman, 2014) and regional climate changes. Regarding the formation of distribution supertype 6 of north and south temperate regions and Types XIV and XVIII (see “Results and Discussion” for a more detailed description in Supporting Information), climate was a significant factor (climate‐mentioned: 70.00% of families; Table S4). The formation of Type XIV was inferred to be influenced by Mediterranean climate, which is characterized by less rainfall at higher temperatures and more rainfall at lower temperatures, and partly related to tectonic movements, particularly the evolution of the Tethys Sea (Wu et al., 2006), as observed in Frankeniaceae and Tecophilaeaceae (Buerki et al., 2013). In Type XVIII, with a disjunct distribution between the north and south temperate zones, as observed in Betulaceae, the deterioration of the climate during the Oligocene and the Quaternary glaciation were likely significant factors promoting the southward migration of this family into the Southern Hemisphere (Chen et al., 1999).

Meanwhile, ancient families of the supertype 6 were always linked to tectonic movements (tectonic movement‐mentioned: 70.00% of families; Table S4). For example, Cupressaceae of Type XVIII was inferred to originate in the intact Pangea during the Triassic and then formed two subfamilies with the separation of Laurasia and Gondwana, and three further intercontinental disjunctions coincided with or immediately followed the breakup of Pangea (Mao et al., 2012) (indicating the second or third formation model; Figure 4B, C). Several land bridges also exerted substantial influence on the formation of this supertype, particularly for Type XVIII. Studies have suggested that the BLB, available to terrestrial biota during the early Paleocene to 7.4–4.8 Ma (Tiffney and Manchester, 2001), played a role in plant exchange such as in Melanthiaceae (Givnish et al., 2016b), Papaveraceae (Peng et al., 2023), and Saxifragaceae (Deng et al., 2015; Ebersbach et al., 2017) (Figure 4B). Similarly, the NALBs, which existed during ~154–151 and ~131–129 Ma in the Mesozoic (Brikiatis, 2016) and the Paleocene to Eocene (Tiffney and Manchester, 2001), were inferred to have similar impacts on the formation of Betulaceae (Chen et al., 1999) (Figure 4B). During the formation of Type XIV, the Southern Hemisphere clade of Coriariaceae reached the New World once through Antarctica acting as a land bridge, as supported by fossil pollen (Renner et al., 2020) (Figure 4B). Together, this distribution supertype comprising 30 families was relatively ancient among six distribution supertypes in origin time (Figure S6; Table S3), primarily originated from Laurasia (Figure S6; Table S5), formed during the Tertiary (Figure 3A; Table S6), which was largely influenced by climate and tectonic movement (Table S4), the Paleogene and Neogene migration routes (Figure 4B), and the first three formation models (Figure 4C).

#### Distribution supertype 4 of the south temperate region

Distribution supertype 4, including only Type XVII (south temperate) and 43 families, was estimated to be the youngest distribution supertype in origin time (Figure S6; Table S3). It originated from Gondwana (Figures S6, S7, S9; Table S5) and formed mainly during the Late Cretaceous to the Neogene (Figure 3A; Table S6). The formation of this distribution type was primarily affected by several factors, namely the LDD, climate, and tectonic movement (LDD‐mentioned, climate‐mentioned, and tectonic movement‐mentioned: 100%, 66.67%, 66.67% of families, respectively; Table S4). For example, Alstroemeriaceae (Chacón et al., 2012) was inferred to originate from the southern Andes and Australasia at 93.4 Ma (115.8–73.4 Ma), which is supported by the fact that Australia, South America, and Antarctica were connected or close to each other at that time (Reguero et al., 2002; Iglesias et al., 2011). Subsequently, a vicariance event between South America and Australasia and the LDD from South America to New Zealand were detected for this family, and its evolution was also likely associated with the uplift of the Patagonian Andes and arid conditions. The LDD was also emphasized as an important factor in the formation of the distribution of Asteliaceae (Birch and Keeley, 2013; Birch and Kocyan, 2021). The Magellan land bridge was also indicated to facilitate the plant exchange among southern temperate continents, such as in Nothofagaceae (Hill, 2001). Additionally, three subtypes were proposed in Type XVII based on the distribution centers of families, namely subtypes XVII.Ⅰ (temperate South America), XVII.ⅠⅠ (temperate Australasia), and XVII.ⅠⅠⅠ (temperate Africa). In the subtype XVII.Ⅱ, three families were inferred to originate from Australia, with the formation of Hydatellaceae (Iles et al., 2014) being related to the climate and the LDD. Overall, the formation of the distribution supertype 4 involved origination *in situ* (Figure 3C; Table S5), along with the LDD, climate, and tectonic movement (Table S4), and the first formation model (Figure 4C).

#### Distribution supertype 5 of the north temperate region

Regarding the formation of the distribution supertype 5 (including Types XII, XIII, XV, and XVI, and 52 families) in the north temperate region, previous studies indicated that it was associated with sizable obstacles, such as the Bering Strait, the Turgai Strait, the North Atlantic, the early Tertiary inland trench and the mid‐Tertiary arid zones of North America, the Chinese arid zones and the Central Asian arid zones (Tiffney and Manchester, 2001). Liliaceae of Type XII with a north temperate distribution was inferred to originate in North America and then migrated to Eurasia by the BLB, overcoming the obstacle of the Bering Strait (Vinnersten and Bremer, 2001; Givnish et al., 2016b) (Figure 4B). In contrast, the crown group Juglandaceae was estimated to be of boreotropical origin from the Middle to Late Cretaceous and then achieved its contemporary distribution via dispersal across the NALBs, facilitated by the retreat of the Turgai seaway, the closure of the Tethys Ocean within Eurasia, and climate change (Zhang et al., 2022b).

Climate exerted the greatest influence on the formation of distribution supertype 5 (climate‐mentioned: 84.62% of families; Table S4). With respect to the Nelumbonaceae of Type XIII (disjunct East Asia and North America), the origins were traced to the mid‐latitudes of Laurasia in the Early Cretaceous, then expanding their distribution range to the northernmost part of the Northern Hemisphere due to the Early Eocene Climate Optimum (EECO), while becoming extinct in Europe and Central Asia during Pleistocene glacial periods and finally forming the current disjunct distribution based on fossil evidence (Li et al., 2014). Similarly, Crossosomataceae of Type XVI (North America) was inferred to originate in the Northern Hemisphere prior to the Tertiary and then likely experienced drastic climatic changes, including glaciation and aridification, becoming adapted to the xeric environments in western North America (Zhu et al., 2006). Furthermore, the formation of Stachyuraceae (Zhu et al., 2006; Feng et al., 2020) of Type XV (East Asia) was inferred to be shaped jointly by mountain‐building movements and paleoclimates such as global cooling and the strengthening of the East Asian summer monsoons, which highlighted the importance of regional climate for the formation of this distribution type. In addition, combined with the fossil distribution of Eucommiaceae (Brown, 1962; Guo, 2000; Zhou and Momohara, 2005) and Cercidiphyllaceae (Qi et al., 2012; Herman, 2013) in Type XV, both Types XV and XVI involved the fourth formation model, and Type XIII involved primarily the second or third formation models (Figure 4C).

Collectively, the temperate element comprising 125 families was estimated to be the oldest element among three floristic elements in origin time (Figure 3C; Table S3). The number of families originating from Gondwana and Laurasia is relatively similar in this element (Figure 3C; Table S5), and it formed during the Tertiary (Figure 3A; Table S6), which was driven primarily by climate, land bridges, and tectonic movement (climate‐mentioned, land bridge‐mentioned, and tectonic movement‐mentioned: 76.92%, 61.54%, and 61.54% of families, respectively; Figure 4A; Table S4). Its formation was always involved in migration routes of the Paleogene and Neogene, and all four formation models (Figure 4B, C).

### Tropical element mainly shaped by climate and tectonic movement

#### Distribution supertype 3 of the Old World tropics

Overall, climate, the LDD, and tectonic movement were the major factors for the formation of distribution supertype 3 characterized by the Old World tropics (climate‐mentioned, LDD‐mentioned, and tectonic movement‐mentioned all: 54.55% of families; Table S4), including Types IV, V (tropical Asia to tropical Australasia), VI (tropical Asia to tropical Africa), VIII, X (tropical Australasia), and XI. Combined with the formation times of these types (mainly from the Late Cretaceous to Neogene, especially Miocene), their formation was associated with the collisions of Africa, the Indian subcontinent, and Australia with the Eurasian plate (Scotese, 2001; Smith et al., 2004). Crypteroniaceae of Type VIII was inferred to originate in Gondwana during the Early to Middle Cretaceous, then diversify on the Indian subcontinent, and subsequently migrated/dispersed to Southeast Asia (Rutschmann et al., 2004), highlighting the role of the Indian subcontinent in the evolution of this family. Similar formation mechanisms also appeared in Dipterocarpaceae, but this family also involved bird‐mediated LDD (Bansal et al., 2022). Furthermore, Musaceae (Kress and Specht, 2006; Janssens et al., 2016) of Type IV and Nepenthaceae (Biswal et al., 2018) of Type V were inferred to originate from the tropics and Africa, respectively, but their subsequent evolution was related to the drop in sea level and the emergence of land between Asia and Oceania after the collision of the Sunda and the Sahul Shelfs as well as climate cooling. The dispersal of Musaceae from northern Indo‐Burma to Africa during the Miocene could be the result of a gradual overland dispersal via an Arabian corridor or a LDD event (Janssens et al., 2016), which were also the significant factors for the formation of the palaeotropical intercontinental disjunctions in Annonaceae corresponding to a post‐boreotropical process (Thomas et al., 2015). Additionally, the rapid diversification of *Impatiens* (Balsaminaceae) was also indicated to be linked to the global cooling during the Pliocene and Pleistocene (Janssens et al., 2009). The formation of Type XI appears to be correlated with the formation and evolution of the Sahara Desert or the aridification of Africa (Wu et al., 2006; Zhang et al., 2014).

The earliest fossils of Pandanaceae (Snead, 1969) and Aponogetonaceae (Grimsson et al., 2014) of Type IV, and Zingiberaceae (Hickey and Peterson, 1978) of Type V were found in Canada during the Paleogene, the United States during the Late Cretaceous, and the United States during the Late Cretaceous, respectively, suggesting that these families were distributed in high‐latitude regions of the Northern Hemisphere in the early stages of their evolution and then migrated southward to form their current distribution due to factors such as climate cooling (highlighting the significance of the second or third formation model; Figure 4C). In addition, supertype 3 also involved the LDD, such as Aponogetonaceae of Type IV (Chen et al., 2015), Argophyllaceae (Maurin and Smissen, 2022), and Campynemataceae (Givnish et al., 2016b) of Type X, and Dipterocarpaceae of Type VI (Bansal et al., 2022). Three subtypes were proposed based on the endemic distribution of families, namely subtypes Ⅹ.Ⅰ (endemic to New Caledonia) and Ⅹ.Ⅱ (endemic to Fiji) in Type Ⅹ, and subtype Ⅺ.Ⅰ (endemic to Madagascar) in Type Ⅺ. Considering the formation history of the islands where these three subtypes are located (Scotese, 2001; Smith et al., 2004), if the origin time for families within these subtypes predated the emergence of the islands, it was highly likely that these families dispersed/migrated to these islands from their surrounding land areas via the LDD and went extinct in their origin place. In summary, the Old World tropics distribution supertype comprising 80 families is relatively young in the origin and formation time (Figure S6; Tables S3, S6), and its formation primarily involved the tectonic movement of the Indian Ocean and surrounding areas and related climates (Wu et al., 2006; Biswal et al., 2018), the migration routes of the Neogene (Figure 4B), and several formation models (Figure 4C).

#### Distribution supertype 2 of tropics involving New World

With respect to Type II characterized by pantropic distribution, two migration routes are often involved: one route is from the New World tropics into the Old World tropics via the NALBs; the other route is a route in the opposite direction (Donoghue, 2008) (Figure 4B). The second migration route can also be traced in the evolution of Urticeae (Huang et al., 2019), which also corresponds to the northern Paleogene migration route. Similarly, Sabiaceae in Type III characterized by the disjunct distribution between tropical Asia and tropical America was inferred to originate in Eurasia during the Early Cretaceous and then spread to the Northern Hemisphere with a boreotropical range expansion during the Paleogene but subsequently migrated southward as global temperature dropped in the Miocene to form the current disjunct distribution (Yang et al., 2018) (namely, the third formation model; Figure 4C). Furthermore, when Antarctica acted as a land bridge from the Cretaceous to the Paleogene, another major migration route was involved for supertype 2 associated with the New World tropics (including Types II, III, VII, and IX, and 158 families), namely the southern Paleogene migration route (Figure 4B), which was inferred to play a role in the evolution of Cunoniaceae or Elaeocarpaceae (Pillon et al., 2021). Additionally, the LDD was also suggested to occur in the formation of this supertype (LDD‐mentioned: 61.29% of families; Table S4), such as the berries of Loranthaceae (Liu et al., 2018) and Smilacaceae (Qi et al., 2023) in Type II, the fruits of Lardizabalaceae (Wang et al., 2020) and Sabiaceae (Yang et al., 2018) in Type III, and Canellaceae (Müller et al., 2015) in Type VII were all inferred to adapt largely to bird‐mediated LDD; Urticaceae (Wu et al., 2018) in Type II, Anisophylleaceae (Zhang et al., 2007) in subtype II.II with tropical Asia‐tropical Africa‐tropical America distribution, and Caricaceae (Carvalho and Renner, 2012) in Type VII mainly to water‐mediated LDD.

Tectonic movements (tectonic movement‐mentioned: 88.57% of families; Table S4) have had the greatest impact on the formation of the supertype 2. Beyond the tectonic movements of the Palaeotropics mentioned above, plate tectonics in other regions of the Gondwana and the associated uplift of mountain ranges played a crucial role in their formation. Specifically, combined with the relatively ancient time of origin and formation of some families associated with this supertype, such as Vochysiaceae (Goncalves et al., 2020) of Type IX, the formation of these families was hypothesized to be associated with the splitting of West Gondwana, which occurred primarily during the Early Cretaceous (135 Ma) to Middle and Late Cretaceous (110–95 Ma) (Sanmartin and Ronquist, 2004). Corsiaceae of subtype II.I with a tropical Asia‐Australasia and tropical America distribution was inferred to originate from Gondwana in the Late Cretaceous, with subsequent evolution potentially related to the fragmentation of the Gondwanan plates (Mennes et al., 2015), and the formation of Anisophylleaceae in subtype II.II is also suggested to be related to the disintegration of Gondwana (Zhang et al., 2007) (Figure 4B). The formation of Type IX was associated with the uplift of the Andes and related climate, such as in Bromeliaceae (Givnish et al., 2011). For the other two formation factors, in addition to the relevant cases mentioned above, the climate also involved the East Asian monsoon climate. For example, the formation and strengthening of the East Asian monsoon is inferred to be linked to the evolution of Theaceae (Yu et al., 2017) within Type III. Regarding land bridges, South America and Panama began to collide during 25–23 Ma (Farris et al., 2011), followed by the closing of the Panama seaway (Montes et al., 2015) to form the Panama land bridge, which also provided opportunities for the exchange of families between South and North America. As a whole, the formation of this distribution supertype associated with the New World tropics was primarily related to tectonic movement (especially, the subsequent fragmentation of Gondwana) and related climate (Table S4) and involved three migration routes (Figure 4B) and the first three formation models (Figure 4C).

Generally, the tropical element comprising 238 families primarily originated from Gondwana (28 families, 65.12% of tropical families; Figure 3C; Table S5), and its formation occurred between the Paleocene and Miocene, which was driven largely by tectonic movements and climate (tectonic movement‐mentioned and climate‐mentioned: 80.43% and 67.39% of families, respectively; Figure 4A; Table S4), the migration routes of the Paleogene and Neogene (Figure 4B), and all four formation models (Figure 4C).

## CONCLUSIONS

Based on the geographic distribution centers and origins of 429 global spermatophyte families, the SSES proposed here is represented by three floristic elements including six distribution supertypes, 18 distribution types, and 10 subtypes through unsupervised machine learning methods and multidisciplinary verification. The origin and formation of the SSES imply that similar geographic distributions of spermatophytes were shaped by their comparable formation processes and mechanisms at levels of floristic element, distribution supertype and type. This study proposes the formation models of the SSES and refines the main migration routes. The SSES and its formation offer new insights for comprehending the spatial evolution of global spermatophytes. Given the complexity of plant families, including their origins, migration, and even partial extinction of various genera, simultaneously analyzing the delineation and formation of various distributions results in a lack of detail. Further studies of plant distribution types at a fine‐scale, such as the genus level, are necessary for a better understanding the spatial evolution of plants.

## MATERIALS AND METHODS

### Construction of the SSES

The global spermatophytes included 429 families, 427 of which were extracted from Plants of the World (POTW) (Christenhusz et al., 2017), while the remaining families, Cephalotaxaceae (Yang et al., 2022b) and Tiganophytaceae (Swanepoel et al., 2020), were obtained from recently published papers (Swanepoel et al., 2020; Yang et al., 2022b). The phylogenetic relationships of these families were based on the revised version of APG IV (APG IV, 2016) and the recent gymnosperms' classification (Christenhusz et al., 2017, 2011; Yang et al., 2022b). To date, the most comprehensive dated phylogeny of these families, except for Cephalotaxaceae and Tiganophytaceae, was obtained from Smith and Brown (2018). Following previous studies (Wu et al., 2003a, 2006, 2010), the modern geographic distribution of families, with the inferred distribution center and most probable origin place, was used for our classification of distribution types. The classification of distribution subtypes primarily involves disjunct distribution and endemic distribution. The specific methods and steps for classifying the distribution types are as follows.

*K*‐means clustering (Macqueen, 1967), an iterative clustering algorithm, was used to preliminarily assign the distribution type based on the Euclidean distance (see “Methods” for a more detailed description in Supporting Information). The input subjected to K‐means clustering was one of two distribution categories for each investigated family grouped into three zones, namely the north temperate zone, the tropical zone and the south temperate zone, and seven continents (Table S8). The distribution categories 0 and 1 represent non‐distribution center and distribution center in the above regions, respectively. A continent is defined as the distribution center of a family when the number of native species of that family on this continent is greater than or equal to four percent of the total number of native species in the family. The four percent cutoff was derived from a statistical analysis of species distribution across 429 families across seven continents. Specifically, there are a total of 3,003 data points for the percentage of species of all families in all continents, and 1,648 data points (namely 55% of data points) were below four percent of the total number of native species in a family. Meanwhile, using a higher threshold would have disrupted the clustering results for several families during *K*‐means clustering analysis and the subsequent classification of their corresponding distribution types (see “Methods” for a more detailed description in Supporting Information). Therefore, this four percent cutoff was essential for accurately distinguishing distribution centers. The above‐mentioned numbers of native species of 429 families were all obtained using the R packages rWCVP v.1.2.4 and rWCVPdata v.0.4.1 (Brown et al., 2023) as of January 2024 (Table S9). A zone is defined as the distribution center of a family primarily determined by the description of the family's distribution in the POTW and the Angiosperm Phylogeny Website (http://www.mobot.org/MOBOT/research/APweb/), such as pantropical, north/south temperate, or cosmopolitan. The algorithm starts by initializing *n* random cluster centers based on the optimal number of clusters determined using the elbow method (Yuan and Yang, 2019) considering distortion, the distribution characteristics of the family, and previous divisions of distribution types (Wu, 2003; Wu et al., 2003b) in MATLAB v.R2021b. The clustering steps are repeated until the cluster centers stabilize or the algorithm reaches the predefined threshold of 10,000 iterations.

Combined with the classification principles of distribution types and subtypes, this study verified and adjusted the results of the K‐means clustering one by one based on the geographic distribution of each family, as well as the origin information of 96 families primarily obtained from the relevant literature in Web of Science as of 2023 (Table S5) to determine the final distribution type and subtype of 429 families (Table S1). To clarify the relationships among distribution types, hierarchical clustering was performed using the WPGMA and NMDS based on the Euclidean distances in the R package vegan v.2.6‐4 (Oksanen et al., 2022). The WPGMA was identified as the best‐performing clustering algorithm among seven clustering algorithms, including unweighted pair‐group method using arithmetic averages (UPGMA), unweighted pair‐group method using centroids (UPGMC), Ward's minimum variance D2 (ward.D2), single linkage (SL), complete lineage (CL), WPGMA, and weighted pair‐group method using centroids (WPGMC), based on the cophenetic correlation coefficient (Sokal and Rohlf, 1962) and Gower's distance (Gower, 1983) (Table S2). The input for the two clustering methods used in this study was one of two distribution categories for each distribution type grouped into three zones and seven continents (Table S10). The distribution categories 0 and 1 represent non‐distribution type center and distribution type center in the above regions, respectively. A region is defined as a distribution type center when the number of families with distribution center of a distribution type in this region is ≥ 56% of the total number of families in this distribution type. Based on the results of WPGMA and NMDS, we validated the classification of 429 families into three and six clusters in the *K*‐means clustering analysis, ultimately identifying three floristic elements and six distribution supertypes, and finalizing the construction of the SSES in reference to Wu et al. (2010) (Figure 2; Tables 1, S1).

Additionally, we tested whether there was a phylogenetic signal for the three floristic elements, six distribution supertypes, and 18 distribution types in spermatophyte families excluding the families Cephalotaxaceae and Tiganophytaceae using Blomberg's *K* (Blomberg et al., 2003) and Pagel's *λ* (Pagel, 1999) with the R function “phylosig” in the R package phytools v.2.1‐1 (Revell, 2012). The geographic distribution maps for 429 families were primarily constructed based on the POTW (Christenhusz et al., 2017), supplemented by additional references (Swanepoel et al., 2020; Yang et al., 2022a, 2022b) and online resources (http://www.mobot.org/mobot/research/apweb/welcome.html; https://powo.science.kew.org). These maps were generated as line layers in ArcGIS v.10.7, using the Natural Earth projection and World Geodetic System 1984 geographic coordinate system (Figures 1A, S3). Then, the pattern diagram for each distribution type, distribution supertype and floristic element was drawn as a polygon layer based on the primary range of their corresponding families (Figures 1A, 2, S3).

### Origins and formation information statistics

The stem age of 429 global spermatophyte families except for the Cephalotaxaceae and Tiganophytaceae (Table S3) was obtained from Smith and Brown (2018), and used to calculate the origin time for the distribution type, distribution supertype, and floristic element (Figures 3C, S6, S9). The origin locations of 96 spermatophyte families were collected from 18 distribution types except for Type XI (Table S5). Origin locations were categorized into three groups and 10 sources: Gondwana (Africa, Antarctica, Australia, South America, and uncertain regions of the Southern Hemisphere), Laurasia (Asia, North America, and uncertain regions of the Northern Hemisphere), and both (tropics and uncertain regions).

The formation time of the corresponding distribution types of 121 families was the time when the corresponding distribution types of these families were established based on their related fossil evidence and/or ancestral area reconstruction results, which were primarily obtained from relevant literature as of 2025 via Web of Science (Table S6). For a family with a disjunct distribution type, its formation time was the time of emergence of the disjunct distribution, such as the time of the split between tropical Africa *Cylicomorpha* and other tropical American taxa in Caricaceae of Type VII (Carvalho and Renner, 2012). For locally distributed (often on a single continent) or island‐endemic families, the formation time was always the crown or stem age of the family based on the related fossil evidence and/or ancestral area reconstruction results, such as the crown age of Stachyuraceae of Type XV (Feng et al., 2020). For a family with a wide distribution type, such as a global distribution or the north temperate distribution, the formation time was when the family occupied the corresponding distributional area, always the corresponding geological period, such as the time of the Hydrocharitaceae occupying a global distribution (Chen et al., 2012). Meanwhile, for a monotypic family, the formation time of its distribution type was always considered to be the diversification time or stem age of the family based on relevant fossil evidence and/or ancestral area reconstruction results, for example, the diversification time of Ginkgoaceae (Zhao et al., 2019). Additionally, the formation time of each distribution supertype and floristic element is determined based on the collective formation times of its constituent distribution types.

Formation factors for 89 families primarily obtained from the relevant literature as of 2025 via Web of Science were classified into four categories: climate, land bridge, tectonic movement, and the LDD (Table S4). Each formation factor was further classified into two categories: mentioned and unmentioned in the corresponding literature of a family. Additionally, the life forms and dispersal types (including animal, water, and wind dispersal) of 429 families were primarily gathered from relevant books (Mabberley, 2008; Christenhusz et al., 2017; Kubitzki, 1990–2018), electronic versions of global and regional floras such as Flora of China (http://www.iplant.cn/foc), Flora of North America (http://floranorthamerica.org/Main_Page), Flora of Australia (https://profiles.ala.org.au/opus/foa), African Plant Database (http://www.ville-ge.ch/musinfo/bd/cjb/africa/recherche.php?langue=an), and The World Flora Online (http://www.worldfloraonline.org/), and recent relevant literature (Table S3).

## CONFLICTS OF INTEREST

The authors declare no conflicts of interest.

## AUTHOR CONTRIBUTIONS

H.S., T.D., and H.C.W. conceived and supervised the study. X.H.H., J.T.C., X.J.Z., P.R.L., and J.Y.P. compiled the data. X.H.H., Q.L., N.L., and Y.T.W. performed the analyses. X.H.H., Q.S.F., and X.Y.K. generated the figures in the manuscript. X.H.H., H.S., and T.D. wrote the draft. J.B.L revised the manuscript. All authors read and approved the final manuscript.

## Supporting information

Additional Supporting Information may be found online in the supporting information tab for this article: http://onlinelibrary.wiley.com/doi/10.1111/jipb.13923/suppinfo

**Figure S1.** Optimized numbers of clusters *K* for the *K*‐means clustering.
**Figure S2A.** Distribution maps of three clusters for global spermatophyte families when *K* = 3 in *K*‐means clustering algorithm
**Figure S2B.** Distribution maps of six clusters for global spermatophyte families when *K* = 6 in *K*‐means clustering algorithm
**Figure S2C.** Distribution maps of 18 clusters for global spermatophyte families when *K* = 18 in *K*‐means clustering algorithm
**Figure S3.** Pattern diagrams and their base maps of 18 distribution types.
**Figure S4.** Ordination of non‐metric multidimensional scaling (NMDS) of 18 distribution types for global spermatophyte families based on Euclidean distances
**Figure S5.** Dendrogram of 18 distribution types for global spermatophyte families based on weighted pair‐group method using arithmetic averages (WPGMA).
**Figure S6.** Time and location of origin of six distribution supertypes.
**Figure S7.** Detailed origin locations of six distribution supertypes based on origin locations of 96 families of spermatophytes
**Figure S8.** Life form of 3 floristic elements and 18 distribution types of global spermatophyte families
**Figure S9.** Time and location of origin of 18 distribution types.
**Figure S10.** Detailed origin locations of 18 distribution types based on origin locations of 96 families of spermatophytes
**Table S1.** Detailed information of 3, 6, and 18 clusters for global spermatophyte families based on K‐means clustering algorithm.
**Table S2.** Performance of clustering algorithms for beta diversity (βsim) of distribution data of 18 distribution type
**Table S3.** Basic information, including dispersal type, life form, and stem age, of 429 spermatophyte families
**Table S4.** Formation factors of the 89 spermatophyte families
**Table S5.** Origin locations of 96 spermatophyte families
**Table S6.** Formation time of corresponding distribution type of 121 spermatophyte families
**Table S7.** Distribution types of 111 mixed annual and perennial herbaceous families
**Table S8.** Distribution situation of each family of global spermatophytes in the north temperate zone (including north frigid zone), the tropical zone, the south temperate zone (including south frigid zone) and seven continents.
**Table S9.** Percentage of species number of 429 spermatophyte families on seven continents to the total species number of corresponding families
**Table S10.** Distribution situation of the 18 distribution types in the north temperate zone, the tropical zone, the south temperate zone and seven continents.

## References

[jipb13923-bib-0001] APG (1998). An ordinal classification for the families of flowering plants. Ann. Mo. Bot. Gard. 85: 531–553.

[jipb13923-bib-0002] APG II (2003). An update of the Angiosperm Phylogeny Group classification for the orders and families of flowering plants: APG II. Bot. J. Linn. Soc. 141: 399–436.

[jipb13923-bib-0003] APG III (2009). An update of the angiosperm phylogeny group classification for the orders and families of flowering plants: APG III. Bot. J. Linn. Soc. 161: 105–121.

[jipb13923-bib-0004] APG IV (2016). An update of the Angiosperm Phylogeny Group classification for the orders and families of flowering plants: APG IV. Bot. J. Linn. Soc. 181: 1–20.

[jipb13923-bib-0005] Bansal, M. , Morley, R.J. , Nagaraju, S.K. , Dutta, S. , Mishra, A.K. , Selveraj, J. , Kumar, S. , Niyolia, D. , Harish, S.M. , Abdelrahim, O.B. , et al. (2022). Southeast Asian Dipterocarp origin and diversification driven by Africa‐India floristic interchange. Science 375: 455–460. 35084986 10.1126/science.abk2177

[jipb13923-bib-0006] Bergen, K.J. , Johnson, P.A. , de Hoop, M.V. , and Beroza, G.C. (2019). Machine learning for data‐driven discovery in solid Earth geoscience. Science 363: eaau0323. 30898903 10.1126/science.aau0323

[jipb13923-bib-0007] Birch, J.L. , and Keeley, S.C. (2013). Dispersal pathways across the Pacific: The historical biogeography of *Astelia* s.l. (Asteliaceae, Asparagales). J. Biogeogr. 40: 1914–1927.

[jipb13923-bib-0008] Birch, J.L. , and Kocyan, A. (2021). Biogeography of the monocotyledon astelioid clade (Asparagales): A history of long‐distance dispersal and diversification with emerging habitats. Mol. Phylogenet. Evol. 163: 107203. 33992785 10.1016/j.ympev.2021.107203

[jipb13923-bib-0009] Biswal, D.K. , Debnath, M. , Konhar, R. , Yanthan, S. , and Tandon, P. (2018). Phylogeny and biogeography of carnivorous plant family Nepenthaceae with reference to the Indian pitcher plant *Nepenthes khasiana* reveals an Indian subcontinent origin of *Nepenthes* colonization in South East Asia during the Miocene epoch. Front. Ecol. Evol. 6: 108.

[jipb13923-bib-0010] Blomberg, S.P. , Garland, T. , and Ives, A.R. (2003). Testing for phylogenetic signal in comparative data: Behavioral traits are more labile. Evolution 57: 717–745. 12778543 10.1111/j.0014-3820.2003.tb00285.x

[jipb13923-bib-0011] Brikiatis, L. (2016). Late Mesozoic North Atlantic land bridges. Earth Sci. Rev. 159: 47–57.

[jipb13923-bib-0012] Brown, M.J.M. , Walker, B.E. , Black, N. , Govaerts, R.H.A. , Ondo, I. , Turner, R. , and Lughadha, E.N. (2023). RWCVP: A companion R package for the World Checklist of Vascular Plants. New Phytol. 240: 1355–1365. 37289204 10.1111/nph.18919

[jipb13923-bib-0013] Brown, R.W. (1962). Paleocene flora of the Rocky Mountains and Great Plains. US Geol. Surv. Prof. Pap. 375: 1–119.

[jipb13923-bib-0014] Buerki, S. , Manning, J.C. , and Forest, F. (2013). Spatio‐temporal history of the disjunct family Tecophilaeaceae: A tale involving the colonization of three Mediterranean‐type ecosystems. Ann. Bot. 111: 361–373. 23277471 10.1093/aob/mcs286 PMC3579441

[jipb13923-bib-0015] Cai, L.M. , Xi, Z.X. , Peterson, K. , Rushworth, C. , Beaulieu, J. , and Davis, C.C. (2016). Phylogeny of Elatinaceae and the tropical Gondwanan origin of the Centroplacaceae (Malpighiaceae, Elatinaceae) clade. PLoS ONE 11: e0161881. 27684711 10.1371/journal.pone.0161881 PMC5042423

[jipb13923-bib-0016] Capo, M. , Perez, A. , and Antonio, J.A. (2022). An efficient Split‐Merge re‐start for the K‐means algorithm. IEEE Trans. Knowl. Data Eng. 34: 1618–1627.

[jipb13923-bib-0017] Carta, A. , Peruzzi, L. , and Ramírez‐Barahona, S. (2022). A global phylogenetic regionalization of vascular plants reveals a deep split between Gondwanan and Laurasian biotas. New Phytol. 233: 1494–1504. 34758121 10.1111/nph.17844 PMC9298788

[jipb13923-bib-0018] Carvalho, F.A. , and Renner, S.S. (2012). A dated phylogeny of the papaya family (Caricaceae) reveals the crop's closest relatives and the family's biogeographic history. Mol. Phylogenet. Evol. 65: 46–53. 22659516 10.1016/j.ympev.2012.05.019

[jipb13923-bib-0019] Chacón, J. , de Assis, M.C. , Meerow, A.W. , and Renner, S.S. (2012). From East Gondwana to Central America: Historical biogeography of the Alstroemeriaceae. J. Biogeogr. 39: 1806–1818.

[jipb13923-bib-0020] Chacón, J. , and Renner, S.S. (2014). Assessing model sensitivity in ancestral area reconstruction using Lagrange: A case study using the Colchicaceae family. J. Biogeogr. 41: 1414–1427.

[jipb13923-bib-0021] Chen, L.Y. , Chen, J.M. , Gituru, R.W. , and Wang, Q.F. (2012). Generic phylogeny, historical biogeography and character evolution of the cosmopolitan aquatic plant family Hydrocharitaceae. BMC Evol. Biol. 12: 30. 22404786 10.1186/1471-2148-12-30 PMC3317846

[jipb13923-bib-0022] Chen, L.Y. , Grimm, G.W. , Wang, Q.F. , and Renner, S.S. (2015). A phylogeny and biogeographic analysis for the Cape‐Pondweed family Aponogetonaceae (Alismatales). Mol. Phylogenet. Evol. 82: 111–117. 25462997 10.1016/j.ympev.2014.10.007

[jipb13923-bib-0023] Chen, L.Y. , Zhao, S.Y. , Mao, K.S. , Les, D.H. , Wang, Q.F. , and Moody, M.L. (2014). Historical biogeography of Haloragaceae: An out‐of‐Australia hypothesis with multiple intercontinental dispersals. Mol. Phylogenet. Evol. 78: 87–95. 24841538 10.1016/j.ympev.2014.04.030

[jipb13923-bib-0024] Chen, Z.D. , Manchester, S.R. , and Sun, H.Y. (1999). Phylogeny and evolution of the Betulaceae as inferred from DNA sequences, morphology, and paleobotany. Am. J. Bot. 86: 1168–1181. 10449397

[jipb13923-bib-0025] Christenhusz, M.J.M. , Fay, M.F. , and Chase, M.W. (2017). *Plants of the World: An Illustrated Encyclopedia of Vascular Plant Families*. Richmond: Royal Botanic Gardens.

[jipb13923-bib-0026] Christenhusz, M.J.M. , Reveal, J.L. , Farjon, A. , Gardner, M.F. , Mill, R.R. , and Chase, M.W. (2011). A new classification and linear sequence of extant gymnosperms. Phytotaxa 19: 55–70.

[jipb13923-bib-0027] Crowl, A.A. , Miles, N.W. , Visger, C.J. , Hansen, K. , Ayers, T. , Haberle, R. , and Cellinese, N. (2016). A global perspective on Campanulaceae: Biogeographic, genomic, and floral evolution. Am. J. Bot. 103: 233–245. 26865121 10.3732/ajb.1500450

[jipb13923-bib-0028] Darwin, C.R. (1991). On the Origin of Species by Means of Natural Selection, 11th ed. London: John Marry.

[jipb13923-bib-0029] Deng, J.B. , Drew, B.T. , Mavrodiev, E.V. , Gitzendanner, M.A. , Soltis, P.S. , and Soltis, D.E. (2015). Phylogeny, divergence times, and historical biogeography of the angiosperm family Saxifragaceae. Mol. Phylogenet. Evol. 83: 86–98. 25479063 10.1016/j.ympev.2014.11.011

[jipb13923-bib-0030] Donoghue, M.J. (2008). A phylogenetic perspective on the distribution of plant diversity. Proc. Natl. Acad. Sci. U.S.A. **105**: 11549–11555. 10.1073/pnas.0801962105 PMC2556411 18695216

[jipb13923-bib-0031] Dupin, J. , Matzke, N.J. , Särkinen, T. , Knapp, S. , Olmstead, R.G. , Bohs, L. , and Smith, S.D. (2017). Bayesian estimation of the global biogeographical history of the Solanaceae. J. Biogeogr. 44: 887–899.

[jipb13923-bib-0032] Ebersbach, J. , Muellner‐Riehl, A.N. , Michalak, I. , Tkach, N. , Hoffmann, M.H. , Röser, M. , Sun, H. , and Favre, A. (2017). In and out of the Qinghai‐Tibet Plateau: Divergence time estimation and historical biogeography of the large arctic‐alpine genus *Saxifraga* L. J. Biogeogr 44: 900–910.

[jipb13923-bib-0033] Farris, D.W. , Jaramillo, C. , Bayona, G. , Restrepo‐Moreno, S.A. , Montes, C. , Cardona, A. , Mora, A. , Speakman, R.J. , Glascock, M.D. , and Valencia, V. (2011). Fracturing of the Panamanian Isthmus during initial collision with South America. Geology 39: 1007–1010.

[jipb13923-bib-0034] Feng, Y. , Comes, H.P. , and Qiu, Y.X. (2020). Phylogenomic insights into the temporal‐spatial divergence history, evolution of leaf habit and hybridization in *Stachyurus* (Stachyuraceae). Mol. Phylogenet. Evol. 150: 106878. 32512196 10.1016/j.ympev.2020.106878

[jipb13923-bib-0035] Gillespie, R.G. , Baldwin, B.G. , Waters, J.M. , Fraser, C.I. , Nikula, R. , and Roderick, G.K. (2012). Long‐distance dispersal: A framework for hypothesis testing. Trends. Ecol. Evol. 27: 47–56. 22014977 10.1016/j.tree.2011.08.009

[jipb13923-bib-0036] Givnish, T.J. , Barfuss, M.H.J. , Van Ee, B. , Riina, R. , Schulte, K. , Horres, R. , Gonsiska, P.A. , Jabaily, R.S. , Crayn, D.M. , Smith, J.A.C. , et al. (2011). Phylogeny, adaptive radiation, and historical biogeography in Bromeliaceae: Insights from an eight‐locus plastid phylogeny. Am. J. Bot. 98: 872–895. 21613186 10.3732/ajb.1000059

[jipb13923-bib-0037] Givnish, T.J. , Spalink, D. , Ames, M. , Lyon, S.P. , Hunter, S.J. , Zuluaga, A. , Doucette, A. , Caro, G.G. , McDaniel, J. , Clements, M.A. , et al. (2016a). Orchid historical biogeography, diversification, Antarctica and the paradox of orchid dispersal. J. Biogeogr. 43: 1905–1916.

[jipb13923-bib-0038] Givnish, T.J. , Zuluaga, A. , Marques, I. , Lam, V.K.Y. , Gomez, M.S. , Iles, W.J.D. , Ames, M. , Spalink, D. , Moeller, J.R. , Briggs, B.G. , et al. (2016b). Phylogenomics and historical biogeography of the monocot order Liliales: Out of Australia and through Antarctica. Cladistics 32: 581–605. 34727673 10.1111/cla.12153

[jipb13923-bib-0039] Goncalves, D.J.P. , Shimizu, G.H. , Ortiz, E.M. , Jansen, R.K. , and Simpson, B.B. (2020). Historical biogeography of Vochysiaceae reveals an unexpected perspective of plant evolution in the Neotropics. Am. J. Bot. 107: 1004–1020. 32643810 10.1002/ajb2.1502

[jipb13923-bib-0040] Good, R. (1974). The Geography of Flowering Plants. 4th ed. New York: John Wiley & Sons Inc.

[jipb13923-bib-0041] Gower, J.C. (1983). Comparing classifications. In Numerical Taxonomy, J. Felsenstein , ed. New York: Springer (pp. 137–155).

[jipb13923-bib-0042] Graham, A. (2018). The role of land bridges, ancient environments, and migrations in the assembly of the North American flora. J. Syst. Evol. 56: 405–429.

[jipb13923-bib-0043] Grimsson, F. , Zetter, R. , Halbritter, H. , and Grimm, G.W. (2014). *Aponogeton* pollen from the Cretaceous and Paleogene of North America and West Greenland: Implications for the origin and palaeobiogeography of the genus. Rev. Palaeobot. Palynol. 200: 161–187. 24926107 10.1016/j.revpalbo.2013.09.005 PMC4047627

[jipb13923-bib-0044] Guo, S.X. (2000). Evolution, palaeobiogeography and palaeoecology of Eucommiaceae. Paleobotanist 49: 65–83.

[jipb13923-bib-0045] Herman, A.B. (2013). Albian‐Paleocene flora of the North Pacific: Systematic composition, Palaeofloristics and phytostratigraphy. Stratigr. Geol. Correl. 21: 689–747.

[jipb13923-bib-0046] Hikcey, L.J. (1977). Stratigraphy and paleobotany of the Golden Valley Formation (Early Tertiary) of western North Daktota. Geol. Soc. Am. Mem. 150: 1–183.

[jipb13923-bib-0047] Hickey, L.J. , and Peterson, R.K. (1978). Zingiberopsis, a fossil genus of the ginger family from Late Cretaceous to early Eocene sediments of Western Interior North America. Can. J. Bot. 56: 1136–1152.

[jipb13923-bib-0048] Hill, R.S. (2001). Biogeography, evolution and palaeoecology of *Nothofagus* (Nothofagaceae): The contribution of the fossil record. Aust. J. Bot. 49: 321–332.

[jipb13923-bib-0049] Huang, X.H. , Deng, T. , Chen, S.T. , Landis, J.B. , Lin, N. , Yang, Y. , Hu, G.W. , Zhou, Z. , Wang, Y.H. , Wang, H.C. , et al. (2021). Western Tethys origin, tropical Asia and tropical America disjunction in *Berchemia* and reinstatement of *Phyllogeiton* (Rhamneae, Rhamnaceae). Taxon 70: 515–525.

[jipb13923-bib-0050] Huang, X.H. , Deng, T. , Moore, M.J. , Wang, H.C. , Li, Z.M. , Lin, N. , Yusupov, Z. , Tojibaev, K.S. , Wang, Y.H. , and Sun, H. (2019). Tropical Asian Origin, boreotropical migration and long‐distance dispersal in Nettles (Urticeae, Urticaceae). Mol. Phylogenet. Evol. 137: 190–199. 31102687 10.1016/j.ympev.2019.05.007

[jipb13923-bib-0051] Hueffel, J.A. , Sperger, T. , Funes‐Ardoiz, I. , Ward, J.S. , Rissanen, K. , and Schoenebeck, F. (2021). Accelerated dinuclear palladium catalyst identification through unsupervised machine learning. Science 374: 1134–1140. 34822285 10.1126/science.abj0999

[jipb13923-bib-0052] Iglesias, A. , Artabe, A.E. , and Morel, E.M. (2011). The evolution of Patagonian climate and vegetation from the Mesozoic to the present. Biol. J. Linn. Soc. 103: 409–422.

[jipb13923-bib-0053] Iles, W.J. , Lee, C. , Sokoloff, D.D. , Remizowa, M.V. , Yadav, S.R. , Barrett, M.D. , Barrett, R.L. , Macfarlane, T.D. , Rudall, P.J. , and Graham, S.W. (2014). Reconstructing the age and historical biogeography of the ancient flowering‐plant family Hydatellaceae (Nymphaeales). BMC Evol. Biol. 14: 102. 24884487 10.1186/1471-2148-14-102 PMC4030046

[jipb13923-bib-0054] Janssens, S.B. , Knox, E.B. , Huysmans, S. , Smets, E.F. , and Merckx, V.S. (2009). Rapid radiation of *Impatiens* (Balsaminaceae) during Pliocene and Pleistocene: Result of a global climate change. Mol. Phylogenet. Evol. 52: 806–824. 19398024 10.1016/j.ympev.2009.04.013

[jipb13923-bib-0055] Janssens, S.B. , Vandelook, F. , De Langhe, E. , Verstraete, B. , Smets, E. , Vandenhouwe, I. , and Swennen, R. (2016). Evolutionary dynamics and biogeography of Musaceae reveal a correlation between the diversification of the banana family and the geological and climatic history of Southeast Asia. New Phytol. 210: 1453–1465. 26832306 10.1111/nph.13856 PMC5066818

[jipb13923-bib-0056] Jin, J.J. , Yang, M.Q. , Fritsch, P.W. , van Velzen, R. , Li, D.Z. , and Yi, T.S. (2020). Born migrators: Historical biogeography of the cosmopolitan family Cannabaceae. J. Syst. Evol. 58: 461–473.

[jipb13923-bib-0057] Kelly, A.E. , and Goulden, M.L. (2008). Rapid shifts in plant distribution with recent climate change. Proc. Natl. Acad. Sci. U.S.A. **105**: 11823–11826. 10.1073/pnas.0802891105 PMC2575286 18697941

[jipb13923-bib-0058] Kress, W.J. , and Specht, C.D. (2006). The evolutionary and biogeographic origin and diversification of the tropical monocot order Zingiberales. Aliso 22: 621–632.

[jipb13923-bib-0059] Kubitzki, K. (1990–2018). The Families and Genera of Vascular Plants. Berlin: Springer.

[jipb13923-bib-0060] Li, Y. , Smith, T. , Svetlana, P. , Yang, J. , Jin, J.H. , and Li, C.S. (2014). Paleobiogeography of the lotus plant (Nelumbonaceae: *Nelumbo*) and its bearing on the paleoclimatic changes. Palaeogeogr. Palaeoclimatol. Palaeoecol. 399: 284–293.

[jipb13923-bib-0061] Li, Z.Z. , Lehtonen, S. , Martins, K. , Wang, Q.F. , and Chen, J.M. (2022). Complete genus‐level plastid phylogenomics of Alismataceae with revisited historical biogeography. Mol. Phylogenet. Evol. 166: 107334. 34715331 10.1016/j.ympev.2021.107334

[jipb13923-bib-0062] Liu, B. , Le, C.T. , Barrett, R.L. , Nickrent, D.L. , Chen, Z.D. , Lu, L.M. , and Vidal‐Russell, R. (2018). Historical biogeography of Loranthaceae (Santalales): Diversification agrees with emergence of tropical forests and radiation of songbirds. Mol. Phylogenet. Evol. 124: 199–212. 29550535 10.1016/j.ympev.2018.03.010

[jipb13923-bib-0063] Liu, Y.P. , Xu, X.T. , Dimitrov, D. , Pellissier, L.C. , Borregaard, M.K. , Shrestha, N. , Su, X.Y. , Luo, A. , Zimmermann, N.E. , Rahbek, C. , et al. (2023). An updated floristic map of the world. Nat. Commun. 14: 2990. 37253755 10.1038/s41467-023-38375-y PMC10229591

[jipb13923-bib-0064] Luebert, F. , Couvreur, T.L.P. , Gottschling, M. , Hilger, H.H. , Miller, J.S. , and Weigend, M. (2017). Historical biogeography of Boraginales: West Gondwanan vicariance followed by long‐distance dispersal? J. Biogeogr. 44: 158–169.

[jipb13923-bib-0065] Mabberley, D.J. (2008). Mabberley's Plant‐Book: A Portable Dictionary of Plants, Their Classification and Uses. 3rd ed. Cambridge: Cambridge University Press.

[jipb13923-bib-0066] Macqueen, J. (1967). Some methods for classification and analysis of multivariate observations. In *Proceedings of the fifth Berkeley Symposium on Mathematical Statistics and Probability, Vol 1: Statistics*. Cam L.M.L., Neyman J., eds, (Berkeley: University of California Press), pp. 281–297.

[jipb13923-bib-0067] Mao, K.S. , Milne, R.I. , Zhang, L.B. , Peng, Y.L. , Liu, J.Q. , Thomas, P. , Mill, R.R. , and Renner, S.S. (2012). Distribution of living Cupressaceae reflects the breakup of Pangea. Proc. Natl. Acad. Sci. U.S.A. **109**: 7793–7798. 10.1073/pnas.1114319109 PMC3356613 22550176

[jipb13923-bib-0068] Maurin, K.J.L. , and Smissen, R.D. (2022). A dated phylogeny of Argophyllaceae (Asterales) is consistent with spread by long‐distance dispersal. N.Z. J. Bot. 60: 27–44.

[jipb13923-bib-0069] Mennes, C.B. , Lam, V.K.Y. , Rudall, P.J. , Lyon, S.P. , Graham, S.W. , Smets, E.F. , Merckx, V.S.F.T. , and Ebach, M. (2015). Ancient Gondwana break‐up explains the distribution of the mycoheterotrophic family Corsiaceae (Liliales). J. Biogeogr. 42: 1123–1136.

[jipb13923-bib-0070] Montes, C. , Cardona, A. , Jaramillo, C. , Pardo, A. , Silva, J.C. , Valencia, V. , Ayala, C. , Perez‐Angel, L.C. , Rodriguez‐Parra, L.A. , Ramirez, V. , et al. (2015). Middle Miocene closure of the Central American Seaway. Science 348: 226–229. 25859042 10.1126/science.aaa2815

[jipb13923-bib-0071] Müller, S. , Salomo, K. , Salazar, J. , Naumann, J. , Jaramillo, M.A. , Neinhuis, C. , Feild, T.S. , and Wanke, S. (2015). Intercontinental long‐distance dispersal of Canellaceae from the New to the Old World revealed by a nuclear single copy gene and chloroplast loci. Mol. Phylogenet. Evol. 84: 205–219. 25579657 10.1016/j.ympev.2014.12.010

[jipb13923-bib-0072] Nathan, R. (2006). Long‐distance dispersal of plants. Science 313: 786–788. 16902126 10.1126/science.1124975

[jipb13923-bib-0073] Nauheimer, L. , Metzler, D. , and Renner, S.S. (2012). Global history of the ancient monocot family Araceae inferred with models accounting for past continental positions and previous ranges based on fossils. New Phytol. 195: 938–950. 22765273 10.1111/j.1469-8137.2012.04220.x

[jipb13923-bib-0074] Nie, Z.L. , Funk, V.A. , Meng, Y. , Deng, T. , Sun, H. , and Wen, J. (2016). Recent assembly of the global herbaceous flora: Evidence from the paper daisies (Asteraceae: Gnaphalieae). New Phytol. 209: 1795–1806. 26528674 10.1111/nph.13740

[jipb13923-bib-0075] Oksanen, J. , Simpson, G.L. , Blanchet, F.G. , Kindt, R. , Legendre, P. , Minchin, P.R. , O'Hara, R.B. , Solymos, P. , Stevens, M.H.H. , Szoecs, E. , et al. (2022). *vegan: Community Ecology Package*. R package version 2.6‐4. https://github.com/vegandevs/vegan

[jipb13923-bib-0076] Pagel, M. (1999). Inferring the historical patterns of biological evolution. Nature 401: 877–884. 10553904 10.1038/44766

[jipb13923-bib-0077] Parmesan, C. , and Yohe, G. (2003). A globally coherent fingerprint of climate change impacts across natural systems. Nature 421: 37–42. 12511946 10.1038/nature01286

[jipb13923-bib-0078] Peng, H.W. , Xiang, K.L. , Erst, A.S. , Lian, L. , Ortiz, R.D.C. , Jabbour, F. , Chen, Z.D. , and Wang, W. (2023). A complete genus‐level phylogeny reveals the Cretaceous biogeographic diversification of the poppy family. Mol. Phylogenet. Evol. 181: 107712. 36693534 10.1016/j.ympev.2023.107712

[jipb13923-bib-0079] Pillon, Y. , Hopkins, H.C.F. , Maurin, O. , Epitawalage, N. , Bradford, J. , Rogers, Z.S. , Baker, W.J. , and Forest, F. (2021). Phylogenomics and biogeography of Cunoniaceae (Oxalidales) with complete generic sampling and taxonomic realignments. Am. J. Bot. 108: 1181–1200. 34278558 10.1002/ajb2.1688 PMC8361763

[jipb13923-bib-0080] Popp, M. , Mirre, V. , and Brochmann, C. (2011). A single Mid‐Pleistocene long‐distance dispersal by a bird can explain the extreme bipolar disjunction in crowberries (*Empetrum*). Proc. Natl. Acad. Sci. U.S.A. **108**: 6520–6525. 10.1073/pnas.1012249108 PMC3081031 21402939

[jipb13923-bib-0081] Qi, X.S. , Chen, C. , Comes, H.P. , Sakaguchi, S. , Liu, Y.H. , Tanaka, N. , Sakio, H. , and Qiu, Y.X. (2012). Molecular data and ecological niche modelling reveal a highly dynamic evolutionary history of the East Asian Tertiary relict *Cercidiphyllum* (Cercidiphyllaceae). New Phytol. 196: 617–630. 22845876 10.1111/j.1469-8137.2012.04242.x

[jipb13923-bib-0082] Qi, Z.C. , Li, P. , Wu, J.J. , Gamisch, A. , Yang, T. , Zhao, Y.P. , Xu, W.Q. , Chen, S.C. , Cameron, K.M. , Qiu, Y.X. , et al. (2023). Climatic niche evolution in Smilacaceae (Liliales) drives patterns of species diversification and richness between the Old and New World. J. Syst. Evol. 61: 733–747.

[jipb13923-bib-0083] Raven, P.H. (1972). Plant species disjunctions: A summary. Ann. Mo. Bot. Gard. 59: 234–246.

[jipb13923-bib-0084] Reguero, M.A. , Marenssi, S.A. , and Santillana, S.N. (2002). Antarctic Peninsula and South America (Patagonia) Paleogene terrestrial faunas and environments: Biogeographic relationships. Palaeogeogr. Palaeoclimatol. Palaeoecol. 179: 189–210.

[jipb13923-bib-0085] Renner, S.S. , Barreda, V.D. , Telleria, M.C. , Palazzesi, L. , and Schuster, T.M. (2020). Early evolution of Coriariaceae (Cucurbitales) in light of a new early Campanian (ca. 82 Mya) pollen record from Antarctica. Taxon 69: 87–99.

[jipb13923-bib-0086] Revell, L.J. (2012). phytools: An R package for phylogenetic comparative biology (and other things). Methods Ecol. Evol. 3: 217–223.

[jipb13923-bib-0087] Ruddiman, W.F. (2014). Earth's Climate: Past and Future. New York: W. H. Freeman and Company.

[jipb13923-bib-0088] Rutschmann, F. , Eriksson, T. , Schonenberger, J. , and Conti, E. (2004). Did Crypteroniaceae really disperse out of india? Molecular dating evidence from rbcL, ndhF, and rpl16 intron sequences. Int. J. Plant Sci. 165: S69–S83.

[jipb13923-bib-0089] Sanmartin, I. , and Ronquist, F. (2004). Southern Hemisphere biogeography inferred by event‐based models: Plant versus animal patterns. Syst. Biol. 53: 216–243. 15205050 10.1080/10635150490423430

[jipb13923-bib-0090] Schenck, H. (1886). Die Biologie der Wassergewächse 162. Bonn: Cohen and Sohn (Fr. Cohen).

[jipb13923-bib-0091] Scotese, C.R. (2001). *Atlas of Earth history, Volume 1, Paleogeography*. Arlington: Paleomap Project.

[jipb13923-bib-0092] Smith, A.G. , Smith, D.G. , Funnell, B.M. (2004). Atlas of Cenozoic and Mesozoic Coastlines. Cambridge: Cambridge University Press.

[jipb13923-bib-0093] Smith, S.A. , and Brown, J.W. (2018). Constructing a broadly inclusive seed plant phylogeny. Am. J. Bot. 105: 302–314. 29746720 10.1002/ajb2.1019

[jipb13923-bib-0094] Snead, R.G. (1969). Microfloral diagnosis of the Creaceous‐Tertiary boundary. Central Alberta. Res. Coun. Alberta Bull. 25: 1–148.

[jipb13923-bib-0095] Sokal, R.R. , and Rohlf, F.J. (1962). The comparison of dendrograms by objective methods. Taxon 11: 33–40.

[jipb13923-bib-0096] Spalink, D. , Drew, B.T. , Pace, M.C. , Zaborsky, J.G. , Starr, J.R. , Cameron, K.M. , Givnish, T.J. , and Sytsma, K.J. (2016). Biogeography of the cosmopolitan sedges (Cyperaceae) and the area‐richness correlation in plants. J. Biogeogr. 43: 1893–1904.

[jipb13923-bib-0097] Swanepoel, W. , Chase, M.W. , Christenhusz, M.J.M. , Maurin, O. , Forest, F. , and Van Wyk, A.E.E. (2020). From the frying pan: An unusual dwarf shrub from Namibia turns out to be a new brassicalean family. Phytotaxa 439: 171–185.

[jipb13923-bib-0098] Takhtajan, A. (1986). Floristic Regions of the World. Berkeley: University of California Press.

[jipb13923-bib-0099] Thomas, D.C. , Chatrou, L.W. , Stull, G.W. , Johnson, D.M. , Harris, D.J. , Thongpairoj, U.S. , and Saunders, R.M.K. (2015). The historical origins of palaeotropical intercontinental disjunctions in the pantropical flowering plant family Annonaceae. Perspect. Plant Ecol. Evol. Syst. 17: 1–16.

[jipb13923-bib-0100] Thorne, R.F. (1972). Major disjunctions in the geographic ranges of seed plants. Q. Rev. Biol. 47: 365–411.

[jipb13923-bib-0101] Tiffney, B.H. , and Manchester, S.R. (2001). The use of geological and paleontological evidence in evaluating plant phylogeographic hypotheses in the Northern Hemisphere tertiary. Int. J. Plant Sci. 162: S3–S17.

[jipb13923-bib-0102] van Balgooy, M.M.J. (1969). A study on the diversity of island floras. Blumea 17: 139–178.

[jipb13923-bib-0103] Vester, H. (1940). Die areale und arealtypen der angiospermen‐familien. Bot. Arch. 41: 203–577.

[jipb13923-bib-0104] Villaverde, T. , Larridon, I. , Shah, T.R. , Fowler, R.M. , Chau, J.H. , Olmstead, R.G. , and Sanmartín, I. (2023). Phylogenomics sheds new light on the drivers behind a long‐lasting systematic riddle: The figwort family Scrophulariaceae. New Phytol. 240: 1601–1615. 36869601 10.1111/nph.18845

[jipb13923-bib-0105] Vinnersten, A. , and Bremer, K. (2001). Age and biogeography of major clades in Liliales. Am. J. Bot. 88: 1695–1703. 21669704

[jipb13923-bib-0106] Wallace, A.R. (1876). *The Geographical Distribution of Animals*. New York: Harper and Brothers.

[jipb13923-bib-0107] Wang, W. , Xiang, X.G. , Xiang, K.L. , Ortiz, R.D. , Jabbour, F. , and Chen, Z.D. (2020). A dated phylogeny of Lardizabalaceae reveals an unusual long‐distance dispersal across the Pacific Ocean and the rapid rise of East Asian subtropical evergreen broadleaved forests in the late Miocene. Cladistics 36: 447–457. 34618951 10.1111/cla.12414

[jipb13923-bib-0108] Wen, J. , Ickert‐Bond, S. , Nie, Z.L. , and Li, R. (2010). Timing and modes of evolution of eastern Asian–North American biogeographic disjunctions in seed plants. In *Darwin's Heritage Today: Proceedings of the Darwin 200 Beijing International Conference*. Long, M., Gu, H., Zhou, Z., eds, (Beijing: Higher Education Press), pp. 252–269.

[jipb13923-bib-0109] Wu, Z.Y. (1965). The tropical floristic affinity of the flora of China. Chin. Sci. Bull. 16: 25–33.

[jipb13923-bib-0110] Wu, Z.Y. (1991). The areal‐types of Chinese genera of seed plants. Acta Bot. Yunnan. 13: 1–139.

[jipb13923-bib-0111] Wu, Z.Y. (2003). Revision of the areal‐types of the world families of seed plants. Acta Bot. Yunnan. 25: 535–538.

[jipb13923-bib-0112] Wu, Z.Y. , Liu, J. , Provan, J. , Wang, H. , Chen, C.J. , Cadotte, M.W. , Luo, Y.H. , Amorim, B.S. , Li, D.Z. , and Milne, R.I. (2018). Testing Darwin's transoceanic dispersal hypothesis for the inland nettle family (Urticaceae). Ecol. Lett. 21: 1515–1529. 30133154 10.1111/ele.13132

[jipb13923-bib-0113] Wu, Z.Y. , Lu, A.M. , Tang, Y.C. , Chen, Z.D. , Li, D.Z. (2003a). The Families and Genera of Angiosperms in China: A Comprehensive Analysis. Beijing: Science Press.

[jipb13923-bib-0114] Wu, Z.Y. , Sun, H. , Zhou, Z.K. , Li, D.Z. , Peng, H. (2010). Floristics of Seed Plants from China. Beijing: Science Press.

[jipb13923-bib-0115] Wu, Z.Y. , Zhou, Z.K. , Li, D.Z. , Peng, H. , and Sun, H. (2003b). The areal‐types of the world families of seed plants. Acta Bot. Yunnan. 25: 245–257.

[jipb13923-bib-0116] Wu, Z.Y. , Zhou, Z.K. , Sun, H. , Li, D.Z. , and Peng, H. (2006). *The Areal‐Types of Seed Plants and Their Origin and Differentiation*. Kunming: Yunnan Science & Technology Press.

[jipb13923-bib-0117] Wulff, E.V. (1943). An Introduction to Historical Plant Geography. Massachusetts: Chronica Botanica Company.

[jipb13923-bib-0118] Yang, L.E. , Sun, L. , Peng, D.L. , Chen, G.J. , Sun, H. , and Nie, Z.L. (2022a). The significance of recent diversification in the Northern Hemisphere in shaping the modern global flora revealed from the herbaceous tribe of Rubieae (Rubiaceae). Mol. Phylogenet. Evol. 177: 107628. 36096462 10.1016/j.ympev.2022.107628

[jipb13923-bib-0119] Yang, T. , Lu, L.M. , Wang, W. , Li, J.H. , Manchester, S.R. , Wen, J. , and Chen, Z.D. (2018). Boreotropical range expansion and long‐distance dispersal explain two amphi‐Pacific tropical disjunctions in Sabiaceae. Mol. Phylogenet. Evol. 124: 181–191. 29548980 10.1016/j.ympev.2018.03.005

[jipb13923-bib-0120] Yang, Y. , Ferguson, D.K. , Liu, B. , Mao, K.S. , Gao, L.M. , Zhang, S.Z. , Wan, T. , Rushforth, K. , and Zhang, Z.X. (2022b). Recent advances on phylogenomics of gymnosperms and an updated classification. Plant Divers. 44: 340–350. 35967253 10.1016/j.pld.2022.05.003 PMC9363647

[jipb13923-bib-0121] Yu, X.Q. , Gao, L.M. , Soltis, D.E. , Soltis, P.S. , Yang, J.B. , Fang, L. , Yang, S.X. , and Li, D.Z. (2017). Insights into the historical assembly of East Asian subtropical evergreen broadleaved forests revealed by the temporal history of the tea family. New Phytol. 215: 1235–1248. 28695680 10.1111/nph.14683

[jipb13923-bib-0122] Yuan, C.H. , and Yang, H.T. (2019). Research on K‐value selection method of K‐means clustering algorithm. J 2: 226–235.

[jipb13923-bib-0123] Zachos, J.C. , Dickens, G.R. , and Zeebe, R.E. (2008). An early Cenozoic perspective on greenhouse warming and carbon‐cycle dynamics. Nature 451: 279–283. 18202643 10.1038/nature06588

[jipb13923-bib-0124] Zhang, L.B. , Simmons, M.P. , and Renner, S.S. (2007). A phylogeny of Anisophylleaceae based on six nuclear and plastid loci: Ancient disjunctions and recent dispersal between South America, Africa, and Asia. Mol. Phylogenet. Evol. 44: 1057–1067. 17433719 10.1016/j.ympev.2007.03.002

[jipb13923-bib-0125] Zhang, Q.Y. , Deng, M. , Bouchenak‐Khelladi, Y. , Zhou, Z.K. , Hu, G.W. , and Xing, Y.W. (2022a). The diversification of the northern temperate woody flora—A case study of the Elm family (Ulmaceae) based on phylogenomic and paleobotanical evidence. J. Syst. Evol. 60: 728–746.

[jipb13923-bib-0126] Zhang, Q.Y. , Ree, R.H. , Salamin, N. , Xing, Y.W. , and Silvestro, D. (2022b). Fossil‐informed models reveal a boreotropical origin and divergent evolutionary trajectories in the walnut family (Juglandaceae). Syst. Biol. 71: 242–258. 10.1093/sysbio/syab030 PMC8677545 33964165

[jipb13923-bib-0127] Zhang, Z.S. , Ramstein, G. , Schuster, M. , Li, C. , Contoux, C. , and Yan, Q. (2014). Aridification of the Sahara desert caused by Tethys Sea shrinkage during the Late Miocene. Nature 513: 401–404. 25230661 10.1038/nature13705

[jipb13923-bib-0128] Zhao, Y.P. , Fan, G.Y. , Yin, P.P. , Sun, S. , Li, N. , Hong, X.N. , Hu, G. , Zhang, H. , Zhang, F.M. , Han, J.D. , et al. (2019). Resequencing 545 ginkgo genomes across the world reveals the evolutionary history of the living fossil. Nat. Commun. 10: 4201. 31519986 10.1038/s41467-019-12133-5 PMC6744486

[jipb13923-bib-0129] Zhou, Z.K. , and Momohara, A. (2005). Fossil history of some endemic seed plants of East Asia and its phytogeographical significance. Acta Bot. Yunnan. 27: 449–470.

[jipb13923-bib-0130] Zhu, Y.P. , Wen, J. , Zhang, Z.Y. , and Chen, Z.D. (2006). Evolutionary relationships and diversification of Stachyuraceae based on sequences of four chloroplast markers and the nuclear ribosomal its region. Taxon 55: 931–940.

