# Supplementary material for: Spatial distribution patterns and formation of global spermatophytes
Source: J Integr Plant Biol. 2025 May 23;67(10):2668–85. doi: 10.1111/jipb.13923 (PMC12498069; doi:10.1111/jipb.13923)
Supplement: Supplementary file 1 — Figure S1. Optimized numbers of clusters K for the K‐means clustering. Figure S2A. Distribution maps of three clusters for global spermatophyte families when K = 3 in K‐means clustering algorithm Figure S2B. Distribution maps of six clusters for global spermatophyte families when K = 6 in K‐means clustering algorithm Figure S2C. Distribution maps of 18 clusters for global spermatophyte families when K = 18 in K‐means clustering algorithm Figure S3. Pattern diagrams and their base maps of 18 distribution types. Figure S4. Ordination of non‐metric multidimensional scaling (NMDS) of 18 distribution types for global spermatophyte families based on Euclidean distances Figure S5. Dendrogram of 18 distribution types for global spermatophyte families based on weighted pair‐group method using arithmetic averages (WPGMA). Figure S6. Time and location of origin of six distribution supertypes. Figure S7. Detailed origin locations of six distribution supertypes based on origin locations of 96 families of spermatophytes Figure S8. Life form of 3 floristic elements and 18 distribution types of global spermatophyte families Figure S9. Time and location of origin of 18 distribution types. Figure S10. Detailed origin locations of 18 distribution types based on origin locations of 96 families of spermatophytes Table S1. Detailed information of 3, 6, and 18 clusters for global spermatophyte families based on K‐means clustering algorithm. Table S2. Performance of clustering algorithms for beta diversity (βsim) of distribution data of 18 distribution type Table S3. Basic information, including dispersal type, life form, and stem age, of 429 spermatophyte families Table S4. Formation factors of the 89 spermatophyte families Table S5. Origin locations of 96 spermatophyte families Table S6. Formation time of corresponding distribution type of 121 spermatophyte families Table S7. Distribution types of 111 mixed annual and perennial herbaceous families Table S8. Distribution situation of each [file JIPB-67-2668-s001.docx]

**SUPPORTING INFORMATION**

**Spatial distribution patterns and formation of global spermatophytes**

**This PDF file includes:**

Supplementary Methods, Results and Discussion

Figures S1 to S10

Tables S1 to S10

**SUPPLEMENTARY METHODS**

**K-means clustering**

K-means clustering (Macqueen, 1967) is an iterative clustering algorithm that randomly selects K objects as the initial cluster centers, then calculates the distance between each object and the cluster centers, and finally assigns each object to the nearest cluster center based on distance. The input subjected to K-means clustering consisted of one of two distribution categories for each studied family grouped into three zones and seven continents (Table S8). The distribution categories 0 and 1 represent non-distribution center and distribution center in the regions mentioned above, respectively. A continent is defined as the distribution center of a family when the number of native species of that family on a continent was greater than or equal to four percent of the total number of native species in the family. The four percent cutoff was derived from statistical analysis of species distributions across 429 families on seven continents. Specifically, there are total 3,003 data points for the percentage of species of all families in all continents, and 1,648 data points (namely 55% of data points) were below four percent of the total number of native species in a family. Therefore, this threshold was essential for distinguishing distribution centers. Meanwhile, using a higher threshold would have disrupted the clustering results for several families during K-means clustering analysis and the subsequent classification of their corresponding distribution types. For example, within the cluster 1 of 18 clusters during K-means clustering analysis, some families such as Ranunculaceae and Scrophulariaceae have less than four percent of the total number of native species on two continents, with some continents showing exactly four percent. Thus, when a higher threshold is used in the K-means clustering analysis, these families are unlikely to be assigned to cluster 1, which contradicts their classification as the typical families of distribution Type I. Similar situations are observed in families such as Passifloraceae and Ochnaceae (Type II), Musaceae and Pedaliaceae (Type IV), Adoxaceae (Type XII), and Montiaceae (Type XVIII). These examples demonstrate the necessity of the four percent threshold for accurately classifying distribution centers. The numbers of native species of 429 family were all obtained using the R package rWCVP v.1.2.4 and rWCVPdata v.0.4.1 (Brown et al., 2023) (Table S9); whether a zone is the distribution center of a family is primarily determined by the description of the family's distribution in the POTW and the Angiosperm Phylogeny Website (http://www.mobot.org/MOBOT/research/APweb/), such as pantropical, north/south temperate, or cosmopolitan. The algorithm starts by initializing *n* random cluster centers based on the optimal number of clusters determined using elbow method (Yuan and Yang, 2019) considering distortion, the distribution characteristics of the family, and the previous divisions of distribution types (Wu, 2003; Wu et al., 2003b) in MATLAB v.R2021b. Secondly, the data objects are classified one by one into clusters represented by the nearest cluster center by calculating the Euclidean distance between each data object (*p*) and each cluster center ($m_{i}$) in the dataset. The sum of squared errors criterion function E is calculated according to Equation (1). Thirdly, the mean of all data objects in each cluster is calculated as the new center of each cluster, and E is calculated based on the new cluster centers, which is used to update the cluster centers. The clustering steps are then repeated until the cluster centers stabilize or the algorithm reaches the predefined threshold of 10,000 iterations, the process proceeds to the next step, otherwise it returns to step 1. Ultimately, the clustering results that meet the termination conditions are output.

$$E=\sum_{i=1}^{n} \sum_{p\in X_{i}} \left\| p-m_{i} \right\|^{2} (1)$$

**SUPPLEMENTARY RESULTS AND DISCUSSION**

**Formation of 18 distribution types**

**Young cosmopolitan element predominantly driven by the LDD and tectonic movement**

Same to the results and discussion part.

**Ancient temperate element largely influenced by climate, land bridge, and tectonic movement**

**Distribution supertype 6 of north and south temperate regions**

With respect to Type XVIII characterized by a disjunct distribution between the north and south temperate zones and comprising16 families, it was estimated as a relatively ancient distribution type among 18 types in origin time (ranking fifteenth in terms of the median stem age; Figure S9; Table S3). This distribution type originated mainly from Laurasia (Figure S9; Table S5), and formed between the Jurassic and Miocene (especially the Tertiary; Figure 3A; Table S6). Tectonic movement was highlighted as a significant factor for formation of this distribution type, especially for its ancient families. For example, Cupressaceae was inferred to originate from the intact Pangea in the Triassic and subsequently formed two subfamilies with the separation of Laurasia and Gondwana, and three further intercontinental disjunctions coincided with or immediately followed the breakup of Pangea (Mao et al., 2012) (indicating the second or third formation model; Figures 4B, 4C). Climate and land bridges also played a significant role in the formation of this distribution type, such as the BLB for Melanthiaceae (Givnish et al., 2016b), Papaveraceae (Peng et al., 2023) and Saxifragaceae (Deng et al., 2015; Ebersbach et al., 2017), as well as the both BLB and NALBs for Betulaceae (Chen et al., 1999) (Figure 4B). In terms of climate, the deterioration of the climate in the Oligocene and the Quaternary glaciation likely drove the southward migration of the Betulaceae into the Southern Hemisphere (Chen et al., 1999). To sum up, the formation of this distribution type largely involved tectonic movement, climate and land bridges (Table S4), the migration routes since the Cretaceous (Figure 4B), and the second and third formation models (Figure 4C).

Type XIV characterized by a pan-Mediterranean distribution and comprising 14 families was estimated as the youngest of 18 distribution types in origin time (ranking first in terms of the median stem age; Figure S9; Table S3), and formed mainly during the Cenozoic (Figure 3A; Table S6). Two families originated in Asia and Antarctica, respectively. The formation of this distribution type was influenced by the Mediterranean climate characterized by less rainfall at higher temperatures and more rainfall at lower temperatures, and partly related to the tectonic movements especially the evolution of the Tethys Sea (Wu et al., 2006), as observed in the Frankeniaceae and Tecophilaeaceae (Buerki et al., 2013). The formation of this type was also inferred to be associated with other factors, such as the land bridge (Renner et al., 2020) and the LDD (Martin-Rodriguez et al., 2020). Additionally, based on the endemic distribution and distribution centers, subtype XIV.I (Mediterranea, West Asia to Central Asia) was proposed within Type XIV. This subtype formed during the Cenozoic (Figure 3A; Table S6). For example, Biebersteiniaceae of this distribution subtype was inferred to partially originate in the eastern Tibetan Plateau in the late Paleogene and then migrate westward to form its current distribution (Muellner et al., 2007) representing the first formation model. The formation of this subtype appeared to be influenced by the collision of the Indian Plate and the Eurasian Plate and the uplift of related regions, and also related to the retreat of the Tethys Sea (Wu et al., 2006). In general, the formation of this distribution type largely involved the migration routes of the Paleogene and Neogene (Figure 4B), climate and tectonic movement (Table S4), and the first three formation models (Figure 4C).

**Distribution supertype 4 of south temperate region**

Same to the results and discussion part.

**Distribution supertype 5 of** **north temperate region**

Regarding Type XII characterized by the north temperate distribution and comprising 20 families, it was estimated as a relatively ancient distribution type in origin time (ranking sixteenth in terms of the median stem age; Figure S9; Table S3). This distribution type originated mainly from Laurasia (especially North America; Figures S9, S10; Table S5), and formed mainly from the Late Cretaceous to the Miocene (Figure 3A; Table S6). Several groups within this distribution type have expanded their range from their place of origin to the current northern temperate zone, overcoming several geographic barriers such as the Bering Strait, the Turgai Strait, the North Atlantic, the early Tertiary inland trench, the mid-Tertiary arid zone of North America, the Chinese arid zone, and the Central Asian arid region (Tiffney and Manchester, 2001). In Type XII, Liliaceae was inferred to be originally North American and then likely migrate from North America to Eurasia by the BLB overcoming the Bering Strait barrier (Givnish et al., 2016b; Vinnersten and Bremer, 2001) (Figure 4B), while crown group Juglandaceae was estimated to be boreotropical origin from the Middle to Late Cretaceous and then achieved its contemporary distribution via dispersal across the NALBs, facilitated by the retreat of the Turgai seaway, the closure of the Tethys Ocean within Eurasia, and climate change (Zhang et al., 2022b). Meanwhile, the diversification in Diapensiaceae was likely shaped by both large‐scale biogeographic factors such as vicariance, and divergence in ecological niche, with the shift to a colder temperatures likely promoting the differentiation and range expansion of some taxa (Gaynor et al., 2020). Generally, the formation of this distribution type largely involved land bridges and tectonic movement (Table S4), the migration routes since the Cretaceous (Figure 4B), and the first formation model (Figure 4C).

With respect to Type XV characterized by East Asian distribution and comprising 11 families, it was estimated as a relatively ancient distribution type in origin time (ranking twelfth in terms of the median stem age; Figure S9; Table S3). This distribution type originated mainly from Laurasia (Figure S9; Table S5), and formed mainly during the Cenozoic (Figure 3A; Table S6). For instance, Stachyuraceae (Feng et al., 2020; Zhu et al., 2006) is inferred to originate in the Northern Hemisphere of the Late Cretaceous or early Tertiary, and then form the current disjunct distribution between Japan and mainland Asian under the joint influence of the mountain-building movements such as the uplift of the Qinghai-Tibet Plateau, and paleoclimates such as global cooling and the strengthening of the East Asian summer monsoon. In combination with the fossil distribution of Eucommiaceae (Brown, 1962; Guo, 2000; Zhou and Momohara, 2005) and Cercidiphyllaceae (Herman, 2013; Qi et al., 2012), some families of this distribution type were likely widely distributed in the Northern Hemisphere in the early stages of their evolution, and then only survived in East Asia due to subsequent climate deterioration (Table S6). Based on its endemic distribution, Sciadopityaceae with one genus and species, originating 90.53 Ma, is proposed to be classified in the subtype XV.I (endemic to Japan). To sum up, the formation of this distribution type largely involved climate and tectonic movement (Table S4), the migration routes of the Paleogene and Neogene (Figure 4B), and the fourth formation model (Figure 4C). In terms of the Type XVI characterized by North America distribution and comprising 9 families, it was estimated as a relatively ancient distribution type in origin time (ranking seventeenth in terms of the median stem age; Figure S9; Table S3) and formed mainly during the Tertiary (Figure 3A; Table S6). Crossosomataceae of this distribution type was inferred to originate in the northern hemisphere prior to the Tertiary, and then likely experienced drastic climatic changes, including drying and glaciation and adapted to the xeric environment in western North America (Zhu et al., 2006). Meanwhile, the reduction in direct overland continuity between North and South America during the Middle Oligocene was also inferred to promote the formation of the current distribution of Sarraceniaceae (Ellison et al., 2012). Therefore, the formation of the Type XVI mainly involved climate, tectonic movement and land bridges (Table S4), and the fourth formation model (Figure 4C).

With respect to Type XIII characterized by the disjunct distribution between East Asia and North America and comprising 12 families, it was estimated as the oldest of 18 distribution types in origin time (ranking eighteenth in terms of the median stem age; Figure S9; Table S3). This distribution type originated always from Laurasia (Figure S9; Table S5), and formed mainly from the Oligocene to the Pleistocene (Figure 3A; Table S6). Based on fossil evidence, the origin of Nelumbonaceae (Li et al., 2014) of this distribution type could be traced back to the mid-latitudes of Laurasia in the Early Cretaceous, then expanded its distribution range, even to the northernmost part of the northern hemisphere in the Eocene, but became extinct in Europe and Central Asia due to Pleistocene glacial periods and finally formed the current disjunct distribution. The NALBs or BLB were inferred to play a role in the evolution of this family, and the cooling climate and increased seasonality in the Eocene East Asia likely promoted the origin of tubers and the differentiation of the ecotypes in lotus (Figure 4B). Moreover, the Penthoraceae were inferred to originate in Australia during the Late Cretaceous, and then spread from south to north forming the current disjunct distribution (Chen et al., 2014), indicating the existence of other migration routes of this distribution type. Overall, the formation of this distribution type largely involved climate and land bridges (Table S4), the migration routes of the Paleogene and Neogene (Figure 4B), and the second or third formation model (Figure 4C).

**Tropical element mainly shaped by climate and tectonic movement**

**Distribution supertype 3 of the** **Old World tropics**

Type VIII characterized by tropical Asia distribution and comprising 8 families was inferred as a relatively young distribution type in origin time (ranking second in terms of the median stem age; Figure S9; Table S3) and formed mainly from the Paleocene to the Miocene (Figure 3A; Table S6). Two families originated in Gondwana and the tropics, respectively (Figure S9; Table S5). Crypteroniaceae was inferred to originate in Gondwana during the early to Middle Cretaceous, then diversify on the Indian plate, and subsequently migrated/dispersed to Southeast Asia (Rutschmann et al., 2004), indicating the role of the Indian plate in the evolution of this family. Similarly, Lowiaceae originated in the tropics during the Late Cretaceous, eventually becoming extinct in other regions and was only found in Asia since middle Miocene (Kress and Specht, 2006). Thus, the formation of this distribution type largely involved the migration routes of the Paleogene and Neogene (Figure 4B), and the second formation model (Figure 4C).

Type X characterized by tropical Australasia distribution and comprising 17 families, it was inferred to be a relatively ancient distribution type in origin time (ranking thirteenth in terms of the median stem age; Figure S9; Table S3) and formed mainly from the Late Cretaceous to the Neogene (Figure 3A; Table S6). Among the five families in this distribution type, three originated in Australia (Figure S9; Table S5). In addition to *in situ* origins, this distribution type also involved the LDD (Table S4), such as Argophyllaceae (Maurin and Smissen, 2022) and Campynemataceae (Givnish et al., 2016b), and its formation was related to the first formation model (Figure 4C). The land connection between New Caledonia and New Zealand in the mid-Tertiary was inferred to contribute to the plant exchange of Corynocarpaceae between these two regions (Wagstaff and Dawson, 2000). Additionally, as for Type XI characterized by tropical Africa distribution and comprising 18 families, it was inferred to be a relatively young distribution type in origin time (ranking fifth in terms of the median stem age; Figure S9; Table S3) and formed mainly during the Miocene (Figure 3A; Table S6). The formation of this distribution type is correlated with the formation and evolution of the Sahara Desert or aridification of Africa (Wu et al., 2006; Zhang et al., 2014). Based on the endemic distribution of families and the relevant geological history and ecological environment, three subtypes were further proposed for the distribution types of the Old World tropics: the subtypes X.I (endemic to New Caledonia) and X.II (endemic to Fiji) in Type X, and the subtype XI.I (endemic to Madagascar) in Type XI. Only the subtypes X.II, inferred based on Degeneriaceae, formed during the Pliocene (Figure 3A; Table S6). Considering the formation history of the islands where these three subtypes are located (Scotese, 2001; Smith et al., 2004), if the origin time of the families within these subtypes predated the emergence the islands, it is highly likely that these families dispersed/migrated to these islands from surrounding land areas via the LDD and went extinct in their origin place.

Type IV characterized by the Old World tropics distribution and comprising 7 families was inferred to be a relatively young distribution type in origin time (ranking fourth in terms of the median stem age; Figure S9; Table S3) and formed mainly in the Miocene (Figure 3A; Table S6); Type V with tropical Asia to tropical Australasia distribution and 14 families was inferred as a relatively ancient distribution type in origin time (ranking tenth in terms of the median stem age; Figure S9; Table S3) and formed mainly in the Miocene; Type VI characterized by tropical Asia to tropical Africa distribution and comprising 16 families was inferred as a relatively ancient distribution type in origin time (ranking fourteenth in terms of the median stem age; Figure S9; Table S3) and formed mainly during the Late Cretaceous, the Eocene, and the Pliocene. Two families of Type IV originated in Australia and the tropics, respectively; one family of Type V originated in Africa; and one family of Type VI originated in Southern Hemisphere regions of South America and Africa. Specifically, crown group Dipterocarpaceae of Type VI was inferred to originate in tropical Africa during the mid-Cretaceous, subsequently undergoing dispersal to the India, with its main distribution being primarily shaped by the combined effects of the India-Asia continental collision and wind-mediated LDD (Bansal et al., 2022). Meanwhile, Musaceae (Janssens et al., 2016) of Type IV and Nepenthaceae (Biswal et al., 2018) of Type V were inferred to originate respectively from the tropics and Africa, but their subsequent evolution was related to the drop of sea level and the emergence of land between Asia and Oceania. The dispersal of Musaceae from northern Indo-Burma to Africa during the Miocene could be the result of a gradual overland dispersal via an Arabian corridor or a LDD event (Janssens et al., 2016), which was also the significant factors in the palaeotropical intercontinental disjunct formation of Annonaceae corresponding to its post-boreotropical processes (Thomas et al., 2015). Furthermore, the earliest fossils of Pandanaceae (Snead, 1969) and Aponogetonaceae (Grimsson et al., 2014) of Type IV, and Zingiberaceae (Hickey and Peterson, 1978) of Type V were found in Canada during the Paleogene, the United States during the Late Cretaceous, and the United States during the Late Cretaceous, respectively, suggesting that these families were distributed in high-latitude regions of the Northern Hemisphere in the early stages of their evolution and then migrated southward to form their current distribution due to factors such as climate cooling. Additionally, the aquatic Aponogetonaceae (Chen et al., 2015), and Musaceae (Janssens et al., 2016) as well as Pittosporaceae (Chandler et al., 2007) of the Type IV were also indicated to have adapted to the LDD to a certain extent.

**Distribution supertype 2 of tropics involving New World**

Type IX characterized by the New World tropics distribution and comprising 35 families was inferred as a relatively young distribution type in origin time (ranking sixth in terms of the median stem age; Figure S9; Table S3). This distribution type originated mainly from Gondwana (Figure S9; Table S5), and formed mainly from the Oligocene to the Miocene (Figure 3A; Table S6). In detail, Bromeliaceae of this distribution type was inferred to originate from the Guiana Shield in the Cretaceous, and then spread and diversify in the New World during the middle Miocene, which was largely influenced by the uplift of the Andes and related climate (Givnish et al., 2011). Long-distance seed dispersal in Bromeliaceae was also inferred (Givnish et al., 2011). Moreover, South America and Panama began to collide between 25 and 23 Ma (Farris et al., 2011), and then the Panama seaway was closed (Montes et al., 2015) forming the Panama land bridge, which also provided opportunities for the exchange of families between South and North America. This distribution type always involved the origination *in situ* (Table S5), and its formation was associated primarily with tectonic movements of the New World tropics and the first formation model (Figure 4C; Table S4).

With respect to Type III characterized by tropical Asia and tropical America disjunct distribution and comprising 13 families, it inferred as a relatively young distribution type in origin time (ranking third in terms of the median stem age; Figure S9; Table S3). This distribution type originated mainly from Laurasia (Figure S9; Table S5), and formed mainly from the Oligocene to Miocene (Figure 3A; Table S6). Sabiaceae, as an example, was inferred to originate in Eurasia of the Early Cretaceous, and then spread to the Northern Hemisphere since the Paleogene via land bridge, and subsequently migrate southward as the global temperature dropped in the Miocene to form the current disjunct distribution (Yang et al., 2018). Regional climate also exerted a stronger effect on the formation of this distribution type; for example the formation and strengthening of the East Asian monsoon were inferred to be linked to the evolution of Theaceae (Yu et al., 2017). Additionally, the fruits of Sabiaceae (Yang et al., 2018) were also inferred to likely adapt to bird-mediated LDD. Overall, the formation of this distribution type largely involved climate, land bridges and tectonic movement (Table S4), and the migration routes of Paleogene and Neogene (Figure 4B), and second or third formation model (Figure 4C). Compared to the origin and evolution of Type XIII with its similar disjunct distribution, Type III had a younger origin time and showed a stronger adaptation to the LDD. Nevertheless, the formation of these two distribution types both largely involved the NALBs or BLB as migration corridors and climate factors.

As for Type VII characterized by South Atlantic disjunct distribution and comprising 11 families, it was inferred to be a relatively young distribution type in origin time (ranking ninth in terms of the median stem age; Figure S9; Table S3), and formed during the Eocene (Figure 3A; Table S6). Two families originated from Gondwana, the third from the tropics, and the other one from North America in this distribution type (Figure S10; Table S5). Based on the relatively ancient formation time of this distribution type of some families, it was thus hypothesized that its South Atlantic disjunct distribution was associated with the splitting of West Gondwana (Sanmartin and Ronquist, 2004). Additionally, the LDD mechanisms were also inferred for some families of this distribution type, such as Canellaceae that was likely associated with bird-mediated dispersal (Müller et al., 2015). Overall, the formation of this distribution type largely involved climate, the LDD and tectonic movement (Table S4), and the migration routes of the Paleogene (Figure 4B) and first formation model (Figure 4C).

Type II characterized by a pantropic distribution and comprising 99 families, was inferred to be a relatively ancient distribution type in origin time (ranking eleventh in terms of the median stem age; Figure S9; Table S3). This distribution type originated always from Gondwana (Figure S9; Table S5), and formed mainly from the Paleocene to Miocene, especially between the Eocene and Miocene (Figure 3A; Table S6). This distribution type is considered to involve two migration routes: one route is from the New World tropics into the Old World tropics via the NALBs; the other route is a route in the opposite direction (Donoghue, 2008) (Figure 4B). The tectonic movements and climate, especially between the Eocene and Miocene, were of great significance to the formation of this distribution type. For example, these migration routes generally expanded towards high latitudes in the early Tertiary warm period, then withdrew to low latitudes as the climate cooled, eventually forming the current pantropical disjunct distribution. Coincidentally, the rapid diversification of Loranthaceae in this distribution type was also detected to be associated with warm climate periods (Liu et al., 2018). The NALBs were inferred to have played a bridging role in the dispersal of families such as Burmanniaceae (Merckx et al., 2008), Malpighiaceae (Davis et al., 2002; Donoghue, 2008) and Melastomataceae (Renner et al., 2001) between both sides of these land bridges, and the BLB and Antarctica with a similar effect on Simaroubaceae (Clayton et al., 2009) and Cunoniaceae (Pillon et al., 2021), respectively (Figure 4B). Furthermore, 76.77% of families of this distribution type have animal or wind or water-mediated dispersal, with 20 families having all three types of dispersal in Type II (Table S3). The LDD mechanism was inferred to exert significant influence on the formation of this distribution type (LDD-mentioned: 70.37% of families; Table S4). Specifically, the berries of Loranthaceae (Liu et al., 2018), Myrtaceae (Thornhill et al., 2015), and Smilacaceae (Qi et al., 2023) were inferred to adapt to bird-mediated LDD, and some fruits of Cucurbitaceae (Schaefer et al., 2009) and Simaroubaceae (Clayton et al., 2009) were inferred to adapt to both bird- and ocean current-mediated LDD. Additionally, the LDD has also been detected in other families of this distribution type, such as Cunoniaceae (Pillon et al., 2021), Chrysobalanaceae (Bardon et al., 2016), and Urticaceae (Huang et al., 2019; Wu et al., 2018). Based on the disjunct distribution of the family, as well as the geological history and ecological environment of the corresponding regions, subtypes II.I (tropical Asia-Australasia and tropical America) and II.II (tropical Asia-tropical Africa-tropical America) were further proposed within Type II. Subtype II.I mainly formed during the Paleogene, and subtype II.II mainly formed during the Oligocene (Figure 3A; Table S6). Only one family, Corsiaceae, in subtype II.I originated in Gondwana, and the subsequent evolution was potentially related to the fragmentation of the Gondwanan plates (Mennes et al., 2015) (Figure 4B). Meanwhile, Thorne (1972) emphasized that the formation of the circum-Pacific tropical disjunct distribution similar to this subtype appeared to be affected by the LDD. As for subtype II.II, its formation was influenced by several factors, but mainly by the tectonic movement and the LDD (Schneider et al., 2022; Zhang et al., 2007). Specifically, the formation of Anisophylleaceae was indicated to be related to the disintegration of Gondwana and water-mediated LDD (Zhang et al., 2007). On the whole, the formation of the Type II largely involved the migration routes of the Paleogene and Neogene (Figure 4B), tectonic movement and the LDD (Table S4), and the second and third formation models (Figure 4C).

**
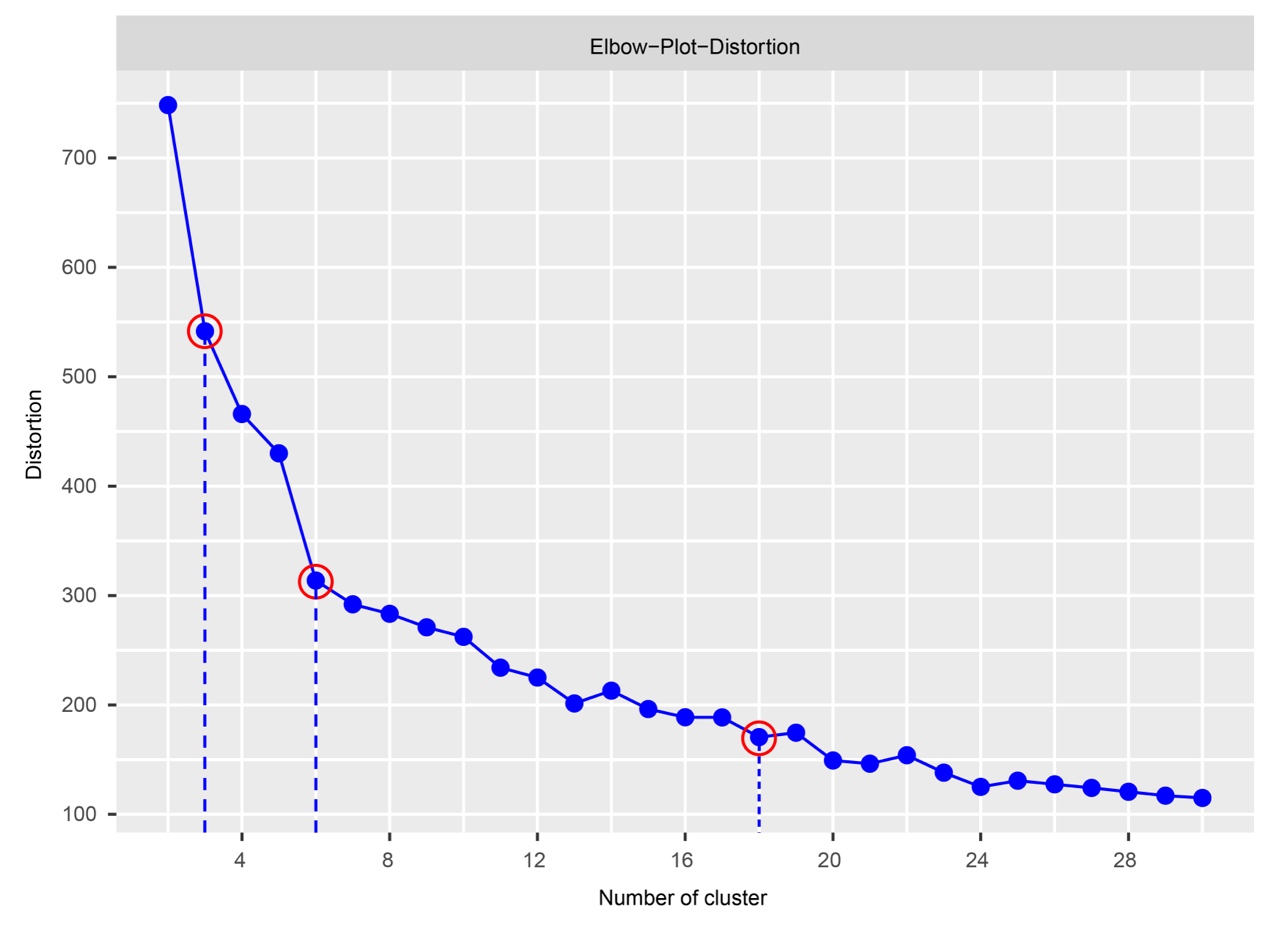
**

**Figure S1. Optimized numbers of clusters *K* for the *K*-means clustering**

The optimized numbers of clusters K for the K-means clustering were indicated by red circles using the elbow method considering distortion in the distribution of global spermatophyte families, which were three, six, and 18 from left to right, respectively.

**
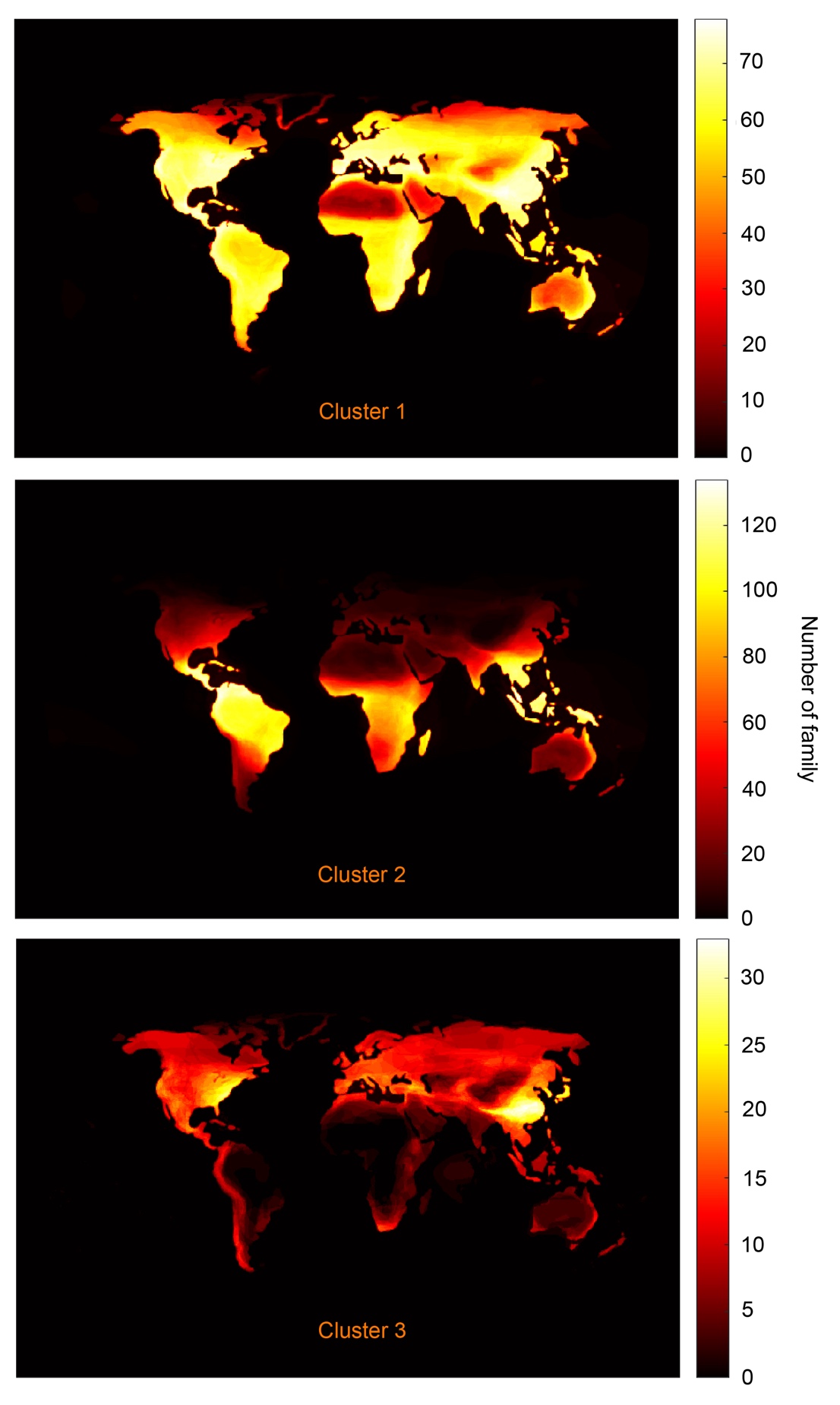
**

**Figure S2A. Distribution maps of three clusters for global spermatophyte families when *K* = 3 in *K*-means clustering algorithm**

**
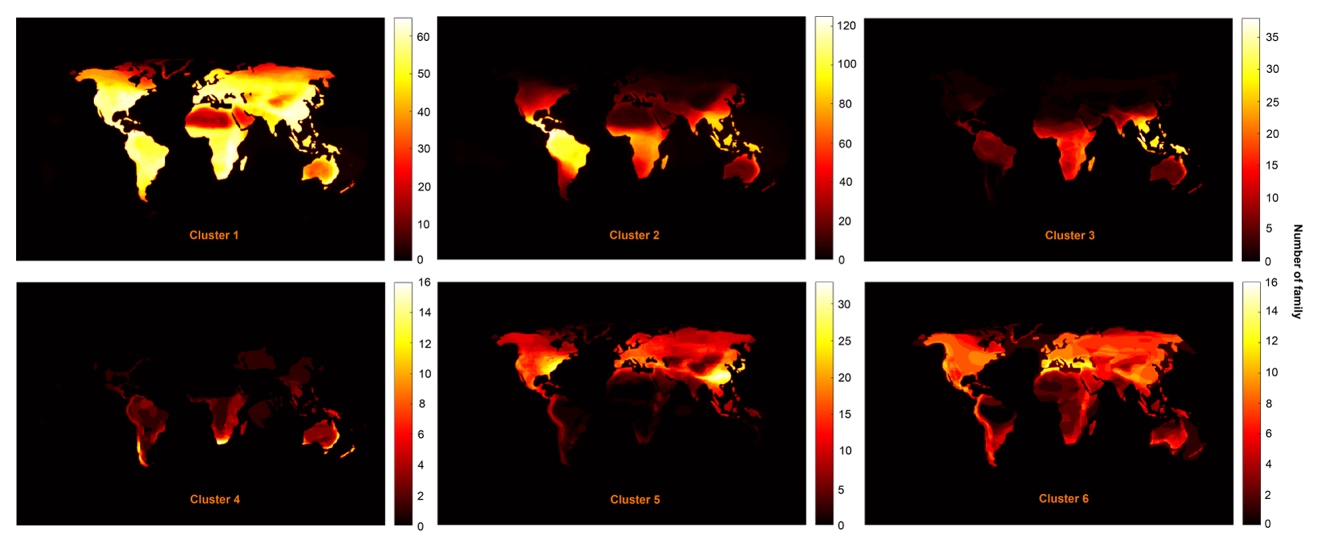
Figure S2B. Distribution maps of six clusters for global spermatophyte families when *K* = 6 in *K*-means clustering algorithm**

**
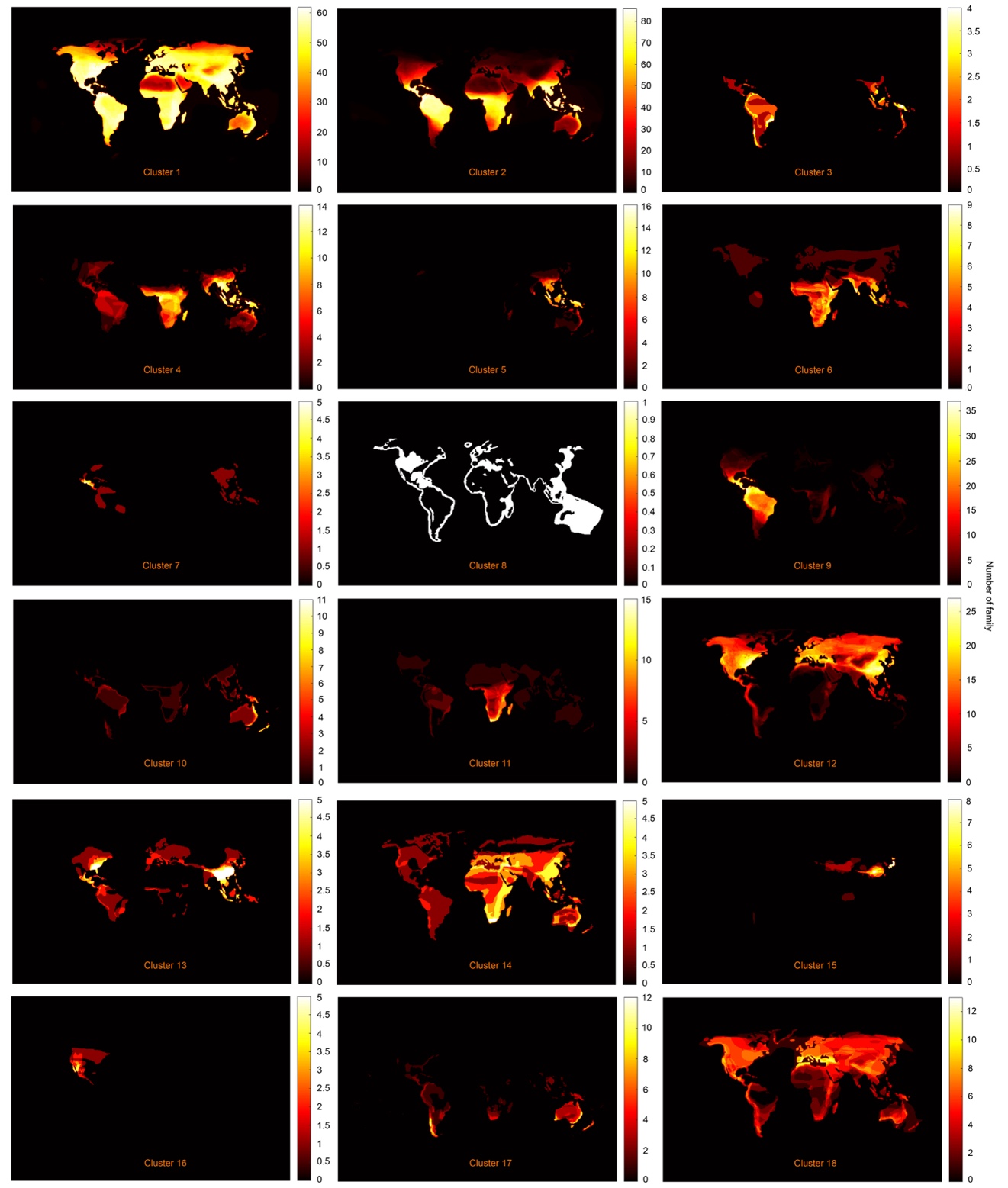
Figure S2C. Distribution maps of 18 clusters for global spermatophyte families when *K* = 18 in *K*-means clustering algorithm**

**
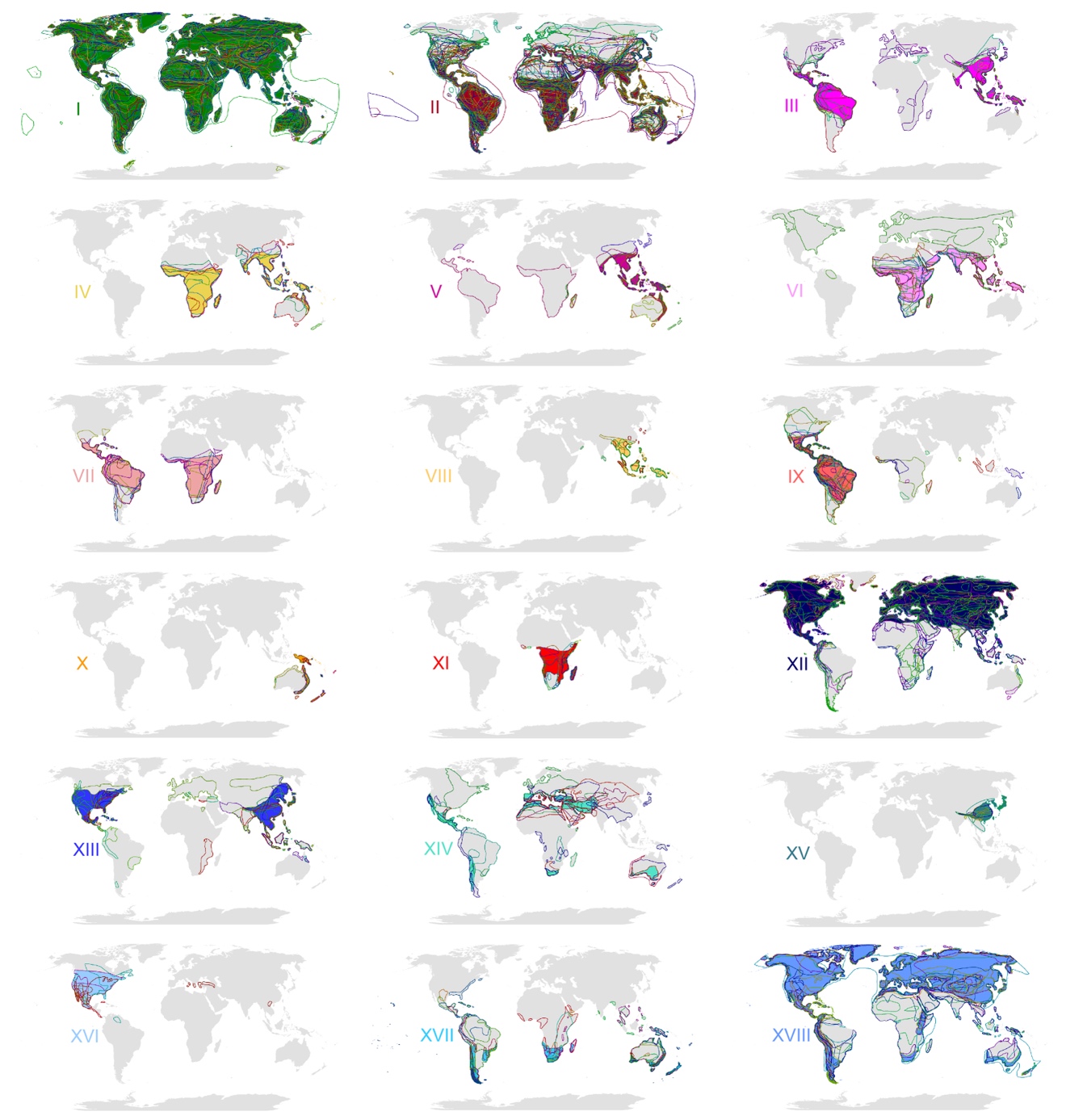
Figure S3.** **Pattern diagrams and their base maps of 18 distribution types**

Different colored and closed lines represent the distribution range of different families, and colored area, except for the gray world map, is the pattern diagrams of a distribution type in each image. 18 distribution types were indicated by roman numerals.

**
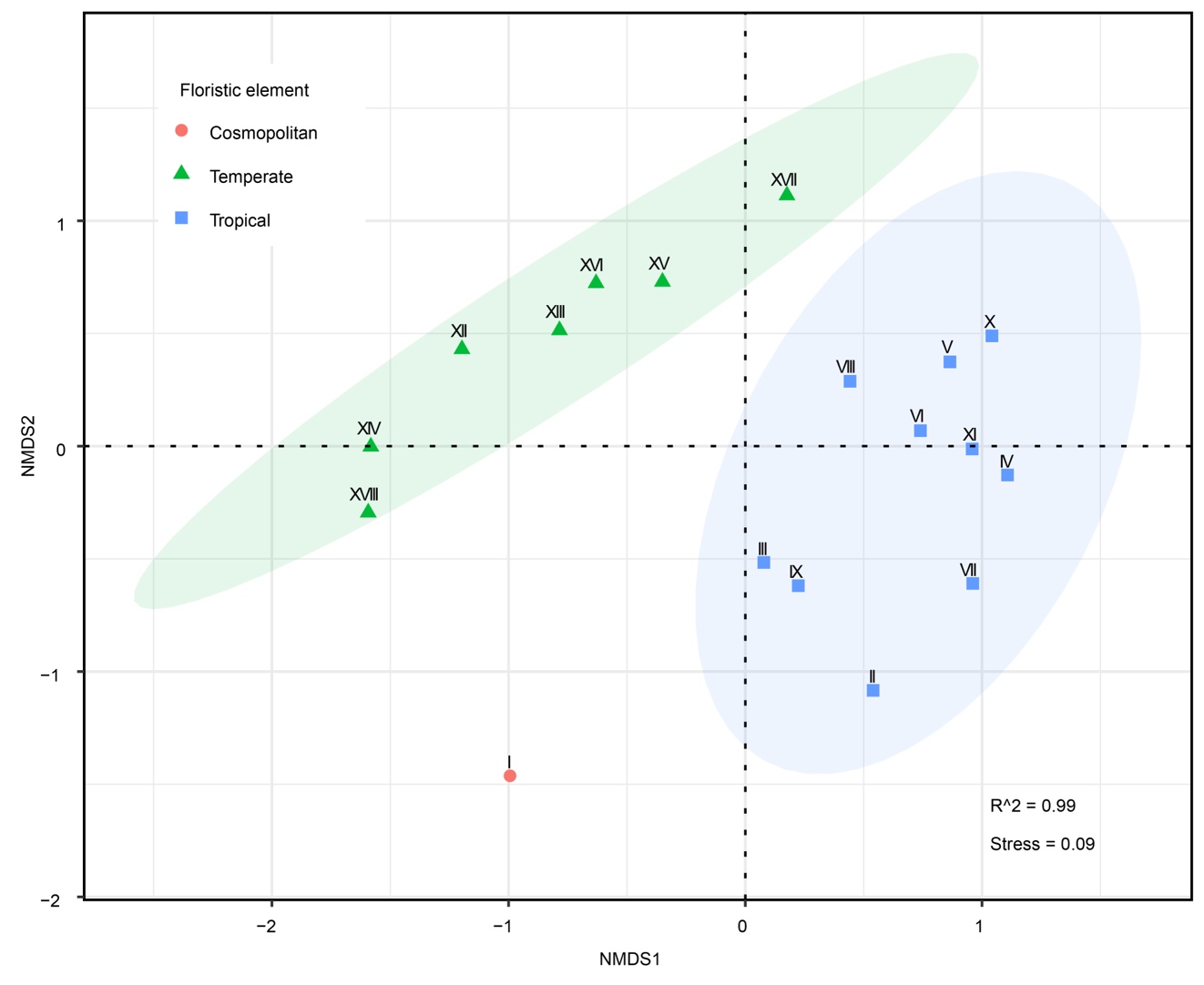
Figure S4.** **Ordination of non‐metric multidimensional scaling (NMDS) of 18 distribution types for global spermatophyte families based on Euclidean distances**

**
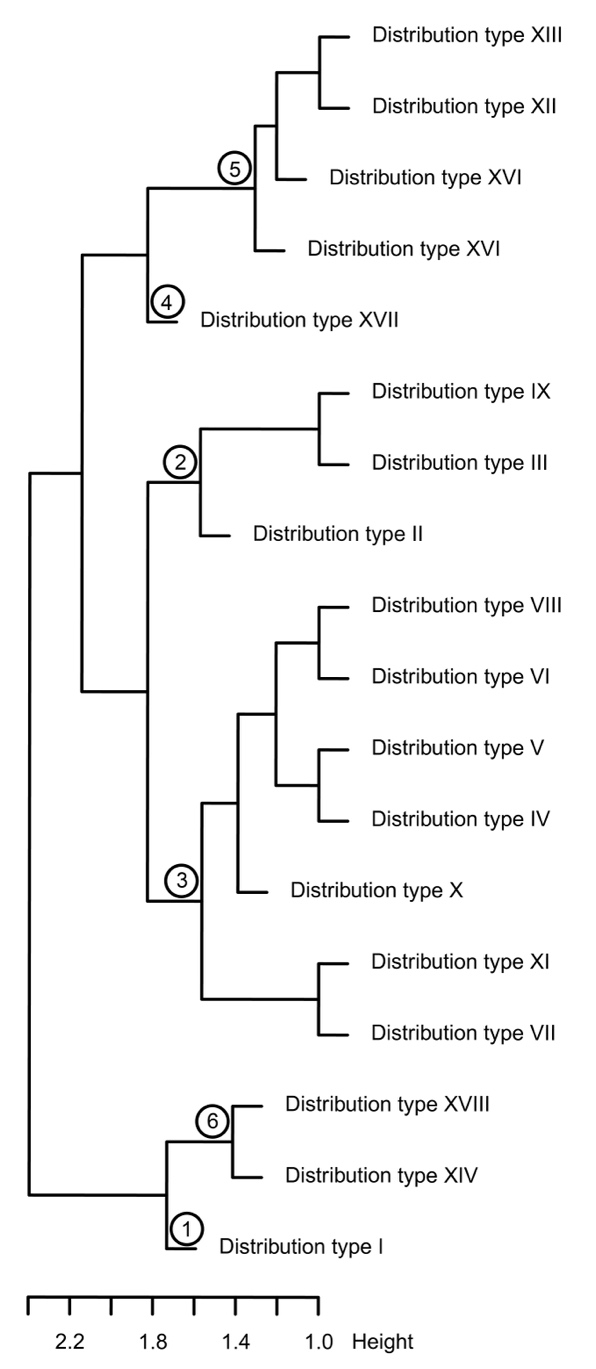
**

**Figure S5****. Dendrogram of 18 distribution types for global spermatophyte families based on weighted pair‐group method using arithmetic averages (WPGMA)**

The arabic numerals with circles on the nodes of the dendrogram represent the number of corresponding clades in this paper.

**
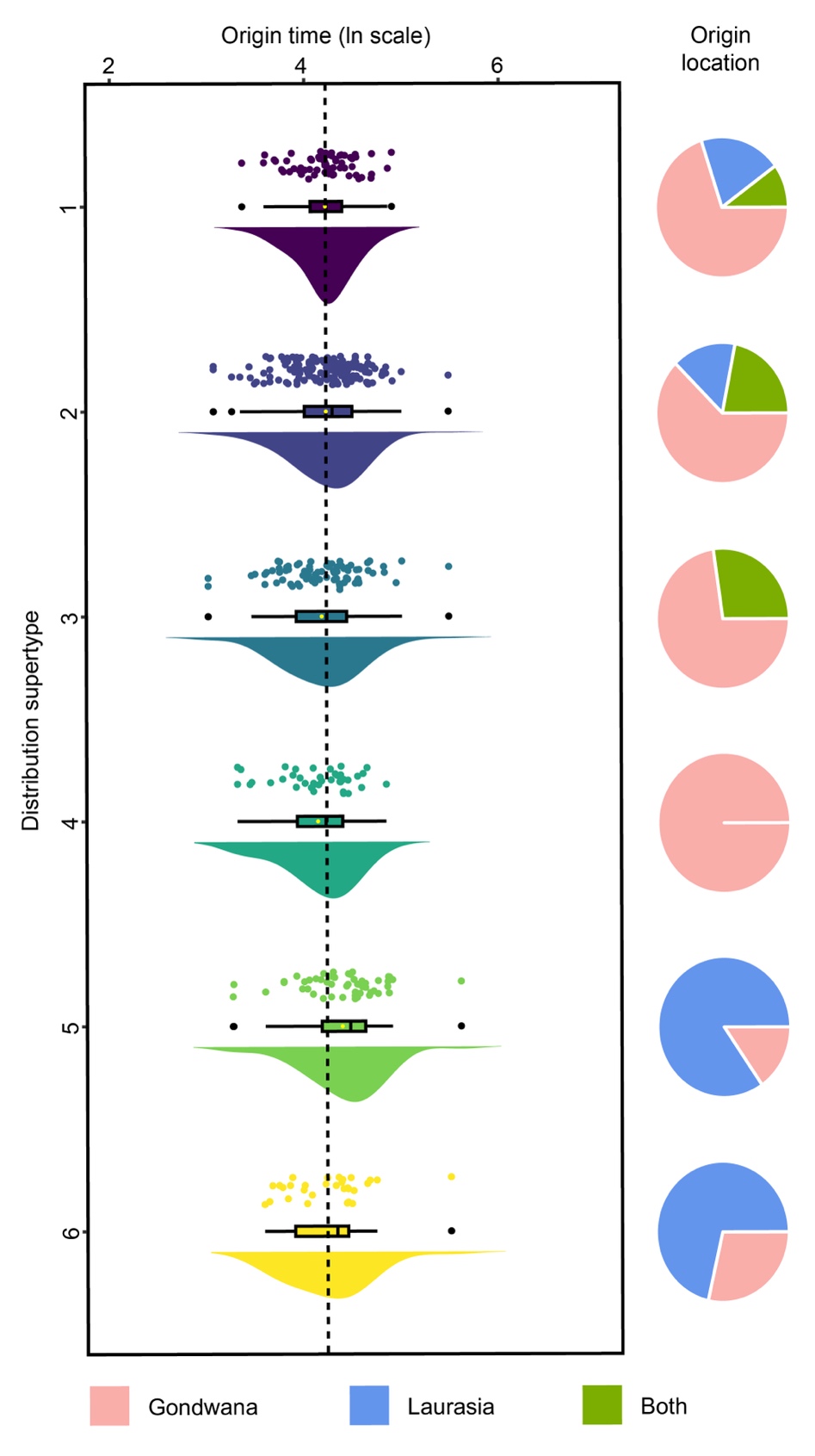
**

**Figure S6. Time and location of origin of six distribution supertypes**

Percentage of origin location for the six distribution supertypes based on origin locations of 96 families of spermatophytes (the right portion), and origin time for the six distribution supertypes based on ln origin time for 429 families of spermatophytes, except for Cephalotaxaceae and Tiganophytaceae (the left portion).


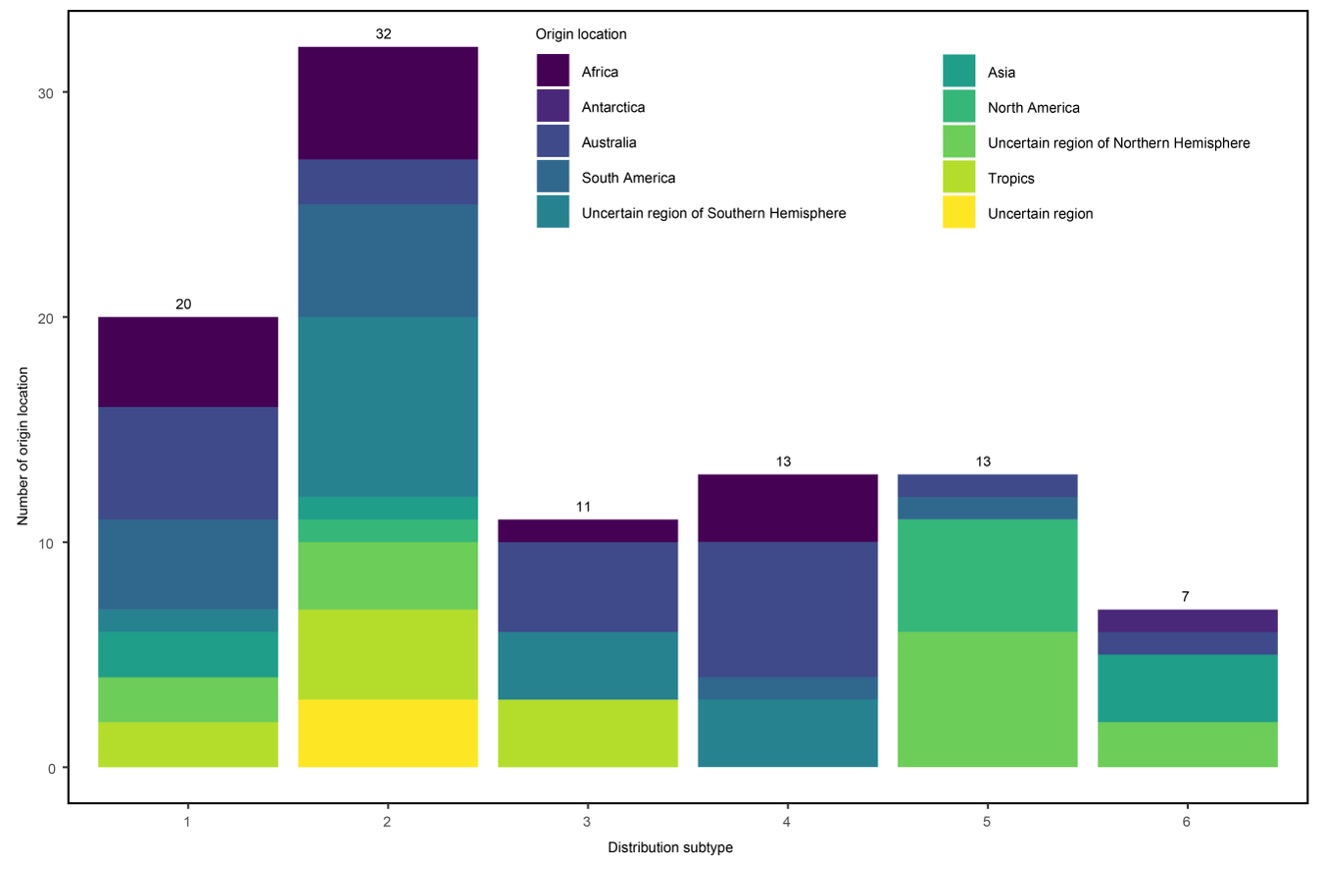
**Figure S7.** **Detailed origin locations of six distribution supertypes based on origin locations of 96 families of spermatophytes**


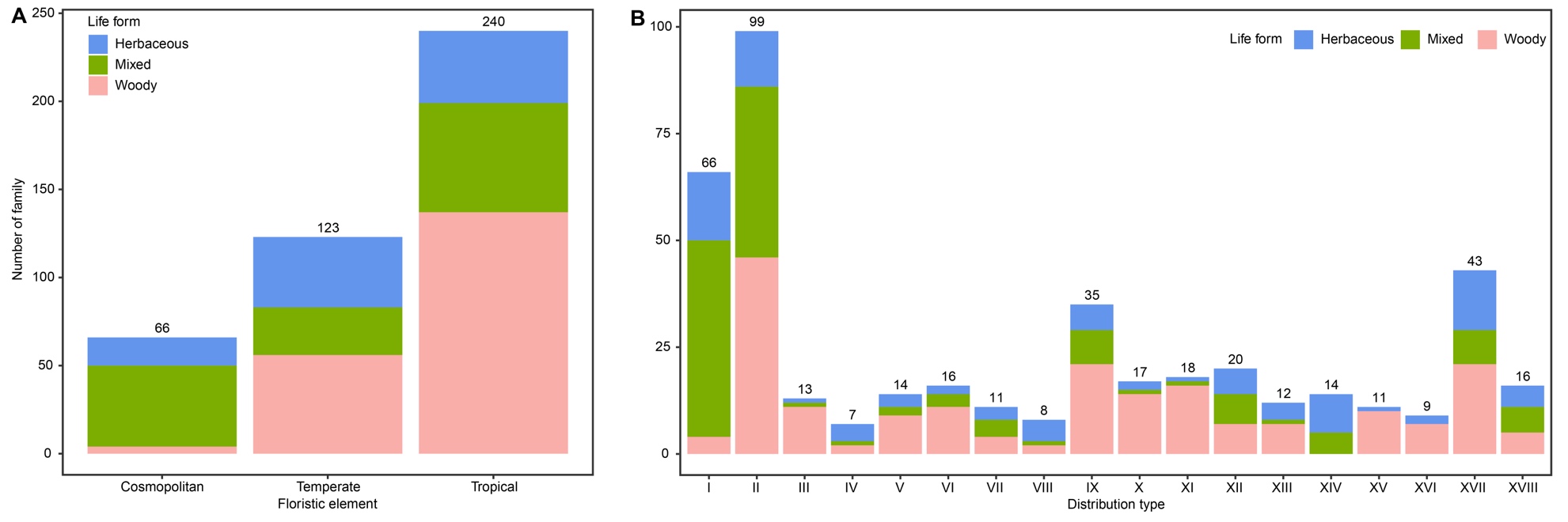
**Figure S8. Life form of 3 floristic elements and 18 distribution types of global spermatophyte families**

Life form of 3 floristic elements (A) and 18 distribution types (B) of global spermatophyte families.


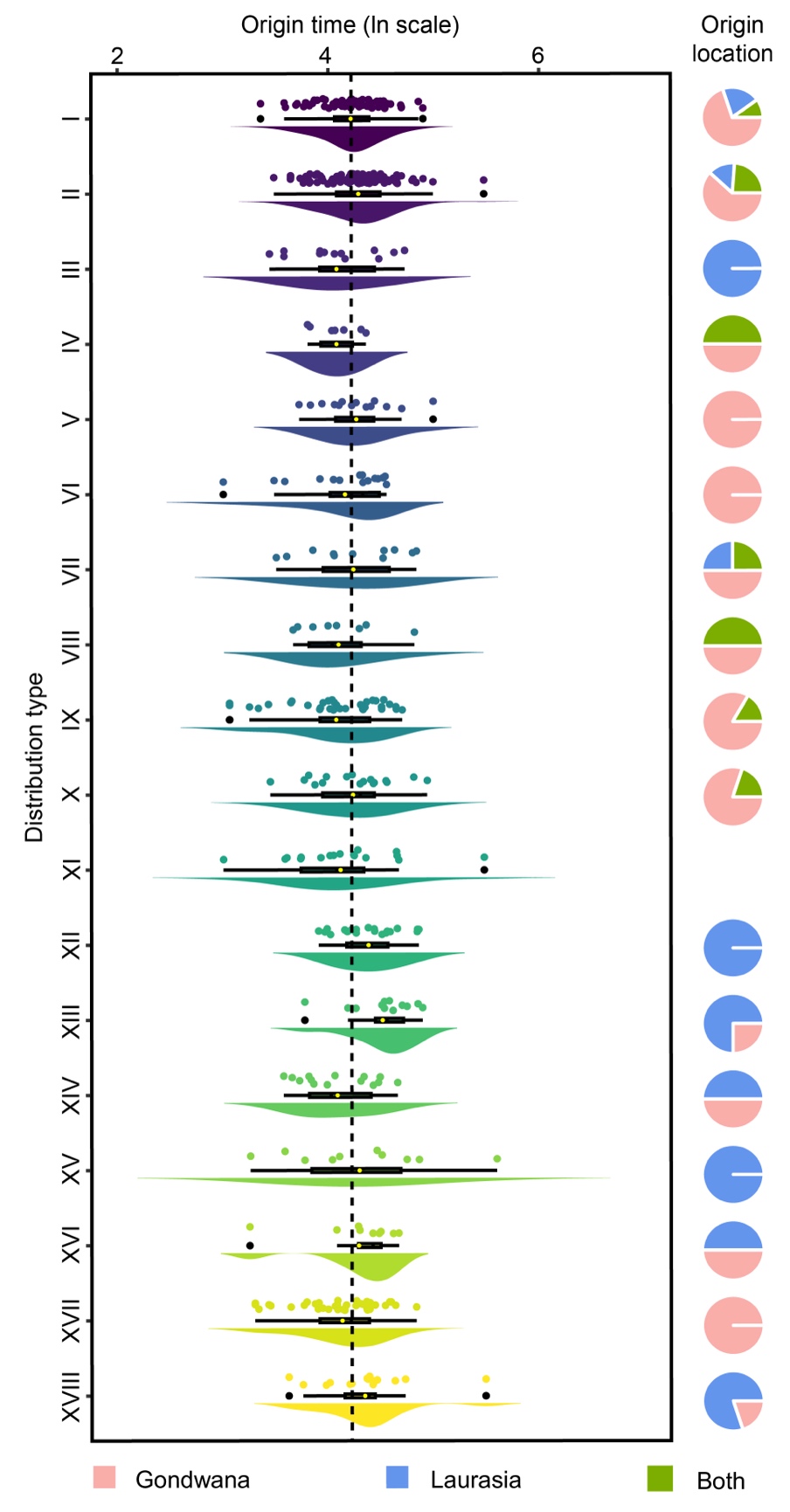


**Figure S9. Time and location of origin of 18 distribution types**

Percentage of origin location for the 18 distribution types based on origin locations of 96 families of spermatophytes (the right portion), and origin time for the 18 distribution types based on ln origin time for 429 families of spermatophytes, except for Cephalotaxaceae and Tiganophytaceae (the left portion).


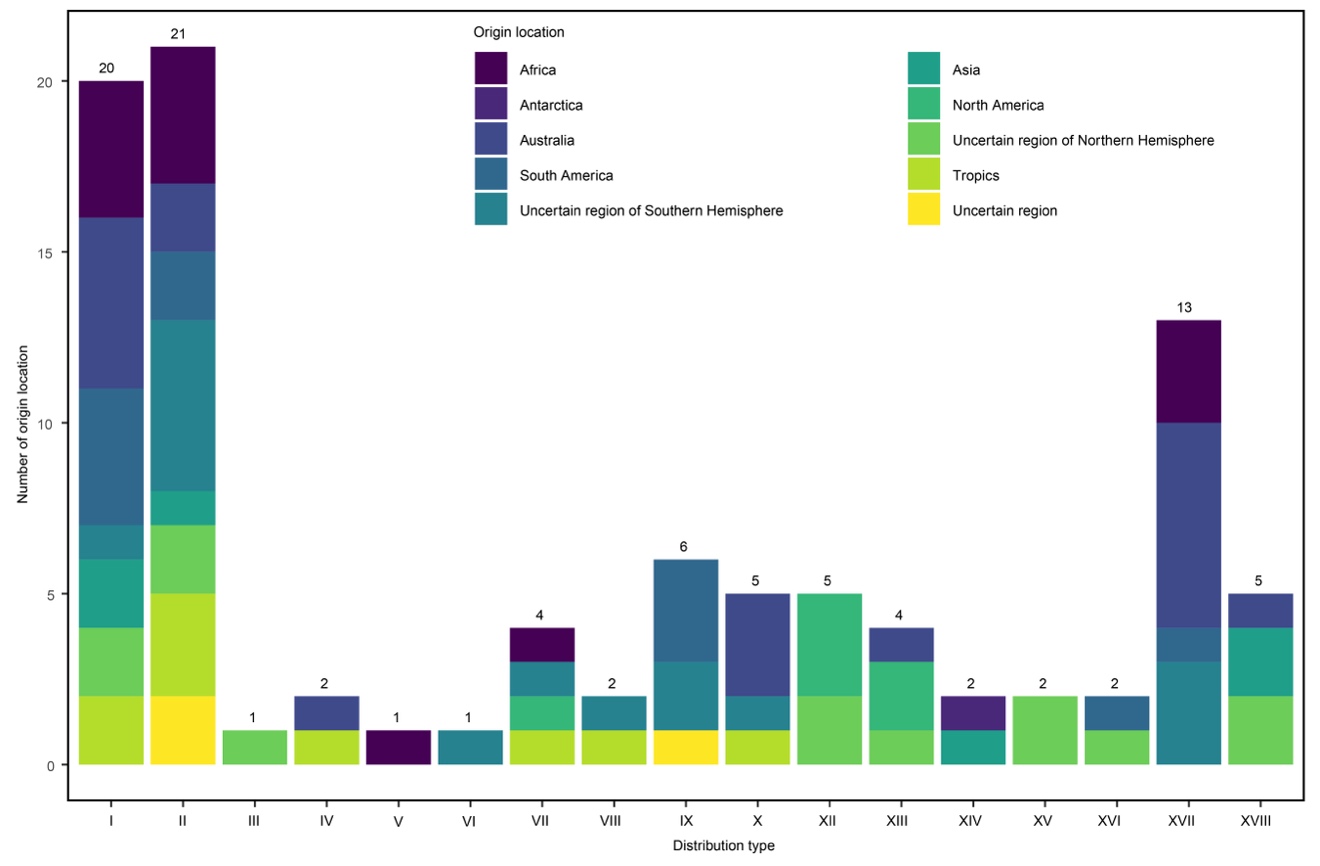
**Figure S10. Detailed origin locations of 18 distribution types based on origin locations of 96 families of spermatophytes**

**Table S1.** **Detailed information of 3, 6, and 18 clusters for global spermatophyte families based on K-means clustering algorithm**

Each family is also listed with the corresponding floristic element, distribution supertype and distribution type based on anthropogenic verification.

| **Number** | **Family** | **3 clusters based on K-means analysis** | **Floristic elements based on anthropogenic verification** | **6 clusters based on K-means analysis** | **Distribution supertypes based on anthropogenic verification** | **18 clusters based on K-means analysis** | **Distribution types based on anthropogenic verification** |
| --- | --- | --- | --- | --- | --- | --- | --- |
| 1 | Acanthaceae | cluster 3 | tropical element | cluster 2 | distribution supertype of tropics involving New World | cluster 2 | distribution type II |
| 2 | Achariaceae | cluster 3 | tropical element | cluster 2 | distribution supertype of tropics involving New World | cluster 2 | distribution type II |
| 3 | Achatocarpaceae | cluster 3 | tropical element | cluster 2 | distribution supertype of tropics involving New World | cluster 9 | distribution type IX |
| 4 | Acoraceae | cluster 2 | temperate element | cluster 5 | distribution supertype of north temperate region | cluster 12 | distribution type XIII |
| 5 | Actinidiaceae | cluster 3 | tropical element | cluster 2 | distribution supertype of tropics involving New World | cluster 2 | distribution type III |
| 6 | Adoxaceae | cluster 1 | temperate element | cluster 6 | distribution supertype of north temperate region | cluster 12 | distribution type XII |
| 7 | Aextoxicaceae | cluster 2 | temperate element | cluster 4 | distribution supertype of south temperate region | cluster 17 | distribution type XVII |
| 8 | Aizoaceae | cluster 3 | tropical element | cluster 3 | distribution supertype of tropics involving New World | cluster 11 | distribution type II |
| 9 | Akaniaceae | cluster 3 | tropical element | cluster 3 | distribution supertype of Old World tropics | cluster 5 | distribution type V |
| 10 | Alismataceae | cluster 1 | cosmopolitan element | cluster 1 | cosmopolitan supertype | cluster 1 | distribution type I |
| 11 | Alseuosmiaceae | cluster 3 | tropical element | cluster 4 | distribution supertype of Old World tropics | cluster 10 | distribution type X |
| 12 | Alstroemeriaceae | cluster 3 | temperate element | cluster 2 | distribution supertype of south temperate region | cluster 9 | distribution type XVII |
| 13 | Altingiaceae | cluster 2 | temperate element | cluster 5 | distribution supertype of north temperate region | cluster 13 | distribution type XIII |
| 14 | Alzateaceae | cluster 3 | tropical element | cluster 2 | distribution supertype of tropics involving New World | cluster 9 | distribution type IX |
| 15 | Amaranthaceae | cluster 1 | cosmopolitan element | cluster 1 | cosmopolitan supertype | cluster 1 | distribution type I |
| 16 | Amaryllidaceae | cluster 1 | cosmopolitan element | cluster 1 | cosmopolitan supertype | cluster 1 | distribution type I |
| 17 | Amborellaceae | cluster 3 | tropical element | cluster 3 | distribution supertype of Old World tropics | cluster 10 | distribution type X |
| 18 | Anacampserotaceae | cluster 2 | temperate element | cluster 4 | distribution supertype of south temperate region | cluster 17 | distribution type XVII |
| 19 | Anacardiaceae | cluster 3 | tropical element | cluster 2 | distribution supertype of tropics involving New World | cluster 2 | distribution type II |
| 20 | Ancistrocladaceae | cluster 3 | tropical element | cluster 3 | distribution supertype of Old World tropics | cluster 6 | distribution type VI |
| 21 | Anisophylleaceae | cluster 3 | tropical element | cluster 2 | distribution supertype of tropics involving New World | cluster 6 | distribution type II |
| 22 | Annonaceae | cluster 3 | tropical element | cluster 2 | distribution supertype of tropics involving New World | cluster 2 | distribution type II |
| 23 | Aphanopetalaceae | cluster 3 | tropical element | cluster 3 | distribution supertype of Old World tropics | cluster 10 | distribution type X |
| 24 | Aphloiaceae | cluster 3 | tropical element | cluster 3 | distribution supertype of Old World tropics | cluster 11 | distribution type XI |
| 25 | Apiaceae | cluster 1 | cosmopolitan element | cluster 1 | cosmopolitan supertype | cluster 1 | distribution type I |
| 26 | Apocynaceae | cluster 3 | tropical element | cluster 2 | distribution supertype of tropics involving New World | cluster 2 | distribution type II |
| 27 | Apodanthaceae | cluster 1 | temperate element | cluster 1 | distribution supertype of north and south temperate regions | cluster 1 | distribution type XIV |
| 28 | Aponogetonaceae | cluster 3 | tropical element | cluster 3 | distribution supertype of Old World tropics | cluster 4 | distribution type IV |
| 29 | Aquifoliaceae | cluster 3 | tropical element | cluster 2 | distribution supertype of tropics involving New World | cluster 9 | distribution type III |
| 30 | Araceae | cluster 1 | cosmopolitan element | cluster 1 | cosmopolitan supertype | cluster 1 | distribution type I |
| 31 | Araliaceae | cluster 3 | tropical element | cluster 2 | distribution supertype of tropics involving New World | cluster 2 | distribution type II |
| 32 | Araucariaceae | cluster 3 | tropical element | cluster 2 | distribution supertype of tropics involving New World | cluster 3 | distribution type II |
| 33 | Arecaceae | cluster 3 | tropical element | cluster 2 | distribution supertype of tropics involving New World | cluster 2 | distribution type II |
| 34 | Argophyllaceae | cluster 3 | tropical element | cluster 4 | distribution supertype of Old World tropics | cluster 10 | distribution type X |
| 35 | Aristolochiaceae | cluster 1 | tropical element | cluster 2 | distribution supertype of tropics involving New World | cluster 2 | distribution type II |
| 36 | Asparagaceae | cluster 1 | cosmopolitan element | cluster 1 | cosmopolitan supertype | cluster 1 | distribution type I |
| 37 | Asphodelaceae | cluster 1 | cosmopolitan element | cluster 1 | cosmopolitan supertype | cluster 14 | distribution type I |
| 38 | Asteliaceae | cluster 2 | temperate element | cluster 4 | distribution supertype of south temperate region | cluster 17 | distribution type XVII |
| 39 | Asteraceae | cluster 1 | cosmopolitan element | cluster 1 | cosmopolitan supertype | cluster 1 | distribution type I |
| 40 | Asteropeiaceae | cluster 3 | tropical element | cluster 3 | distribution supertype of Old World tropics | cluster 11 | distribution type XI |
| 41 | Atherospermataceae | cluster 2 | temperate element | cluster 4 | distribution supertype of south temperate region | cluster 17 | distribution type XVII |
| 42 | Austrobaileyaceae | cluster 3 | tropical element | cluster 3 | distribution supertype of Old World tropics | cluster 10 | distribution type X |
| 43 | Balanopaceae | cluster 3 | tropical element | cluster 3 | distribution supertype of Old World tropics | cluster 10 | distribution type X |
| 44 | Balanophoraceae | cluster 3 | tropical element | cluster 2 | distribution supertype of tropics involving New World | cluster 2 | distribution type II |
| 45 | Balsaminaceae | cluster 3 | tropical element | cluster 3 | distribution supertype of Old World tropics | cluster 6 | distribution type VI |
| 46 | Barbeuiaceae | cluster 3 | tropical element | cluster 3 | distribution supertype of Old World tropics | cluster 11 | distribution type XI |
| 47 | Barbeyaceae | cluster 3 | tropical element | cluster 3 | distribution supertype of Old World tropics | cluster 6 | distribution type VI |
| 48 | Basellaceae | cluster 3 | tropical element | cluster 2 | distribution supertype of tropics involving New World | cluster 9 | distribution type VII |
| 49 | Bataceae | cluster 3 | tropical element | cluster 2 | distribution supertype of tropics involving New World | cluster 9 | distribution type II |
| 50 | Begoniaceae | cluster 3 | tropical element | cluster 2 | distribution supertype of tropics involving New World | cluster 2 | distribution type II |
| 51 | Berberidaceae | cluster 2 | temperate element | cluster 5 | distribution supertype of north and south temperate regions | cluster 12 | distribution type XVIII |
| 52 | Berberidopsidaceae | cluster 2 | temperate element | cluster 4 | distribution supertype of south temperate region | cluster 17 | distribution type XVII |
| 53 | Betulaceae | cluster 2 | temperate element | cluster 5 | distribution supertype of north and south temperate regions | cluster 12 | distribution type XVIII |
| 54 | Biebersteiniaceae | cluster 2 | temperate element | cluster 5 | distribution supertype of north and south temperate regions | cluster 15 | distribution type XIV |
| 55 | Bignoniaceae | cluster 3 | tropical element | cluster 2 | distribution supertype of tropics involving New World | cluster 2 | distribution type II |
| 56 | Bixaceae | cluster 3 | tropical element | cluster 2 | distribution supertype of tropics involving New World | cluster 2 | distribution type II |
| 57 | Blandfordiaceae | cluster 2 | temperate element | cluster 4 | distribution supertype of south temperate region | cluster 17 | distribution type XVII |
| 58 | Bonnetiaceae | cluster 3 | tropical element | cluster 3 | distribution supertype of tropics involving New World | cluster 3 | distribution type III |
| 59 | Boraginaceae | cluster 1 | cosmopolitan element | cluster 1 | cosmopolitan supertype | cluster 1 | distribution type I |
| 60 | Boryaceae | cluster 2 | temperate element | cluster 4 | distribution supertype of south temperate region | cluster 17 | distribution type XVII |
| 61 | Brassicaceae | cluster 1 | cosmopolitan element | cluster 1 | cosmopolitan supertype | cluster 1 | distribution type I |
| 62 | Bromeliaceae | cluster 3 | tropical element | cluster 2 | distribution supertype of tropics involving New World | cluster 9 | distribution type IX |
| 63 | Brunelliaceae | cluster 3 | tropical element | cluster 2 | distribution supertype of tropics involving New World | cluster 9 | distribution type IX |
| 64 | Bruniaceae | cluster 2 | temperate element | cluster 4 | distribution supertype of south temperate region | cluster 11 | distribution type XVII |
| 65 | Burmanniaceae | cluster 3 | tropical element | cluster 2 | distribution supertype of tropics involving New World | cluster 2 | distribution type II |
| 66 | Burseraceae | cluster 3 | tropical element | cluster 2 | distribution supertype of tropics involving New World | cluster 2 | distribution type II |
| 67 | Butomaceae | cluster 2 | temperate element | cluster 5 | distribution supertype of north temperate region | cluster 12 | distribution type XII |
| 68 | Buxaceae | cluster 3 | temperate element | cluster 2 | distribution supertype of north temperate region | cluster 13 | distribution type XII |
| 69 | Byblidaceae | cluster 3 | tropical element | cluster 3 | distribution supertype of Old World tropics | cluster 10 | distribution type X |
| 70 | Cabombaceae | cluster 1 | cosmopolitan element | cluster 1 | cosmopolitan supertype | cluster 1 | distribution type I |
| 71 | Cactaceae | cluster 3 | tropical element | cluster 2 | distribution supertype of tropics involving New World | cluster 9 | distribution type IX |
| 72 | Calophyllaceae | cluster 3 | tropical element | cluster 2 | distribution supertype of tropics involving New World | cluster 2 | distribution type II |
| 73 | Calycanthaceae | cluster 2 | temperate element | cluster 5 | distribution supertype of north temperate region | cluster 12 | distribution type XIII |
| 74 | Calyceraceae | cluster 2 | temperate element | cluster 4 | distribution supertype of south temperate region | cluster 17 | distribution type XVII |
| 75 | Campanulaceae | cluster 1 | cosmopolitan element | cluster 1 | cosmopolitan supertype | cluster 1 | distribution type I |
| 76 | Campynemataceae | cluster 3 | tropical element | cluster 3 | distribution supertype of Old World tropics | cluster 10 | distribution type X |
| 77 | Canellaceae | cluster 3 | tropical element | cluster 2 | distribution supertype of tropics involving New World | cluster 9 | distribution type VII |
| 78 | Cannabaceae | cluster 1 | cosmopolitan element | cluster 1 | cosmopolitan supertype | cluster 1 | distribution type I |
| 79 | Cannaceae | cluster 3 | tropical element | cluster 2 | distribution supertype of tropics involving New World | cluster 9 | distribution type IX |
| 80 | Capparaceae | cluster 3 | tropical element | cluster 2 | distribution supertype of tropics involving New World | cluster 2 | distribution type II |
| 81 | Caprifoliaceae | cluster 1 | temperate element | cluster 6 | distribution supertype of north temperate region | cluster 12 | distribution type XII |
| 82 | Cardiopteridaceae | cluster 3 | tropical element | cluster 2 | distribution supertype of tropics involving New World | cluster 2 | distribution type II |
| 83 | Caricaceae | cluster 3 | tropical element | cluster 2 | distribution supertype of tropics involving New World | cluster 9 | distribution type VII |
| 84 | Carlemanniaceae | cluster 3 | tropical element | cluster 3 | distribution supertype of Old World tropics | cluster 5 | distribution type VIII |
| 85 | Caryocaraceae | cluster 3 | tropical element | cluster 2 | distribution supertype of tropics involving New World | cluster 9 | distribution type IX |
| 86 | Caryophyllaceae | cluster 1 | cosmopolitan element | cluster 1 | cosmopolitan supertype | cluster 1 | distribution type I |
| 87 | Casuarinaceae | cluster 3 | tropical element | cluster 3 | distribution supertype of Old World tropics | cluster 5 | distribution type V |
| 88 | Celastraceae | cluster 1 | cosmopolitan element | cluster 1 | cosmopolitan supertype | cluster 1 | distribution type I |
| 89 | Centroplacaceae | cluster 3 | tropical element | cluster 3 | distribution supertype of Old World tropics | cluster 4 | distribution type VI |
| 90 | Cephalotaceae | cluster 2 | temperate element | cluster 4 | distribution supertype of south temperate region | cluster 17 | distribution type XVII |
| 91 | Cephalotaxaceae | cluster 2 | temperate element | cluster 5 | distribution supertype of north temperate region | cluster 15 | distribution type XV |
| 92 | Ceratophyllaceae | cluster 1 | cosmopolitan element | cluster 1 | cosmopolitan supertype | cluster 1 | distribution type I |
| 93 | Cercidiphyllaceae | cluster 2 | temperate element | cluster 5 | distribution supertype of north temperate region | cluster 15 | distribution type XV |
| 94 | Chloranthaceae | cluster 3 | tropical element | cluster 2 | distribution supertype of tropics involving New World | cluster 2 | distribution type II |
| 95 | Chrysobalanaceae | cluster 3 | tropical element | cluster 2 | distribution supertype of tropics involving New World | cluster 2 | distribution type II |
| 96 | Circaeasteraceae | cluster 2 | temperate element | cluster 5 | distribution supertype of north temperate region | cluster 15 | distribution type XV |
| 97 | Cistaceae | cluster 2 | temperate element | cluster 5 | distribution supertype of north and south temperate regions | cluster 12 | distribution type XIV |
| 98 | Cleomaceae | cluster 3 | tropical element | cluster 2 | distribution supertype of tropics involving New World | cluster 2 | distribution type II |
| 99 | Clethraceae | cluster 3 | tropical element | cluster 2 | distribution supertype of tropics involving New World | cluster 2 | distribution type III |
| 100 | Clusiaceae | cluster 3 | tropical element | cluster 2 | distribution supertype of tropics involving New World | cluster 2 | distribution type II |
| 101 | Colchicaceae | cluster 1 | cosmopolitan element | cluster 1 | cosmopolitan supertype | cluster 14 | distribution type I |
| 102 | Columelliaceae | cluster 2 | temperate element | cluster 4 | distribution supertype of south temperate region | cluster 17 | distribution type XVII |
| 103 | Combretaceae | cluster 3 | tropical element | cluster 2 | distribution supertype of tropics involving New World | cluster 2 | distribution type II |
| 104 | Commelinaceae | cluster 3 | tropical element | cluster 2 | distribution supertype of tropics involving New World | cluster 2 | distribution type II |
| 105 | Connaraceae | cluster 3 | tropical element | cluster 2 | distribution supertype of tropics involving New World | cluster 2 | distribution type II |
| 106 | Convolvulaceae | cluster 1 | cosmopolitan element | cluster 1 | cosmopolitan supertype | cluster 1 | distribution type I |
| 107 | Coriariaceae | cluster 1 | temperate element | cluster 6 | distribution supertype of north and south temperate regions | cluster 18 | distribution type XIV |
| 108 | Cornaceae | cluster 1 | temperate element | cluster 6 | distribution supertype of north temperate region | cluster 18 | distribution type XII |
| 109 | Corsiaceae | cluster 3 | tropical element | cluster 2 | distribution supertype of tropics involving New World | cluster 3 | distribution type II |
| 110 | Corynocarpaceae | cluster 3 | tropical element | cluster 3 | distribution supertype of Old World tropics | cluster 10 | distribution type X |
| 111 | Costaceae | cluster 3 | tropical element | cluster 2 | distribution supertype of tropics involving New World | cluster 2 | distribution type II |
| 112 | Crassulaceae | cluster 1 | cosmopolitan element | cluster 1 | cosmopolitan supertype | cluster 1 | distribution type I |
| 113 | Crossosomataceae | cluster 2 | temperate element | cluster 5 | distribution supertype of north temperate region | cluster 16 | distribution type XVI |
| 114 | Crypteroniaceae | cluster 3 | tropical element | cluster 3 | distribution supertype of Old World tropics | cluster 5 | distribution type VIII |
| 115 | Ctenolophonaceae | cluster 3 | tropical element | cluster 3 | distribution supertype of Old World tropics | cluster 4 | distribution type VI |
| 116 | Cucurbitaceae | cluster 3 | tropical element | cluster 2 | distribution supertype of tropics involving New World | cluster 2 | distribution type II |
| 117 | Cunoniaceae | cluster 3 | tropical element | cluster 2 | distribution supertype of tropics involving New World | cluster 2 | distribution type II |
| 118 | Cupressaceae | cluster 1 | temperate element | cluster 6 | distribution supertype of north and south temperate regions | cluster 18 | distribution type XVIII |
| 119 | Curtisiaceae | cluster 2 | temperate element | cluster 4 | distribution supertype of south temperate region | cluster 11 | distribution type XVII |
| 120 | Cycadaceae | cluster 3 | tropical element | cluster 3 | distribution supertype of Old World tropics | cluster 5 | distribution type V |
| 121 | Cyclanthaceae | cluster 3 | tropical element | cluster 2 | distribution supertype of tropics involving New World | cluster 9 | distribution type IX |
| 122 | Cymodoceaceae | cluster 3 | tropical element | cluster 2 | distribution supertype of tropics involving New World | cluster 2 | distribution type II |
| 123 | Cynomoriaceae | cluster 2 | temperate element | cluster 5 | distribution supertype of north and south temperate regions | cluster 12 | distribution type XIV |
| 124 | Cyperaceae | cluster 1 | cosmopolitan element | cluster 1 | cosmopolitan supertype | cluster 1 | distribution type I |
| 125 | Cyrillaceae | cluster 3 | tropical element | cluster 2 | distribution supertype of tropics involving New World | cluster 9 | distribution type IX |
| 126 | Cytinaceae | cluster 1 | temperate element | cluster 6 | distribution supertype of north and south temperate regions | cluster 18 | distribution type XIV |
| 127 | Daphniphyllaceae | cluster 3 | tropical element | cluster 3 | distribution supertype of Old World tropics | cluster 5 | distribution type V |
| 128 | Dasypogonaceae | cluster 2 | temperate element | cluster 4 | distribution supertype of south temperate region | cluster 17 | distribution type XVII |
| 129 | Datiscaceae | cluster 2 | temperate element | cluster 5 | distribution supertype of north and south temperate regions | cluster 12 | distribution type XIV |
| 130 | Degeneriaceae | cluster 3 | tropical element | cluster 3 | distribution supertype of Old World tropics | cluster 10 | distribution type X |
| 131 | Diapensiaceae | cluster 2 | temperate element | cluster 5 | distribution supertype of north temperate region | cluster 12 | distribution type XII |
| 132 | Dichapetalaceae | cluster 3 | tropical element | cluster 2 | distribution supertype of tropics involving New World | cluster 2 | distribution type II |
| 133 | Didiereaceae | cluster 3 | tropical element | cluster 3 | distribution supertype of Old World tropics | cluster 11 | distribution type XI |
| 134 | Dilleniaceae | cluster 3 | tropical element | cluster 2 | distribution supertype of tropics involving New World | cluster 2 | distribution type II |
| 135 | Dioncophyllaceae | cluster 3 | tropical element | cluster 3 | distribution supertype of Old World tropics | cluster 11 | distribution type XI |
| 136 | Dioscoreaceae | cluster 3 | tropical element | cluster 2 | distribution supertype of tropics involving New World | cluster 2 | distribution type II |
| 137 | Dipentodontaceae | cluster 3 | tropical element | cluster 2 | distribution supertype of tropics involving New World | cluster 2 | distribution type III |
| 138 | Dipterocarpaceae | cluster 3 | tropical element | cluster 3 | distribution supertype of Old World tropics | cluster 6 | distribution type VI |
| 139 | Dirachmaceae | cluster 3 | tropical element | cluster 3 | distribution supertype of Old World tropics | cluster 11 | distribution type XI |
| 140 | Doryanthaceae | cluster 2 | temperate element | cluster 4 | distribution supertype of south temperate region | cluster 17 | distribution type XVII |
| 141 | Droseraceae | cluster 1 | cosmopolitan element | cluster 1 | cosmopolitan supertype | cluster 1 | distribution type I |
| 142 | Drosophyllaceae | cluster 2 | temperate element | cluster 5 | distribution supertype of north and south temperate regions | cluster 12 | distribution type XIV |
| 143 | Ebenaceae | cluster 3 | tropical element | cluster 2 | distribution supertype of tropics involving New World | cluster 2 | distribution type II |
| 144 | Ecdeiocoleaceae | cluster 2 | temperate element | cluster 4 | distribution supertype of south temperate region | cluster 17 | distribution type XVII |
| 145 | Elaeagnaceae | cluster 2 | temperate element | cluster 5 | distribution supertype of north temperate region | cluster 12 | distribution type XII |
| 146 | Elaeocarpaceae | cluster 3 | tropical element | cluster 2 | distribution supertype of tropics involving New World | cluster 2 | distribution type II |
| 147 | Elatinaceae | cluster 1 | cosmopolitan element | cluster 1 | cosmopolitan supertype | cluster 1 | distribution type I |
| 148 | Emblingiaceae | cluster 2 | temperate element | cluster 4 | distribution supertype of south temperate region | cluster 17 | distribution type XVII |
| 149 | Ephedraceae | cluster 1 | temperate element | cluster 6 | distribution supertype of north and south temperate regions | cluster 18 | distribution type XVIII |
| 150 | Ericaceae | cluster 1 | temperate element | cluster 6 | distribution supertype of north and south temperate regions | cluster 18 | distribution type XVIII |
| 151 | Eriocaulaceae | cluster 3 | tropical element | cluster 2 | distribution supertype of tropics involving New World | cluster 2 | distribution type II |
| 152 | Erythroxylaceae | cluster 3 | tropical element | cluster 2 | distribution supertype of tropics involving New World | cluster 2 | distribution type II |
| 153 | Escalloniaceae | cluster 3 | tropical element | cluster 4 | distribution supertype of tropics involving New World | cluster 3 | distribution type II |
| 154 | Eucommiaceae | cluster 2 | temperate element | cluster 5 | distribution supertype of north temperate region | cluster 15 | distribution type XV |
| 155 | Euphorbiaceae | cluster 3 | tropical element | cluster 2 | distribution supertype of tropics involving New World | cluster 2 | distribution type II |
| 156 | Euphroniaceae | cluster 3 | tropical element | cluster 3 | distribution supertype of tropics involving New World | cluster 9 | distribution type IX |
| 157 | Eupomatiaceae | cluster 3 | tropical element | cluster 4 | distribution supertype of Old World tropics | cluster 10 | distribution type X |
| 158 | Eupteleaceae | cluster 2 | temperate element | cluster 5 | distribution supertype of north temperate region | cluster 15 | distribution type XV |
| 159 | Fabaceae | cluster 1 | cosmopolitan element | cluster 1 | cosmopolitan supertype | cluster 1 | distribution type I |
| 160 | Fagaceae | cluster 2 | temperate element | cluster 5 | distribution supertype of north temperate region | cluster 12 | distribution type XII |
| 161 | Flagellariaceae | cluster 3 | tropical element | cluster 3 | distribution supertype of Old World tropics | cluster 4 | distribution type IV |
| 162 | Fouquieriaceae | cluster 2 | temperate element | cluster 5 | distribution supertype of north temperate region | cluster 16 | distribution type XVI |
| 163 | Francoaceae | cluster 3 | tropical element | cluster 4 | distribution supertype of tropics involving New World | cluster 11 | distribution type VII |
| 164 | Frankeniaceae | cluster 1 | temperate element | cluster 6 | distribution supertype of north and south temperate regions | cluster 18 | distribution type XIV |
| 165 | Garryaceae | cluster 2 | temperate element | cluster 5 | distribution supertype of north temperate region | cluster 12 | distribution type XIII |
| 166 | Geissolomataceae | cluster 2 | temperate element | cluster 4 | distribution supertype of south temperate region | cluster 11 | distribution type XVII |
| 167 | Gelsemiaceae | cluster 3 | tropical element | cluster 2 | distribution supertype of tropics involving New World | cluster 2 | distribution type II |
| 168 | Gentianaceae | cluster 1 | cosmopolitan element | cluster 1 | cosmopolitan supertype | cluster 1 | distribution type I |
| 169 | Geraniaceae | cluster 1 | temperate element | cluster 6 | distribution supertype of north and south temperate regions | cluster 18 | distribution type XVIII |
| 170 | Gerrardinaceae | cluster 3 | tropical element | cluster 3 | distribution supertype of Old World tropics | cluster 11 | distribution type XI |
| 171 | Gesneriaceae | cluster 3 | tropical element | cluster 2 | distribution supertype of tropics involving New World | cluster 2 | distribution type II |
| 172 | Ginkgoaceae | cluster 2 | temperate element | cluster 5 | distribution supertype of north temperate region | cluster 15 | distribution type XV |
| 173 | Gisekiaceae | cluster 3 | tropical element | cluster 3 | distribution supertype of Old World tropics | cluster 6 | distribution type VI |
| 174 | Gnetaceae | cluster 3 | tropical element | cluster 2 | distribution supertype of tropics involving New World | cluster 2 | distribution type II |
| 175 | Gomortegaceae | cluster 2 | temperate element | cluster 4 | distribution supertype of south temperate region | cluster 17 | distribution type XVII |
| 176 | Goodeniaceae | cluster 3 | tropical element | cluster 3 | distribution supertype of tropics involving New World | cluster 10 | distribution type II |
| 177 | Goupiaceae | cluster 3 | tropical element | cluster 2 | distribution supertype of tropics involving New World | cluster 9 | distribution type IX |
| 178 | Griseliniaceae | cluster 2 | temperate element | cluster 4 | distribution supertype of south temperate region | cluster 17 | distribution type XVII |
| 179 | Grossulariaceae | cluster 2 | temperate element | cluster 5 | distribution supertype of north and south temperate regions | cluster 12 | distribution type XVIII |
| 180 | Grubbiaceae | cluster 2 | temperate element | cluster 4 | distribution supertype of south temperate region | cluster 11 | distribution type XVII |
| 181 | Guamatelaceae | cluster 3 | tropical element | cluster 3 | distribution supertype of tropics involving New World | cluster 7 | distribution type IX |
| 182 | Gunneraceae | cluster 3 | tropical element | cluster 2 | distribution supertype of tropics involving New World | cluster 9 | distribution type II |
| 183 | Gyrostemonaceae | cluster 2 | temperate element | cluster 4 | distribution supertype of south temperate region | cluster 17 | distribution type XVII |
| 184 | Haemodoraceae | cluster 2 | temperate element | cluster 4 | distribution supertype of south temperate region | cluster 17 | distribution type XVII |
| 185 | Halophytaceae | cluster 2 | temperate element | cluster 4 | distribution supertype of south temperate region | cluster 17 | distribution type XVII |
| 186 | Haloragaceae | cluster 1 | cosmopolitan element | cluster 1 | cosmopolitan supertype | cluster 1 | distribution type I |
| 187 | Hamamelidaceae | cluster 3 | temperate element | cluster 2 | distribution supertype of north temperate region | cluster 4 | distribution type XII |
| 188 | Hanguanaceae | cluster 3 | tropical element | cluster 3 | distribution supertype of Old World tropics | cluster 5 | distribution type V |
| 189 | Heliconiaceae | cluster 3 | tropical element | cluster 2 | distribution supertype of tropics involving New World | cluster 9 | distribution type IX |
| 190 | Helwingiaceae | cluster 2 | temperate element | cluster 5 | distribution supertype of north temperate region | cluster 15 | distribution type XV |
| 191 | Hernandiaceae | cluster 3 | tropical element | cluster 2 | distribution supertype of tropics involving New World | cluster 2 | distribution type II |
| 192 | Himantandraceae | cluster 3 | tropical element | cluster 3 | distribution supertype of Old World tropics | cluster 5 | distribution type V |
| 193 | Huaceae | cluster 3 | tropical element | cluster 3 | distribution supertype of Old World tropics | cluster 11 | distribution type XI |
| 194 | Humiriaceae | cluster 3 | tropical element | cluster 2 | distribution supertype of tropics involving New World | cluster 9 | distribution type IX |
| 195 | Hydatellaceae | cluster 2 | temperate element | cluster 4 | distribution supertype of south temperate region | cluster 17 | distribution type XVII |
| 196 | Hydrangeaceae | cluster 2 | temperate element | cluster 5 | distribution supertype of north temperate region | cluster 12 | distribution type XIII |
| 197 | Hydrocharitaceae | cluster 1 | cosmopolitan element | cluster 1 | cosmopolitan supertype | cluster 1 | distribution type I |
| 198 | Hydroleaceae | cluster 3 | tropical element | cluster 2 | distribution supertype of tropics involving New World | cluster 2 | distribution type II |
| 199 | Hydrostachyaceae | cluster 3 | tropical element | cluster 3 | distribution supertype of Old World tropics | cluster 11 | distribution type XI |
| 200 | Hypericaceae | cluster 1 | cosmopolitan element | cluster 1 | cosmopolitan supertype | cluster 1 | distribution type I |
| 201 | Hypoxidaceae | cluster 3 | tropical element | cluster 2 | distribution supertype of tropics involving New World | cluster 4 | distribution type II |
| 202 | Icacinaceae | cluster 3 | tropical element | cluster 2 | distribution supertype of tropics involving New World | cluster 2 | distribution type II |
| 203 | Iridaceae | cluster 1 | cosmopolitan element | cluster 1 | cosmopolitan supertype | cluster 1 | distribution type I |
| 204 | Irvingiaceae | cluster 3 | tropical element | cluster 3 | distribution supertype of Old World tropics | cluster 6 | distribution type VI |
| 205 | Iteaceae | cluster 2 | temperate element | cluster 5 | distribution supertype of north temperate region | cluster 12 | distribution type XIII |
| 206 | Ixioliriaceae | cluster 2 | temperate element | cluster 5 | distribution supertype of north and south temperate regions | cluster 15 | distribution type XIV |
| 207 | Ixonanthaceae | cluster 3 | tropical element | cluster 2 | distribution supertype of tropics involving New World | cluster 2 | distribution type II |
| 208 | Joinvilleaceae | cluster 3 | tropical element | cluster 3 | distribution supertype of Old World tropics | cluster 5 | distribution type VIII |
| 209 | Juglandaceae | cluster 2 | temperate element | cluster 5 | distribution supertype of north temperate region | cluster 12 | distribution type XII |
| 210 | Juncaceae | cluster 1 | temperate element | cluster 6 | distribution supertype of north and south temperate regions | cluster 18 | distribution type XVIII |
| 211 | Juncaginaceae | cluster 1 | cosmopolitan element | cluster 1 | cosmopolitan supertype | cluster 1 | distribution type I |
| 212 | Kewaceae | cluster 2 | temperate element | cluster 4 | distribution supertype of south temperate region | cluster 11 | distribution type XVII |
| 213 | Kirkiaceae | cluster 3 | tropical element | cluster 3 | distribution supertype of Old World tropics | cluster 11 | distribution type XI |
| 214 | Koeberliniaceae | cluster 3 | tropical element | cluster 2 | distribution supertype of tropics involving New World | cluster 9 | distribution type IX |
| 215 | Krameriaceae | cluster 3 | tropical element | cluster 2 | distribution supertype of tropics involving New World | cluster 9 | distribution type IX |
| 216 | Lacistemataceae | cluster 3 | tropical element | cluster 2 | distribution supertype of tropics involving New World | cluster 9 | distribution type IX |
| 217 | Lamiaceae | cluster 1 | cosmopolitan element | cluster 1 | cosmopolitan supertype | cluster 1 | distribution type I |
| 218 | Lanariaceae | cluster 2 | temperate element | cluster 4 | distribution supertype of south temperate region | cluster 11 | distribution type XVII |
| 219 | Lardizabalaceae | cluster 2 | temperate element | cluster 5 | distribution supertype of north and south temperate regions | cluster 15 | distribution type XVIII |
| 220 | Lauraceae | cluster 3 | tropical element | cluster 2 | distribution supertype of tropics involving New World | cluster 2 | distribution type II |
| 221 | Lecythidaceae | cluster 3 | tropical element | cluster 2 | distribution supertype of tropics involving New World | cluster 2 | distribution type II |
| 222 | Lentibulariaceae | cluster 1 | cosmopolitan element | cluster 1 | cosmopolitan supertype | cluster 1 | distribution type I |
| 223 | Lepidobotryaceae | cluster 3 | tropical element | cluster 2 | distribution supertype of tropics involving New World | cluster 9 | distribution type VII |
| 224 | Liliaceae | cluster 2 | temperate element | cluster 5 | distribution supertype of north temperate region | cluster 12 | distribution type XII |
| 225 | Limeaceae | cluster 3 | tropical element | cluster 3 | distribution supertype of Old World tropics | cluster 6 | distribution type VI |
| 226 | Limnanthaceae | cluster 2 | temperate element | cluster 5 | distribution supertype of north temperate region | cluster 16 | distribution type XVI |
| 227 | Linaceae | cluster 1 | cosmopolitan element | cluster 1 | cosmopolitan supertype | cluster 1 | distribution type I |
| 228 | Linderniaceae | cluster 3 | tropical element | cluster 2 | distribution supertype of tropics involving New World | cluster 4 | distribution type II |
| 229 | Loasaceae | cluster 3 | tropical element | cluster 2 | distribution supertype of tropics involving New World | cluster 9 | distribution type IX |
| 230 | Loganiaceae | cluster 3 | tropical element | cluster 2 | distribution supertype of tropics involving New World | cluster 2 | distribution type II |
| 231 | Lophiocarpaceae | cluster 3 | tropical element | cluster 3 | distribution supertype of Old World tropics | cluster 6 | distribution type VI |
| 232 | Lophopyxidaceae | cluster 3 | tropical element | cluster 3 | distribution supertype of Old World tropics | cluster 5 | distribution type VIII |
| 233 | Loranthaceae | cluster 3 | tropical element | cluster 2 | distribution supertype of tropics involving New World | cluster 2 | distribution type II |
| 234 | Lowiaceae | cluster 3 | tropical element | cluster 3 | distribution supertype of Old World tropics | cluster 5 | distribution type VIII |
| 235 | Lythraceae | cluster 1 | cosmopolitan element | cluster 1 | cosmopolitan supertype | cluster 1 | distribution type I |
| 236 | Macarthuriaceae | cluster 2 | temperate element | cluster 4 | distribution supertype of south temperate region | cluster 17 | distribution type XVII |
| 237 | Magnoliaceae | cluster 3 | temperate element | cluster 2 | distribution supertype of north temperate region | cluster 13 | distribution type XIII |
| 238 | Malpighiaceae | cluster 3 | tropical element | cluster 2 | distribution supertype of tropics involving New World | cluster 2 | distribution type II |
| 239 | Malvaceae | cluster 3 | tropical element | cluster 2 | distribution supertype of tropics involving New World | cluster 2 | distribution type II |
| 240 | Marantaceae | cluster 3 | tropical element | cluster 2 | distribution supertype of tropics involving New World | cluster 2 | distribution type II |
| 241 | Marcgraviaceae | cluster 3 | tropical element | cluster 2 | distribution supertype of tropics involving New World | cluster 9 | distribution type IX |
| 242 | Martyniaceae | cluster 3 | tropical element | cluster 2 | distribution supertype of tropics involving New World | cluster 9 | distribution type IX |
| 243 | Maundiaceae | cluster 2 | temperate element | cluster 4 | distribution supertype of south temperate region | cluster 17 | distribution type XVII |
| 244 | Mayacaceae | cluster 3 | tropical element | cluster 2 | distribution supertype of tropics involving New World | cluster 9 | distribution type IX |
| 245 | Mazaceae | cluster 2 | temperate element | cluster 4 | distribution supertype of north and south temperate regions | cluster 14 | distribution type XVIII |
| 246 | Melanthiaceae | cluster 2 | temperate element | cluster 5 | distribution supertype of north temperate region | cluster 12 | distribution type XII |
| 247 | Melastomataceae | cluster 3 | tropical element | cluster 2 | distribution supertype of tropics involving New World | cluster 2 | distribution type II |
| 248 | Meliaceae | cluster 3 | tropical element | cluster 2 | distribution supertype of tropics involving New World | cluster 2 | distribution type II |
| 249 | Menispermaceae | cluster 3 | tropical element | cluster 2 | distribution supertype of tropics involving New World | cluster 2 | distribution type II |
| 250 | Menyanthaceae | cluster 1 | cosmopolitan element | cluster 1 | cosmopolitan supertype | cluster 1 | distribution type I |
| 251 | Metteniusaceae | cluster 3 | tropical element | cluster 2 | distribution supertype of tropics involving New World | cluster 2 | distribution type II |
| 252 | Microteaceae | cluster 3 | tropical element | cluster 2 | distribution supertype of tropics involving New World | cluster 9 | distribution type IX |
| 253 | Misodendraceae | cluster 2 | temperate element | cluster 4 | distribution supertype of south temperate region | cluster 17 | distribution type XVII |
| 254 | Mitrastemonaceae | cluster 3 | tropical element | cluster 2 | distribution supertype of tropics involving New World | cluster 2 | distribution type III |
| 255 | Molluginaceae | cluster 3 | tropical element | cluster 2 | distribution supertype of tropics involving New World | cluster 2 | distribution type II |
| 256 | Monimiaceae | cluster 3 | tropical element | cluster 2 | distribution supertype of tropics involving New World | cluster 2 | distribution type II |
| 257 | Montiaceae | cluster 1 | temperate element | cluster 6 | distribution supertype of north and south temperate regions | cluster 18 | distribution type XVIII |
| 258 | Montiniaceae | cluster 3 | tropical element | cluster 3 | distribution supertype of Old World tropics | cluster 11 | distribution type XI |
| 259 | Moraceae | cluster 1 | cosmopolitan element | cluster 1 | cosmopolitan supertype | cluster 1 | distribution type I |
| 260 | Moringaceae | cluster 3 | tropical element | cluster 3 | distribution supertype of Old World tropics | cluster 6 | distribution type VI |
| 261 | Muntingiaceae | cluster 3 | tropical element | cluster 2 | distribution supertype of tropics involving New World | cluster 9 | distribution type IX |
| 262 | Musaceae | cluster 3 | tropical element | cluster 3 | distribution supertype of Old World tropics | cluster 4 | distribution type IV |
| 263 | Myodocarpaceae | cluster 3 | tropical element | cluster 3 | distribution supertype of Old World tropics | cluster 5 | distribution type V |
| 264 | Myricaceae | cluster 1 | cosmopolitan element | cluster 1 | cosmopolitan supertype | cluster 1 | distribution type I |
| 265 | Myristicaceae | cluster 3 | tropical element | cluster 2 | distribution supertype of tropics involving New World | cluster 2 | distribution type II |
| 266 | Myrothamnaceae | cluster 3 | tropical element | cluster 3 | distribution supertype of Old World tropics | cluster 11 | distribution type XI |
| 267 | Myrtaceae | cluster 1 | tropical element | cluster 2 | distribution supertype of tropics involving New World | cluster 2 | distribution type II |
| 268 | Nartheciaceae | cluster 2 | temperate element | cluster 5 | distribution supertype of north temperate region | cluster 12 | distribution type XII |
| 269 | Nelumbonaceae | cluster 2 | temperate element | cluster 5 | distribution supertype of north temperate region | cluster 12 | distribution type XIII |
| 270 | Nepenthaceae | cluster 3 | tropical element | cluster 3 | distribution supertype of Old World tropics | cluster 5 | distribution type V |
| 271 | Neuradaceae | cluster 2 | temperate element | cluster 5 | distribution supertype of north and south temperate regions | cluster 14 | distribution type XIV |
| 272 | Nitrariaceae | cluster 1 | temperate element | cluster 6 | distribution supertype of north and south temperate regions | cluster 18 | distribution type XIV |
| 273 | Nothofagaceae | cluster 2 | temperate element | cluster 4 | distribution supertype of south temperate region | cluster 17 | distribution type XVII |
| 274 | Nyctaginaceae | cluster 3 | tropical element | cluster 2 | distribution supertype of tropics involving New World | cluster 2 | distribution type II |
| 275 | Nymphaeaceae | cluster 1 | cosmopolitan element | cluster 1 | cosmopolitan supertype | cluster 1 | distribution type I |
| 276 | Nyssaceae | cluster 2 | temperate element | cluster 5 | distribution supertype of north temperate region | cluster 13 | distribution type XIII |
| 277 | Ochnaceae | cluster 3 | tropical element | cluster 2 | distribution supertype of tropics involving New World | cluster 2 | distribution type II |
| 278 | Olacaceae | cluster 3 | tropical element | cluster 2 | distribution supertype of tropics involving New World | cluster 2 | distribution type II |
| 279 | Oleaceae | cluster 1 | cosmopolitan element | cluster 1 | cosmopolitan supertype | cluster 1 | distribution type I |
| 280 | Onagraceae | cluster 1 | cosmopolitan element | cluster 1 | cosmopolitan supertype | cluster 1 | distribution type I |
| 281 | Oncothecaceae | cluster 3 | tropical element | cluster 3 | distribution supertype of Old World tropics | cluster 10 | distribution type X |
| 282 | Opiliaceae | cluster 3 | tropical element | cluster 2 | distribution supertype of tropics involving New World | cluster 2 | distribution type II |
| 283 | Orchidaceae | cluster 1 | cosmopolitan element | cluster 1 | cosmopolitan supertype | cluster 1 | distribution type I |
| 284 | Orobanchaceae | cluster 1 | cosmopolitan element | cluster 1 | cosmopolitan supertype | cluster 1 | distribution type I |
| 285 | Oxalidaceae | cluster 1 | cosmopolitan element | cluster 1 | cosmopolitan supertype | cluster 1 | distribution type I |
| 286 | Paeoniaceae | cluster 2 | temperate element | cluster 5 | distribution supertype of north temperate region | cluster 12 | distribution type XII |
| 287 | Pandaceae | cluster 3 | tropical element | cluster 3 | distribution supertype of Old World tropics | cluster 4 | distribution type VI |
| 288 | Pandanaceae | cluster 3 | tropical element | cluster 3 | distribution supertype of Old World tropics | cluster 4 | distribution type IV |
| 289 | Papaveraceae | cluster 2 | temperate element | cluster 5 | distribution supertype of north and south temperate regions | cluster 12 | distribution type XVIII |
| 290 | Paracryphiaceae | cluster 3 | tropical element | cluster 3 | distribution supertype of Old World tropics | cluster 5 | distribution type V |
| 291 | Passifloraceae | cluster 3 | tropical element | cluster 2 | distribution supertype of tropics involving New World | cluster 2 | distribution type II |
| 292 | Paulowniaceae | cluster 2 | temperate element | cluster 5 | distribution supertype of north temperate region | cluster 15 | distribution type XV |
| 293 | Pedaliaceae | cluster 3 | tropical element | cluster 3 | distribution supertype of Old World tropics | cluster 4 | distribution type IV |
| 294 | Penaeaceae | cluster 2 | temperate element | cluster 4 | distribution supertype of south temperate region | cluster 11 | distribution type XVII |
| 295 | Pennantiaceae | cluster 2 | temperate element | cluster 4 | distribution supertype of south temperate region | cluster 17 | distribution type XVII |
| 296 | Pentadiplandraceae | cluster 3 | tropical element | cluster 3 | distribution supertype of Old World tropics | cluster 11 | distribution type XI |
| 297 | Pentaphragmataceae | cluster 3 | tropical element | cluster 3 | distribution supertype of Old World tropics | cluster 5 | distribution type VIII |
| 298 | Pentaphylacaceae | cluster 3 | tropical element | cluster 2 | distribution supertype of tropics involving New World | cluster 2 | distribution type II |
| 299 | Penthoraceae | cluster 2 | temperate element | cluster 5 | distribution supertype of north temperate region | cluster 12 | distribution type XIII |
| 300 | Peraceae | cluster 3 | tropical element | cluster 2 | distribution supertype of tropics involving New World | cluster 2 | distribution type II |
| 301 | Peridiscaceae | cluster 3 | tropical element | cluster 3 | distribution supertype of tropics involving New World | cluster 11 | distribution type VII |
| 302 | Petenaeaceae | cluster 3 | tropical element | cluster 3 | distribution supertype of tropics involving New World | cluster 7 | distribution type IX |
| 303 | Petermanniaceae | cluster 2 | temperate element | cluster 4 | distribution supertype of south temperate region | cluster 17 | distribution type XVII |
| 304 | Petiveriaceae | cluster 3 | tropical element | cluster 2 | distribution supertype of tropics involving New World | cluster 9 | distribution type IX |
| 305 | Petrosaviaceae | cluster 3 | tropical element | cluster 3 | distribution supertype of Old World tropics | cluster 5 | distribution type VIII |
| 306 | Phellinaceae | cluster 3 | tropical element | cluster 3 | distribution supertype of Old World tropics | cluster 10 | distribution type X |
| 307 | Philesiaceae | cluster 2 | temperate element | cluster 4 | distribution supertype of south temperate region | cluster 17 | distribution type XVII |
| 308 | Philydraceae | cluster 3 | tropical element | cluster 3 | distribution supertype of Old World tropics | cluster 5 | distribution type V |
| 309 | Phrymaceae | cluster 1 | cosmopolitan element | cluster 1 | cosmopolitan supertype | cluster 1 | distribution type I |
| 310 | Phyllanthaceae | cluster 3 | tropical element | cluster 2 | distribution supertype of tropics involving New World | cluster 2 | distribution type II |
| 311 | Phyllonomaceae | cluster 3 | tropical element | cluster 2 | distribution supertype of tropics involving New World | cluster 9 | distribution type IX |
| 312 | Physenaceae | cluster 3 | tropical element | cluster 3 | distribution supertype of Old World tropics | cluster 11 | distribution type XI |
| 313 | Phytolaccaceae | cluster 3 | tropical element | cluster 2 | distribution supertype of tropics involving New World | cluster 2 | distribution type II |
| 314 | Picramniaceae | cluster 3 | tropical element | cluster 2 | distribution supertype of tropics involving New World | cluster 9 | distribution type IX |
| 315 | Picrodendraceae | cluster 3 | tropical element | cluster 2 | distribution supertype of tropics involving New World | cluster 2 | distribution type II |
| 316 | Pinaceae | cluster 2 | temperate element | cluster 5 | distribution supertype of north temperate region | cluster 12 | distribution type XII |
| 317 | Piperaceae | cluster 3 | tropical element | cluster 2 | distribution supertype of tropics involving New World | cluster 2 | distribution type II |
| 318 | Pittosporaceae | cluster 3 | tropical element | cluster 3 | distribution supertype of Old World tropics | cluster 4 | distribution type IV |
| 319 | Plantaginaceae | cluster 1 | cosmopolitan element | cluster 1 | cosmopolitan supertype | cluster 1 | distribution type I |
| 320 | Platanaceae | cluster 2 | temperate element | cluster 5 | distribution supertype of north temperate region | cluster 12 | distribution type XVI |
| 321 | Plocospermataceae | cluster 3 | tropical element | cluster 3 | distribution supertype of tropics involving New World | cluster 7 | distribution type IX |
| 322 | Plumbaginaceae | cluster 1 | cosmopolitan element | cluster 1 | cosmopolitan supertype | cluster 14 | distribution type I |
| 323 | Poaceae | cluster 1 | cosmopolitan element | cluster 1 | cosmopolitan supertype | cluster 1 | distribution type I |
| 324 | Podocarpaceae | cluster 3 | tropical element | cluster 2 | distribution supertype of tropics involving New World | cluster 2 | distribution type II |
| 325 | Podostemaceae | cluster 3 | tropical element | cluster 2 | distribution supertype of tropics involving New World | cluster 2 | distribution type II |
| 326 | Polemoniaceae | cluster 2 | temperate element | cluster 5 | distribution supertype of north and south temperate regions | cluster 12 | distribution type XVIII |
| 327 | Polygalaceae | cluster 1 | cosmopolitan element | cluster 1 | cosmopolitan supertype | cluster 1 | distribution type I |
| 328 | Polygonaceae | cluster 1 | cosmopolitan element | cluster 1 | cosmopolitan supertype | cluster 1 | distribution type I |
| 329 | Pontederiaceae | cluster 3 | tropical element | cluster 2 | distribution supertype of tropics involving New World | cluster 2 | distribution type II |
| 330 | Portulacaceae | cluster 1 | cosmopolitan element | cluster 1 | cosmopolitan supertype | cluster 1 | distribution type I |
| 331 | Posidoniaceae | cluster 2 | temperate element | cluster 6 | distribution supertype of north and south temperate regions | cluster 14 | distribution type XIV |
| 332 | Potamogetonaceae | cluster 1 | cosmopolitan element | cluster 1 | cosmopolitan supertype | cluster 1 | distribution type I |
| 333 | Primulaceae | cluster 1 | cosmopolitan element | cluster 1 | cosmopolitan supertype | cluster 1 | distribution type I |
| 334 | Proteaceae | cluster 3 | tropical element | cluster 3 | distribution supertype of tropics involving New World | cluster 10 | distribution type II |
| 335 | Putranjivaceae | cluster 3 | tropical element | cluster 2 | distribution supertype of tropics involving New World | cluster 2 | distribution type II |
| 336 | Quillajaceae | cluster 2 | temperate element | cluster 4 | distribution supertype of south temperate region | cluster 17 | distribution type XVII |
| 337 | Rafflesiaceae | cluster 3 | tropical element | cluster 3 | distribution supertype of Old World tropics | cluster 5 | distribution type VIII |
| 338 | Ranunculaceae | cluster 1 | cosmopolitan element | cluster 1 | cosmopolitan supertype | cluster 1 | distribution type I |
| 339 | Rapateaceae | cluster 3 | tropical element | cluster 3 | distribution supertype of tropics involving New World | cluster 9 | distribution type IX |
| 340 | Resedaceae | cluster 2 | temperate element | cluster 5 | distribution supertype of north temperate region | cluster 12 | distribution type XII |
| 341 | Restionaceae | cluster 2 | temperate element | cluster 4 | distribution supertype of south temperate region | cluster 17 | distribution type XVII |
| 342 | Rhabdodendraceae | cluster 3 | tropical element | cluster 3 | distribution supertype of tropics involving New World | cluster 9 | distribution type IX |
| 343 | Rhamnaceae | cluster 1 | cosmopolitan element | cluster 1 | cosmopolitan supertype | cluster 1 | distribution type I |
| 344 | Rhizophoraceae | cluster 3 | tropical element | cluster 2 | distribution supertype of tropics involving New World | cluster 2 | distribution type II |
| 345 | Ripogonaceae | cluster 3 | tropical element | cluster 3 | distribution supertype of Old World tropics | cluster 10 | distribution type X |
| 346 | Roridulaceae | cluster 2 | temperate element | cluster 4 | distribution supertype of south temperate region | cluster 11 | distribution type XVII |
| 347 | Rosaceae | cluster 1 | cosmopolitan element | cluster 1 | cosmopolitan supertype | cluster 1 | distribution type I |
| 348 | Rousseaceae | cluster 3 | tropical element | cluster 3 | distribution supertype of Old World tropics | cluster 10 | distribution type X |
| 349 | Rubiaceae | cluster 1 | cosmopolitan element | cluster 1 | cosmopolitan supertype | cluster 1 | distribution type I |
| 350 | Ruppiaceae | cluster 1 | cosmopolitan element | cluster 1 | cosmopolitan supertype | cluster 8 | distribution type I |
| 351 | Rutaceae | cluster 3 | tropical element | cluster 2 | distribution supertype of tropics involving New World | cluster 2 | distribution type II |
| 352 | Sabiaceae | cluster 3 | tropical element | cluster 2 | distribution supertype of tropics involving New World | cluster 9 | distribution type III |
| 353 | Salicaceae | cluster 1 | cosmopolitan element | cluster 1 | cosmopolitan supertype | cluster 1 | distribution type I |
| 354 | Salvadoraceae | cluster 3 | tropical element | cluster 3 | distribution supertype of Old World tropics | cluster 6 | distribution type VI |
| 355 | Santalaceae | cluster 1 | cosmopolitan element | cluster 1 | cosmopolitan supertype | cluster 1 | distribution type I |
| 356 | Sapindaceae | cluster 3 | tropical element | cluster 2 | distribution supertype of tropics involving New World | cluster 2 | distribution type II |
| 357 | Sapotaceae | cluster 3 | tropical element | cluster 2 | distribution supertype of tropics involving New World | cluster 2 | distribution type II |
| 358 | Sarcobataceae | cluster 2 | temperate element | cluster 5 | distribution supertype of north temperate region | cluster 16 | distribution type XVI |
| 359 | Sarcolaenaceae | cluster 3 | tropical element | cluster 3 | distribution supertype of Old World tropics | cluster 11 | distribution type XI |
| 360 | Sarraceniaceae | cluster 3 | temperate element | cluster 2 | distribution supertype of north temperate region | cluster 9 | distribution type XVI |
| 361 | Saururaceae | cluster 2 | temperate element | cluster 5 | distribution supertype of north temperate region | cluster 12 | distribution type XIII |
| 362 | Saxifragaceae | cluster 2 | temperate element | cluster 5 | distribution supertype of north and south temperate regions | cluster 12 | distribution type XVIII |
| 363 | Scheuchzeriaceae | cluster 2 | temperate element | cluster 5 | distribution supertype of north temperate region | cluster 12 | distribution type XII |
| 364 | Schisandraceae | cluster 2 | temperate element | cluster 5 | distribution supertype of north temperate region | cluster 12 | distribution type XIII |
| 365 | Schlegeliaceae | cluster 3 | tropical element | cluster 2 | distribution supertype of tropics involving New World | cluster 9 | distribution type IX |
| 366 | Schoepfiaceae | cluster 3 | tropical element | cluster 2 | distribution supertype of tropics involving New World | cluster 3 | distribution type III |
| 367 | Sciadopityaceae | cluster 2 | temperate element | cluster 5 | distribution supertype of north temperate region | cluster 15 | distribution type XV |
| 368 | Scrophulariaceae | cluster 1 | cosmopolitan element | cluster 1 | cosmopolitan supertype | cluster 1 | distribution type I |
| 369 | Setchellanthaceae | cluster 2 | temperate element | cluster 5 | distribution supertype of north temperate region | cluster 16 | distribution type XVI |
| 370 | Simaroubaceae | cluster 3 | tropical element | cluster 2 | distribution supertype of tropics involving New World | cluster 2 | distribution type II |
| 371 | Simmondsiaceae | cluster 2 | temperate element | cluster 5 | distribution supertype of north temperate region | cluster 16 | distribution type XVI |
| 372 | Siparunaceae | cluster 3 | tropical element | cluster 2 | distribution supertype of tropics involving New World | cluster 9 | distribution type VII |
| 373 | Sladeniaceae | cluster 3 | tropical element | cluster 3 | distribution supertype of Old World tropics | cluster 6 | distribution type VI |
| 374 | Smilacaceae | cluster 3 | tropical element | cluster 2 | distribution supertype of tropics involving New World | cluster 2 | distribution type II |
| 375 | Solanaceae | cluster 1 | cosmopolitan element | cluster 1 | cosmopolitan supertype | cluster 1 | distribution type I |
| 376 | Sphaerosepalaceae | cluster 3 | tropical element | cluster 3 | distribution supertype of Old World tropics | cluster 11 | distribution type XI |
| 377 | Sphenocleaceae | cluster 3 | tropical element | cluster 3 | distribution supertype of Old World tropics | cluster 4 | distribution type IV |
| 378 | Stachyuraceae | cluster 2 | temperate element | cluster 5 | distribution supertype of north temperate region | cluster 15 | distribution type XV |
| 379 | Staphyleaceae | cluster 3 | tropical element | cluster 2 | distribution supertype of tropics involving New World | cluster 2 | distribution type III |
| 380 | Stegnospermataceae | cluster 2 | temperate element | cluster 5 | distribution supertype of north temperate region | cluster 16 | distribution type XVI |
| 381 | Stemonaceae | cluster 3 | tropical element | cluster 3 | distribution supertype of Old World tropics | cluster 5 | distribution type V |
| 382 | Stemonuraceae | cluster 3 | tropical element | cluster 3 | distribution supertype of tropics involving New World | cluster 4 | distribution type II |
| 383 | Stilbaceae | cluster 2 | temperate element | cluster 4 | distribution supertype of south temperate region | cluster 14 | distribution type XVII |
| 384 | Strasburgeriaceae | cluster 3 | tropical element | cluster 4 | distribution supertype of Old World tropics | cluster 10 | distribution type X |
| 385 | Strelitziaceae | cluster 3 | tropical element | cluster 4 | distribution supertype of tropics involving New World | cluster 11 | distribution type VII |
| 386 | Stylidiaceae | cluster 2 | temperate element | cluster 4 | distribution supertype of south temperate region | cluster 17 | distribution type XVII |
| 387 | Styracaceae | cluster 3 | tropical element | cluster 2 | distribution supertype of tropics involving New World | cluster 9 | distribution type III |
| 388 | Surianaceae | cluster 3 | tropical element | cluster 2 | distribution supertype of tropics involving New World | cluster 2 | distribution type II |
| 389 | Symplocaceae | cluster 3 | tropical element | cluster 2 | distribution supertype of tropics involving New World | cluster 2 | distribution type II |
| 390 | Talinaceae | cluster 3 | tropical element | cluster 2 | distribution supertype of tropics involving New World | cluster 9 | distribution type VII |
| 391 | Tamaricaceae | cluster 2 | temperate element | cluster 5 | distribution supertype of north temperate region | cluster 12 | distribution type XII |
| 392 | Tapisciaceae | cluster 3 | tropical element | cluster 2 | distribution supertype of tropics involving New World | cluster 9 | distribution type III |
| 393 | Taxaceae | cluster 1 | temperate element | cluster 6 | distribution supertype of north temperate region | cluster 18 | distribution type XII |
| 394 | Tecophilaeaceae | cluster 2 | temperate element | cluster 4 | distribution supertype of north and south temperate regions | cluster 17 | distribution type XIV |
| 395 | Tetracarpaeaceae | cluster 2 | temperate element | cluster 4 | distribution supertype of south temperate region | cluster 17 | distribution type XVII |
| 396 | Tetrachondraceae | cluster 1 | temperate element | cluster 1 | distribution supertype of north and south temperate regions | cluster 1 | distribution type XVIII |
| 397 | Tetramelaceae | cluster 3 | tropical element | cluster 3 | distribution supertype of Old World tropics | cluster 5 | distribution type V |
| 398 | Tetrameristaceae | cluster 3 | tropical element | cluster 2 | distribution supertype of tropics involving New World | cluster 9 | distribution type III |
| 399 | Theaceae | cluster 3 | tropical element | cluster 3 | distribution supertype of tropics involving New World | cluster 7 | distribution type III |
| 400 | Thomandersiaceae | cluster 3 | tropical element | cluster 3 | distribution supertype of Old World tropics | cluster 11 | distribution type XI |
| 401 | Thurniaceae | cluster 3 | tropical element | cluster 4 | distribution supertype of tropics involving New World | cluster 11 | distribution type VII |
| 402 | Thymelaeaceae | cluster 1 | cosmopolitan element | cluster 1 | cosmopolitan supertype | cluster 1 | distribution type I |
| 403 | Ticodendraceae | cluster 3 | tropical element | cluster 3 | distribution supertype of tropics involving New World | cluster 7 | distribution type IX |
| 404 | Tiganophytaceae | cluster 2 | temperate element | cluster 4 | distribution supertype of south temperate region | cluster 11 | distribution type XVII |
| 405 | Tofieldiaceae | cluster 2 | temperate element | cluster 5 | distribution supertype of north temperate region | cluster 12 | distribution type XII |
| 406 | Torricelliaceae | cluster 3 | tropical element | cluster 3 | distribution supertype of Old World tropics | cluster 6 | distribution type VI |
| 407 | Tovariaceae | cluster 3 | tropical element | cluster 2 | distribution supertype of tropics involving New World | cluster 9 | distribution type IX |
| 408 | Trigoniaceae | cluster 3 | tropical element | cluster 2 | distribution supertype of tropics involving New World | cluster 9 | distribution type IX |
| 409 | Trimeniaceae | cluster 3 | tropical element | cluster 3 | distribution supertype of Old World tropics | cluster 5 | distribution type V |
| 410 | Triuridaceae | cluster 3 | tropical element | cluster 2 | distribution supertype of tropics involving New World | cluster 2 | distribution type II |
| 411 | Trochodendraceae | cluster 2 | temperate element | cluster 5 | distribution supertype of north temperate region | cluster 15 | distribution type XV |
| 412 | Tropaeolaceae | cluster 2 | temperate element | cluster 4 | distribution supertype of south temperate region | cluster 17 | distribution type XVII |
| 413 | Typhaceae | cluster 1 | cosmopolitan element | cluster 1 | cosmopolitan supertype | cluster 1 | distribution type I |
| 414 | Ulmaceae | cluster 1 | cosmopolitan element | cluster 5 | cosmopolitan supertype | cluster 13 | distribution type I |
| 415 | Urticaceae | cluster 3 | tropical element | cluster 2 | distribution supertype of tropics involving New World | cluster 2 | distribution type II |
| 416 | Vahliaceae | cluster 3 | tropical element | cluster 3 | distribution supertype of Old World tropics | cluster 6 | distribution type VI |
| 417 | Velloziaceae | cluster 3 | tropical element | cluster 3 | distribution supertype of tropics involving New World | cluster 11 | distribution type VII |
| 418 | Verbenaceae | cluster 1 | cosmopolitan element | cluster 1 | cosmopolitan supertype | cluster 1 | distribution type I |
| 419 | Violaceae | cluster 1 | cosmopolitan element | cluster 1 | cosmopolitan supertype | cluster 1 | distribution type I |
| 420 | Vitaceae | cluster 3 | tropical element | cluster 2 | distribution supertype of tropics involving New World | cluster 2 | distribution type II |
| 421 | Vochysiaceae | cluster 3 | tropical element | cluster 2 | distribution supertype of tropics involving New World | cluster 9 | distribution type IX |
| 422 | Welwitschiaceae | cluster 3 | tropical element | cluster 3 | distribution supertype of Old World tropics | cluster 11 | distribution type XI |
| 423 | Winteraceae | cluster 3 | tropical element | cluster 3 | distribution supertype of tropics involving New World | cluster 10 | distribution type II |
| 424 | Xeronemataceae | cluster 3 | tropical element | cluster 4 | distribution supertype of Old World tropics | cluster 10 | distribution type X |
| 425 | Xyridaceae | cluster 3 | tropical element | cluster 2 | distribution supertype of tropics involving New World | cluster 2 | distribution type II |
| 426 | Zamiaceae | cluster 3 | tropical element | cluster 2 | distribution supertype of tropics involving New World | cluster 2 | distribution type II |
| 427 | Zingiberaceae | cluster 3 | tropical element | cluster 3 | distribution supertype of Old World tropics | cluster 4 | distribution type V |
| 428 | Zosteraceae | cluster 1 | temperate element | cluster 6 | distribution supertype of north and south temperate regions | cluster 18 | distribution type XVIII |
| 429 | Zygophyllaceae | cluster 1 | cosmopolitan element | cluster 1 | cosmopolitan supertype | cluster 1 | distribution type I |

**Table S2. Performance of clustering algorithms for beta diversity (****βsim) of distribution data of 18 distribution type**

| **Clustering algorithms** | **Cophenetic correlation coefficient** | **Gower’s distance** |
| --- | --- | --- |
| UPGMA | 0.78 | 13.19 |
| UPGMC | 0.43 | 151.05 |
| ward.D2 | 0.70 | 443.39 |
| SL | 0.60 | 72.21 |
| CL | 0.73 | 53.45 |
| **WPGMA** | **0.80** | **12.10** |
| WPGMC | 0.57 | 123.77 |

Abbreviations: UPGMA, unweighted pair-group method using arithmetic averages; UPGMC, unweighted pair-group method using centroids; ward.D2, Ward's minimum variance D2; SL, single linkage; CL, complete lineage; WPGMA, weighted pair-group method using arithmetic averages; WPGMC, weighted pair-group method using centroids. The best clustering algorithm is shown in bold.

**Table S3. Basic information, including** **dispersal type, life form, and** **stem age, of 429 spermatophyte families**

| **Number** | **family** | **Distribution type (including distribution subtype)** | **Age** | | **Dispersal type** | | | | **Life form** |
| --- | --- | --- | --- | --- | --- | --- | --- | --- | --- |
|  |  |  | **Stem age** | **Ln stem age** | **Animal dispersal (1 presents yes, 0 presents no)** | **Water dispersal (1 presents yes, 0 presents no)** | **Wind dispersal (1 presents yes, 0 presents no)** | **Note** |  |
| 1 | Acanthaceae | II | 41.89 | 3.74 | 1 | 0 | 1 |  | Mixed |
| 2 | Achariaceae | II.II | 90.00 | 4.50 | 0 | 0 | 0 |  | Mixed |
| 3 | Achatocarpaceae | IX | 64.16 | 4.16 | 1 | 0 | 0 |  | Woody |
| 4 | Acoraceae | XIII | 133.23 | 4.89 | 0 | 1 | 0 |  | Herbaceous |
| 5 | Actinidiaceae | III | 84.81 | 4.44 | 1 | 0 | 0 |  | Woody |
| 6 | Adoxaceae | XII | 70.94 | 4.26 | 1 | 0 | 0 |  | Mixed |
| 7 | Aextoxicaceae | XVII.I | 27.13 | 3.30 | 0 | 0 | 0 |  | Woody |
| 8 | Aizoaceae | II | 74.88 | 4.32 | 1 | 1 | 1 |  | Mixed |
| 9 | Akaniaceae | V | 71.11 | 4.26 | 0 | 0 | 0 |  | Woody |
| 10 | Alismataceae | I | 72.05 | 4.28 | 1 | 1 | 0 |  | Herbaceous |
| 11 | Alseuosmiaceae | X | 53.50 | 3.98 | 0 | 0 | 0 |  | Woody |
| 12 | Alstroemeriaceae | XVII | 59.12 | 4.08 | 1 | 0 | 0 |  | Herbaceous |
| 13 | Altingiaceae | XIII | 92.67 | 4.53 | 1 | 0 | 1 |  | Woody |
| 14 | Alzateaceae | IX | 30.89 | 3.43 | 0 | 0 | 0 |  | Woody |
| 15 | Amaranthaceae | I | 64.16 | 4.16 | 1 | 1 | 1 |  | Mixed |
| 16 | Amaryllidaceae | I | 62.49 | 4.14 | 1 | 1 | 1 |  | Mixed |
| 17 | Amborellaceae | X.I | 139.40 | 4.94 | 0 | 0 | 0 |  | Woody |
| 18 | Anacampserotaceae | XVII | 28.10 | 3.34 | 1 | 0 | 1 |  | Mixed |
| 19 | Anacardiaceae | II | 51.56 | 3.94 | 1 | 1 | 1 |  | Woody |
| 20 | Ancistrocladaceae | VI | 36.16 | 3.59 | 0 | 1 | 1 |  | Woody |
| 21 | Anisophylleaceae | II.II | 64.60 | 4.17 | 0 | 1 | 0 |  | Woody |
| 22 | Annonaceae | II | 94.22 | 4.55 | 1 | 0 | 0 |  | Woody |
| 23 | Aphanopetalaceae | X | 83.59 | 4.43 | 0 | 0 | 0 |  | Woody |
| 24 | Aphloiaceae | XI | 69.67 | 4.24 | 0 | 0 | 0 |  | Woody |
| 25 | Apiaceae | I | 58.00 | 4.06 | 1 | 0 | 1 |  | Mixed |
| 26 | Apocynaceae | II | 52.07 | 3.95 | 1 | 1 | 1 |  | Mixed |
| 27 | Apodanthaceae | XIV | 45.24 | 3.81 | 1 | 0 | 0 |  | Herbaceous |
| 28 | Aponogetonaceae | IV | 78.20 | 4.36 | 1 | 1 | 0 |  | Herbaceous |
| 29 | Aquifoliaceae | III | 64.17 | 4.16 | 1 | 0 | 0 |  | Woody |
| 30 | Araceae | I | 128.86 | 4.86 | 1 | 1 | 1 |  | Herbaceous |
| 31 | Araliaceae | II | 60.19 | 4.10 | 1 | 0 | 0 |  | Mixed |
| 32 | Araucariaceae | II.I | 85.29 | 4.45 | 0 | 0 | 0 |  | Woody |
| 33 | Arecaceae | II | 97.21 | 4.58 | 1 | 1 | 0 |  | Woody |
| 34 | Argophyllaceae | X | 64.92 | 4.17 | 0 | 0 | 0 | Long-distance dispersal is considered possible in the formation of this family, but is not assigned to a category by Maurin and Smissen (2022) | Woody |
| 35 | Aristolochiaceae | II | 105.44 | 4.66 | 1 | 1 | 1 |  | Mixed |
| 36 | Asparagaceae | I | 62.49 | 4.14 | 1 | 0 | 1 |  | Mixed |
| 37 | Asphodelaceae | I | 67.55 | 4.21 | 1 | 0 | 1 |  | Mixed |
| 38 | Asteliaceae | XVII | 64.50 | 4.17 | 1 | 0 | 1 |  | Mixed |
| 39 | Asteraceae | I | 49.34 | 3.90 | 1 | 1 | 1 |  | Mixed |
| 40 | Asteropeiaceae | XI.I | 42.06 | 3.74 | 0 | 0 | 0 |  | Woody |
| 41 | Atherospermataceae | XVII | 78.38 | 4.36 | 0 | 0 | 0 |  | Woody |
| 42 | Austrobaileyaceae | X | 122.61 | 4.81 | 1 | 0 | 0 |  | Woody |
| 43 | Balanopaceae | X | 81.54 | 4.40 | 1 | 0 | 0 |  | Woody |
| 44 | Balanophoraceae | II | 79.38 | 4.37 | 1 | 0 | 1 |  | Mixed |
| 45 | Balsaminaceae | VI | 50.46 | 3.92 | 0 | 1 | 0 |  | Mixed |
| 46 | Barbeuiaceae | XI.I | 77.76 | 4.35 | 0 | 0 | 0 |  | Mixed |
| 47 | Barbeyaceae | VI | 60.83 | 4.11 | 0 | 0 | 1 |  | Woody |
| 48 | Basellaceae | VII | 36.78 | 3.61 | 1 | 0 | 1 |  | Herbaceous |
| 49 | Bataceae | II.I | 32.64 | 3.49 | 0 | 1 | 0 |  | Woody |
| 50 | Begoniaceae | II | 45.42 | 3.82 | 1 | 0 | 1 |  | Mixed |
| 51 | Berberidaceae | XVIII | 80.28 | 4.39 | 1 | 1 | 1 |  | Mixed |
| 52 | Berberidopsidaceae | XVII | 27.13 | 3.30 | 0 | 0 | 0 |  | Woody |
| 53 | Betulaceae | XVIII | 78.45 | 4.36 | 1 | 0 | 1 |  | Woody |
| 54 | Biebersteiniaceae | XIV.I | 87.51 | 4.47 | 0 | 0 | 0 |  | Herbaceous |
| 55 | Bignoniaceae | II | 46.01 | 3.83 | 1 | 1 | 1 |  | Mixed |
| 56 | Bixaceae | II | 50.77 | 3.93 | 0 | 0 | 0 |  | Woody |
| 57 | Blandfordiaceae | XVII.II | 84.54 | 4.44 | 0 | 0 | 0 |  | Herbaceous |
| 58 | Bonnetiaceae | III | 88.38 | 4.48 | 0 | 0 | 0 |  | Woody |
| 59 | Boraginaceae | I | 88.25 | 4.48 | 1 | 1 | 1 |  | Mixed |
| 60 | Boryaceae | XVII.II | 93.32 | 4.54 | 0 | 0 | 0 |  | Woody |
| 61 | Brassicaceae | I | 44.22 | 3.79 | 1 | 1 | 1 |  | Mixed |
| 62 | Bromeliaceae | IX | 75.89 | 4.33 | 1 | 0 | 1 |  | Mixed |
| 63 | Brunelliaceae | IX | 75.58 | 4.33 | 0 | 0 | 0 |  | Woody |
| 64 | Bruniaceae | XVII.III | 78.27 | 4.36 | 0 | 0 | 0 |  | Woody |
| 65 | Burmanniaceae | II | 79.90 | 4.38 | 1 | 1 | 1 |  | Herbaceous |
| 66 | Burseraceae | II | 51.56 | 3.94 | 1 | 0 | 1 |  | Woody |
| 67 | Butomaceae | XII | 63.68 | 4.15 | 0 | 1 | 0 |  | Herbaceous |
| 68 | Buxaceae | XII | 128.55 | 4.86 | 1 | 1 | 0 |  | Mixed |
| 69 | Byblidaceae | X | 47.99 | 3.87 | 0 | 0 | 0 |  | Mixed |
| 70 | Cabombaceae | I | 109.57 | 4.70 | 0 | 1 | 0 |  | Herbaceous |
| 71 | Cactaceae | IX | 28.10 | 3.34 | 1 | 1 | 1 |  | Mixed |
| 72 | Calophyllaceae | II | 88.38 | 4.48 | 1 | 1 | 0 |  | Woody |
| 73 | Calycanthaceae | XIII | 114.90 | 4.74 | 0 | 0 | 0 |  | Woody |
| 74 | Calyceraceae | XVII.I | 49.34 | 3.90 | 1 | 1 | 1 |  | Herbaceous |
| 75 | Campanulaceae | I | 64.92 | 4.17 | 1 | 0 | 1 |  | Mixed |
| 76 | Campynemataceae | X | 84.27 | 4.43 | 0 | 0 | 0 | Long-distance dispersal is considered possible in the formation of this family, but is not assigned to a category by Givnish et al. (2016b) | Herbaceous |
| 77 | Canellaceae | VII | 126.00 | 4.84 | 1 | 0 | 0 |  | Woody |
| 78 | Cannabaceae | I | 73.47 | 4.30 | 1 | 1 | 0 |  | Mixed |
| 79 | Cannaceae | IX | 38.24 | 3.64 | 0 | 1 | 0 |  | Herbaceous |
| 80 | Capparaceae | II | 38.28 | 3.64 | 0 | 0 | 0 |  | Woody |
| 81 | Caprifoliaceae | XII | 70.94 | 4.26 | 1 | 1 | 1 |  | Mixed |
| 82 | Cardiopteridaceae | II | 74.14 | 4.31 | 1 | 0 | 1 |  | Mixed |
| 83 | Caricaceae | VII | 57.40 | 4.05 | 0 | 1 | 0 |  | Mixed |
| 84 | Carlemanniaceae | VIII | 40.79 | 3.71 | 1 | 0 | 0 |  | Mixed |
| 85 | Caryocaraceae | IX | 92.23 | 4.52 | 1 | 0 | 0 |  | Woody |
| 86 | Caryophyllaceae | I | 70.11 | 4.25 | 1 | 1 | 1 |  | Mixed |
| 87 | Casuarinaceae | V | 84.77 | 4.44 | 0 | 0 | 0 |  | Woody |
| 88 | Celastraceae | I | 92.61 | 4.53 | 1 | 1 | 1 |  | Mixed |
| 89 | Centroplacaceae | VI | 94.89 | 4.55 | 1 | 0 | 0 |  | Woody |
| 90 | Cephalotaceae | XVII.II | 75.58 | 4.33 | 0 | 0 | 0 |  | Herbaceous |
| 91 | Cephalotaxaceae | XV | - | - | 1 | 0 | 0 |  | Woody |
| 92 | Ceratophyllaceae | I | 134.42 | 4.90 | 1 | 1 | 0 |  | Herbaceous |
| 93 | Cercidiphyllaceae | XV | 60.49 | 4.10 | 0 | 0 | 1 |  | Woody |
| 94 | Chloranthaceae | II.I | 134.62 | 4.90 | 1 | 0 | 0 |  | Mixed |
| 95 | Chrysobalanaceae | II | 54.52 | 4.00 | 1 | 1 | 0 |  | Woody |
| 96 | Circaeasteraceae | XV | 86.33 | 4.46 | 0 | 0 | 0 |  | Herbaceous |
| 97 | Cistaceae | XIV | 35.62 | 3.57 | 0 | 0 | 0 |  | Mixed |
| 98 | Cleomaceae | II | 38.28 | 3.64 | 0 | 0 | 0 |  | Mixed |
| 99 | Clethraceae | III | 50.60 | 3.92 | 0 | 0 | 0 |  | Woody |
| 100 | Clusiaceae | II | 89.07 | 4.49 | 1 | 1 | 0 |  | Woody |
| 101 | Colchicaceae | I | 59.12 | 4.08 | 1 | 0 | 1 |  | Herbaceous |
| 102 | Columelliaceae | XVII.I | 78.27 | 4.36 | 0 | 0 | 0 |  | Woody |
| 103 | Combretaceae | II | 96.64 | 4.57 | 1 | 1 | 1 |  | Woody |
| 104 | Commelinaceae | II | 68.23 | 4.22 | 0 | 0 | 0 |  | Herbaceous |
| 105 | Connaraceae | II | 57.39 | 4.05 | 1 | 0 | 0 |  | Woody |
| 106 | Convolvulaceae | I | 66.65 | 4.20 | 0 | 0 | 0 |  | Mixed |
| 107 | Coriariaceae | XIV | 46.24 | 3.83 | 1 | 0 | 0 |  | Mixed |
| 108 | Cornaceae | XII | 84.52 | 4.44 | 1 | 0 | 0 |  | Mixed |
| 109 | Corsiaceae | II.I | 84.27 | 4.43 | 0 | 0 | 0 |  | Herbaceous |
| 110 | Corynocarpaceae | X | 45.24 | 3.81 | 1 | 0 | 0 |  | Woody |
| 111 | Costaceae | II | 46.13 | 3.83 | 1 | 1 | 0 |  | Herbaceous |
| 112 | Crassulaceae | I | 93.58 | 4.54 | 1 | 1 | 1 |  | Mixed |
| 113 | Crossosomataceae | XVI | 25.81 | 3.25 | 0 | 0 | 0 |  | Woody |
| 114 | Crypteroniaceae | VIII | 39.06 | 3.67 | 0 | 0 | 1 |  | Woody |
| 115 | Ctenolophonaceae | VI | 87.55 | 4.47 | 0 | 0 | 0 |  | Woody |
| 116 | Cucurbitaceae | II | 61.93 | 4.13 | 1 | 1 | 1 |  | Mixed |
| 117 | Cunoniaceae | II | 84.53 | 4.44 | 1 | 1 | 1 |  | Woody |
| 118 | Cupressaceae | XVIII | 67.82 | 4.22 | 0 | 0 | 0 |  | Woody |
| 119 | Curtisiaceae | XVII.III | 59.40 | 4.08 | 0 | 0 | 0 |  | Woody |
| 120 | Cycadaceae | V | 147.82 | 5.00 | 1 | 1 | 0 |  | Woody |
| 121 | Cyclanthaceae | IX | 44.91 | 3.80 | 1 | 1 | 0 |  | Mixed |
| 122 | Cymodoceaceae | II | 45.80 | 3.82 | 0 | 1 | 0 |  | Herbaceous |
| 123 | Cynomoriaceae | XIV.I | 105.05 | 4.65 | 0 | 0 | 0 |  | Herbaceous |
| 124 | Cyperaceae | I | 55.16 | 4.01 | 1 | 1 | 1 |  | Mixed |
| 125 | Cyrillaceae | IX | 50.60 | 3.92 | 0 | 0 | 0 |  | Woody |
| 126 | Cytinaceae | XIV | 38.58 | 3.65 | 0 | 0 | 0 |  | Herbaceous |
| 127 | Daphniphyllaceae | V | 60.49 | 4.10 | 0 | 0 | 0 |  | Woody |
| 128 | Dasypogonaceae | XVII.II | 97.21 | 4.58 | 0 | 0 | 0 |  | Mixed |
| 129 | Datiscaceae | XIV | 41.41 | 3.72 | 0 | 0 | 0 |  | Herbaceous |
| 130 | Degeneriaceae | X.II | 95.09 | 4.55 | 1 | 0 | 0 |  | Woody |
| 131 | Diapensiaceae | XII | 52.70 | 3.96 | 0 | 0 | 0 |  | Mixed |
| 132 | Dichapetalaceae | II | 51.90 | 3.95 | 0 | 0 | 0 |  | Woody |
| 133 | Didiereaceae | XI | 36.78 | 3.61 | 0 | 0 | 1 |  | Woody |
| 134 | Dilleniaceae | II.I | 122.58 | 4.81 | 1 | 1 | 0 |  | Mixed |
| 135 | Dioncophyllaceae | XI | 36.16 | 3.59 | 0 | 0 | 1 |  | Woody |
| 136 | Dioscoreaceae | II | 79.90 | 4.38 | 1 | 0 | 1 |  | Mixed |
| 137 | Dipentodontaceae | III | 35.93 | 3.58 | 0 | 0 | 0 |  | Woody |
| 138 | Dipterocarpaceae | VI | 20.16 | 3.00 | 0 | 1 | 1 |  | Woody |
| 139 | Dirachmaceae | XI | 60.83 | 4.11 | 0 | 0 | 0 |  | Woody |
| 140 | Doryanthaceae | XVII.II | 74.13 | 4.31 | 0 | 0 | 0 |  | Herbaceous |
| 141 | Droseraceae | I | 82.99 | 4.42 | 0 | 1 | 0 |  | Herbaceous |
| 142 | Drosophyllaceae | XIV.I | 57.93 | 4.06 | 0 | 0 | 0 | Long-distance dispersal is considered possible in the formation of this family, but is not assigned to a category by Martin-Rodriguez et al. (2020) | Mixed |
| 143 | Ebenaceae | II | 72.51 | 4.28 | 1 | 1 | 0 |  | Woody |
| 144 | Ecdeiocoleaceae | XVII.II | 54.41 | 4.00 | 0 | 0 | 0 |  | Herbaceous |
| 145 | Elaeagnaceae | XII | 65.22 | 4.18 | 1 | 1 | 0 |  | Woody |
| 146 | Elaeocarpaceae | II.I | 80.87 | 4.39 | 1 | 0 | 0 |  | Woody |
| 147 | Elatinaceae | I | 73.88 | 4.30 | 0 | 1 | 0 |  | Mixed |
| 148 | Emblingiaceae | XVII.II | 63.50 | 4.15 | 0 | 0 | 0 |  | Herbaceous |
| 149 | Ephedraceae | XVIII | 242.30 | 5.49 | 1 | 0 | 1 |  | Woody |
| 150 | Ericaceae | XVIII | 79.23 | 4.37 | 1 | 0 | 1 |  | Mixed |
| 151 | Eriocaulaceae | II | 77.13 | 4.35 | 0 | 1 | 1 |  | Herbaceous |
| 152 | Erythroxylaceae | II | 72.86 | 4.29 | 1 | 0 | 0 |  | Woody |
| 153 | Escalloniaceae | II.I | 92.73 | 4.53 | 0 | 0 | 0 |  | Mixed |
| 154 | Eucommiaceae | XV | 43.55 | 3.77 | 0 | 0 | 1 |  | Woody |
| 155 | Euphorbiaceae | II | 73.23 | 4.29 | 1 | 0 | 0 |  | Mixed |
| 156 | Euphroniaceae | IX | 54.52 | 4.00 | 0 | 0 | 0 |  | Woody |
| 157 | Eupomatiaceae | X | 94.22 | 4.55 | 1 | 0 | 0 |  | Woody |
| 158 | Eupteleaceae | XV | 114.83 | 4.74 | 0 | 0 | 1 |  | Woody |
| 159 | Fabaceae | I | 96.07 | 4.57 | 1 | 1 | 1 |  | Mixed |
| 160 | Fagaceae | XII | 97.88 | 4.58 | 1 | 0 | 0 |  | Woody |
| 161 | Flagellariaceae | IV | 74.69 | 4.31 | 0 | 0 | 0 |  | Herbaceous |
| 162 | Fouquieriaceae | XVI | 82.99 | 4.42 | 0 | 0 | 0 |  | Woody |
| 163 | Francoaceae | VII | 102.39 | 4.63 | 1 | 0 | 1 |  | Mixed |
| 164 | Frankeniaceae | XIV | 53.83 | 3.99 | 1 | 0 | 0 |  | Mixed |
| 165 | Garryaceae | XIII | 43.55 | 3.77 | 1 | 0 | 0 |  | Woody |
| 166 | Geissolomataceae | XVII.III | 51.63 | 3.94 | 0 | 0 | 0 |  | Woody |
| 167 | Gelsemiaceae | II.II | 49.33 | 3.90 | 0 | 0 | 1 |  | Woody |
| 168 | Gentianaceae | I | 52.07 | 3.95 | 1 | 1 | 1 |  | Mixed |
| 169 | Geraniaceae | XVIII | 102.39 | 4.63 | 1 | 0 | 1 |  | Mixed |
| 170 | Gerrardinaceae | XI | 57.27 | 4.05 | 0 | 0 | 0 |  | Woody |
| 171 | Gesneriaceae | II | 58.10 | 4.06 | 1 | 1 | 1 |  | Mixed |
| 172 | Ginkgoaceae | XV | 269.71 | 5.60 | 0 | 0 | 0 |  | Woody |
| 173 | Gisekiaceae | VI | 73.70 | 4.30 | 0 | 0 | 0 |  | Herbaceous |
| 174 | Gnetaceae | II.II | 239.31 | 5.48 | 1 | 1 | 0 |  | Woody |
| 175 | Gomortegaceae | XVII.I | 78.38 | 4.36 | 0 | 0 | 0 |  | Woody |
| 176 | Goodeniaceae | II | 57.05 | 4.04 | 1 | 0 | 1 |  | Mixed |
| 177 | Goupiaceae | IX | 83.07 | 4.42 | 0 | 0 | 0 |  | Woody |
| 178 | Griseliniaceae | XVII | 70.53 | 4.26 | 1 | 0 | 0 |  | Woody |
| 179 | Grossulariaceae | XVIII | 84.08 | 4.43 | 0 | 0 | 0 |  | Woody |
| 180 | Grubbiaceae | XVII.III | 59.40 | 4.08 | 0 | 0 | 0 |  | Woody |
| 181 | Guamatelaceae | IX | 25.81 | 3.25 | 0 | 0 | 0 |  | Woody |
| 182 | Gunneraceae | II | 104.64 | 4.65 | 0 | 0 | 0 |  | Herbaceous |
| 183 | Gyrostemonaceae | XVII.II | 49.68 | 3.91 | 0 | 0 | 1 |  | Mixed |
| 184 | Haemodoraceae | XVII | 44.33 | 3.79 | 0 | 0 | 0 |  | Herbaceous |
| 185 | Halophytaceae | XVII.I | 38.13 | 3.64 | 0 | 1 | 0 |  | Herbaceous |
| 186 | Haloragaceae | I | 70.74 | 4.26 | 1 | 1 | 1 |  | Mixed |
| 187 | Hamamelidaceae | XII | 94.12 | 4.54 | 0 | 1 | 1 |  | Woody |
| 188 | Hanguanaceae | V | 68.23 | 4.22 | 0 | 1 | 0 |  | Herbaceous |
| 189 | Heliconiaceae | IX | 50.29 | 3.92 | 1 | 0 | 0 |  | Herbaceous |
| 190 | Helwingiaceae | XV | 56.55 | 4.04 | 0 | 0 | 0 |  | Woody |
| 191 | Hernandiaceae | II | 105.09 | 4.65 | 1 | 1 | 1 |  | Woody |
| 192 | Himantandraceae | V | 95.09 | 4.55 | 1 | 0 | 0 |  | Woody |
| 193 | Huaceae | XI | 106.36 | 4.67 | 0 | 0 | 0 |  | Woody |
| 194 | Humiriaceae | IX | 103.51 | 4.64 | 0 | 1 | 0 |  | Woody |
| 195 | Hydatellaceae | XVII.II | 125.43 | 4.83 | 0 | 1 | 0 |  | Herbaceous |
| 196 | Hydrangeaceae | XIII | 90.52 | 4.51 | 1 | 0 | 1 |  | Mixed |
| 197 | Hydrocharitaceae | I | 63.68 | 4.15 | 1 | 1 | 0 |  | Herbaceous |
| 198 | Hydroleaceae | II | 56.55 | 4.04 | 0 | 1 | 0 |  | Mixed |
| 199 | Hydrostachyaceae | XI | 104.06 | 4.64 | 0 | 1 | 0 |  | Herbaceous |
| 200 | Hypericaceae | I | 67.82 | 4.22 | 1 | 0 | 1 |  | Mixed |
| 201 | Hypoxidaceae | II | 59.38 | 4.08 | 1 | 0 | 0 |  | Herbaceous |
| 202 | Icacinaceae | II | 68.35 | 4.22 | 1 | 0 | 0 |  | Woody |
| 203 | Iridaceae | I | 80.66 | 4.39 | 1 | 1 | 1 |  | Mixed |
| 204 | Irvingiaceae | VI | 93.61 | 4.54 | 1 | 1 | 1 |  | Woody |
| 205 | Iteaceae | XIII | 97.10 | 4.58 | 0 | 0 | 0 |  | Woody |
| 206 | Ixioliriaceae | XIV.I | 74.13 | 4.31 | 0 | 0 | 0 |  | Herbaceous |
| 207 | Ixonanthaceae | II.II | 89.70 | 4.50 | 0 | 0 | 0 |  | Woody |
| 208 | Joinvilleaceae | VIII | 54.41 | 4.00 | 0 | 0 | 0 |  | Herbaceous |
| 209 | Juglandaceae | XII | 90.65 | 4.51 | 1 | 0 | 1 |  | Woody |
| 210 | Juncaceae | XVIII | 55.16 | 4.01 | 1 | 1 | 1 |  | Herbaceous |
| 211 | Juncaginaceae | I | 68.76 | 4.23 | 1 | 1 | 0 |  | Herbaceous |
| 212 | Kewaceae | XVII.III | 77.77 | 4.35 | 0 | 0 | 0 |  | Mixed |
| 213 | Kirkiaceae | XI | 69.27 | 4.24 | 0 | 0 | 0 |  | Woody |
| 214 | Koeberliniaceae | IX | 57.82 | 4.06 | 0 | 0 | 0 |  | Woody |
| 215 | Krameriaceae | IX | 60.88 | 4.11 | 1 | 1 | 1 |  | Mixed |
| 216 | Lacistemataceae | IX | 74.11 | 4.31 | 0 | 0 | 0 |  | Woody |
| 217 | Lamiaceae | I | 40.29 | 3.70 | 1 | 1 | 1 |  | Mixed |
| 218 | Lanariaceae | XVII.III | 59.38 | 4.08 | 0 | 0 | 0 |  | Herbaceous |
| 219 | Lardizabalaceae | XVIII | 86.33 | 4.46 | 1 | 0 | 0 |  | Woody |
| 220 | Lauraceae | II | 104.88 | 4.65 | 1 | 1 | 0 |  | Woody |
| 221 | Lecythidaceae | II | 72.51 | 4.28 | 0 | 0 | 0 |  | Woody |
| 222 | Lentibulariaceae | I | 43.42 | 3.77 | 0 | 1 | 1 |  | Herbaceous |
| 223 | Lepidobotryaceae | VII | 92.61 | 4.53 | 0 | 0 | 0 |  | Woody |
| 224 | Liliaceae | XII | 55.58 | 4.02 | 1 | 0 | 1 |  | Herbaceous |
| 225 | Limeaceae | VI | 85.04 | 4.44 | 0 | 0 | 0 |  | Mixed |
| 226 | Limnanthaceae | XVI | 73.31 | 4.29 | 0 | 0 | 0 |  | Herbaceous |
| 227 | Linaceae | I | 89.70 | 4.50 | 0 | 0 | 0 |  | Mixed |
| 228 | Linderniaceae | II | 48.40 | 3.88 | 0 | 0 | 0 |  | Mixed |
| 229 | Loasaceae | IX | 90.52 | 4.51 | 0 | 0 | 0 |  | Mixed |
| 230 | Loganiaceae | II | 49.33 | 3.90 | 1 | 0 | 1 |  | Mixed |
| 231 | Lophiocarpaceae | VI | 79.56 | 4.38 | 0 | 0 | 0 |  | Mixed |
| 232 | Lophopyxidaceae | VIII | 59.01 | 4.08 | 0 | 0 | 0 |  | Woody |
| 233 | Loranthaceae | II | 67.87 | 4.22 | 1 | 0 | 1 |  | Woody |
| 234 | Lowiaceae | VIII | 47.14 | 3.85 | 0 | 0 | 0 |  | Herbaceous |
| 235 | Lythraceae | I | 72.64 | 4.29 | 0 | 1 | 0 |  | Mixed |
| 236 | Macarthuriaceae | XVII.II | 80.69 | 4.39 | 0 | 0 | 0 |  | Mixed |
| 237 | Magnoliaceae | XIII | 100.66 | 4.61 | 0 | 0 | 0 |  | Woody |
| 238 | Malpighiaceae | II | 73.88 | 4.30 | 0 | 0 | 0 |  | Mixed |
| 239 | Malvaceae | II | 61.44 | 4.12 | 1 | 1 | 1 |  | Mixed |
| 240 | Marantaceae | II | 38.24 | 3.64 | 1 | 1 | 0 |  | Herbaceous |
| 241 | Marcgraviaceae | IX | 55.44 | 4.02 | 1 | 0 | 0 |  | Woody |
| 242 | Martyniaceae | IX | 21.37 | 3.06 | 1 | 0 | 0 |  | Herbaceous |
| 243 | Maundiaceae | XVII.II | 62.54 | 4.14 | 0 | 1 | 0 |  | Herbaceous |
| 244 | Mayacaceae | IX | 77.17 | 4.35 | 0 | 1 | 0 |  | Herbaceous |
| 245 | Mazaceae | XVIII | 37.38 | 3.62 | 0 | 0 | 0 |  | Herbaceous |
| 246 | Melanthiaceae | XII | 84.19 | 4.43 | 1 | 0 | 1 |  | Herbaceous |
| 247 | Melastomataceae | II | 72.80 | 4.29 | 0 | 1 | 0 |  | Mixed |
| 248 | Meliaceae | II | 66.89 | 4.20 | 1 | 1 | 1 |  | Mixed |
| 249 | Menispermaceae | II | 89.93 | 4.50 | 1 | 0 | 0 |  | Mixed |
| 250 | Menyanthaceae | I | 68.27 | 4.22 | 1 | 1 | 0 |  | Herbaceous |
| 251 | Metteniusaceae | II | 101.55 | 4.62 | 0 | 0 | 0 |  | Woody |
| 252 | Microteaceae | IX | 90.44 | 4.50 | 0 | 0 | 0 |  | Herbaceous |
| 253 | Misodendraceae | XVII.I | 58.01 | 4.06 | 0 | 0 | 1 |  | Woody |
| 254 | Mitrastemonaceae | III | 102.13 | 4.63 | 0 | 0 | 0 |  | Herbaceous |
| 255 | Molluginaceae | II | 73.11 | 4.29 | 0 | 0 | 0 |  | Mixed |
| 256 | Monimiaceae | II | 104.88 | 4.65 | 1 | 1 | 1 |  | Woody |
| 257 | Montiaceae | XVIII | 42.80 | 3.76 | 0 | 0 | 0 |  | Mixed |
| 258 | Montiniaceae | XI | 72.04 | 4.28 | 0 | 0 | 0 |  | Woody |
| 259 | Moraceae | I | 68.51 | 4.23 | 1 | 1 | 0 |  | Mixed |
| 260 | Moringaceae | VI | 57.40 | 4.05 | 0 | 1 | 1 |  | Woody |
| 261 | Muntingiaceae | IX | 38.58 | 3.65 | 0 | 0 | 0 |  | Woody |
| 262 | Musaceae | IV | 58.26 | 4.06 | 1 | 0 | 0 |  | Herbaceous |
| 263 | Myodocarpaceae | V | 58.00 | 4.06 | 1 | 0 | 1 |  | Woody |
| 264 | Myricaceae | I | 89.76 | 4.50 | 1 | 1 | 0 |  | Woody |
| 265 | Myristicaceae | II | 109.59 | 4.70 | 1 | 1 | 0 |  | Woody |
| 266 | Myrothamnaceae | XI | 104.64 | 4.65 | 0 | 0 | 1 |  | Woody |
| 267 | Myrtaceae | II | 85.64 | 4.45 | 1 | 1 | 0 |  | Woody |
| 268 | Nartheciaceae | XII | 95.23 | 4.56 | 0 | 0 | 0 |  | Herbaceous |
| 269 | Nelumbonaceae | XIII | 127.50 | 4.85 | 0 | 1 | 0 |  | Herbaceous |
| 270 | Nepenthaceae | V | 78.32 | 4.36 | 0 | 0 | 0 |  | Mixed |
| 271 | Neuradaceae | XIV | 76.30 | 4.33 | 0 | 0 | 0 |  | Herbaceous |
| 272 | Nitrariaceae | XIV | 83.35 | 4.42 | 1 | 0 | 1 |  | Mixed |
| 273 | Nothofagaceae | XVII | 102.82 | 4.63 | 0 | 0 | 1 |  | Woody |
| 274 | Nyctaginaceae | II | 71.44 | 4.27 | 1 | 1 | 1 |  | Mixed |
| 275 | Nymphaeaceae | I | 109.57 | 4.70 | 1 | 1 | 0 |  | Herbaceous |
| 276 | Nyssaceae | XIII | 92.60 | 4.53 | 1 | 1 | 0 |  | Woody |
| 277 | Ochnaceae | II.II | 92.23 | 4.52 | 1 | 1 | 1 |  | Mixed |
| 278 | Olacaceae | II | 103.58 | 4.64 | 1 | 0 | 0 |  | Woody |
| 279 | Oleaceae | I | 40.79 | 3.71 | 1 | 0 | 1 |  | Woody |
| 280 | Onagraceae | I | 72.64 | 4.29 | 0 | 0 | 0 |  | Mixed |
| 281 | Oncothecaceae | X.I | 68.35 | 4.22 | 0 | 0 | 0 |  | Woody |
| 282 | Opiliaceae | II | 80.62 | 4.39 | 0 | 0 | 0 |  | Woody |
| 283 | Orchidaceae | I | 108.96 | 4.69 | 0 | 0 | 0 | Long-distance dispersal is considered possible in the formation of this family, but is not assigned to a category by Givnish et al. (2016a) | Herbaceous |
| 284 | Orobanchaceae | I | 36.04 | 3.58 | 0 | 0 | 1 |  | Mixed |
| 285 | Oxalidaceae | I | 57.39 | 4.05 | 1 | 0 | 0 |  | Mixed |
| 286 | Paeoniaceae | XII | 104.84 | 4.65 | 1 | 0 | 0 |  | Mixed |
| 287 | Pandaceae | VI | 91.69 | 4.52 | 0 | 0 | 0 |  | Woody |
| 288 | Pandanaceae | IV | 44.91 | 3.80 | 0 | 1 | 0 |  | Woody |
| 289 | Papaveraceae | XVIII | 112.94 | 4.73 | 1 | 0 | 1 |  | Mixed |
| 290 | Paracryphiaceae | V | 81.85 | 4.40 | 1 | 0 | 0 |  | Woody |
| 291 | Passifloraceae | II | 91.13 | 4.51 | 1 | 0 | 1 |  | Mixed |
| 292 | Paulowniaceae | XV | 36.04 | 3.58 | 0 | 0 | 0 |  | Woody |
| 293 | Pedaliaceae | IV | 46.01 | 3.83 | 1 | 0 | 1 |  | Mixed |
| 294 | Penaeaceae | XVII.III | 30.89 | 3.43 | 1 | 0 | 1 |  | Woody |
| 295 | Pennantiaceae | XVII.II | 80.81 | 4.39 | 1 | 0 | 0 |  | Woody |
| 296 | Pentadiplandraceae | XI | 55.38 | 4.01 | 1 | 0 | 0 |  | Woody |
| 297 | Pentaphragmataceae | VIII | 78.11 | 4.36 | 0 | 0 | 0 |  | Herbaceous |
| 298 | Pentaphylacaceae | II | 75.90 | 4.33 | 0 | 0 | 0 |  | Woody |
| 299 | Penthoraceae | XIII | 70.74 | 4.26 | 0 | 1 | 1 |  | Herbaceous |
| 300 | Peraceae | II.II | 74.23 | 4.31 | 1 | 0 | 0 |  | Mixed |
| 301 | Peridiscaceae | VII | 121.41 | 4.80 | 0 | 0 | 0 |  | Woody |
| 302 | Petenaeaceae | IX | 57.27 | 4.05 | 0 | 0 | 0 |  | Woody |
| 303 | Petermanniaceae | XVII.II | 80.49 | 4.39 | 0 | 0 | 0 |  | Woody |
| 304 | Petiveriaceae | IX | 58.90 | 4.08 | 1 | 0 | 1 |  | Mixed |
| 305 | Petrosaviaceae | VIII | 123.52 | 4.82 | 0 | 0 | 0 |  | Herbaceous |
| 306 | Phellinaceae | X.I | 31.43 | 3.45 | 1 | 0 | 0 |  | Woody |
| 307 | Philesiaceae | XVII.I | 43.22 | 3.77 | 0 | 0 | 0 |  | Woody |
| 308 | Philydraceae | V | 62.33 | 4.13 | 0 | 1 | 1 |  | Herbaceous |
| 309 | Phrymaceae | I | 36.49 | 3.60 | 1 | 1 | 0 |  | Mixed |
| 310 | Phyllanthaceae | II | 83.78 | 4.43 | 0 | 0 | 0 |  | Mixed |
| 311 | Phyllonomaceae | IX | 56.55 | 4.04 | 1 | 0 | 0 |  | Woody |
| 312 | Physenaceae | XI.I | 42.06 | 3.74 | 0 | 0 | 0 |  | Woody |
| 313 | Phytolaccaceae | II.II | 70.33 | 4.25 | 0 | 0 | 0 |  | Mixed |
| 314 | Picramniaceae | IX | 109.94 | 4.70 | 0 | 0 | 0 |  | Woody |
| 315 | Picrodendraceae | II | 83.78 | 4.43 | 0 | 0 | 0 |  | Woody |
| 316 | Pinaceae | XII | 126.82 | 4.84 | 0 | 0 | 0 |  | Woody |
| 317 | Piperaceae | II | 65.52 | 4.18 | 1 | 0 | 0 |  | Mixed |
| 318 | Pittosporaceae | IV | 63.37 | 4.15 | 1 | 0 | 1 |  | Woody |
| 319 | Plantaginaceae | I | 52.93 | 3.97 | 1 | 1 | 0 |  | Mixed |
| 320 | Platanaceae | XVI | 106.22 | 4.67 | 0 | 0 | 0 |  | Woody |
| 321 | Plocospermataceae | IX | 77.05 | 4.34 | 0 | 0 | 0 |  | Woody |
| 322 | Plumbaginaceae | I | 67.90 | 4.22 | 0 | 1 | 1 |  | Mixed |
| 323 | Poaceae | I | 58.58 | 4.07 | 1 | 1 | 1 |  | Mixed |
| 324 | Podocarpaceae | II | 85.29 | 4.45 | 1 | 1 | 0 |  | Woody |
| 325 | Podostemaceae | II | 67.82 | 4.22 | 0 | 1 | 0 |  | Herbaceous |
| 326 | Polemoniaceae | XVIII | 82.99 | 4.42 | 0 | 0 | 0 |  | Mixed |
| 327 | Polygalaceae | I | 93.27 | 4.54 | 1 | 0 | 1 |  | Mixed |
| 328 | Polygonaceae | I | 67.90 | 4.22 | 0 | 1 | 1 |  | Mixed |
| 329 | Pontederiaceae | II | 44.33 | 3.79 | 0 | 1 | 0 |  | Herbaceous |
| 330 | Portulacaceae | I | 28.80 | 3.36 | 0 | 0 | 0 |  | Herbaceous |
| 331 | Posidoniaceae | XIV | 47.37 | 3.86 | 0 | 1 | 0 |  | Herbaceous |
| 332 | Potamogetonaceae | I | 53.23 | 3.97 | 1 | 1 | 0 |  | Herbaceous |
| 333 | Primulaceae | I | 86.95 | 4.47 | 1 | 1 | 0 |  | Mixed |
| 334 | Proteaceae | II | 106.22 | 4.67 | 1 | 0 | 1 |  | Woody |
| 335 | Putranjivaceae | II | 59.01 | 4.08 | 1 | 0 | 0 |  | Woody |
| 336 | Quillajaceae | XVII.I | 98.70 | 4.59 | 0 | 0 | 0 |  | Woody |
| 337 | Rafflesiaceae | VIII | 73.23 | 4.29 | 1 | 0 | 0 |  | Herbaceous |
| 338 | Ranunculaceae | I | 80.28 | 4.39 | 1 | 1 | 0 |  | Mixed |
| 339 | Rapateaceae | IX | 97.55 | 4.58 | 0 | 1 | 0 |  | Herbaceous |
| 340 | Resedaceae | XII | 49.68 | 3.91 | 1 | 0 | 0 |  | Mixed |
| 341 | Restionaceae | XVII | 80.80 | 4.39 | 0 | 0 | 0 |  | Herbaceous |
| 342 | Rhabdodendraceae | IX | 103.95 | 4.64 | 0 | 0 | 0 |  | Woody |
| 343 | Rhamnaceae | I | 75.61 | 4.33 | 1 | 1 | 1 |  | Mixed |
| 344 | Rhizophoraceae | II | 72.86 | 4.29 | 1 | 1 | 1 |  | Woody |
| 345 | Ripogonaceae | X | 43.40 | 3.77 | 0 | 0 | 0 |  | Woody |
| 346 | Roridulaceae | XVII.III | 84.81 | 4.44 | 0 | 0 | 0 |  | Woody |
| 347 | Rosaceae | I | 98.96 | 4.59 | 1 | 0 | 1 |  | Mixed |
| 348 | Rousseaceae | X | 76.05 | 4.33 | 1 | 0 | 0 |  | Woody |
| 349 | Rubiaceae | I | 67.73 | 4.22 | 1 | 1 | 1 |  | Mixed |
| 350 | Ruppiaceae | I | 45.80 | 3.82 | 0 | 1 | 0 |  | Herbaceous |
| 351 | Rutaceae | II | 69.27 | 4.24 | 1 | 1 | 0 |  | Mixed |
| 352 | Sabiaceae | III | 112.88 | 4.73 | 1 | 0 | 0 |  | Woody |
| 353 | Salicaceae | I | 74.11 | 4.31 | 0 | 0 | 1 |  | Woody |
| 354 | Salvadoraceae | VI | 32.64 | 3.49 | 0 | 0 | 0 |  | Woody |
| 355 | Santalaceae | I | 79.38 | 4.37 | 1 | 0 | 0 |  | Mixed |
| 356 | Sapindaceae | II | 79.15 | 4.37 | 1 | 0 | 1 |  | Mixed |
| 357 | Sapotaceae | II | 61.91 | 4.13 | 1 | 0 | 0 |  | Woody |
| 358 | Sarcobataceae | XVI | 58.90 | 4.08 | 0 | 0 | 0 |  | Woody |
| 359 | Sarcolaenaceae | XI.I | 20.16 | 3.00 | 0 | 0 | 0 |  | Woody |
| 360 | Sarraceniaceae | XVI | 88.09 | 4.48 | 0 | 1 | 0 |  | Herbaceous |
| 361 | Saururaceae | XIII | 65.52 | 4.18 | 0 | 0 | 0 |  | Herbaceous |
| 362 | Saxifragaceae | XVIII | 84.08 | 4.43 | 1 | 1 | 1 |  | Herbaceous |
| 363 | Scheuchzeriaceae | XII | 79.59 | 4.38 | 0 | 0 | 0 |  | Herbaceous |
| 364 | Schisandraceae | XIII | 109.73 | 4.70 | 1 | 1 | 0 |  | Woody |
| 365 | Schlegeliaceae | IX | 21.37 | 3.06 | 0 | 0 | 0 |  | Woody |
| 366 | Schoepfiaceae | III | 58.01 | 4.06 | 0 | 0 | 0 |  | Mixed |
| 367 | Sciadopityaceae | XV.I | 90.53 | 4.51 | 1 | 0 | 0 |  | Woody |
| 368 | Scrophulariaceae | I | 47.99 | 3.87 | 1 | 1 | 1 |  | Mixed |
| 369 | Setchellanthaceae | XVI | 72.31 | 4.28 | 0 | 0 | 0 |  | Woody |
| 370 | Simaroubaceae | II | 66.89 | 4.20 | 1 | 1 | 1 |  | Woody |
| 371 | Simmondsiaceae | XVI | 100.87 | 4.61 | 1 | 0 | 0 |  | Woody |
| 372 | Siparunaceae | VII | 91.80 | 4.52 | 0 | 0 | 0 |  | Woody |
| 373 | Sladeniaceae | VI | 75.90 | 4.33 | 0 | 0 | 0 |  | Woody |
| 374 | Smilacaceae | II | 43.22 | 3.77 | 1 | 0 | 0 |  | Mixed |
| 375 | Solanaceae | I | 66.65 | 4.20 | 1 | 1 | 1 |  | Mixed |
| 376 | Sphaerosepalaceae | XI.I | 50.77 | 3.93 | 0 | 0 | 0 |  | Woody |
| 377 | Sphenocleaceae | IV | 56.55 | 4.04 | 0 | 0 | 0 |  | Herbaceous |
| 378 | Stachyuraceae | XV | 25.99 | 3.26 | 0 | 0 | 0 |  | Woody |
| 379 | Staphyleaceae | III | 31.29 | 3.44 | 1 | 1 | 1 |  | Woody |
| 380 | Stegnospermataceae | XVI | 89.49 | 4.49 | 0 | 0 | 0 |  | Woody |
| 381 | Stemonaceae | V | 51.47 | 3.94 | 1 | 0 | 0 |  | Mixed |
| 382 | Stemonuraceae | II | 74.14 | 4.31 | 1 | 1 | 0 |  | Woody |
| 383 | Stilbaceae | XVII.III | 48.02 | 3.87 | 0 | 0 | 0 |  | Mixed |
| 384 | Strasburgeriaceae | X | 51.63 | 3.94 | 1 | 0 | 0 |  | Woody |
| 385 | Strelitziaceae | VII | 47.14 | 3.85 | 1 | 0 | 0 |  | Mixed |
| 386 | Stylidiaceae | XVII | 31.43 | 3.45 | 1 | 0 | 0 |  | Mixed |
| 387 | Styracaceae | III | 52.70 | 3.96 | 1 | 1 | 1 |  | Woody |
| 388 | Surianaceae | II | 93.27 | 4.54 | 0 | 1 | 0 |  | Woody |
| 389 | Symplocaceae | II.I | 81.19 | 4.40 | 1 | 1 | 0 |  | Woody |
| 390 | Talinaceae | VII | 33.32 | 3.51 | 0 | 0 | 0 |  | Mixed |
| 391 | Tamaricaceae | XII | 53.83 | 3.99 | 0 | 0 | 1 |  | Woody |
| 392 | Tapisciaceae | III | 35.93 | 3.58 | 0 | 0 | 0 |  | Woody |
| 393 | Taxaceae | XII | 67.82 | 4.22 | 1 | 0 | 0 |  | Woody |
| 394 | Tecophilaeaceae | XIV | 88.95 | 4.49 | 0 | 0 | 0 |  | Herbaceous |
| 395 | Tetracarpaeaceae | XVII.II | 78.75 | 4.37 | 0 | 0 | 0 |  | Woody |
| 396 | Tetrachondraceae | XVIII | 66.71 | 4.20 | 0 | 1 | 0 |  | Herbaceous |
| 397 | Tetramelaceae | V | 41.41 | 3.72 | 0 | 0 | 0 |  | Woody |
| 398 | Tetrameristaceae | III | 50.46 | 3.92 | 0 | 0 | 0 |  | Woody |
| 399 | Theaceae | III | 61.91 | 4.13 | 0 | 0 | 1 |  | Woody |
| 400 | Thomandersiaceae | XI | 41.89 | 3.74 | 0 | 0 | 0 |  | Woody |
| 401 | Thurniaceae | VII | 68.78 | 4.23 | 0 | 0 | 0 |  | Herbaceous |
| 402 | Thymelaeaceae | I | 70.12 | 4.25 | 0 | 0 | 0 |  | Mixed |
| 403 | Ticodendraceae | IX | 78.45 | 4.36 | 0 | 0 | 0 |  | Woody |
| 404 | Tiganophytaceae | XVII | - | - | 0 | 0 | 0 |  | Woody |
| 405 | Tofieldiaceae | XII | 126.55 | 4.84 | 0 | 0 | 0 |  | Herbaceous |
| 406 | Torricelliaceae | VI | 75.52 | 4.32 | 0 | 0 | 0 |  | Woody |
| 407 | Tovariaceae | IX | 59.47 | 4.09 | 0 | 0 | 0 |  | Mixed |
| 408 | Trigoniaceae | IX | 51.90 | 3.95 | 0 | 1 | 1 |  | Woody |
| 409 | Trimeniaceae | V | 109.73 | 4.70 | 1 | 0 | 0 |  | Woody |
| 410 | Triuridaceae | II.II | 72.30 | 4.28 | 1 | 1 | 1 |  | Herbaceous |
| 411 | Trochodendraceae | XV | 129.00 | 4.86 | 0 | 0 | 1 |  | Woody |
| 412 | Tropaeolaceae | XVII.I | 71.11 | 4.26 | 0 | 0 | 0 |  | Herbaceous |
| 413 | Typhaceae | I | 75.89 | 4.33 | 0 | 1 | 1 |  | Herbaceous |
| 414 | Ulmaceae | I | 79.17 | 4.37 | 0 | 1 | 1 |  | Woody |
| 415 | Urticaceae | II | 68.51 | 4.23 | 1 | 1 | 1 |  | Mixed |
| 416 | Vahliaceae | VI | 92.68 | 4.53 | 0 | 0 | 0 |  | Herbaceous |
| 417 | Velloziaceae | VII | 57.78 | 4.06 | 0 | 0 | 0 |  | Herbaceous |
| 418 | Verbenaceae | I | 47.16 | 3.85 | 1 | 0 | 1 |  | Mixed |
| 419 | Violaceae | I | 83.07 | 4.42 | 1 | 1 | 1 |  | Mixed |
| 420 | Vitaceae | II | 121.29 | 4.80 | 1 | 1 | 0 |  | Mixed |
| 421 | Vochysiaceae | IX | 85.64 | 4.45 | 0 | 1 | 1 |  | Mixed |
| 422 | Welwitschiaceae | XI | 239.31 | 5.48 | 0 | 0 | 1 |  | Woody |
| 423 | Winteraceae | II.I | 126.00 | 4.84 | 0 | 0 | 0 |  | Woody |
| 424 | Xeronemataceae | X | 73.72 | 4.30 | 0 | 0 | 0 |  | Herbaceous |
| 425 | Xyridaceae | II | 77.13 | 4.35 | 0 | 0 | 0 |  | Herbaceous |
| 426 | Zamiaceae | II | 147.82 | 5.00 | 1 | 0 | 0 |  | Woody |
| 427 | Zingiberaceae | V | 46.13 | 3.83 | 1 | 1 | 0 |  | Herbaceous |
| 428 | Zosteraceae | XVIII | 53.23 | 3.97 | 0 | 1 | 0 |  | Herbaceous |
| 429 | Zygophyllaceae | I | 60.88 | 4.11 | 1 | 0 | 1 |  | Mixed |

**Table S4. Formation factors of the 89 spermatophyte families**

| **Number** | **Family** | **Distribution type (including distribution subtype)** | **Climate** | **Land bridges** | **Tectonic movements** | **long‐distance dispersal** | **Reference** |
| --- | --- | --- | --- | --- | --- | --- | --- |
| 1 | Alismataceae | I | Unmentioned | Mentioned | Mentioned | Mentioned | (Chen et al., 2012a; Li et al., 2022) |
| 2 | Alstroemeriaceae | XVII | Mentioned | Unmentioned | Mentioned | Mentioned | (Chacón et al., 2012) |
| 3 | Altingiaceae | XIII | Mentioned | Mentioned | Unmentioned | Unmentioned | (Lai et al., 2021) |
| 4 | Anisophylleaceae | II.II | Unmentioned | Unmentioned | Mentioned | Mentioned | (Zhang et al., 2007) |
| 5 | Annonaceae | II | Mentioned | Mentioned | Mentioned | Mentioned | (Onstein et al., 2019; Thomas et al., 2015) |
| 6 | Apocynaceae | II | Mentioned | Mentioned | Unmentioned | Mentioned | (Bitencourt et al., 2021) |
| 7 | Aponogetonaceae | IV | Unmentioned | Unmentioned | Unmentioned | Mentioned | (Chen et al., 2015) |
| 8 | Araceae | I | Mentioned | Unmentioned | Mentioned | Mentioned | (Nauheimer et al., 2012) |
| 9 | Argophyllaceae | X | Unmentioned | Unmentioned | Unmentioned | Mentioned | (Maurin and Smissen, 2022) |
| 10 | Asteliaceae | XVII | Unmentioned | Unmentioned | Mentioned | Mentioned | (Birch and Keeley, 2013; Birch and Kocyan, 2021) |
| 11 | Balsaminaceae | VI | Mentioned | Mentioned | Mentioned | Unmentioned | (Janssens et al., 2009) |
| 12 | Betulaceae | XVIII | Mentioned | Mentioned | Unmentioned | Unmentioned | (Chen et al., 1999) |
| 13 | Boraginaceae | I | Unmentioned | Unmentioned | Mentioned | Mentioned | (Luebert et al., 2017) |
| 14 | Brassicaceae | I | Mentioned | Unmentioned | Unmentioned | Mentioned | (Franzke et al., 2009) |
| 15 | Bromeliaceae | IX | Mentioned | Unmentioned | Mentioned | Mentioned | (Givnish et al., 2011) |
| 16 | Burmanniaceae | II | Mentioned | Mentioned | Mentioned | Unmentioned | (Merckx et al., 2008) |
| 17 | Campanulaceae | I | Mentioned | Mentioned | Mentioned | Mentioned | (Crowl et al., 2016) |
| 18 | Campynemataceae | X | Unmentioned | Unmentioned | Unmentioned | Mentioned | (Givnish et al., 2016b) |
| 19 | Canellaceae | VII | Mentioned | Unmentioned | Mentioned | Mentioned | (Müller et al., 2015) |
| 20 | Cannabaceae | I | Mentioned | Mentioned | Mentioned | Mentioned | (Jin et al., 2020) |
| 21 | Caricaceae | VII | Mentioned | Mentioned | Mentioned | Mentioned | (Carvalho and Renner, 2012) |
| 22 | Centroplacaceae | VI | Mentioned | Unmentioned | Unmentioned | Unmentioned | (Cai et al., 2016) |
| 23 | Cercidiphyllaceae | XV | Mentioned | Unmentioned | Unmentioned | Unmentioned | (Qi et al., 2012) |
| 24 | Chrysobalanaceae | II | Mentioned | Unmentioned | Unmentioned | Mentioned | (Bardon et al., 2016) |
| 25 | Colchicaceae | I | Mentioned | Mentioned | Mentioned | Mentioned | (Chacón and Renner, 2014) |
| 26 | Coriariaceae | XIV | Mentioned | Mentioned | Mentioned | Unmentioned | (Renner et al., 2020) |
| 27 | Corsiaceae | II.I | Unmentioned | Unmentioned | Mentioned | Unmentioned | (Mennes et al., 2015) |
| 28 | Corynocarpaceae | X | Unmentioned | Mentioned | Unmentioned | Unmentioned | (Wagstaff and Dawson, 2000) |
| 29 | Costaceae | II | Unmentioned | Mentioned | Mentioned | Mentioned | (Specht, 2006) |
| 30 | Crossosomataceae | XVI | Mentioned | Unmentioned | Unmentioned | Unmentioned | (Zhu et al., 2006) |
| 31 | Crypteroniaceae | VIII | Mentioned | Unmentioned | Mentioned | Unmentioned | (Rutschmann et al., 2004) |
| 32 | Cucurbitaceae | II | Unmentioned | Unmentioned | Mentioned | Mentioned | (Schaefer et al., 2009) |
| 33 | Cunoniaceae | II | Unmentioned | Mentioned | Mentioned | Mentioned | (Pillon et al., 2021) |
| 34 | Cupressaceae | XVIII | Mentioned | Unmentioned | Mentioned | Unmentioned | (Mao et al., 2012) |
| 35 | Cycadaceae | V | Mentioned | Mentioned | Mentioned | Mentioned | (Coiro et al., 2023; Liu et al., 2022; Liu et al., 2021) |
| 36 | Cyperaceae | I | Unmentioned | Unmentioned | Mentioned | Mentioned | (Spalink et al., 2016) |
| 37 | Diapensiaceae | XII | Mentioned | Mentioned | Mentioned | Unmentioned | (Gaynor et al., 2020) |
| 38 | Dipterocarpaceae | VI | Mentioned | Unmentioned | Mentioned | Mentioned | (Bansal et al., 2022) |
| 39 | Drosophyllaceae | XIV.I | Unmentioned | Unmentioned | Unmentioned | Mentioned | (Martin-Rodriguez et al., 2020) |
| 40 | Elaeocarpaceae | II.I | Unmentioned | Mentioned | Mentioned | Unmentioned | (Pillon et al., 2021) |
| 41 | Elatinaceae | I | Unmentioned | Mentioned | Mentioned | Mentioned | (Cai et al., 2016) |
| 42 | Ephedraceae | XVIII | Unmentioned | Mentioned | Unmentioned | Mentioned | (Ickert-Bond et al., 2009) |
| 43 | Eupteleaceae | XV | Mentioned | Mentioned | Mentioned | Unmentioned | (Cao et al., 2016) |
| 44 | Gesneriaceae | II | Unmentioned | Unmentioned | Mentioned | Unmentioned | (Roalson and Roberts, 2016) |
| 45 | Haloragaceae | I | Unmentioned | Mentioned | Mentioned | Mentioned | (Chen et al., 2014) |
| 46 | Humiriaceae | IX | Mentioned | Unmentioned | Mentioned | Unmentioned | (Herrera et al., 2014) |
| 47 | Hydatellaceae | XVII.II | Mentioned | Unmentioned | Unmentioned | Mentioned | (Iles et al., 2014) |
| 48 | Hydrocharitaceae | I | Unmentioned | Mentioned | Mentioned | Mentioned | (Chen et al., 2012b) |
| 49 | Hypoxidaceae | II | Mentioned | Unmentioned | Mentioned | Mentioned | (Birch and Kocyan, 2021) |
| 50 | Juglandaceae | XII | Mentioned | Mentioned | Mentioned | Unmentioned | (Zhang et al., 2022b) |
| 51 | Lardizabalaceae | XVIII | Mentioned | Unmentioned | Mentioned | Mentioned | (Wang et al., 2020) |
| 52 | Liliaceae | XII | Unmentioned | Mentioned | Mentioned | Unmentioned | (Vinnersten and Bremer, 2001) |
| 53 | Loranthaceae | II | Mentioned | Mentioned | Mentioned | Mentioned | (Liu et al., 2018) |
| 54 | Magnoliaceae | XIII | Mentioned | Mentioned | Unmentioned | Unmentioned | (Nie et al., 2008) |
| 55 | Malpighiaceae | II | Mentioned | Mentioned | Mentioned | Unmentioned | (Cai et al., 2016; Davis et al., 2002) |
| 56 | Marantaceae | II | Mentioned | Unmentioned | Mentioned | Mentioned | (Prince and Kress, 2006) |
| 57 | Mazaceae | XVIII | Unmentioned | Mentioned | Mentioned | Unmentioned | (Deng, 2015) |
| 58 | Melanthiaceae | XII | Unmentioned | Mentioned | Unmentioned | Unmentioned | (Givnish et al., 2016b) |
| 59 | Menispermaceae | II | Unmentioned | Unmentioned | Mentioned | Unmentioned | (Wang et al., 2012) |
| 60 | Monimiaceae | II | Mentioned | Mentioned | Mentioned | Mentioned | (Renner et al., 2010) |
| 61 | Musaceae | IV | Mentioned | Mentioned | Mentioned | Mentioned | (Janssens et al., 2016) |
| 62 | Myricaceae | I | Mentioned | Mentioned | Mentioned | Unmentioned | (Herbert, 2004) |
| 63 | Myristicaceae | II | Mentioned | Mentioned | Mentioned | Unmentioned | (Frost et al., 2022) |
| 64 | Myrtaceae | II | Mentioned | Unmentioned | Mentioned | Mentioned | (Berger et al., 2016; Thornhill et al., 2015) |
| 65 | Nelumbonaceae | XIII | Mentioned | Mentioned | Unmentioned | Unmentioned | (Li et al., 2014) |
| 66 | Nepenthaceae | V | Unmentioned | Unmentioned | Mentioned | Unmentioned | (Biswal et al., 2018) |
| 67 | Ochnaceae | II.II | Mentioned | Mentioned | Mentioned | Mentioned | (Schneider et al., 2022) |
| 68 | Orchidaceae | I | Mentioned | Mentioned | Mentioned | Mentioned | (Givnish et al., 2016a) |
| 69 | Paeoniaceae | XII | Mentioned | Mentioned | Mentioned | Unmentioned | (Zhou et al., 2021) |
| 70 | Papaveraceae | XVIII | Mentioned | Mentioned | Mentioned | Unmentioned | (Peng et al., 2023) |
| 71 | Poaceae | I | Unmentioned | Unmentioned | Mentioned | Mentioned | (Gallaher et al., 2022) |
| 72 | Podocarpaceae | II | Mentioned | Unmentioned | Unmentioned | Mentioned | (Klaus and Matzke, 2020) |
| 73 | Sabiaceae | III | Mentioned | Mentioned | Mentioned | Mentioned | (Yang et al., 2018) |
| 74 | Sapindaceae | II | Mentioned | Mentioned | Mentioned | Mentioned | (Buerki et al., 2011; Wang et al., 2013) |
| 75 | Sarraceniaceae | XVI | Mentioned | Mentioned | Mentioned | Unmentioned | (Ellison et al., 2012) |
| 76 | Saxifragaceae | XVIII | Mentioned | Mentioned | Mentioned | Mentioned | (Deng et al., 2015; Ebersbach et al., 2017) |
| 77 | Scrophulariaceae | I | Mentioned | Unmentioned | Mentioned | Mentioned | (Villaverde et al., 2023) |
| 78 | Simaroubaceae | II | Unmentioned | Mentioned | Mentioned | Mentioned | (Clayton et al., 2009) |
| 79 | Smilacaceae | II | Mentioned | Mentioned | Mentioned | Mentioned | (Qi et al., 2023) |
| 80 | Solanaceae | I | Unmentioned | Mentioned | Unmentioned | Mentioned | (Dupin et al., 2017) |
| 81 | Stachyuraceae | XV | Mentioned | Unmentioned | Mentioned | Unmentioned | (Feng et al., 2020; Zhu et al., 2006) |
| 82 | Symplocaceae | II.I | Mentioned | Mentioned | Unmentioned | Mentioned | (Fritsch et al., 2015) |
| 83 | Tecophilaeaceae | XIV | Mentioned | Unmentioned | Mentioned | Unmentioned | (Buerki et al., 2013) |
| 84 | Theaceae | III | Mentioned | Mentioned | Mentioned | Unmentioned | (Yan et al., 2021; Yu et al., 2017) |
| 85 | Ulmaceae | I | Mentioned | Mentioned | Mentioned | Mentioned | (Zhang et al., 2022a) |
| 86 | Urticaceae | II | Mentioned | Mentioned | Mentioned | Mentioned | (Huang et al., 2019; Wu et al., 2018) |
| 87 | Velloziaceae | VII | Mentioned | Unmentioned | Mentioned | Unmentioned | (Alcantara et al., 2018; Mello-Silva et al., 2011) |
| 88 | Vochysiaceae | IX | Unmentioned | Unmentioned | Mentioned | Unmentioned | (Goncalves et al., 2020) |
| 89 | Zamiaceae | II | Mentioned | Mentioned | Mentioned | Unmentioned | (Coiro et al., 2023) |

**Table S5. Origin locations of 96 spermatophyte families**

| **Number** | **Family** | **Distribution type (including distribution subtype)** | **Origin place of stem group [Laurasia (Asia, North America, and uncertain region of Northern Hemisphere), Gondwana (Africa, Antarctica, Australia, South America, and uncertain region of Southern Hemisphere) and Both (tropics, uncertain region)]** | **Reference** |
| --- | --- | --- | --- | --- |
| 1 | Alstroemeriaceae | XVII | Gondwana (uncertain region of Southern Hemisphere) | (Chacón et al., 2012) |
| 2 | Altingiaceae | XIII | Laurasia (North America) | (Lai et al., 2021) |
| 3 | Alzateaceae | IX | Both (uncertain region) | (Berger et al., 2016) |
| 4 | Aphanopetalaceae | X | Gondwana (Australia) | (Chen et al., 2014) |
| 5 | Araceae | I | Laurasia (uncertain region of Northern Hemisphere) | (Nauheimer et al., 2012) |
| 6 | Araucariaceae | II.I | Both (uncertain region) | (Klaus and Matzke, 2020) |
| 7 | Asteliaceae | XVII | Gondwana (Australia) | (Birch and Kocyan, 2021) |
| 8 | Asteraceae | I | Gondwana (South America) | (Christenhusz et al., 2017) |
| 9 | Betulaceae | XVIII | Laurasia (uncertain region of Northern Hemisphere) | (Chen et al., 1999) |
| 10 | Biebersteiniaceae | XIV.I | Laurasia (Asia) | (Muellner et al., 2007) |
| 11 | Bignoniaceae | II | Gondwana (South America) | (Olmstead, 2013; Olmstead et al., 2009) |
| 12 | Blandfordiaceae | XVII.Ⅱ | Gondwana (Australia) | (Birch and Kocyan, 2021) |
| 13 | Boraginaceae | I | Gondwana (Africa) | (Luebert et al., 2017) |
| 14 | Boryaceae | XVII.Ⅱ | Gondwana (Australia) | (Birch and Kocyan, 2021) |
| 15 | Brassicaceae | I | Laurasia (Asia) | (Franzke et al., 2009) |
| 16 | Bromeliaceae | IX | Gondwana (South America) | (Givnish et al., 2011) |
| 17 | Burmanniaceae | II | Gondwana (uncertain region of Southern Hemisphere) | (Merckx et al., 2008) |
| 18 | Butomaceae | XII | Laurasia (uncertain region of Northern Hemisphere) | (Chen et al., 2013) |
| 19 | Campanulaceae | I | Gondwana (Africa) | (Crowl et al., 2016) |
| 20 | Campynemataceae | X | Gondwana (Australia) | (Givnish et al., 2016b) |
| 21 | Canellaceae | VII | Laurasia (North America) | (Müller et al., 2015) |
| 22 | Cannaceae | IX | Gondwana (uncertain region of Southern Hemisphere) | (Kress and Specht, 2006) |
| 23 | Caricaceae | VII | Gondwana (Africa) | (Carvalho and Renner, 2012) |
| 24 | Centroplacaceae | VI | Gondwana (uncertain region of Southern Hemisphere) | (Cai et al., 2016) |
| 25 | Chrysobalanaceae | II | Both (tropics) | (Bardon et al., 2016) |
| 26 | Colchicaceae | I | Gondwana (Australia) | (Chacón and Renner, 2014) |
| 27 | Combretaceae | II | Gondwana (uncertain region of Southern Hemisphere) | (Maurin et al., 2023) |
| 28 | Convolvulaceae | I | Both (tropics) | (Christenhusz et al., 2017) |
| 29 | Coriariaceae | XIV | Gondwana (Antarctica) | (Renner et al., 2020) |
| 30 | Corsiaceae | II.I | Gondwana (uncertain region of Southern Hemisphere) | (Mennes et al., 2015) |
| 31 | Corynocarpaceae | X | Both (tropics) | (Wagstaff and Dawson, 2000) |
| 32 | Crassulaceae | I | Gondwana (Africa) | (Christenhusz et al., 2017) |
| 33 | Crossosomataceae | XVI | Laurasia (uncertain region of Northern Hemisphere) | (Zhu et al., 2006) |
| 34 | Crypteroniaceae | VIII | Gondwana (uncertain region of Southern Hemisphere) | (Rutschmann et al., 2004) |
| 35 | Cucurbitaceae | II | Laurasia (Asia) | (Schaefer et al., 2009) |
| 36 | Cunoniaceae | II | Gondwana (Australia) | (Pillon et al., 2021) |
| 37 | Cupressaceae | XVIII | Laurasia (Asia) | (Mao et al., 2012) |
| 38 | Curtisiaceae | XVII.Ⅲ | Gondwana (Africa) | (Fu et al., 2019) |
| 39 | Cyperaceae | I | Gondwana (South America) | (Spalink et al., 2016) |
| 40 | Diapensiaceae | XII | Laurasia (North America) | (Gaynor et al., 2020) |
| 41 | Elatinaceae | I | Gondwana (Africa) | (Cai et al., 2016) |
| 42 | Eriocaulaceae | II | Gondwana (uncertain region of Southern Hemisphere) | (Janssen and Bremer, 2004; Trovó et al., 2013) |
| 43 | Gesneriaceae | II | Gondwana (South America) | (Roalson and Roberts, 2016) |
| 44 | Grubbiaceae | XVII.Ⅲ | Gondwana (Africa) | (Fu et al., 2019) |
| 45 | Gunneraceae | II | Gondwana (uncertain region of Southern Hemisphere) | (Fuller and Hickey, 2005) |
| 46 | Halophytaceae | XVII.I | Gondwana (South America) | (Ocampo and Columbus, 2010) |
| 47 | Haloragaceae | I | Gondwana (Australia) | (Chen et al., 2014) |
| 48 | Heliconiaceae | IX | Gondwana (South America) | (Kress and Specht, 2006) |
| 49 | Helwingiaceae | XV | Laurasia (uncertain region of Northern Hemisphere) | (Wu et al., 2003a) |
| 50 | Humiriaceae | IX | Gondwana (South America) | (Herrera et al., 2014) |
| 51 | Hypoxidaceae | II | Gondwana (Africa) | (Birch and Kocyan, 2021) |
| 52 | Lamiaceae | I | Laurasia (Asia) | (Rose et al., 2022) |
| 53 | Lanariaceae | XVII.Ⅲ | Gondwana (Africa) | (Birch and Kocyan, 2021) |
| 54 | Lardizabalaceae | XVIII | Laurasia (Asia) | (Wang et al., 2020) |
| 55 | Liliaceae | XII | Laurasia (North America) | (Vinnersten and Bremer, 2001) |
| 56 | Loranthaceae | II | Gondwana (Australia) | (Liu et al., 2018) |
| 57 | Lowiaceae | VIII | Both (tropics) | (Kress and Specht, 2006) |
| 58 | Malpighiaceae | II | Gondwana (Africa) | (Cai et al., 2016) |
| 59 | Marantaceae | II | Gondwana (Africa) | (Prince and Kress, 2006) |
| 60 | Maundiaceae | XVII.II | Gondwana (Australia) | (Chen et al., 2013) |
| 61 | Mazaceae | XVIII | Laurasia (uncertain region of Northern Hemisphere) | (Deng, 2015) |
| 62 | Melanthiaceae | XII | Laurasia (North America) | (Givnish et al., 2016b) |
| 63 | Musaceae | IV | Both (tropics) | (Kress and Specht, 2006) |
| 64 | Myricaceae | I | Gondwana (Australia) | (Herbert, 2004) |
| 65 | Myrtaceae | II | Gondwana (Africa) | (Berger et al., 2016) |
| 66 | Nelumbonaceae | XIII | Laurasia (uncertain region of Northern Hemisphere) | (Li et al., 2014) |
| 67 | Nepenthaceae | V | Gondwana (Africa) | (Biswal et al., 2018) |
| 68 | Orchidaceae | I | Gondwana (Australia) | (Givnish et al., 2016a) |
| 69 | Orobanchaceae | I | Laurasia (uncertain region of Northern Hemisphere) | (Wolfe et al., 2005) |
| 70 | Penthoraceae | XIII | Gondwana (Australia) | (Chen et al., 2014) |
| 71 | Petermanniaceae | XVII.II | Gondwana (Australia) | (Givnish et al., 2016b) |
| 72 | Philesiaceae | XVII.Ⅰ | Gondwana (uncertain region of Southern Hemisphere) | (Givnish et al., 2016b; Vinnersten and Bremer, 2001) |
| 73 | Pittosporaceae | IV | Gondwana (Australia) | (Chandler et al., 2007) |
| 74 | Podocarpaceae | II | Both (uncertain region) | (Klaus and Matzke, 2020) |
| 75 | Polygonaceae | I | Gondwana (uncertain region of Southern Hemisphere) | (Schuster et al., 2013) |
| 76 | Potamogetonaceae | I | Gondwana (Australia) | (Chen et al., 2013) |
| 77 | Restionaceae | XVII | Gondwana (uncertain region of Southern Hemisphere) | (Christenhusz et al., 2017) |
| 78 | Ripogonaceae | X | Gondwana (uncertain region of Southern Hemisphere) | (Givnish et al., 2016b; Vinnersten and Bremer, 2001) |
| 79 | Rubiaceae | I | Both (tropics) | (Christenhusz et al., 2017) |
| 80 | Rutaceae | II | Both (tropics) | (Appelhans et al., 2012) |
| 81 | Sabiaceae | III | Laurasia (uncertain region of Northern Hemisphere) | (Yang et al., 2018) |
| 82 | Sapindaceae | II | Both (tropics) | (Wang et al., 2013) |
| 83 | Sarraceniaceae | XVI | Gondwana (South America) | (Ellison et al., 2012) |
| 84 | Saururaceae | XIII | Laurasia (North America) | (Grímsson et al., 2017) |
| 85 | Scheuchzeriaceae | XII | Laurasia (uncertain region of Northern Hemisphere) | (Chen et al., 2015) |
| 86 | Smilacaceae | Ⅱ | Laurasia (uncertain region of Northern Hemisphere) | (Qi et al., 2023) |
| 87 | Solanaceae | I | Gondwana (South America) | (Dupin et al., 2017) |
| 88 | Stachyuraceae | XV | Laurasia (uncertain region of Northern Hemisphere) | (Zhu et al., 2006) |
| 89 | Strelitziaceae | VII | Both (tropics) | (Kress and Specht, 2006) |
| 90 | Tetracarpaeaceae | XVII.II | Gondwana (Australia) | (Chen et al., 2014) |
| 91 | Urticaceae | II | Laurasia (uncertain region of Northern Hemisphere) | (Wu et al., 2018) |
| 92 | Velloziaceae | VII | Gondwana (uncertain region of Southern Hemisphere) | (Mello-Silva et al., 2011) |
| 93 | Verbenaceae | I | Gondwana (South America) | (Marx et al., 2010) |
| 94 | Vochysiaceae | IX | Gondwana (uncertain region of Southern Hemisphere) | (Goncalves et al., 2020) |
| 95 | Xeronemataceae | X | Gondwana (Australia) | (Bremer and Janssen, 2006) |
| 96 | Zosteraceae | XVIII | Gondwana (Australia) | (Chen et al., 2013) |

**Table S6.** **Formation time of corresponding distribution type of 121 spermatophyte families**

| **Number** | **Family** | **Distribution type (including distribution subtype)** | **Minimum age for the formation of distribution type** | **Maximum age for the formation of distribution type** | **Dating method** | **Note** | **Reference** |
| --- | --- | --- | --- | --- | --- | --- | --- |
| 1 | Akaniaceae | V | 16.63 | 16.63 | secondary calibration | This time is the crown age of this family, namely the formation time of tropical Asia to tropical Australasia distribution in this family | (Smith and Brown, 2018) |
| 2 | Alismataceae | I | 5.33 | 23.03 | fossil calibration | This time is the formation time of widespread distribution in this family. | (Chen et al., 2012a; Li et al., 2022) |
| 3 | Alstroemeriaceae | XVII | 37.80 | 77.60 | fossil calibration and secondary calibration | This time is the crown age of this family, namely the formation time of south temperate in this family. | (Chacón et al., 2012) |
| 4 | Alzateaceae | IX | 30.89 | 30.89 | secondary calibration | This time is the stem age of this family, namely the formation time of New World tropics distribution in this family. | (Berger et al., 2016; Smith and Brown, 2018) |
| 5 | Anisophylleaceae | II.II | 22.00 | 37.00 | fossil calibration and substitution rate | This time is the formation time of the tropical Asia-tropical Africa-tropical America disjunct distribution in this family. | (Zhang et al., 2007) |
| 6 | Annonaceae | II | 5.33 | 23.03 | fossil calibration | This time is the formation time of the pantropic disjunct distribution in this family. | (Onstein et al., 2019) |
| 7 | Aphanopetalaceae | X | 75.70 | 92.00 | fossil calibration | This time is the stem age of this family, namely the formation time of tropical Australasia distribution in this family. | (Chen et al., 2014) |
| 8 | Apocynaceae | II | 33.90 | 56.00 | fossil calibration | This time is the formation time of pantropic disjunct distribution in this family. | (Bitencourt et al., 2021; Fishbein et al., 2018) |
| 9 | Aponogetonaceae | IV | 5.33 | 23.03 | substitution rate | This time is the formation time of the Old World tropics distribution in this family. | (Chen et al., 2015) |
| 10 | Araceae | I | 33.90 | 56.00 | fossil calibration | This time is the formation time of the widespread distribution in this family. | (Nauheimer et al., 2012) |
| 11 | Argophyllaceae | X | 11.30 | 46.00 | fossil calibration | This time is the crown age of this family, namely the formation time of tropical Australasia distribution in this family. | (Maurin and Smissen, 2022) |
| 12 | Asteliaceae | XVII | 3.70 | 23.10 | fossil calibration and secondary calibration | This time is the formation time of south temperate in this family. | (Birch and Keeley, 2013) |
| 13 | Balsaminaceae | VI | 2.58 | 5.33 | fossil calibration and secondary calibration | This time is the formation time of the tropical Asia to tropical Africa distribution in this family. | (Janssens et al., 2009) |
| 14 | Berberidopsidaceae | XVII | 4.24 | 16.81 | secondary calibration | This time is the formation time of south temperate in this family. | (Menegoz et al., 2024) |
| 15 | Betulaceae | XVIII | 23.03 | 33.90 | fossil et al. | This time is the formation time of the north and south temperate disjunct distribution in this family. | (Chen et al., 1999) |
| 16 | Biebersteiniaceae | XIV.I | 33.90 | 66.00 | fossil calibration and substitution rate | This time is the diversification time of this family in Mediterranea, West Asia to Central Asia. | (Muellner et al., 2007) |
| 17 | Blandfordiaceae | XVII.II | 55.60 | 82.60 | fossil calibration and secondary calibration | This time is the stem age of this family, namely the formation time of temperate Australasia in this family. | (Birch and Kocyan, 2021) |
| 18 | Boraginaceae | I | 33.90 | 56.00 | fossil calibration and secondary calibration | This time is the formation time of the widespread distribution in this family. | (Luebert et al., 2017) |
| 19 | Boryaceae | XVII.II | 61.00 | 90.00 | fossil calibration and secondary calibration | This time is the stem age of this family, namely the formation time of temperate Australasia in this family. | (Birch and Kocyan, 2021) |
| 20 | Bromeliaceae | IX | 11.63 | 15.98 | fossil calibration and secondary calibration | This time is the formation time of New World tropics distribution in this family. | (Givnish et al., 2011) |
| 21 | Butomaceae | XII | 63.68 | 63.68 | secondary calibration | This time is the stem age of this family, namely the formation time of north temperate distribution in this family. | (Chen et al., 2013; Smith and Brown, 2018) |
| 22 | Campanulaceae | I | 33.9 | 56 | fossil calibration and secondary calibration | This time is the formation time of the widespread distribution in this family. | (Crowl et al., 2016) |
| 23 | Campynemataceae | X | 15.10 | 70.90 | fossil calibration and secondary calibration | This time is the crown age of this family, namely the formation time of tropical Australasia distribution in this family. | (Givnish et al., 2016b) |
| 24 | Canellaceae | VII | 35.00 | 50.00 | fossil calibration | This time is the formation time of South Atlantic disjunct distribution in this family. | (Müller et al., 2015) |
| 25 | Cannabaceae | I | 2.58 | 5.33 | fossil calibration | This time is the formation time of the widespread distribution in this family. | (Jin et al., 2020) |
| 26 | Cannaceae | IX | 24.60 | 34.00 | fossil calibration and secondary calibration | This time is the crown age of this family, namely the formation time of New World tropics distribution in this family. | (Kress and Specht, 2006) |
| 27 | Caricaceae | VII | 28.10 | 43.10 | secondary calibration | This time is the formation time of South Atlantic disjunct distribution in this family. | (Carvalho and Renner, 2012) |
| 28 | Centroplacaceae | VI | 78.50 | 87.70 | fossil calibration and secondary calibration | This time is the formation time of the tropical Asia to tropical Africa distribution in this family. | (Cai et al., 2016) |
| 29 | Cephalotaceae | XVII.II | 95.50 | 95.50 | fossil calibration | This time is the stem age of this family, namely the formation time of temperate Australasia in this family. | (Pillon et al., 2021) |
| 30 | Cephalotaxaceae | XV | 0.01 | 2.58 | fossil et al. | This time is the formation time of East Asia distribution in this family. | (Shi et al., 2010) |
| 31 | Cercidiphyllaceae | XV | 1.34 | 13.42 | fossil calibration | This time is the crown age of this family, namely the formation time of East Asia distribution in this family. | (Qi et al., 2012) |
| 32 | Chrysobalanaceae | II | 23.03 | 33.90 | fossil calibration | This time is the formation time of the pantropic disjunct distribution in this family. | (Bardon et al., 2016) |
| 33 | Circaeasteraceae | XV | 26.12 | 83.13 | fossil calibration and secondary calibration | This time is the crown age of this family, namely the formation time of East Asia distribution in this family. | (Zhang et al., 2020) |
| 34 | Colchicaceae | I | 5.33 | 23.03 | fossil calibration and secondary calibration | This time is the formation time of the widespread distribution in this family. | (Chacón and Renner, 2014) |
| 35 | Combretaceae | II | 56 | 66 | fossil calibration and secondary calibration | This time is the formation time of the pantropic disjunct distribution in this family. | (Maurin et al., 2023) |
| 36 | Corsiaceae | II.I | 30.00 | 76.00 | fossil calibration, secondary calibration and substitution rate | This time is the formation time of the tropical Asia-Australasia and tropical America disjunct distribution in this family. | (Mennes et al., 2015) |
| 37 | Costaceae | II | 33.90 | 56.00 | secondary calibration | This time is the formation time of the pantropic disjunct distribution in this family. | (Specht, 2006) |
| 38 | Crypteroniaceae | VIII | 23.03 | 66.00 | calibration of fossil and geological event | This time is the crown age of this family, namely the formation time of tropical Asia distribution in this family. | (Rutschmann et al., 2004) |
| 39 | Cucurbitaceae | II | 11.63 | 15.98 | fossil calibration | This time is the formation time of the pantropic disjunct distribution in this family. | (Schaefer et al., 2009) |
| 40 | Cunoniaceae | II | 23.03 | 33.90 | fossil calibration | This time is the formation time of the pantropic disjunct distribution in this family. | (Pillon et al., 2021) |
| 41 | Cupressaceae | XVIII | 124.00 | 183.00 | fossil calibration | This time is the formation time of the north and south temperate disjunct distribution in this family. | (Mao et al., 2012) |
| 42 | Curtisiaceae | XVII.III | 0.50 | 0.50 | fossil calibration | This time is the crown age of this family, namely the formation time of temperate Africa in this family. | (Fu et al., 2019) |
| 43 | Cycadaceae | V | 5.33 | 23.03 | calibration of fossil and geological event, secondary calibration and substitution rate | This time is the formation time of the tropical Asia to tropical Australasia distribution in this family. | (Coiro et al., 2023; Liu et al., 2024) |
| 44 | Cyperaceae | I | 33.90 | 56.00 | fossil calibration and secondary calibration | This time is the formation time of the widespread distribution in this family. | (Spalink et al., 2016) |
| 45 | Cyrillaceae | IX | 8.95 | 8.95 | secondary calibration | This time is the crown age of this family, namely the formation time of New World tropics distribution in this family. | (Smith and Brown, 2018) |
| 46 | Datiscaceae | XIV | 12.86 | 12.86 | secondary calibration | This time is the crown age of this family, namely the formation time of pan-Mediterranean diffused distribution in this family. | (Smith and Brown, 2018) |
| 47 | Degeneriaceae | X.II | 3.13 | 3.13 | secondary calibration | This time is the crown age of this family, namely the formation time of Fiji distribution in this family. | (Smith and Brown, 2018) |
| 48 | Diapensiaceae | XII | 23.03 | 33.90 | secondary calibration | This time is the formation time of the north temperate distribution in this family. | (Gaynor et al., 2020) |
| 49 | Dipterocarpaceae | VI | 33.90 | 56.00 | fossil calibration | This time is the formation time of tropical Asia to tropical Africa distribution in this family. | (Bansal et al., 2022) |
| 50 | Doryanthaceae | XVII.II | 11.81 | 11.81 | secondary calibration | This time is the crown age of this family, namely the formation time of temperate Australasia in this family. | (Smith and Brown, 2018) |
| 51 | Drosophyllaceae | XIV.I | 0.27 | 1.65 | fossil calibration and secondary calibration | This time is the crown age of this family, namely the formation time of Mediterranea, West Asia to Central Asia distribution in this family. | (Martin-Rodriguez et al., 2020) |
| 52 | Elaeocarpaceae | II.I | 33.90 | 56.00 | fossil calibration | This time is the formation time of the tropical Asia-Australasia and tropical America disjunct distribution in this family. | (Pillon et al., 2021) |
| 53 | Elatinaceae | I | 5.33 | 33.90 | fossil calibration and secondary calibration | This time is the formation time of the widespread distribution in this family. | (Cai et al., 2016) |
| 54 | Ephedraceae | XVIII | 8.84 | 41.53 | fossil calibration | This time is the formation time of north and south temperate disjunct distribution in this family. | (Ickert-Bond et al., 2009) |
| 55 | Eucommiaceae | XV | 0.01 | 2.58 | fossil et al. | This time is the formation time of East Asia distribution in this family. | (Guo, 2000; Zhou and Momohara, 2005) |
| 56 | Eupomatiaceae | X | 10.92 | 10.92 | secondary calibration | This time is the crown age of this family, namely the formation time of tropical Australasia distribution in this family. | (Smith and Brown, 2018) |
| 57 | Eupteleaceae | XV | 1.23 | 10.87 | fossil calibration | This time is the crown age of this family, namely the formation time of East Asia distribution in this family. | (Cao et al., 2016) |
| 58 | Gesneriaceae | II | 33.90 | 56.00 | calibration of fossil and geological event, and secondary calibration | This time is the formation time of the pantropic disjunct distribution in this family. | (Roalson and Roberts, 2016) |
| 59 | Ginkgoaceae | XV | 0.52 | 0.52 | uncertainty | This time is the crown age of this family, namely the formation time of East Asia distribution in this family. | (Zhao et al., 2019) |
| 60 | Grubbiaceae | XVII.III | 18.10 | 18.10 | fossil calibration | This time is the crown age of this family, namely the formation time of temperate Africa in this family. | (Fu et al., 2019) |
| 61 | Halophytaceae | XVII.I | 6.10 | 31.40 | geological event calibration | This time is the stem age of this family, namely the formation time of temperate South America in this family. | (Ocampo and Columbus, 2010) |
| 62 | Haloragaceae | I | 5.33 | 23.03 | fossil calibration | This time is the formation time of the widespread distribution in this family. | (Chen et al., 2014) |
| 63 | Heliconiaceae | IX | 21.00 | 43.00 | fossil calibration and secondary calibration | This time is the crown age of this family, namely the formation time of New World tropics distribution in this family. | (Kress and Specht, 2006) |
| 64 | Hydatellaceae | XVII.II | 15.70 | 23.40 | fossil calibration | This time is the crown age of this family, namely the formation time of temperate Australasia in this family. | (Iles et al., 2014) |
| 65 | Hydrocharitaceae | I | 5.33 | 33.90 | fossil calibration | This time is the formation time of the widespread distribution in this family. | (Chen et al., 2012b) |
| 66 | Hypoxidaceae | II | 3.60 | 5.33 | fossil calibration and secondary calibration | This time is the formation time of pantropic disjunct distribution in this family. | (Birch and Kocyan, 2021) |
| 67 | Juglandaceae | XII | 5.33 | 23.03 | fossil calibration | This time is the formation time of north temperate distribution in this family. | (Zhang et al., 2022b) |
| 68 | Lamiaceae | I | 5.33 | 23.03 | fossil calibration and secondary calibration | This time is the formation time of widespread distribution in this family. | (Rose et al., 2022) |
| 69 | Lanariaceae | XVII.III | 40.30 | 61.40 | fossil calibration and secondary calibration | This time is the stem age of this family, namely the formation time of temperate Africa in this family. | (Birch and Kocyan, 2021) |
| 70 | Lardizabalaceae | XVIII | 12.23 | 41.31 | fossil calibration and secondary calibration | This time is the formation time of the tropical Asia and tropical America disjunct distribution in this family. | (Wang et al., 2020) |
| 71 | Lepidobotryaceae | VII | 21.72 | 21.72 | secondary calibration | This time is the crown age of this family, namely the formation time of South Atlantic disjunct distribution in this family. | (Smith and Brown, 2018) |
| 72 | Liliaceae | XII | 33.90 | 56.00 | fossil calibration and secondary calibration | This time is the formation time of the north temperate distribution in this family. | (Givnish et al., 2016b) |
| 73 | Loranthaceae | II | 23.03 | 33.90 | fossil calibration | This time is the formation time of the pantropic disjunct distribution in this family. | (Liu et al., 2018) |
| 74 | Lowiaceae | VIII | 9.10 | 16.90 | fossil calibration and secondary calibration | This time is the crown age of this family, namely the formation time of tropical Asia distribution in this family. | (Kress and Specht, 2006) |
| 75 | Magnoliaceae | XIII | 10.57 | 30.22 | fossil calibration | This time is the formation time of East Asia and North America disjunct distribution in this family. | (Nie et al., 2008) |
| 76 | Malpighiaceae | II | 33.90 | 56.00 | fossil calibration and secondary calibration | This time is the formation time of the pantropic disjunct distribution in this family. | (Cai et al., 2016) |
| 77 | Maundiaceae | XVII.II | 75.00 | 75.00 | secondary calibration | This time is the stem age of this family, namely the formation time of temperate Australasia in this family. | (Chen et al., 2013; Janssen and Bremer, 2004) |
| 78 | Mazaceae | XVIII | 6.46 | 26.30 | fossil calibration and secondary calibration | This time is the formation time of the north and south temperate disjunct distribution in this family. | (Deng, 2015) |
| 79 | Melanthiaceae | XII | 5.33 | 23.03 | fossil calibration and secondary calibration | This time is the formation time of north temperate distribution in this family. | (Givnish et al., 2016b) |
| 80 | Menispermaceae | II | 60.00 | 70.00 | fossil calibration and secondary calibration | This time is the formation time of the pantropic disjunct distribution in this family. | (Wang et al., 2012) |
| 81 | Monimiaceae | II | 23.03 | 33.90 | fossil calibration | This time is the formation time of the pantropic disjunct distribution in this family. | (Renner et al., 2010) |
| 82 | Musaceae | IV | 5.33 | 23.03 | fossil calibration and secondary calibration | This time is the formation time of the Old World tropics distribution in this family. | (Janssens et al., 2016) |
| 83 | Myristicaceae | II | 33.90 | 56.00 | secondary calibration | This time is the formation time of the pantropic disjunct distribution in this family. | (Frost et al., 2022) |
| 84 | Myrothamnaceae | XI | 8.60 | 8.60 | secondary calibration | This time is the crown age of this family, namely the formation time of tropical Africa distribution in this family. | (Smith and Brown, 2018) |
| 85 | Myrtaceae | II | 5.33 | 23.03 | fossil calibration and secondary calibration | This time is the formation time of the pantropic disjunct distribution in this family. | (Thornhill et al., 2015) |
| 86 | Nelumbonaceae | XIII | 0.30 | 4.20 | fossil calibration | This time is the formation time of East Asia and North America disjunct distribution in this family. | (Xue et al., 2012) |
| 87 | Nepenthaceae | V | 6.36 | 6.36 | uncertainty | This time is the formation time of the tropical Asia to tropical Australasia distribution in this family. | (Biswal et al., 2018) |
| 88 | Ochnaceae | II.II | 23.03 | 33.90 | secondary calibration | This time is the formation time of tropical Asia-tropical Africa-tropical America disjunct distribution in this family. | (Schneider et al., 2022) |
| 89 | Orchidaceae | I | 33.90 | 56.00 | fossil calibration and secondary calibration | This time is the formation time of the widespread distribution in this family. | (Givnish et al., 2016a) |
| 90 | Paeoniaceae | XII | 5.33 | 23.03 | calibration of fossil and geological event, and secondary calibration | This time is the formation time of north temperate distribution in this family. | (Zhou et al., 2021) |
| 91 | Papaveraceae | XVIII | 33.90 | 56.00 | fossil calibration and secondary calibration | This time is the formation time of the north and south temperate disjunct distribution in this family. | (Peng et al., 2023) |
| 92 | Pennantiaceae | XVII.II | 21.67 | 21.67 | secondary calibration | This time is the crown age of this family, namely the formation time of temperate Australasia in this family. | (Smith and Brown, 2018) |
| 93 | Penthoraceae | XIII | 1.00 | 5.80 | fossil calibration | This time is the formation time of East Asia and North America disjunct distribution in this family. | (Chen et al., 2014) |
| 94 | Petermanniaceae | XVII.II | 82.10 | 117.20 | fossil calibration and secondary calibration | This time is the stem age of this family, namely the formation time of temperate Australasia in this family. | (Givnish et al., 2016b) |
| 95 | Philesiaceae | XVII.I | 2.30 | 36.60 | fossil calibration and secondary calibration | This time is the crown age of this family, namely the formation time of temperate South America in this family. | (Givnish et al., 2016b) |
| 96 | Poaceae | I | 33.90 | 56.00 | fossil calibration | This time is the formation time of the widespread distribution in this family. | (Gallaher et al., 2022) |
| 97 | Podocarpaceae | II | 5.33 | 23.03 | fossil calibration | This time is the formation time of the pantropic disjunct distribution in this family. | (Klaus and Matzke, 2020) |
| 98 | Sabiaceae | III | 5.33 | 23.03 | fossil calibration and secondary calibration | This time is the formation time of the tropical Asia and tropical America disjunct distribution in this family. | (Yang et al., 2018) |
| 99 | Sapindaceae | II | 33.90 | 56.00 | fossil calibration | This time is the formation time of the pantropic disjunct distribution in this family. | (Buerki et al., 2011) |
| 100 | Sarraceniaceae | XVI | 25.00 | 44.00 | secondary calibration | This time is the crown age of this family, namely the formation time of North America distribution in this family. | (Ellison et al., 2012) |
| 101 | Saxifragaceae | XVIII | 23.03 | 33.90 | fossil calibration | This time is the formation time of the north and south temperate disjunct distribution in this family. | (Ebersbach et al., 2017) |
| 102 | Scheuchzeriaceae | XII | 79.59 | 79.59 | secondary calibration | This time is the stem age of this family, namely the formation time of north temperate distribution in this family. | (Chen et al., 2015; Smith and Brown, 2018) |
| 103 | Sciadopityaceae | XV.I | 0.01 | 2.58 | fossil et al. | This time is the formation time of Japan distribution in this family. | (Worth et al., 2013) |
| 104 | Scrophulariaceae | I | 23.03 | 33.9 | secondary calibration | This time is the formation time of the widespread distribution in this family. | (Villaverde et al., 2023) |
| 105 | Setchellanthaceae | XVI | 2.70 | 13.82 | fossil calibration | This time is the crown age of this family, namely the formation time of North America distribution in this family. | (Hernández-Hernández et al., 2013) |
| 106 | Simaroubaceae | II | 5.33 | 23.03 | fossil calibration | This time is the formation time of the pantropic disjunct distribution in this family. | (Clayton et al., 2009) |
| 107 | Smilacaceae | II | 0.68 | 5.80 | fossil calibration | This time is the formation time of pantropic disjunct distribution in this family. | (Qi et al., 2023) |
| 108 | Solanaceae | I | 5.33 | 23.03 | fossil calibration and substitution rate | This time is the formation time of the widespread distribution in this family. | (Dupin et al., 2017; Särkinen et al., 2013) |
| 109 | Stachyuraceae | XV | 5.20 | 17.10 | fossil calibration | This time is the crown age of this family, namely the formation time of East Asia distribution in this family. | (Feng et al., 2020; Zhu et al., 2006) |
| 110 | Strasburgeriaceae | X | 23.51 | 23.51 | secondary calibration | This time is the crown age of this family, namely the formation time of tropical Australasia distribution in this family. | (Smith and Brown, 2018) |
| 111 | Strelitziaceae | VII | 44.00 | 54.00 | fossil calibration and secondary calibration | This time is the formation time of South Atlantic disjunct distribution in this family. | (Kress and Specht, 2006) |
| 112 | Symplocaceae | II.I | 34.00 | 37.00 | fossil calibration | This time is the formation time of the tropical Asia-Australasia and tropical America disjunct distribution in this family. | (Fritsch et al., 2015) |
| 113 | Tecophilaeaceae | XIV | 56.00 | 66.00 | secondary calibration | This time is the formation time of pan-Mediterranean diffused distribution in this family. | (Buerki et al., 2013) |
| 114 | Tetracarpaeaceae | XVII.II | 72.90 | 85.30 | fossil calibration | This time is the stem age of this family, namely the formation time of temperate Australasia in this family. | (Chen et al., 2014) |
| 115 | Theaceae | III | 15.98 | 27.82 | fossil calibration | This time is the formation time of the tropical Asia and tropical America disjunct distribution in this family. | (Yan et al., 2021) |
| 116 | Trochodendraceae | XV | 30.00 | 44.00 | fossil calibration | This time is the crown age of this family, namely the formation time of East Asia distribution in this family. | (Sun et al., 2013) |
| 117 | Ulmaceae | I | 33.90 | 56.00 | fossil calibration | This time is the formation time of the widespread distribution in this family. | (Zhang et al., 2022a) |
| 118 | Urticaceae | II | 5.33 | 23.03 | fossil calibration | This time is the formation time of the pantropic disjunct distribution in this family. | (Wu et al., 2018) |
| 119 | Velloziaceae | VII | 40.00 | 55.00 | fossil calibration | This time is the formation time of South Atlantic disjunct distribution in this family. | (Alcantara et al., 2018) |
| 120 | Vochysiaceae | IX | 5.33 | 23.03 | fossil calibration and secondary calibration | This time is the formation time of New World tropics distribution in this family. | (Goncalves et al., 2020) |
| 121 | Zamiaceae | II | 5.33 | 23.03 | fossil calibration and substitution rate | This time is the formation time of the pantropic disjunct distribution in this family. | (Coiro et al., 2023) |

**Table S7.** **Distribution types of 111 mixed annual and perennial herbaceous families**

| **Number** | **Mixed annual and perennial herbaceous family** | **Distribution type (including distribution subtype)** |
| --- | --- | --- |
| 1 | Acanthaceae | Ⅱ |
| 2 | Aizoaceae | Ⅱ |
| 3 | Alismataceae | Ⅰ |
| 4 | Alstroemeriaceae | XVII |
| 5 | Amaranthaceae | Ⅰ |
| 6 | Apiaceae | Ⅰ |
| 7 | Apocynaceae | Ⅱ |
| 8 | Asparagaceae | Ⅰ |
| 9 | Asteraceae | Ⅰ |
| 10 | Balanophoraceae | Ⅱ |
| 11 | Balsaminaceae | Ⅵ |
| 12 | Begoniaceae | Ⅱ |
| 13 | Boraginaceae | Ⅰ |
| 14 | Brassicaceae | Ⅰ |
| 15 | Burmanniaceae | Ⅱ |
| 16 | Calyceraceae | XVII.Ⅰ |
| 17 | Campanulaceae | Ⅰ |
| 18 | Cannabaceae | Ⅰ |
| 19 | Caprifoliaceae | Ⅻ |
| 20 | Caryophyllaceae | Ⅰ |
| 21 | Celastraceae | Ⅰ |
| 22 | Circaeasteraceae | XV |
| 23 | Cistaceae | XIV |
| 24 | Cleomaceae | Ⅱ |
| 25 | Commelinaceae | Ⅱ |
| 26 | Convolvulaceae | Ⅰ |
| 27 | Crassulaceae | Ⅰ |
| 28 | Cucurbitaceae | Ⅱ |
| 29 | Cyperaceae | Ⅰ |
| 30 | Droseraceae | Ⅰ |
| 31 | Elatinaceae | Ⅰ |
| 32 | Eriocaulaceae | Ⅱ |
| 33 | Euphorbiaceae | Ⅱ |
| 34 | Fabaceae | Ⅰ |
| 35 | Francoaceae | Ⅶ |
| 36 | Frankeniaceae | XIV |
| 37 | Gentianaceae | Ⅰ |
| 38 | Geraniaceae | XVIII |
| 39 | Gisekiaceae | Ⅵ |
| 40 | Goodeniaceae | Ⅱ |
| 41 | Gunneraceae | Ⅱ |
| 42 | Gyrostemonaceae | XVII.Ⅱ |
| 43 | Haloragaceae | Ⅰ |
| 44 | Hydatellaceae | XVII.Ⅱ |
| 45 | Hydrocharitaceae | Ⅰ |
| 46 | Hydroleaceae | Ⅱ |
| 47 | Hypericaceae | Ⅰ |
| 48 | Iridaceae | Ⅰ |
| 49 | Juncaceae | XVIII |
| 50 | Juncaginaceae | Ⅰ |
| 51 | Kewaceae | XVII.Ⅲ |
| 52 | Lamiaceae | Ⅰ |
| 53 | Lentibulariaceae | Ⅰ |
| 54 | Limeaceae | Ⅵ |
| 55 | Linaceae | Ⅰ |
| 56 | Linderniaceae | Ⅱ |
| 57 | Loasaceae | Ⅸ |
| 58 | Loganiaceae | Ⅱ |
| 59 | Lophiocarpaceae | Ⅵ |
| 60 | Lythraceae | Ⅰ |
| 61 | Malvaceae | Ⅱ |
| 62 | Martyniaceae | Ⅸ |
| 63 | Mazaceae | XVIII |
| 64 | Melastomataceae | Ⅱ |
| 65 | Menyanthaceae | Ⅰ |
| 66 | Molluginaceae | Ⅱ |
| 67 | Montiaceae | XVIII |
| 68 | Neuradaceae | XIV |
| 69 | Nitrariaceae | XIV |
| 70 | Nyctaginaceae | Ⅱ |
| 71 | Nymphaeaceae | Ⅰ |
| 72 | Ochnaceae | Ⅱ.Ⅱ |
| 73 | Onagraceae | Ⅰ |
| 74 | Orobanchaceae | Ⅰ |
| 75 | Oxalidaceae | Ⅰ |
| 76 | Papaveraceae | XVIII |
| 77 | Passifloraceae | Ⅱ |
| 78 | Pedaliaceae | Ⅳ |
| 79 | Petiveriaceae | Ⅸ |
| 80 | Phrymaceae | Ⅰ |
| 81 | Phyllanthaceae | Ⅱ |
| 82 | Piperaceae | Ⅱ |
| 83 | Plantaginaceae | Ⅰ |
| 84 | Plumbaginaceae | Ⅰ |
| 85 | Poaceae | Ⅰ |
| 86 | Podostemaceae | Ⅱ |
| 87 | Polemoniaceae | XVIII |
| 88 | Polygalaceae | Ⅰ |
| 89 | Polygonaceae | Ⅰ |
| 90 | Pontederiaceae | Ⅱ |
| 91 | Portulacaceae | Ⅰ |
| 92 | Potamogetonaceae | Ⅰ |
| 93 | Primulaceae | Ⅰ |
| 94 | Ranunculaceae | Ⅰ |
| 95 | Resedaceae | Ⅻ |
| 96 | Rhamnaceae | Ⅰ |
| 97 | Rosaceae | Ⅰ |
| 98 | Rubiaceae | Ⅰ |
| 99 | Ruppiaceae | Ⅰ |
| 100 | Santalaceae | Ⅰ |
| 101 | Saxifragaceae | XVIII |
| 102 | Scrophulariaceae | Ⅰ |
| 103 | Solanaceae | Ⅰ |
| 104 | Stylidiaceae | XVII |
| 105 | Talinaceae | Ⅶ |
| 106 | Tropaeolaceae | XVII.Ⅰ |
| 107 | Urticaceae | Ⅱ |
| 108 | Vahliaceae | Ⅵ |
| 109 | Verbenaceae | Ⅰ |
| 110 | Violaceae | Ⅰ |
| 111 | Zygophyllaceae | Ⅰ |

**Table S8. Distribution situation of each family of global spermatophytes in the north temperate zone (including north frigid zone), the tropical zone, the south temperate zone (including south frigid zone) and seven continent**

The 0 and 1 represent non-distribution center and distribution center of a family in the above regions, respectively.

| **Number** | **Family** | **North temperate zone** | **Tropical zone** | **South temperate zone** | **Asia** | **Oceania** | **Europe** | **Africa** | **South America** | **North America** | **Antarctica** | **Notes** |
| --- | --- | --- | --- | --- | --- | --- | --- | --- | --- | --- | --- | --- |
| 1 | Acanthaceae | 0 | 1 | 0 | 1 | 0 | 0 | 1 | 1 | 1 | 0 |  |
| 2 | Achariaceae | 0 | 1 | 0 | 1 | 1 | 0 | 1 | 1 | 1 | 0 |  |
| 3 | Achatocarpaceae | 0 | 1 | 0 | 0 | 0 | 0 | 0 | 1 | 1 | 0 |  |
| 4 | Acoraceae | 1 | 0 | 0 | 1 | 0 | 0 | 0 | 0 | 1 | 0 | This family exists in Europe and Africa in *Plants of the world* and ANGIOSPERM PHYLOGENY WEBSITE (http://www.mobot.org/mobot/research/apweb/welcome.html), but based on the suggestion of Wu et al., (2003a), these two regions are still not processed as distribution centers and are both assigned a value of 0. |
| 5 | Actinidiaceae | 0 | 1 | 0 | 1 | 1 | 0 | 0 | 1 | 1 | 0 |  |
| 6 | Adoxaceae | 1 | 0 | 0 | 1 | 0 | 1 | 1 | 1 | 1 | 0 |  |
| 7 | Aextoxicaceae | 0 | 0 | 1 | 0 | 0 | 0 | 0 | 1 | 0 | 0 |  |
| 8 | Aizoaceae | 0 | 1 | 0 | 0 | 0 | 0 | 1 | 0 | 0 | 0 |  |
| 9 | Akaniaceae | 0 | 1 | 0 | 1 | 1 | 0 | 0 | 0 | 0 | 0 |  |
| 10 | Alismataceae | 1 | 1 | 1 | 1 | 1 | 1 | 1 | 1 | 1 | 0 |  |
| 11 | Alseuosmiaceae | 0 | 1 | 1 | 0 | 1 | 0 | 0 | 0 | 0 | 0 |  |
| 12 | Alstroemeriaceae | 0 | 1 | 1 | 0 | 0 | 0 | 0 | 1 | 1 | 0 |  |
| 13 | Altingiaceae | 1 | 1 | 0 | 1 | 0 | 0 | 0 | 0 | 1 | 0 |  |
| 14 | Alzateaceae | 0 | 1 | 0 | 0 | 0 | 0 | 0 | 1 | 1 | 0 |  |
| 15 | Amaranthaceae | 1 | 1 | 1 | 1 | 1 | 1 | 1 | 1 | 1 | 0 |  |
| 16 | Amaryllidaceae | 1 | 1 | 1 | 1 | 0 | 1 | 1 | 1 | 1 | 0 |  |
| 17 | Amborellaceae | 0 | 1 | 0 | 0 | 1 | 0 | 0 | 0 | 0 | 0 |  |
| 18 | Anacampserotaceae | 1 | 0 | 1 | 0 | 0 | 0 | 1 | 1 | 0 | 0 | This family exists in Asia in *Plants of the world* and ANGIOSPERM PHYLOGENY WEBSITE (http://www.mobot.org/mobot/research/apweb/welcome.html), but based on the number of native species in Asia, this continent is still assigned a value of 0. |
| 19 | Anacardiaceae | 0 | 1 | 0 | 1 | 1 | 0 | 1 | 1 | 1 | 0 |  |
| 20 | Ancistrocladaceae | 0 | 1 | 0 | 1 | 0 | 0 | 1 | 0 | 0 | 0 |  |
| 21 | Anisophylleaceae | 0 | 1 | 0 | 1 | 0 | 0 | 1 | 1 | 0 | 0 |  |
| 22 | Annonaceae | 0 | 1 | 0 | 1 | 1 | 0 | 1 | 1 | 1 | 0 |  |
| 23 | Aphanopetalaceae | 0 | 1 | 0 | 0 | 1 | 0 | 0 | 0 | 0 | 0 |  |
| 24 | Aphloiaceae | 0 | 1 | 0 | 0 | 0 | 0 | 1 | 0 | 0 | 0 |  |
| 25 | Apiaceae | 1 | 1 | 1 | 1 | 1 | 1 | 1 | 1 | 1 | 0 |  |
| 26 | Apocynaceae | 0 | 1 | 0 | 1 | 1 | 0 | 1 | 1 | 1 | 0 |  |
| 27 | Apodanthaceae | 1 | 1 | 1 | 1 | 1 | 0 | 1 | 1 | 1 | 0 |  |
| 28 | Aponogetonaceae | 0 | 1 | 0 | 1 | 1 | 0 | 1 | 0 | 0 | 0 |  |
| 29 | Aquifoliaceae | 0 | 1 | 0 | 1 | 0 | 0 | 0 | 1 | 1 | 0 |  |
| 30 | Araceae | 1 | 1 | 1 | 1 | 1 | 0 | 1 | 1 | 1 | 0 |  |
| 31 | Araliaceae | 0 | 1 | 0 | 1 | 1 | 0 | 1 | 1 | 1 | 0 |  |
| 32 | Araucariaceae | 0 | 1 | 0 | 1 | 1 | 0 | 0 | 1 | 0 | 0 |  |
| 33 | Arecaceae | 0 | 1 | 0 | 1 | 1 | 0 | 1 | 1 | 1 | 0 |  |
| 34 | Argophyllaceae | 0 | 1 | 1 | 0 | 1 | 0 | 0 | 0 | 0 | 0 |  |
| 35 | Aristolochiaceae | 1 | 1 | 0 | 1 | 1 | 0 | 1 | 1 | 1 | 0 |  |
| 36 | Asparagaceae | 1 | 1 | 1 | 1 | 1 | 1 | 1 | 0 | 1 | 0 |  |
| 37 | Asphodelaceae | 1 | 1 | 1 | 1 | 1 | 0 | 1 | 0 | 0 | 0 |  |
| 38 | Asteliaceae | 0 | 0 | 1 | 0 | 1 | 0 | 0 | 0 | 0 | 0 |  |
| 39 | Asteraceae | 1 | 1 | 1 | 1 | 1 | 1 | 1 | 1 | 1 | 0 |  |
| 40 | Asteropeiaceae | 0 | 1 | 0 | 0 | 0 | 0 | 1 | 0 | 0 | 0 |  |
| 41 | Atherospermataceae | 0 | 0 | 1 | 0 | 1 | 0 | 0 | 1 | 0 | 0 |  |
| 42 | Austrobaileyaceae | 0 | 1 | 0 | 0 | 1 | 0 | 0 | 0 | 0 | 0 |  |
| 43 | Balanopaceae | 0 | 1 | 0 | 0 | 1 | 0 | 0 | 0 | 0 | 0 |  |
| 44 | Balanophoraceae | 0 | 1 | 0 | 1 | 1 | 0 | 1 | 1 | 1 | 0 |  |
| 45 | Balsaminaceae | 0 | 1 | 0 | 1 | 0 | 0 | 1 | 0 | 0 | 0 |  |
| 46 | Barbeuiaceae | 0 | 1 | 0 | 0 | 0 | 0 | 1 | 0 | 0 | 0 |  |
| 47 | Barbeyaceae | 0 | 1 | 0 | 1 | 0 | 0 | 1 | 0 | 0 | 0 |  |
| 48 | Basellaceae | 0 | 1 | 0 | 0 | 0 | 0 | 1 | 1 | 1 | 0 | This family does not exist in Aisa and Australasia in *Plants of the world* and ANGIOSPERM PHYLOGENY WEBSITE (http://www.mobot.org/mobot/research/apweb/welcome.html). |
| 49 | Bataceae | 0 | 1 | 0 | 0 | 1 | 0 | 0 | 1 | 1 | 0 |  |
| 50 | Begoniaceae | 0 | 1 | 0 | 1 | 1 | 0 | 1 | 1 | 1 | 0 |  |
| 51 | Berberidaceae | 1 | 0 | 1 | 1 | 0 | 0 | 0 | 1 | 1 | 0 |  |
| 52 | Berberidopsidaceae | 0 | 0 | 1 | 0 | 1 | 0 | 0 | 1 | 0 | 0 |  |
| 53 | Betulaceae | 1 | 0 | 0 | 1 | 0 | 1 | 0 | 0 | 1 | 0 |  |
| 54 | Biebersteiniaceae | 1 | 0 | 0 | 1 | 0 | 1 | 0 | 0 | 0 | 0 |  |
| 55 | Bignoniaceae | 0 | 1 | 0 | 1 | 0 | 0 | 1 | 1 | 1 | 0 |  |
| 56 | Bixaceae | 0 | 1 | 0 | 1 | 1 | 0 | 1 | 1 | 1 | 0 |  |
| 57 | Blandfordiaceae | 0 | 0 | 1 | 0 | 1 | 0 | 0 | 0 | 0 | 0 |  |
| 58 | Bonnetiaceae | 0 | 1 | 0 | 1 | 0 | 0 | 0 | 1 | 0 | 0 |  |
| 59 | Boraginaceae | 1 | 1 | 1 | 1 | 1 | 1 | 1 | 1 | 1 | 0 |  |
| 60 | Boryaceae | 0 | 0 | 1 | 0 | 1 | 0 | 0 | 0 | 0 | 0 |  |
| 61 | Brassicaceae | 1 | 1 | 1 | 1 | 1 | 1 | 1 | 1 | 1 | 0 |  |
| 62 | Bromeliaceae | 0 | 1 | 0 | 0 | 0 | 0 | 0 | 1 | 1 | 0 |  |
| 63 | Brunelliaceae | 0 | 1 | 0 | 0 | 0 | 0 | 0 | 1 | 1 | 0 |  |
| 64 | Bruniaceae | 0 | 0 | 1 | 0 | 0 | 0 | 1 | 0 | 0 | 0 |  |
| 65 | Burmanniaceae | 0 | 1 | 0 | 1 | 1 | 0 | 1 | 1 | 1 | 0 |  |
| 66 | Burseraceae | 0 | 1 | 0 | 1 | 1 | 0 | 1 | 1 | 1 | 0 |  |
| 67 | Butomaceae | 1 | 0 | 0 | 1 | 0 | 1 | 1 | 0 | 0 | 0 |  |
| 68 | Buxaceae | 1 | 1 | 0 | 1 | 0 | 0 | 1 | 1 | 1 | 0 |  |
| 69 | Byblidaceae | 0 | 1 | 0 | 0 | 1 | 0 | 0 | 0 | 0 | 0 |  |
| 70 | Cabombaceae | 1 | 1 | 1 | 1 | 1 | 0 | 1 | 1 | 1 | 0 |  |
| 71 | Cactaceae | 0 | 1 | 0 | 0 | 0 | 0 | 0 | 1 | 1 | 0 |  |
| 72 | Calophyllaceae | 0 | 1 | 0 | 1 | 1 | 0 | 1 | 1 | 1 | 0 |  |
| 73 | Calycanthaceae | 1 | 0 | 0 | 1 | 1 | 0 | 0 | 0 | 1 | 0 |  |
| 74 | Calyceraceae | 0 | 0 | 1 | 0 | 0 | 0 | 0 | 1 | 0 | 0 |  |
| 75 | Campanulaceae | 1 | 1 | 1 | 1 | 1 | 1 | 1 | 1 | 1 | 0 |  |
| 76 | Campynemataceae | 0 | 1 | 0 | 0 | 1 | 0 | 0 | 0 | 0 | 0 |  |
| 77 | Canellaceae | 0 | 1 | 0 | 0 | 0 | 0 | 1 | 1 | 1 | 0 |  |
| 78 | Cannabaceae | 1 | 1 | 1 | 1 | 1 | 1 | 1 | 1 | 1 | 0 |  |
| 79 | Cannaceae | 0 | 1 | 0 | 0 | 0 | 0 | 0 | 1 | 1 | 0 |  |
| 80 | Capparaceae | 0 | 1 | 0 | 1 | 1 | 0 | 1 | 1 | 1 | 0 |  |
| 81 | Caprifoliaceae | 1 | 0 | 0 | 1 | 0 | 1 | 1 | 1 | 1 | 0 |  |
| 82 | Cardiopteridaceae | 0 | 1 | 0 | 1 | 1 | 0 | 1 | 1 | 0 | 0 |  |
| 83 | Caricaceae | 0 | 1 | 0 | 0 | 0 | 0 | 1 | 1 | 1 | 0 |  |
| 84 | Carlemanniaceae | 0 | 1 | 0 | 1 | 0 | 0 | 0 | 0 | 0 | 0 |  |
| 85 | Caryocaraceae | 0 | 1 | 0 | 0 | 0 | 0 | 0 | 1 | 1 | 0 |  |
| 86 | Caryophyllaceae | 1 | 1 | 1 | 1 | 0 | 1 | 1 | 1 | 1 | 0 |  |
| 87 | Casuarinaceae | 0 | 1 | 1 | 1 | 1 | 0 | 0 | 0 | 0 | 0 | This family exists in Africa but not in Northern America in *Plants of the world* and ANGIOSPERM PHYLOGENY WEBSITE (http://www.mobot.org/mobot/research/apweb/welcome.html), but based on the number of native species in Africa, this continent is still assigned a value of 0. |
| 88 | Celastraceae | 1 | 1 | 1 | 1 | 1 | 0 | 1 | 1 | 1 | 0 |  |
| 89 | Centroplacaceae | 0 | 1 | 0 | 1 | 1 | 0 | 1 | 0 | 0 | 0 |  |
| 90 | Cephalotaceae | 0 | 0 | 1 | 0 | 1 | 0 | 0 | 0 | 0 | 0 |  |
| 91 | Cephalotaxaceae | 1 | 0 | 0 | 1 | 0 | 0 | 0 | 0 | 0 | 0 |  |
| 92 | Ceratophyllaceae | 1 | 1 | 1 | 1 | 1 | 1 | 1 | 1 | 1 | 0 |  |
| 93 | Cercidiphyllaceae | 1 | 0 | 0 | 1 | 0 | 0 | 0 | 0 | 0 | 0 |  |
| 94 | Chloranthaceae | 0 | 1 | 0 | 1 | 1 | 0 | 0 | 1 | 1 | 0 |  |
| 95 | Chrysobalanaceae | 0 | 1 | 0 | 1 | 1 | 0 | 1 | 1 | 1 | 0 |  |
| 96 | Circaeasteraceae | 1 | 0 | 0 | 1 | 0 | 0 | 0 | 0 | 0 | 0 |  |
| 97 | Cistaceae | 1 | 0 | 0 | 1 | 0 | 1 | 1 | 0 | 1 | 0 |  |
| 98 | Cleomaceae | 0 | 1 | 0 | 1 | 1 | 0 | 1 | 1 | 1 | 0 |  |
| 99 | Clethraceae | 0 | 1 | 0 | 1 | 1 | 0 | 0 | 1 | 1 | 0 |  |
| 100 | Clusiaceae | 0 | 1 | 0 | 1 | 1 | 0 | 1 | 1 | 1 | 0 |  |
| 101 | Colchicaceae | 1 | 1 | 1 | 1 | 1 | 1 | 1 | 0 | 0 | 0 |  |
| 102 | Columelliaceae | 0 | 0 | 1 | 0 | 0 | 0 | 0 | 1 | 1 | 0 |  |
| 103 | Combretaceae | 0 | 1 | 0 | 1 | 1 | 0 | 1 | 1 | 1 | 0 |  |
| 104 | Commelinaceae | 0 | 1 | 0 | 1 | 1 | 0 | 1 | 1 | 1 | 0 |  |
| 105 | Connaraceae | 0 | 1 | 0 | 1 | 1 | 0 | 1 | 1 | 1 | 0 |  |
| 106 | Convolvulaceae | 1 | 1 | 1 | 1 | 1 | 0 | 1 | 1 | 1 | 0 |  |
| 107 | Coriariaceae | 1 | 0 | 1 | 1 | 1 | 1 | 1 | 1 | 1 | 0 |  |
| 108 | Cornaceae | 1 | 0 | 0 | 1 | 1 | 1 | 1 | 0 | 1 | 0 |  |
| 109 | Corsiaceae | 0 | 1 | 0 | 1 | 1 | 0 | 0 | 1 | 0 | 1 | This family exists in Asia in *Plants of the world* and ANGIOSPERM PHYLOGENY WEBSITE (http://www.mobot.org/mobot/research/apweb/welcome.html), and Asia is then assigned a value of 1 based on the number of native species in this continent. |
| 110 | Corynocarpaceae | 0 | 1 | 0 | 0 | 1 | 0 | 0 | 0 | 0 | 0 |  |
| 111 | Costaceae | 0 | 1 | 0 | 1 | 1 | 0 | 1 | 1 | 1 | 0 |  |
| 112 | Crassulaceae | 1 | 1 | 1 | 1 | 0 | 1 | 1 | 1 | 1 | 0 |  |
| 113 | Crossosomataceae | 1 | 0 | 0 | 0 | 0 | 0 | 0 | 0 | 1 | 0 |  |
| 114 | Crypteroniaceae | 0 | 1 | 0 | 1 | 1 | 0 | 0 | 0 | 0 | 0 |  |
| 115 | Ctenolophonaceae | 0 | 1 | 0 | 1 | 1 | 0 | 1 | 0 | 0 | 0 |  |
| 116 | Cucurbitaceae | 0 | 1 | 0 | 1 | 1 | 0 | 1 | 1 | 1 | 0 |  |
| 117 | Cunoniaceae | 0 | 1 | 0 | 1 | 1 | 0 | 1 | 1 | 1 | 0 |  |
| 118 | Cupressaceae | 1 | 0 | 1 | 1 | 1 | 1 | 1 | 0 | 1 | 0 |  |
| 119 | Curtisiaceae | 0 | 0 | 1 | 0 | 0 | 0 | 1 | 0 | 0 | 0 |  |
| 120 | Cycadaceae | 0 | 1 | 0 | 1 | 1 | 0 | 0 | 0 | 0 | 0 |  |
| 121 | Cyclanthaceae | 0 | 1 | 0 | 0 | 0 | 0 | 0 | 1 | 1 | 0 |  |
| 122 | Cymodoceaceae | 0 | 1 | 0 | 1 | 1 | 1 | 1 | 1 | 1 | 0 |  |
| 123 | Cynomoriaceae | 1 | 0 | 0 | 1 | 0 | 1 | 1 | 0 | 0 | 0 |  |
| 124 | Cyperaceae | 1 | 1 | 1 | 1 | 1 | 1 | 1 | 1 | 1 | 0 |  |
| 125 | Cyrillaceae | 0 | 1 | 0 | 0 | 0 | 0 | 0 | 1 | 1 | 0 |  |
| 126 | Cytinaceae | 1 | 0 | 1 | 1 | 0 | 1 | 1 | 1 | 1 | 0 |  |
| 127 | Daphniphyllaceae | 0 | 1 | 0 | 1 | 1 | 0 | 0 | 0 | 0 | 0 |  |
| 128 | Dasypogonaceae | 0 | 0 | 1 | 0 | 1 | 0 | 0 | 0 | 0 | 0 |  |
| 129 | Datiscaceae | 1 | 0 | 0 | 1 | 0 | 1 | 0 | 0 | 1 | 0 |  |
| 130 | Degeneriaceae | 0 | 1 | 0 | 0 | 1 | 0 | 0 | 0 | 0 | 0 |  |
| 131 | Diapensiaceae | 1 | 0 | 0 | 1 | 0 | 1 | 0 | 0 | 1 | 0 |  |
| 132 | Dichapetalaceae | 0 | 1 | 0 | 1 | 0 | 0 | 1 | 1 | 1 | 0 |  |
| 133 | Didiereaceae | 0 | 1 | 0 | 0 | 0 | 0 | 1 | 0 | 0 | 0 |  |
| 134 | Dilleniaceae | 0 | 1 | 0 | 1 | 1 | 0 | 0 | 1 | 1 | 0 |  |
| 135 | Dioncophyllaceae | 0 | 1 | 0 | 0 | 0 | 0 | 1 | 0 | 0 | 0 |  |
| 136 | Dioscoreaceae | 0 | 1 | 0 | 1 | 0 | 0 | 1 | 1 | 1 | 0 |  |
| 137 | Dipentodontaceae | 0 | 1 | 0 | 1 | 1 | 0 | 0 | 1 | 1 | 0 |  |
| 138 | Dipterocarpaceae | 0 | 1 | 0 | 1 | 0 | 0 | 1 | 0 | 0 | 0 |  |
| 139 | Dirachmaceae | 0 | 1 | 0 | 0 | 0 | 0 | 1 | 0 | 0 | 0 |  |
| 140 | Doryanthaceae | 0 | 0 | 1 | 0 | 1 | 0 | 0 | 0 | 0 | 0 |  |
| 141 | Droseraceae | 1 | 1 | 1 | 1 | 1 | 0 | 1 | 1 | 1 | 0 |  |
| 142 | Drosophyllaceae | 1 | 0 | 0 | 0 | 0 | 1 | 1 | 0 | 0 | 0 |  |
| 143 | Ebenaceae | 0 | 1 | 0 | 1 | 1 | 0 | 1 | 1 | 1 | 0 | This family exists in Europe in *Plants of the world*, but based on the number of native species in Europe, this continent is still assigned a value of 0. |
| 144 | Ecdeiocoleaceae | 0 | 0 | 1 | 0 | 1 | 0 | 0 | 0 | 0 | 0 |  |
| 145 | Elaeagnaceae | 1 | 0 | 0 | 1 | 0 | 0 | 0 | 0 | 1 | 0 |  |
| 146 | Elaeocarpaceae | 0 | 1 | 0 | 1 | 1 | 0 | 0 | 1 | 1 | 0 |  |
| 147 | Elatinaceae | 1 | 1 | 1 | 1 | 1 | 1 | 1 | 1 | 1 | 0 |  |
| 148 | Emblingiaceae | 0 | 0 | 1 | 0 | 1 | 0 | 0 | 0 | 0 | 0 |  |
| 149 | Ephedraceae | 1 | 0 | 1 | 1 | 0 | 1 | 1 | 1 | 1 | 0 |  |
| 150 | Ericaceae | 1 | 0 | 1 | 1 | 1 | 0 | 1 | 1 | 1 | 0 |  |
| 151 | Eriocaulaceae | 0 | 1 | 0 | 1 | 1 | 0 | 1 | 1 | 1 | 0 |  |
| 152 | Erythroxylaceae | 0 | 1 | 0 | 1 | 0 | 0 | 1 | 1 | 1 | 0 |  |
| 153 | Escalloniaceae | 0 | 1 | 1 | 1 | 1 | 0 | 0 | 1 | 0 | 0 |  |
| 154 | Eucommiaceae | 1 | 0 | 0 | 1 | 0 | 0 | 0 | 0 | 0 | 0 |  |
| 155 | Euphorbiaceae | 0 | 1 | 0 | 1 | 1 | 0 | 1 | 1 | 1 | 0 |  |
| 156 | Euphroniaceae | 0 | 1 | 0 | 0 | 0 | 0 | 0 | 1 | 0 | 0 |  |
| 157 | Eupomatiaceae | 0 | 1 | 1 | 0 | 1 | 0 | 0 | 0 | 0 | 0 |  |
| 158 | Eupteleaceae | 1 | 0 | 0 | 1 | 0 | 0 | 0 | 0 | 0 | 0 |  |
| 159 | Fabaceae | 1 | 1 | 1 | 1 | 1 | 1 | 1 | 1 | 1 | 0 |  |
| 160 | Fagaceae | 1 | 0 | 0 | 1 | 0 | 0 | 0 | 0 | 1 | 0 |  |
| 161 | Flagellariaceae | 0 | 1 | 0 | 1 | 1 | 0 | 1 | 0 | 0 | 0 |  |
| 162 | Fouquieriaceae | 1 | 0 | 0 | 0 | 0 | 0 | 0 | 0 | 1 | 0 |  |
| 163 | Francoaceae | 0 | 1 | 1 | 0 | 0 | 0 | 1 | 1 | 0 | 0 | This family does not exist in Aisa in *Plants of the world* and ANGIOSPERM PHYLOGENY WEBSITE (http://www.mobot.org/mobot/research/apweb/welcome.html). |
| 164 | Frankeniaceae | 1 | 0 | 1 | 1 | 1 | 1 | 1 | 1 | 1 | 0 |  |
| 165 | Garryaceae | 1 | 0 | 0 | 1 | 0 | 0 | 0 | 0 | 1 | 0 |  |
| 166 | Geissolomataceae | 0 | 0 | 1 | 0 | 0 | 0 | 1 | 0 | 0 | 0 |  |
| 167 | Gelsemiaceae | 0 | 1 | 0 | 1 | 0 | 0 | 1 | 1 | 1 | 0 |  |
| 168 | Gentianaceae | 1 | 1 | 1 | 1 | 1 | 1 | 1 | 1 | 1 | 0 |  |
| 169 | Geraniaceae | 1 | 0 | 1 | 1 | 1 | 1 | 1 | 1 | 1 | 0 |  |
| 170 | Gerrardinaceae | 0 | 1 | 0 | 0 | 0 | 0 | 1 | 0 | 0 | 0 |  |
| 171 | Gesneriaceae | 0 | 1 | 0 | 1 | 1 | 0 | 1 | 1 | 1 | 0 |  |
| 172 | Ginkgoaceae | 1 | 0 | 0 | 1 | 0 | 0 | 0 | 0 | 0 | 0 |  |
| 173 | Gisekiaceae | 0 | 1 | 0 | 1 | 0 | 0 | 1 | 0 | 0 | 0 |  |
| 174 | Gnetaceae | 0 | 1 | 0 | 1 | 1 | 0 | 1 | 1 | 0 | 0 |  |
| 175 | Gomortegaceae | 0 | 0 | 1 | 0 | 0 | 0 | 0 | 1 | 0 | 0 |  |
| 176 | Goodeniaceae | 0 | 1 | 0 | 0 | 1 | 0 | 0 | 0 | 0 | 0 |  |
| 177 | Goupiaceae | 0 | 1 | 0 | 0 | 0 | 0 | 0 | 1 | 1 | 0 |  |
| 178 | Griseliniaceae | 0 | 0 | 1 | 0 | 1 | 0 | 0 | 1 | 0 | 0 |  |
| 179 | Grossulariaceae | 1 | 0 | 0 | 1 | 0 | 1 | 0 | 1 | 1 | 0 |  |
| 180 | Grubbiaceae | 0 | 0 | 1 | 0 | 0 | 0 | 1 | 0 | 0 | 0 |  |
| 181 | Guamatelaceae | 0 | 1 | 0 | 0 | 0 | 0 | 0 | 0 | 1 | 0 |  |
| 182 | Gunneraceae | 0 | 1 | 0 | 0 | 1 | 0 | 0 | 1 | 1 | 0 |  |
| 183 | Gyrostemonaceae | 0 | 0 | 1 | 0 | 1 | 0 | 0 | 0 | 0 | 0 |  |
| 184 | Haemodoraceae | 0 | 0 | 1 | 0 | 1 | 0 | 1 | 1 | 0 | 0 |  |
| 185 | Halophytaceae | 0 | 0 | 1 | 0 | 0 | 0 | 0 | 1 | 0 | 0 |  |
| 186 | Haloragaceae | 1 | 1 | 1 | 1 | 1 | 0 | 1 | 1 | 1 | 0 |  |
| 187 | Hamamelidaceae | 1 | 1 | 0 | 1 | 1 | 0 | 1 | 0 | 1 | 0 |  |
| 188 | Hanguanaceae | 0 | 1 | 0 | 1 | 1 | 0 | 0 | 0 | 0 | 0 |  |
| 189 | Heliconiaceae | 0 | 1 | 0 | 0 | 0 | 0 | 0 | 1 | 1 | 0 |  |
| 190 | Helwingiaceae | 1 | 0 | 0 | 1 | 0 | 0 | 0 | 0 | 0 | 0 |  |
| 191 | Hernandiaceae | 0 | 1 | 0 | 1 | 1 | 0 | 1 | 1 | 1 | 0 |  |
| 192 | Himantandraceae | 0 | 1 | 0 | 1 | 1 | 0 | 0 | 0 | 0 | 0 | This family exists in Asia in *Plants of the world* and ANGIOSPERM PHYLOGENY WEBSITE (http://www.mobot.org/mobot/research/apweb/welcome.html), and Asia is then assigned a value of 1 based on the number of native species in this continent . |
| 193 | Huaceae | 0 | 1 | 0 | 0 | 0 | 0 | 1 | 0 | 0 | 0 |  |
| 194 | Humiriaceae | 0 | 1 | 0 | 0 | 0 | 0 | 0 | 1 | 1 | 0 |  |
| 195 | Hydatellaceae | 0 | 0 | 1 | 1 | 1 | 0 | 0 | 0 | 0 | 0 |  |
| 196 | Hydrangeaceae | 1 | 0 | 0 | 1 | 0 | 0 | 0 | 1 | 1 | 0 | This family exists in Europe in *Plants of the world* and ANGIOSPERM PHYLOGENY WEBSITE (http://www.mobot.org/mobot/research/apweb/welcome.html), but based on the number of native species in Europe, this continent is still assigned a value of 0. |
| 197 | Hydrocharitaceae | 1 | 1 | 1 | 1 | 1 | 1 | 1 | 1 | 1 | 0 |  |
| 198 | Hydroleaceae | 0 | 1 | 0 | 1 | 1 | 0 | 1 | 1 | 1 | 0 |  |
| 199 | Hydrostachyaceae | 0 | 1 | 0 | 0 | 0 | 0 | 1 | 0 | 0 | 0 |  |
| 200 | Hypericaceae | 1 | 1 | 1 | 1 | 0 | 1 | 1 | 1 | 1 | 0 |  |
| 201 | Hypoxidaceae | 0 | 1 | 1 | 1 | 1 | 0 | 1 | 0 | 1 | 0 |  |
| 202 | Icacinaceae | 0 | 1 | 0 | 1 | 1 | 0 | 1 | 1 | 0 | 0 |  |
| 203 | Iridaceae | 1 | 1 | 1 | 1 | 0 | 1 | 1 | 1 | 1 | 0 |  |
| 204 | Irvingiaceae | 0 | 1 | 0 | 1 | 0 | 0 | 1 | 0 | 0 | 0 |  |
| 205 | Iteaceae | 1 | 0 | 0 | 1 | 0 | 0 | 1 | 0 | 1 | 0 |  |
| 206 | Ixioliriaceae | 1 | 0 | 0 | 1 | 0 | 0 | 0 | 0 | 0 | 0 |  |
| 207 | Ixonanthaceae | 0 | 1 | 0 | 1 | 1 | 0 | 1 | 1 | 0 | 0 |  |
| 208 | Joinvilleaceae | 0 | 1 | 0 | 1 | 1 | 0 | 0 | 0 | 0 | 0 |  |
| 209 | Juglandaceae | 1 | 0 | 0 | 1 | 0 | 0 | 0 | 1 | 1 | 0 | This family does not exist in Africa in *Plants of the world* and ANGIOSPERM PHYLOGENY WEBSITE (http://www.mobot.org/mobot/research/apweb/welcome.html). |
| 210 | Juncaceae | 1 | 0 | 1 | 1 | 1 | 1 | 1 | 1 | 1 | 0 |  |
| 211 | Juncaginaceae | 1 | 1 | 1 | 1 | 1 | 1 | 1 | 1 | 1 | 0 |  |
| 212 | Kewaceae | 0 | 0 | 1 | 0 | 0 | 0 | 1 | 0 | 0 | 0 |  |
| 213 | Kirkiaceae | 0 | 1 | 0 | 0 | 0 | 0 | 1 | 0 | 0 | 0 |  |
| 214 | Koeberliniaceae | 0 | 1 | 0 | 0 | 0 | 0 | 0 | 1 | 1 | 0 |  |
| 215 | Krameriaceae | 0 | 1 | 0 | 0 | 0 | 0 | 0 | 1 | 1 | 0 |  |
| 216 | Lacistemataceae | 0 | 1 | 0 | 0 | 0 | 0 | 0 | 1 | 1 | 0 |  |
| 217 | Lamiaceae | 1 | 1 | 1 | 1 | 1 | 1 | 1 | 1 | 1 | 0 |  |
| 218 | Lanariaceae | 0 | 0 | 1 | 0 | 0 | 0 | 1 | 0 | 0 | 0 |  |
| 219 | Lardizabalaceae | 1 | 0 | 1 | 1 | 0 | 0 | 0 | 1 | 0 | 0 |  |
| 220 | Lauraceae | 0 | 1 | 0 | 1 | 1 | 0 | 1 | 1 | 1 | 0 |  |
| 221 | Lecythidaceae | 0 | 1 | 0 | 1 | 1 | 0 | 1 | 1 | 1 | 0 |  |
| 222 | Lentibulariaceae | 1 | 1 | 1 | 1 | 1 | 1 | 1 | 1 | 1 | 0 |  |
| 223 | Lepidobotryaceae | 0 | 1 | 0 | 0 | 0 | 0 | 1 | 1 | 1 | 0 |  |
| 224 | Liliaceae | 1 | 0 | 0 | 1 | 0 | 1 | 1 | 0 | 1 | 0 |  |
| 225 | Limeaceae | 0 | 1 | 0 | 1 | 0 | 0 | 1 | 0 | 0 | 0 |  |
| 226 | Limnanthaceae | 1 | 0 | 0 | 0 | 0 | 0 | 0 | 0 | 1 | 0 |  |
| 227 | Linaceae | 1 | 1 | 1 | 1 | 1 | 1 | 1 | 1 | 1 | 0 |  |
| 228 | Linderniaceae | 0 | 1 | 0 | 1 | 1 | 0 | 1 | 0 | 1 | 0 |  |
| 229 | Loasaceae | 0 | 1 | 0 | 0 | 0 | 0 | 0 | 1 | 1 | 0 | This family does not exist in Australasia in *Plants of the world* and ANGIOSPERM PHYLOGENY WEBSITE (http://www.mobot.org/mobot/research/apweb/welcome.html). |
| 230 | Loganiaceae | 0 | 1 | 0 | 1 | 1 | 0 | 1 | 1 | 1 | 0 |  |
| 231 | Lophiocarpaceae | 0 | 1 | 0 | 1 | 0 | 0 | 1 | 0 | 0 | 0 |  |
| 232 | Lophopyxidaceae | 0 | 1 | 0 | 1 | 1 | 0 | 0 | 0 | 0 | 0 |  |
| 233 | Loranthaceae | 0 | 1 | 0 | 1 | 1 | 0 | 1 | 1 | 1 | 0 |  |
| 234 | Lowiaceae | 0 | 1 | 0 | 1 | 0 | 0 | 0 | 0 | 0 | 0 |  |
| 235 | Lythraceae | 1 | 1 | 1 | 1 | 1 | 0 | 1 | 1 | 1 | 0 |  |
| 236 | Macarthuriaceae | 0 | 0 | 1 | 0 | 1 | 0 | 0 | 0 | 0 | 0 |  |
| 237 | Magnoliaceae | 1 | 1 | 0 | 1 | 0 | 0 | 0 | 1 | 1 | 0 |  |
| 238 | Malpighiaceae | 0 | 1 | 0 | 1 | 0 | 0 | 1 | 1 | 1 | 0 |  |
| 239 | Malvaceae | 0 | 1 | 0 | 1 | 1 | 0 | 1 | 1 | 1 | 0 |  |
| 240 | Marantaceae | 0 | 1 | 0 | 1 | 0 | 0 | 1 | 1 | 1 | 0 |  |
| 241 | Marcgraviaceae | 0 | 1 | 0 | 0 | 0 | 0 | 0 | 1 | 1 | 0 |  |
| 242 | Martyniaceae | 0 | 1 | 0 | 0 | 0 | 0 | 0 | 1 | 1 | 0 |  |
| 243 | Maundiaceae | 0 | 0 | 1 | 0 | 1 | 0 | 0 | 0 | 0 | 0 |  |
| 244 | Mayacaceae | 0 | 1 | 0 | 0 | 0 | 0 | 1 | 1 | 1 | 0 |  |
| 245 | Mazaceae | 1 | 0 | 1 | 1 | 1 | 0 | 0 | 0 | 0 | 0 |  |
| 246 | Melanthiaceae | 1 | 0 | 0 | 1 | 0 | 0 | 0 | 0 | 1 | 0 |  |
| 247 | Melastomataceae | 0 | 1 | 0 | 1 | 1 | 0 | 1 | 1 | 1 | 0 |  |
| 248 | Meliaceae | 0 | 1 | 0 | 1 | 1 | 0 | 1 | 1 | 1 | 0 |  |
| 249 | Menispermaceae | 0 | 1 | 0 | 1 | 1 | 0 | 1 | 1 | 1 | 0 |  |
| 250 | Menyanthaceae | 1 | 1 | 1 | 1 | 1 | 0 | 1 | 1 | 1 | 0 |  |
| 251 | Metteniusaceae | 0 | 1 | 0 | 1 | 1 | 0 | 1 | 1 | 1 | 0 |  |
| 252 | Microteaceae | 0 | 1 | 0 | 0 | 0 | 0 | 0 | 1 | 1 | 0 |  |
| 253 | Misodendraceae | 0 | 0 | 1 | 0 | 0 | 0 | 0 | 1 | 0 | 0 |  |
| 254 | Mitrastemonaceae | 0 | 1 | 0 | 1 | 1 | 0 | 0 | 1 | 1 | 0 |  |
| 255 | Molluginaceae | 0 | 1 | 0 | 1 | 1 | 0 | 1 | 1 | 1 | 0 |  |
| 256 | Monimiaceae | 0 | 1 | 0 | 1 | 1 | 0 | 1 | 1 | 1 | 0 |  |
| 257 | Montiaceae | 1 | 0 | 1 | 1 | 1 | 0 | 0 | 1 | 1 | 0 |  |
| 258 | Montiniaceae | 0 | 1 | 0 | 0 | 0 | 0 | 1 | 0 | 0 | 0 |  |
| 259 | Moraceae | 1 | 1 | 1 | 1 | 1 | 0 | 1 | 1 | 1 | 0 |  |
| 260 | Moringaceae | 0 | 1 | 0 | 1 | 0 | 0 | 1 | 0 | 0 | 0 |  |
| 261 | Muntingiaceae | 0 | 1 | 0 | 0 | 0 | 0 | 0 | 1 | 1 | 0 |  |
| 262 | Musaceae | 0 | 1 | 0 | 1 | 1 | 0 | 1 | 0 | 0 | 0 |  |
| 263 | Myodocarpaceae | 0 | 1 | 0 | 1 | 1 | 0 | 0 | 0 | 0 | 0 |  |
| 264 | Myricaceae | 1 | 1 | 1 | 1 | 1 | 1 | 1 | 1 | 1 | 0 |  |
| 265 | Myristicaceae | 0 | 1 | 0 | 1 | 1 | 0 | 1 | 1 | 1 | 0 |  |
| 266 | Myrothamnaceae | 0 | 1 | 0 | 0 | 0 | 0 | 1 | 0 | 0 | 0 |  |
| 267 | Myrtaceae | 0 | 1 | 1 | 1 | 1 | 0 | 1 | 1 | 1 | 0 |  |
| 268 | Nartheciaceae | 1 | 0 | 0 | 1 | 0 | 1 | 0 | 1 | 1 | 0 |  |
| 269 | Nelumbonaceae | 1 | 0 | 0 | 1 | 1 | 0 | 0 | 0 | 1 | 0 | This family does not exist in Europe and Southern America in *Plants of the world* and ANGIOSPERM PHYLOGENY WEBSITE (http://www.mobot.org/mobot/research/apweb/welcome.html). |
| 270 | Nepenthaceae | 0 | 1 | 0 | 1 | 1 | 0 | 0 | 0 | 0 | 0 |  |
| 271 | Neuradaceae | 1 | 0 | 1 | 1 | 0 | 0 | 1 | 0 | 0 | 0 |  |
| 272 | Nitrariaceae | 1 | 0 | 1 | 1 | 1 | 1 | 1 | 0 | 1 | 0 |  |
| 273 | Nothofagaceae | 0 | 0 | 1 | 0 | 1 | 0 | 0 | 1 | 0 | 0 |  |
| 274 | Nyctaginaceae | 0 | 1 | 0 | 1 | 1 | 0 | 1 | 1 | 1 | 0 |  |
| 275 | Nymphaeaceae | 1 | 1 | 1 | 1 | 1 | 1 | 1 | 1 | 1 | 0 |  |
| 276 | Nyssaceae | 1 | 1 | 0 | 1 | 0 | 0 | 0 | 0 | 1 | 0 |  |
| 277 | Ochnaceae | 0 | 1 | 0 | 1 | 0 | 0 | 1 | 1 | 1 | 0 |  |
| 278 | Olacaceae | 0 | 1 | 0 | 1 | 1 | 0 | 1 | 1 | 1 | 0 |  |
| 279 | Oleaceae | 1 | 1 | 1 | 1 | 1 | 0 | 1 | 1 | 1 | 0 |  |
| 280 | Onagraceae | 1 | 1 | 1 | 1 | 1 | 1 | 1 | 1 | 1 | 0 |  |
| 281 | Oncothecaceae | 0 | 1 | 0 | 0 | 1 | 0 | 0 | 0 | 0 | 0 |  |
| 282 | Opiliaceae | 0 | 1 | 0 | 1 | 1 | 0 | 1 | 1 | 1 | 0 |  |
| 283 | Orchidaceae | 1 | 1 | 1 | 1 | 1 | 0 | 1 | 1 | 1 | 0 |  |
| 284 | Orobanchaceae | 1 | 1 | 1 | 1 | 0 | 1 | 1 | 1 | 1 | 0 |  |
| 285 | Oxalidaceae | 1 | 1 | 1 | 1 | 0 | 0 | 1 | 1 | 1 | 0 |  |
| 286 | Paeoniaceae | 1 | 0 | 0 | 1 | 0 | 1 | 1 | 0 | 1 | 0 |  |
| 287 | Pandaceae | 0 | 1 | 0 | 1 | 1 | 0 | 1 | 0 | 0 | 0 |  |
| 288 | Pandanaceae | 0 | 1 | 0 | 1 | 1 | 0 | 1 | 0 | 0 | 0 |  |
| 289 | Papaveraceae | 1 | 0 | 0 | 1 | 0 | 1 | 1 | 0 | 1 | 0 | This family does not exist in Australasia in *Plants of the world* and ANGIOSPERM PHYLOGENY WEBSITE (http://www.mobot.org/mobot/research/apweb/welcome.html). |
| 290 | Paracryphiaceae | 0 | 1 | 0 | 1 | 1 | 0 | 0 | 0 | 0 | 0 |  |
| 291 | Passifloraceae | 0 | 1 | 0 | 1 | 0 | 0 | 1 | 1 | 1 | 0 |  |
| 292 | Paulowniaceae | 1 | 0 | 0 | 1 | 0 | 0 | 0 | 0 | 0 | 0 |  |
| 293 | Pedaliaceae | 0 | 1 | 0 | 1 | 1 | 0 | 1 | 0 | 0 | 0 |  |
| 294 | Penaeaceae | 0 | 0 | 1 | 0 | 0 | 0 | 1 | 0 | 0 | 0 |  |
| 295 | Pennantiaceae | 0 | 0 | 1 | 0 | 1 | 0 | 0 | 0 | 0 | 0 |  |
| 296 | Pentadiplandraceae | 0 | 1 | 0 | 0 | 0 | 0 | 1 | 0 | 0 | 0 |  |
| 297 | Pentaphragmataceae | 0 | 1 | 0 | 1 | 0 | 0 | 0 | 0 | 0 | 0 |  |
| 298 | Pentaphylacaceae | 0 | 1 | 0 | 1 | 1 | 0 | 0 | 1 | 1 | 0 |  |
| 299 | Penthoraceae | 1 | 0 | 0 | 1 | 0 | 0 | 0 | 0 | 1 | 0 |  |
| 300 | Peraceae | 0 | 1 | 0 | 1 | 0 | 0 | 1 | 1 | 1 | 0 |  |
| 301 | Peridiscaceae | 0 | 1 | 0 | 0 | 0 | 0 | 1 | 1 | 0 | 0 |  |
| 302 | Petenaeaceae | 0 | 1 | 0 | 0 | 0 | 0 | 0 | 0 | 1 | 0 |  |
| 303 | Petermanniaceae | 0 | 0 | 1 | 0 | 1 | 0 | 0 | 0 | 0 | 0 |  |
| 304 | Petiveriaceae | 0 | 1 | 0 | 0 | 1 | 0 | 0 | 1 | 1 | 0 |  |
| 305 | Petrosaviaceae | 0 | 1 | 0 | 1 | 0 | 0 | 0 | 0 | 0 | 0 |  |
| 306 | Phellinaceae | 0 | 1 | 0 | 0 | 1 | 0 | 0 | 0 | 0 | 0 |  |
| 307 | Philesiaceae | 0 | 0 | 1 | 0 | 0 | 0 | 0 | 1 | 0 | 0 |  |
| 308 | Philydraceae | 0 | 1 | 0 | 1 | 1 | 0 | 0 | 0 | 0 | 0 |  |
| 309 | Phrymaceae | 1 | 1 | 1 | 1 | 1 | 0 | 0 | 1 | 1 | 0 |  |
| 310 | Phyllanthaceae | 0 | 1 | 0 | 1 | 1 | 0 | 1 | 1 | 1 | 0 |  |
| 311 | Phyllonomaceae | 0 | 1 | 0 | 0 | 0 | 0 | 0 | 1 | 1 | 0 |  |
| 312 | Physenaceae | 0 | 1 | 0 | 0 | 0 | 0 | 1 | 0 | 0 | 0 |  |
| 313 | Phytolaccaceae | 0 | 1 | 0 | 1 | 0 | 0 | 1 | 1 | 1 | 0 | This family does not exist in Australasia in *Plants of the world* and ANGIOSPERM PHYLOGENY WEBSITE (http://www.mobot.org/mobot/research/apweb/welcome.html). |
| 314 | Picramniaceae | 0 | 1 | 0 | 0 | 0 | 0 | 0 | 1 | 1 | 0 |  |
| 315 | Picrodendraceae | 0 | 1 | 0 | 0 | 1 | 0 | 1 | 1 | 1 | 0 |  |
| 316 | Pinaceae | 1 | 0 | 0 | 1 | 0 | 1 | 0 | 0 | 1 | 0 |  |
| 317 | Piperaceae | 0 | 1 | 0 | 1 | 1 | 0 | 0 | 1 | 1 | 0 |  |
| 318 | Pittosporaceae | 0 | 1 | 0 | 1 | 1 | 0 | 1 | 0 | 0 | 0 |  |
| 319 | Plantaginaceae | 1 | 1 | 1 | 1 | 1 | 1 | 1 | 1 | 1 | 0 |  |
| 320 | Platanaceae | 1 | 0 | 0 | 1 | 0 | 1 | 0 | 0 | 1 | 0 |  |
| 321 | Plocospermataceae | 0 | 1 | 0 | 0 | 0 | 0 | 0 | 0 | 1 | 0 |  |
| 322 | Plumbaginaceae | 1 | 1 | 1 | 1 | 0 | 1 | 1 | 0 | 0 | 0 |  |
| 323 | Poaceae | 1 | 1 | 1 | 1 | 1 | 1 | 1 | 1 | 1 | 0 |  |
| 324 | Podocarpaceae | 0 | 1 | 0 | 1 | 1 | 0 | 1 | 1 | 1 | 0 |  |
| 325 | Podostemaceae | 0 | 1 | 0 | 1 | 0 | 0 | 1 | 1 | 1 | 0 |  |
| 326 | Polemoniaceae | 1 | 0 | 0 | 1 | 0 | 0 | 0 | 1 | 1 | 0 |  |
| 327 | Polygalaceae | 1 | 1 | 1 | 1 | 1 | 1 | 1 | 1 | 1 | 0 |  |
| 328 | Polygonaceae | 1 | 1 | 1 | 1 | 0 | 1 | 1 | 1 | 1 | 0 |  |
| 329 | Pontederiaceae | 0 | 1 | 0 | 1 | 1 | 0 | 1 | 1 | 1 | 0 |  |
| 330 | Portulacaceae | 1 | 1 | 1 | 1 | 1 | 1 | 1 | 1 | 1 | 0 |  |
| 331 | Posidoniaceae | 0 | 0 | 1 | 1 | 1 | 1 | 1 | 0 | 0 | 0 |  |
| 332 | Potamogetonaceae | 1 | 1 | 1 | 1 | 1 | 1 | 1 | 1 | 1 | 0 |  |
| 333 | Primulaceae | 1 | 1 | 1 | 1 | 1 | 1 | 1 | 1 | 1 | 0 |  |
| 334 | Proteaceae | 0 | 1 | 0 | 0 | 1 | 0 | 1 | 1 | 0 | 0 |  |
| 335 | Putranjivaceae | 0 | 1 | 0 | 1 | 1 | 0 | 1 | 1 | 1 | 0 |  |
| 336 | Quillajaceae | 0 | 0 | 1 | 0 | 0 | 0 | 0 | 1 | 0 | 0 |  |
| 337 | Rafflesiaceae | 0 | 1 | 0 | 1 | 0 | 0 | 0 | 0 | 0 | 0 |  |
| 338 | Ranunculaceae | 1 | 1 | 1 | 1 | 1 | 1 | 1 | 0 | 1 | 0 |  |
| 339 | Rapateaceae | 0 | 1 | 0 | 0 | 0 | 0 | 0 | 1 | 0 | 0 |  |
| 340 | Resedaceae | 1 | 0 | 0 | 1 | 0 | 1 | 1 | 0 | 1 | 0 |  |
| 341 | Restionaceae | 0 | 0 | 1 | 0 | 1 | 0 | 1 | 0 | 0 | 0 |  |
| 342 | Rhabdodendraceae | 0 | 1 | 0 | 0 | 0 | 0 | 0 | 1 | 0 | 0 |  |
| 343 | Rhamnaceae | 1 | 1 | 1 | 1 | 1 | 0 | 1 | 1 | 1 | 0 |  |
| 344 | Rhizophoraceae | 0 | 1 | 0 | 1 | 1 | 0 | 1 | 1 | 1 | 0 |  |
| 345 | Ripogonaceae | 0 | 1 | 0 | 0 | 1 | 0 | 0 | 0 | 0 | 0 |  |
| 346 | Roridulaceae | 0 | 0 | 1 | 0 | 0 | 0 | 1 | 0 | 0 | 0 |  |
| 347 | Rosaceae | 1 | 1 | 1 | 1 | 0 | 1 | 1 | 1 | 1 | 0 |  |
| 348 | Rousseaceae | 0 | 1 | 0 | 0 | 1 | 0 | 1 | 0 | 0 | 0 |  |
| 349 | Rubiaceae | 1 | 1 | 1 | 1 | 1 | 0 | 1 | 1 | 1 | 0 |  |
| 350 | Ruppiaceae | 1 | 1 | 1 | 1 | 1 | 1 | 1 | 1 | 1 | 1 |  |
| 351 | Rutaceae | 0 | 1 | 0 | 1 | 1 | 0 | 1 | 1 | 1 | 0 |  |
| 352 | Sabiaceae | 0 | 1 | 0 | 1 | 0 | 0 | 0 | 1 | 1 | 0 |  |
| 353 | Salicaceae | 1 | 1 | 1 | 1 | 1 | 1 | 1 | 1 | 1 | 0 |  |
| 354 | Salvadoraceae | 0 | 1 | 0 | 1 | 0 | 0 | 1 | 0 | 0 | 0 | This family does not exist in Australasia in *Plants of the world* and ANGIOSPERM PHYLOGENY WEBSITE (http://www.mobot.org/mobot/research/apweb/welcome.html). |
| 355 | Santalaceae | 1 | 1 | 1 | 1 | 1 | 0 | 1 | 1 | 1 | 0 |  |
| 356 | Sapindaceae | 0 | 1 | 0 | 1 | 1 | 0 | 1 | 1 | 1 | 0 |  |
| 357 | Sapotaceae | 0 | 1 | 0 | 1 | 1 | 0 | 1 | 1 | 1 | 0 |  |
| 358 | Sarcobataceae | 1 | 0 | 0 | 0 | 0 | 0 | 0 | 0 | 1 | 0 |  |
| 359 | Sarcolaenaceae | 0 | 1 | 0 | 0 | 0 | 0 | 1 | 0 | 0 | 0 |  |
| 360 | Sarraceniaceae | 1 | 1 | 0 | 0 | 0 | 0 | 0 | 1 | 1 | 0 |  |
| 361 | Saururaceae | 1 | 0 | 0 | 1 | 0 | 0 | 0 | 0 | 1 | 0 |  |
| 362 | Saxifragaceae | 1 | 0 | 0 | 1 | 0 | 1 | 0 | 0 | 1 | 0 |  |
| 363 | Scheuchzeriaceae | 1 | 0 | 0 | 1 | 0 | 1 | 0 | 0 | 1 | 0 |  |
| 364 | Schisandraceae | 1 | 0 | 0 | 1 | 0 | 0 | 0 | 0 | 1 | 0 |  |
| 365 | Schlegeliaceae | 0 | 1 | 0 | 0 | 0 | 0 | 0 | 1 | 1 | 0 |  |
| 366 | Schoepfiaceae | 0 | 1 | 1 | 1 | 0 | 0 | 0 | 1 | 1 | 0 |  |
| 367 | Sciadopityaceae | 1 | 0 | 0 | 1 | 0 | 0 | 0 | 0 | 0 | 0 |  |
| 368 | Scrophulariaceae | 1 | 1 | 1 | 1 | 1 | 1 | 1 | 1 | 0 | 0 |  |
| 369 | Setchellanthaceae | 1 | 0 | 0 | 0 | 0 | 0 | 0 | 0 | 1 | 0 |  |
| 370 | Simaroubaceae | 0 | 1 | 0 | 1 | 1 | 0 | 1 | 1 | 1 | 0 |  |
| 371 | Simmondsiaceae | 1 | 0 | 0 | 0 | 0 | 0 | 0 | 0 | 1 | 0 |  |
| 372 | Siparunaceae | 0 | 1 | 0 | 0 | 0 | 0 | 0 | 1 | 1 | 0 |  |
| 373 | Sladeniaceae | 0 | 1 | 0 | 1 | 0 | 0 | 1 | 0 | 0 | 0 |  |
| 374 | Smilacaceae | 0 | 1 | 0 | 1 | 1 | 0 | 0 | 1 | 1 | 0 |  |
| 375 | Solanaceae | 1 | 1 | 1 | 1 | 1 | 0 | 1 | 1 | 1 | 0 |  |
| 376 | Sphaerosepalaceae | 0 | 1 | 0 | 0 | 0 | 0 | 1 | 0 | 0 | 0 |  |
| 377 | Sphenocleaceae | 0 | 1 | 0 | 1 | 1 | 0 | 1 | 0 | 0 | 0 |  |
| 378 | Stachyuraceae | 1 | 0 | 0 | 1 | 0 | 0 | 0 | 0 | 0 | 0 |  |
| 379 | Staphyleaceae | 0 | 1 | 0 | 1 | 1 | 0 | 0 | 1 | 1 | 0 |  |
| 380 | Stegnospermataceae | 1 | 1 | 0 | 0 | 0 | 0 | 0 | 0 | 1 | 0 |  |
| 381 | Stemonaceae | 0 | 1 | 0 | 1 | 1 | 0 | 0 | 0 | 0 | 0 | This family does not exist in Southern America in *Plants of the world* and ANGIOSPERM PHYLOGENY WEBSITE (http://www.mobot.org/mobot/research/apweb/welcome.html). |
| 382 | Stemonuraceae | 0 | 1 | 0 | 1 | 1 | 0 | 1 | 0 | 0 | 0 |  |
| 383 | Stilbaceae | 0 | 0 | 1 | 1 | 0 | 0 | 1 | 0 | 0 | 0 |  |
| 384 | Strasburgeriaceae | 0 | 1 | 1 | 0 | 1 | 0 | 0 | 0 | 0 | 0 |  |
| 385 | Strelitziaceae | 0 | 1 | 1 | 0 | 0 | 0 | 1 | 1 | 0 | 0 |  |
| 386 | Stylidiaceae | 0 | 0 | 1 | 0 | 1 | 0 | 0 | 0 | 0 | 0 |  |
| 387 | Styracaceae | 0 | 1 | 0 | 1 | 0 | 0 | 0 | 1 | 1 | 0 |  |
| 388 | Surianaceae | 0 | 1 | 0 | 1 | 1 | 0 | 1 | 1 | 1 | 0 |  |
| 389 | Symplocaceae | 0 | 1 | 0 | 1 | 1 | 0 | 0 | 1 | 1 | 0 |  |
| 390 | Talinaceae | 0 | 1 | 0 | 0 | 0 | 0 | 1 | 1 | 1 | 0 | This family does not exist in Aisa in *Plants of the world* and ANGIOSPERM PHYLOGENY WEBSITE (http://www.mobot.org/mobot/research/apweb/welcome.html). |
| 391 | Tamaricaceae | 1 | 0 | 0 | 1 | 0 | 1 | 1 | 0 | 0 | 0 |  |
| 392 | Tapisciaceae | 0 | 1 | 0 | 1 | 0 | 0 | 0 | 1 | 1 | 0 |  |
| 393 | Taxaceae | 1 | 0 | 0 | 1 | 1 | 1 | 1 | 0 | 1 | 0 |  |
| 394 | Tecophilaeaceae | 0 | 0 | 1 | 0 | 0 | 0 | 1 | 1 | 1 | 0 |  |
| 395 | Tetracarpaeaceae | 0 | 0 | 1 | 0 | 1 | 0 | 0 | 0 | 0 | 0 |  |
| 396 | Tetrachondraceae | 1 | 1 | 1 | 0 | 1 | 0 | 0 | 1 | 1 | 0 |  |
| 397 | Tetramelaceae | 0 | 1 | 0 | 1 | 1 | 0 | 0 | 0 | 0 | 0 |  |
| 398 | Tetrameristaceae | 0 | 1 | 0 | 1 | 0 | 0 | 0 | 1 | 1 | 0 |  |
| 399 | Theaceae | 0 | 1 | 0 | 1 | 0 | 0 | 0 | 0 | 1 | 0 |  |
| 400 | Thomandersiaceae | 0 | 1 | 0 | 0 | 0 | 0 | 1 | 0 | 0 | 0 |  |
| 401 | Thurniaceae | 0 | 1 | 1 | 0 | 0 | 0 | 1 | 1 | 0 | 0 |  |
| 402 | Thymelaeaceae | 1 | 1 | 1 | 1 | 1 | 1 | 1 | 1 | 1 | 0 |  |
| 403 | Ticodendraceae | 0 | 1 | 0 | 0 | 0 | 0 | 0 | 0 | 1 | 0 |  |
| 404 | Tiganophytaceae | 0 | 0 | 1 | 0 | 0 | 0 | 1 | 0 | 0 | 0 |  |
| 405 | Tofieldiaceae | 1 | 0 | 0 | 1 | 0 | 1 | 0 | 1 | 1 | 0 |  |
| 406 | Torricelliaceae | 0 | 1 | 0 | 1 | 0 | 0 | 1 | 0 | 0 | 0 |  |
| 407 | Tovariaceae | 0 | 1 | 0 | 0 | 0 | 0 | 0 | 1 | 1 | 0 |  |
| 408 | Trigoniaceae | 0 | 1 | 0 | 0 | 0 | 0 | 0 | 1 | 1 | 0 |  |
| 409 | Trimeniaceae | 0 | 1 | 0 | 1 | 1 | 0 | 0 | 0 | 0 | 0 | This family exists in Asia in *Plants of the world* and ANGIOSPERM PHYLOGENY WEBSITE (http://www.mobot.org/mobot/research/apweb/welcome.html), and Asia is then assigned a value of 1 based on the number of native species in this continent. |
| 410 | Triuridaceae | 0 | 1 | 0 | 1 | 1 | 0 | 1 | 1 | 1 | 0 |  |
| 411 | Trochodendraceae | 1 | 0 | 0 | 1 | 0 | 0 | 0 | 0 | 0 | 0 |  |
| 412 | Tropaeolaceae | 0 | 0 | 1 | 0 | 0 | 0 | 0 | 1 | 0 | 0 |  |
| 413 | Typhaceae | 1 | 1 | 1 | 1 | 1 | 1 | 1 | 1 | 1 | 0 |  |
| 414 | Ulmaceae | 1 | 1 | 0 | 1 | 0 | 1 | 0 | 1 | 1 | 0 |  |
| 415 | Urticaceae | 0 | 1 | 0 | 1 | 1 | 0 | 1 | 1 | 1 | 0 |  |
| 416 | Vahliaceae | 0 | 1 | 0 | 1 | 0 | 0 | 1 | 0 | 0 | 0 |  |
| 417 | Velloziaceae | 0 | 1 | 0 | 0 | 0 | 0 | 1 | 1 | 0 | 0 |  |
| 418 | Verbenaceae | 1 | 1 | 1 | 0 | 0 | 0 | 1 | 1 | 1 | 0 |  |
| 419 | Violaceae | 1 | 1 | 1 | 1 | 1 | 1 | 1 | 1 | 1 | 0 |  |
| 420 | Vitaceae | 0 | 1 | 0 | 1 | 1 | 0 | 1 | 1 | 1 | 0 |  |
| 421 | Vochysiaceae | 0 | 1 | 0 | 0 | 0 | 0 | 0 | 1 | 1 | 0 |  |
| 422 | Welwitschiaceae | 0 | 1 | 0 | 0 | 0 | 0 | 1 | 0 | 0 | 0 |  |
| 423 | Winteraceae | 0 | 1 | 0 | 0 | 1 | 0 | 0 | 1 | 0 | 0 |  |
| 424 | Xeronemataceae | 0 | 1 | 1 | 0 | 1 | 0 | 0 | 0 | 0 | 0 |  |
| 425 | Xyridaceae | 0 | 1 | 0 | 1 | 1 | 0 | 1 | 1 | 1 | 0 |  |
| 426 | Zamiaceae | 0 | 1 | 0 | 0 | 1 | 0 | 1 | 1 | 1 | 0 |  |
| 427 | Zingiberaceae | 0 | 1 | 0 | 1 | 1 | 0 | 1 | 0 | 0 | 0 |  |
| 428 | Zosteraceae | 1 | 0 | 1 | 1 | 1 | 1 | 1 | 1 | 1 | 0 |  |
| 429 | Zygophyllaceae | 1 | 1 | 1 | 1 | 1 | 0 | 1 | 1 | 1 | 0 |  |

**Table S9.** **Percentage of species number of 429 spermatophyte families on seven continents to the total species number of corresponding families**

| **Number** | **Family** | **Asia (namely, Asia-Temperate and Asia-Tropical continents but not including Papuasia region)** | **Australasia (namely, Australasia and Pacific continents, and Papuasia region)** | **Europe (namely, Europe continent)** | **Africa (namely, Africa continent)** | **Southern America (namely, Southern America continent but not including Central America and Caribbean regions)** | **Northern America (namely, Northern America continent, and Central America and Caribbean regions)** | **Antarctica (namely, Antarctica continent)** |
| --- | --- | --- | --- | --- | --- | --- | --- | --- |
| 1 | Acanthaceae | 29% | 3% | 0% | 35% | 23% | 13% | 0% |
| 2 | Achariaceae | 42% | 6% | 0% | 39% | 14% | 5% | 0% |
| 3 | Achatocarpaceae | 0% | 0% | 0% | 0% | 70% | 50% | 0% |
| 4 | Acoraceae | 100% | 0% | 0% | 0% | 0% | 50% | 0% |
| 5 | Actinidiaceae | 64% | 16% | 0% | 0% | 14% | 6% | 0% |
| 6 | Adoxaceae | 57% | 2% | 4% | 5% | 16% | 26% | 0% |
| 7 | Aextoxicaceae | 0% | 0% | 0% | 0% | 100% | 0% | 0% |
| 8 | Aizoaceae | 1% | 3% | 0% | 96% | 1% | 0% | 0% |
| 9 | Akaniaceae | 50% | 50% | 0% | 0% | 0% | 0% | 0% |
| 10 | Alismataceae | 26% | 5% | 14% | 16% | 40% | 46% | 0% |
| 11 | Alseuosmiaceae | 0% | 100% | 0% | 0% | 0% | 0% | 0% |
| 12 | Alstroemeriaceae | 0% | 1% | 0% | 0% | 96% | 5% | 0% |
| 13 | Altingiaceae | 93% | 0% | 0% | 0% | 0% | 7% | 0% |
| 14 | Alzateaceae | 0% | 0% | 0% | 0% | 100% | 100% | 0% |
| 15 | Amaranthaceae | 31% | 23% | 8% | 21% | 18% | 14% | 0% |
| 16 | Amaryllidaceae | 38% | 1% | 12% | 19% | 26% | 10% | 0% |
| 17 | Amborellaceae | 0% | 100% | 0% | 0% | 0% | 0% | 0% |
| 18 | Anacampserotaceae | 0% | 2% | 0% | 90% | 5% | 3% | 0% |
| 19 | Anacardiaceae | 38% | 8% | 1% | 35% | 13% | 12% | 0% |
| 20 | Ancistrocladaceae | 30% | 0% | 0% | 70% | 0% | 0% | 0% |
| 21 | Anisophylleaceae | 44% | 0% | 0% | 50% | 6% | 0% | 0% |
| 22 | Annonaceae | 40% | 7% | 0% | 20% | 27% | 9% | 0% |
| 23 | Aphanopetalaceae | 0% | 100% | 0% | 0% | 0% | 0% | 0% |
| 24 | Aphloiaceae | 0% | 0% | 0% | 100% | 0% | 0% | 0% |
| 25 | Apiaceae | 54% | 6% | 14% | 15% | 7% | 14% | 0% |
| 26 | Apocynaceae | 28% | 10% | 0% | 29% | 24% | 13% | 0% |
| 27 | Apodanthaceae | 8% | 25% | 0% | 8% | 33% | 42% | 0% |
| 28 | Aponogetonaceae | 23% | 22% | 0% | 55% | 0% | 0% | 0% |
| 29 | Aquifoliaceae | 51% | 2% | 0% | 1% | 34% | 14% | 0% |
| 30 | Araceae | 32% | 4% | 1% | 4% | 49% | 18% | 0% |
| 31 | Araliaceae | 35% | 25% | 0% | 8% | 26% | 8% | 0% |
| 32 | Araucariaceae | 16% | 79% | 0% | 0% | 5% | 0% | 0% |
| 33 | Arecaceae | 39% | 19% | 0% | 12% | 20% | 15% | 0% |
| 34 | Argophyllaceae | 0% | 100% | 0% | 0% | 0% | 0% | 0% |
| 35 | Aristolochiaceae | 47% | 5% | 3% | 4% | 25% | 21% | 0% |
| 36 | Asparagaceae | 32% | 6% | 5% | 40% | 2% | 19% | 0% |
| 37 | Asphodelaceae | 12% | 12% | 2% | 76% | 0% | 0% | 0% |
| 38 | Asteliaceae | 0% | 95% | 0% | 3% | 3% | 0% | 3% |
| 39 | Asteraceae | 23% | 5% | 24% | 16% | 20% | 17% | 0% |
| 40 | Asteropeiaceae | 0% | 0% | 0% | 100% | 0% | 0% | 0% |
| 41 | Atherospermataceae | 0% | 95% | 0% | 0% | 5% | 0% | 0% |
| 42 | Austrobaileyaceae | 0% | 100% | 0% | 0% | 0% | 0% | 0% |
| 43 | Balanopaceae | 0% | 100% | 0% | 0% | 0% | 0% | 0% |
| 44 | Balanophoraceae | 44% | 12% | 0% | 15% | 32% | 10% | 0% |
| 45 | Balsaminaceae | 69% | 1% | 0% | 30% | 0% | 1% | 0% |
| 46 | Barbeuiaceae | 0% | 0% | 0% | 100% | 0% | 0% | 0% |
| 47 | Barbeyaceae | 100% | 0% | 0% | 100% | 0% | 0% | 0% |
| 48 | Basellaceae | 5% | 5% | 0% | 21% | 74% | 11% | 0% |
| 49 | Bataceae | 0% | 50% | 0% | 0% | 50% | 50% | 0% |
| 50 | Begoniaceae | 54% | 5% | 0% | 9% | 23% | 11% | 0% |
| 51 | Berberidaceae | 71% | 0% | 2% | 1% | 21% | 8% | 0% |
| 52 | Berberidopsidaceae | 0% | 67% | 0% | 0% | 33% | 0% | 0% |
| 53 | Betulaceae | 83% | 0% | 12% | 1% | 1% | 16% | 0% |
| 54 | Biebersteiniaceae | 100% | 0% | 50% | 0% | 0% | 0% | 0% |
| 55 | Bignoniaceae | 9% | 3% | 0% | 13% | 61% | 23% | 0% |
| 56 | Bixaceae | 4% | 15% | 0% | 27% | 46% | 31% | 0% |
| 57 | Blandfordiaceae | 0% | 100% | 0% | 0% | 0% | 0% | 0% |
| 58 | Bonnetiaceae | 8% | 3% | 0% | 0% | 89% | 3% | 0% |
| 59 | Boraginaceae | 39% | 6% | 9% | 12% | 15% | 30% | 0% |
| 60 | Boryaceae | 0% | 100% | 0% | 0% | 0% | 0% | 0% |
| 61 | Brassicaceae | 46% | 5% | 20% | 13% | 10% | 20% | 0% |
| 62 | Bromeliaceae | 0% | 0% | 0% | 0% | 83% | 22% | 0% |
| 63 | Brunelliaceae | 0% | 0% | 0% | 0% | 93% | 13% | 0% |
| 64 | Bruniaceae | 0% | 0% | 0% | 100% | 0% | 0% | 0% |
| 65 | Burmanniaceae | 51% | 11% | 0% | 13% | 26% | 9% | 0% |
| 66 | Burseraceae | 18% | 7% | 0% | 34% | 26% | 20% | 0% |
| 67 | Butomaceae | 100% | 0% | 50% | 50% | 0% | 0% | 0% |
| 68 | Buxaceae | 37% | 0% | 2% | 19% | 5% | 41% | 0% |
| 69 | Byblidaceae | 0% | 100% | 0% | 0% | 0% | 0% | 0% |
| 70 | Cabombaceae | 14% | 14% | 0% | 14% | 86% | 71% | 0% |
| 71 | Cactaceae | 0% | 0% | 0% | 0% | 53% | 49% | 0% |
| 72 | Calophyllaceae | 44% | 16% | 0% | 8% | 32% | 5% | 0% |
| 73 | Calycanthaceae | 64% | 9% | 0% | 0% | 0% | 27% | 0% |
| 74 | Calyceraceae | 0% | 0% | 0% | 0% | 98% | 0% | 2% |
| 75 | Campanulaceae | 21% | 10% | 11% | 24% | 25% | 13% | 0% |
| 76 | Campynemataceae | 0% | 100% | 0% | 0% | 0% | 0% | 0% |
| 77 | Canellaceae | 0% | 0% | 0% | 30% | 39% | 35% | 0% |
| 78 | Cannabaceae | 46% | 25% | 4% | 16% | 15% | 22% | 0% |
| 79 | Cannaceae | 0% | 0% | 0% | 0% | 92% | 50% | 0% |
| 80 | Capparaceae | 32% | 7% | 0% | 44% | 17% | 12% | 0% |
| 81 | Caprifoliaceae | 50% | 0% | 21% | 11% | 20% | 11% | 0% |
| 82 | Cardiopteridaceae | 39% | 32% | 0% | 15% | 22% | 2% | 0% |
| 83 | Caricaceae | 0% | 0% | 0% | 5% | 81% | 29% | 0% |
| 84 | Carlemanniaceae | 100% | 0% | 0% | 0% | 0% | 0% | 0% |
| 85 | Caryocaraceae | 0% | 0% | 0% | 0% | 96% | 15% | 0% |
| 86 | Caryophyllaceae | 57% | 3% | 28% | 13% | 8% | 9% | 0% |
| 87 | Casuarinaceae | 8% | 94% | 0% | 0% | 0% | 1% | 0% |
| 88 | Celastraceae | 37% | 9% | 0% | 24% | 18% | 17% | 0% |
| 89 | Centroplacaceae | 78% | 11% | 0% | 11% | 0% | 0% | 0% |
| 90 | Cephalotaceae | 0% | 100% | 0% | 0% | 0% | 0% | 0% |
| 91 | Cephalotaxaceae | 100% | 0% | 0% | 0% | 0% | 0% | 0% |
| 92 | Ceratophyllaceae | 67% | 33% | 67% | 50% | 33% | 50% | 0% |
| 93 | Cercidiphyllaceae | 100% | 0% | 0% | 0% | 0% | 0% | 0% |
| 94 | Chloranthaceae | 25% | 18% | 0% | 1% | 48% | 21% | 0% |
| 95 | Chrysobalanaceae | 5% | 4% | 0% | 12% | 74% | 12% | 0% |
| 96 | Circaeasteraceae | 100% | 0% | 0% | 0% | 0% | 0% | 0% |
| 97 | Cistaceae | 23% | 0% | 45% | 53% | 1% | 20% | 0% |
| 98 | Cleomaceae | 24% | 7% | 1% | 32% | 27% | 25% | 0% |
| 99 | Clethraceae | 19% | 4% | 0% | 1% | 23% | 55% | 0% |
| 100 | Clusiaceae | 23% | 12% | 0% | 11% | 45% | 14% | 0% |
| 101 | Colchicaceae | 36% | 14% | 19% | 41% | 0% | 2% | 0% |
| 102 | Columelliaceae | 0% | 0% | 0% | 0% | 100% | 13% | 0% |
| 103 | Combretaceae | 18% | 16% | 0% | 52% | 15% | 6% | 0% |
| 104 | Commelinaceae | 24% | 6% | 0% | 39% | 19% | 22% | 0% |
| 105 | Connaraceae | 25% | 4% | 0% | 24% | 44% | 10% | 0% |
| 106 | Convolvulaceae | 27% | 8% | 3% | 24% | 32% | 23% | 0% |
| 107 | Coriariaceae | 33% | 60% | 7% | 7% | 7% | 7% | 0% |
| 108 | Cornaceae | 69% | 16% | 4% | 4% | 1% | 19% | 0% |
| 109 | Corsiaceae | 0% | 96% | 0% | 0% | 4% | 0% | 4% |
| 110 | Corynocarpaceae | 0% | 100% | 0% | 0% | 0% | 0% | 0% |
| 111 | Costaceae | 18% | 12% | 0% | 22% | 39% | 21% | 0% |
| 112 | Crassulaceae | 29% | 1% | 7% | 35% | 4% | 30% | 0% |
| 113 | Crossosomataceae | 0% | 0% | 0% | 0% | 0% | 100% | 0% |
| 114 | Crypteroniaceae | 100% | 8% | 0% | 0% | 0% | 0% | 0% |
| 115 | Ctenolophonaceae | 50% | 50% | 0% | 50% | 0% | 0% | 0% |
| 116 | Cucurbitaceae | 30% | 9% | 0% | 27% | 25% | 18% | 0% |
| 117 | Cunoniaceae | 6% | 56% | 0% | 10% | 28% | 5% | 0% |
| 118 | Cupressaceae | 40% | 17% | 9% | 11% | 2% | 34% | 0% |
| 119 | Curtisiaceae | 0% | 0% | 0% | 100% | 0% | 0% | 0% |
| 120 | Cycadaceae | 66% | 34% | 0% | 1% | 0% | 0% | 0% |
| 121 | Cyclanthaceae | 0% | 0% | 0% | 0% | 85% | 31% | 0% |
| 122 | Cymodoceaceae | 41% | 59% | 6% | 47% | 18% | 24% | 0% |
| 123 | Cynomoriaceae | 100% | 0% | 100% | 100% | 0% | 0% | 0% |
| 124 | Cyperaceae | 33% | 18% | 5% | 25% | 19% | 22% | 1% |
| 125 | Cyrillaceae | 0% | 0% | 0% | 0% | 9% | 100% | 0% |
| 126 | Cytinaceae | 17% | 0% | 17% | 67% | 8% | 25% | 0% |
| 127 | Daphniphyllaceae | 97% | 7% | 0% | 0% | 0% | 0% | 0% |
| 128 | Dasypogonaceae | 0% | 100% | 0% | 0% | 0% | 0% | 0% |
| 129 | Datiscaceae | 50% | 0% | 50% | 0% | 0% | 50% | 0% |
| 130 | Degeneriaceae | 0% | 100% | 0% | 0% | 0% | 0% | 0% |
| 131 | Diapensiaceae | 74% | 0% | 5% | 0% | 0% | 37% | 0% |
| 132 | Dichapetalaceae | 9% | 3% | 0% | 55% | 25% | 14% | 0% |
| 133 | Didiereaceae | 0% | 0% | 0% | 100% | 0% | 0% | 0% |
| 134 | Dilleniaceae | 13% | 63% | 0% | 3% | 21% | 4% | 0% |
| 135 | Dioncophyllaceae | 0% | 0% | 0% | 100% | 0% | 0% | 0% |
| 136 | Dioscoreaceae | 25% | 3% | 1% | 13% | 45% | 19% | 0% |
| 137 | Dipentodontaceae | 24% | 14% | 0% | 0% | 52% | 24% | 0% |
| 138 | Dipterocarpaceae | 93% | 3% | 0% | 5% | 0% | 0% | 0% |
| 139 | Dirachmaceae | 0% | 0% | 0% | 100% | 0% | 0% | 0% |
| 140 | Doryanthaceae | 0% | 100% | 0% | 0% | 0% | 0% | 0% |
| 141 | Droseraceae | 6% | 66% | 2% | 15% | 17% | 4% | 0% |
| 142 | Drosophyllaceae | 0% | 0% | 100% | 100% | 0% | 0% | 0% |
| 143 | Ebenaceae | 41% | 11% | 0% | 35% | 11% | 5% | 0% |
| 144 | Ecdeiocoleaceae | 0% | 100% | 0% | 0% | 0% | 0% | 0% |
| 145 | Elaeagnaceae | 96% | 1% | 2% | 0% | 0% | 4% | 0% |
| 146 | Elaeocarpaceae | 40% | 42% | 0% | 2% | 14% | 6% | 0% |
| 147 | Elatinaceae | 28% | 21% | 17% | 36% | 17% | 16% | 2% |
| 148 | Emblingiaceae | 0% | 100% | 0% | 0% | 0% | 0% | 0% |
| 149 | Ephedraceae | 57% | 0% | 10% | 13% | 17% | 18% | 0% |
| 150 | Ericaceae | 35% | 24% | 1% | 19% | 15% | 10% | 0% |
| 151 | Eriocaulaceae | 17% | 5% | 0% | 12% | 63% | 6% | 0% |
| 152 | Erythroxylaceae | 6% | 2% | 0% | 21% | 63% | 17% | 0% |
| 153 | Escalloniaceae | 38% | 30% | 0% | 1% | 32% | 1% | 0% |
| 154 | Eucommiaceae | 100% | 0% | 0% | 0% | 0% | 0% | 0% |
| 155 | Euphorbiaceae | 20% | 10% | 2% | 28% | 26% | 20% | 0% |
| 156 | Euphroniaceae | 0% | 0% | 0% | 0% | 100% | 0% | 0% |
| 157 | Eupomatiaceae | 0% | 100% | 0% | 0% | 0% | 0% | 0% |
| 158 | Eupteleaceae | 100% | 0% | 0% | 0% | 0% | 0% | 0% |
| 159 | Fabaceae | 30% | 13% | 5% | 24% | 22% | 15% | 0% |
| 160 | Fagaceae | 72% | 1% | 3% | 1% | 0% | 25% | 0% |
| 161 | Flagellariaceae | 40% | 80% | 0% | 40% | 0% | 0% | 0% |
| 162 | Fouquieriaceae | 0% | 0% | 0% | 0% | 0% | 100% | 0% |
| 163 | Francoaceae | 3% | 0% | 0% | 47% | 53% | 0% | 0% |
| 164 | Frankeniaceae | 7% | 54% | 7% | 22% | 11% | 9% | 0% |
| 165 | Garryaceae | 37% | 0% | 0% | 0% | 0% | 63% | 0% |
| 166 | Geissolomataceae | 0% | 0% | 0% | 100% | 0% | 0% | 0% |
| 167 | Gelsemiaceae | 14% | 0% | 0% | 57% | 14% | 14% | 0% |
| 168 | Gentianaceae | 40% | 5% | 5% | 14% | 29% | 13% | 0% |
| 169 | Geraniaceae | 25% | 5% | 13% | 52% | 11% | 6% | 0% |
| 170 | Gerrardinaceae | 0% | 0% | 0% | 100% | 0% | 0% | 0% |
| 171 | Gesneriaceae | 49% | 10% | 0% | 5% | 28% | 11% | 0% |
| 172 | Ginkgoaceae | 100% | 0% | 0% | 0% | 0% | 0% | 0% |
| 173 | Gisekiaceae | 29% | 0% | 0% | 100% | 0% | 0% | 0% |
| 174 | Gnetaceae | 70% | 16% | 0% | 9% | 16% | 2% | 0% |
| 175 | Gomortegaceae | 0% | 0% | 0% | 0% | 100% | 0% | 0% |
| 176 | Goodeniaceae | 3% | 98% | 0% | 1% | 0% | 1% | 0% |
| 177 | Goupiaceae | 0% | 0% | 0% | 0% | 50% | 100% | 0% |
| 178 | Griseliniaceae | 0% | 29% | 0% | 0% | 71% | 0% | 0% |
| 179 | Grossulariaceae | 39% | 0% | 5% | 2% | 26% | 34% | 0% |
| 180 | Grubbiaceae | 0% | 0% | 0% | 100% | 0% | 0% | 0% |
| 181 | Guamatelaceae | 0% | 0% | 0% | 0% | 0% | 100% | 0% |
| 182 | Gunneraceae | 2% | 24% | 0% | 2% | 68% | 6% | 2% |
| 183 | Gyrostemonaceae | 0% | 100% | 0% | 0% | 0% | 0% | 0% |
| 184 | Haemodoraceae | 0% | 85% | 0% | 8% | 5% | 3% | 0% |
| 185 | Halophytaceae | 0% | 0% | 0% | 0% | 100% | 0% | 0% |
| 186 | Haloragaceae | 14% | 77% | 2% | 5% | 5% | 10% | 1% |
| 187 | Hamamelidaceae | 71% | 4% | 0% | 15% | 1% | 10% | 0% |
| 188 | Hanguanaceae | 100% | 5% | 0% | 0% | 0% | 0% | 0% |
| 189 | Heliconiaceae | 1% | 3% | 0% | 0% | 72% | 39% | 0% |
| 190 | Helwingiaceae | 100% | 0% | 0% | 0% | 0% | 0% | 0% |
| 191 | Hernandiaceae | 38% | 20% | 0% | 16% | 23% | 20% | 0% |
| 192 | Himantandraceae | 0% | 100% | 0% | 0% | 0% | 0% | 0% |
| 193 | Huaceae | 0% | 0% | 0% | 100% | 0% | 0% | 0% |
| 194 | Humiriaceae | 0% | 0% | 0% | 1% | 93% | 12% | 0% |
| 195 | Hydatellaceae | 8% | 92% | 0% | 0% | 0% | 0% | 0% |
| 196 | Hydrangeaceae | 75% | 1% | 0% | 0% | 4% | 23% | 0% |
| 197 | Hydrocharitaceae | 52% | 25% | 7% | 37% | 13% | 14% | 0% |
| 198 | Hydroleaceae | 7% | 7% | 0% | 43% | 14% | 50% | 0% |
| 199 | Hydrostachyaceae | 0% | 0% | 0% | 100% | 0% | 0% | 0% |
| 200 | Hypericaceae | 40% | 1% | 11% | 18% | 23% | 17% | 0% |
| 201 | Hypoxidaceae | 16% | 11% | 0% | 66% | 3% | 10% | 0% |
| 202 | Icacinaceae | 33% | 9% | 0% | 48% | 9% | 3% | 0% |
| 203 | Iridaceae | 18% | 2% | 7% | 55% | 15% | 7% | 0% |
| 204 | Irvingiaceae | 17% | 0% | 0% | 83% | 0% | 0% | 0% |
| 205 | Iteaceae | 79% | 0% | 0% | 4% | 0% | 17% | 0% |
| 206 | Ixioliriaceae | 100% | 0% | 0% | 0% | 0% | 0% | 0% |
| 207 | Ixonanthaceae | 17% | 6% | 0% | 33% | 50% | 0% | 0% |
| 208 | Joinvilleaceae | 33% | 100% | 0% | 0% | 0% | 0% | 0% |
| 209 | Juglandaceae | 45% | 3% | 3% | 1% | 10% | 46% | 0% |
| 210 | Juncaceae | 37% | 18% | 19% | 13% | 15% | 30% | 2% |
| 211 | Juncaginaceae | 9% | 66% | 11% | 29% | 14% | 17% | 3% |
| 212 | Kewaceae | 0% | 0% | 0% | 100% | 0% | 0% | 0% |
| 213 | Kirkiaceae | 0% | 0% | 0% | 100% | 0% | 0% | 0% |
| 214 | Koeberliniaceae | 0% | 0% | 0% | 0% | 50% | 50% | 0% |
| 215 | Krameriaceae | 0% | 0% | 0% | 0% | 47% | 59% | 0% |
| 216 | Lacistemataceae | 0% | 0% | 0% | 0% | 100% | 18% | 0% |
| 217 | Lamiaceae | 43% | 8% | 8% | 21% | 13% | 14% | 0% |
| 218 | Lanariaceae | 0% | 0% | 0% | 100% | 0% | 0% | 0% |
| 219 | Lardizabalaceae | 95% | 0% | 0% | 0% | 5% | 0% | 0% |
| 220 | Lauraceae | 42% | 15% | 0% | 7% | 27% | 11% | 0% |
| 221 | Lecythidaceae | 17% | 8% | 0% | 17% | 57% | 12% | 0% |
| 222 | Lentibulariaceae | 19% | 20% | 8% | 12% | 29% | 30% | 0% |
| 223 | Lepidobotryaceae | 0% | 0% | 0% | 50% | 50% | 50% | 0% |
| 224 | Liliaceae | 70% | 0% | 14% | 4% | 0% | 21% | 0% |
| 225 | Limeaceae | 12% | 0% | 0% | 92% | 0% | 0% | 0% |
| 226 | Limnanthaceae | 0% | 0% | 0% | 0% | 0% | 100% | 0% |
| 227 | Linaceae | 33% | 4% | 22% | 24% | 10% | 21% | 0% |
| 228 | Linderniaceae | 43% | 18% | 0% | 39% | 3% | 7% | 0% |
| 229 | Loasaceae | 0% | 1% | 0% | 1% | 63% | 38% | 0% |
| 230 | Loganiaceae | 16% | 32% | 0% | 16% | 32% | 10% | 0% |
| 231 | Lophiocarpaceae | 14% | 0% | 0% | 100% | 0% | 0% | 0% |
| 232 | Lophopyxidaceae | 100% | 100% | 0% | 0% | 0% | 0% | 0% |
| 233 | Loranthaceae | 27% | 12% | 0% | 24% | 29% | 12% | 0% |
| 234 | Lowiaceae | 100% | 0% | 0% | 0% | 0% | 0% | 0% |
| 235 | Lythraceae | 22% | 5% | 3% | 19% | 43% | 21% | 0% |
| 236 | Macarthuriaceae | 0% | 100% | 0% | 0% | 0% | 0% | 0% |
| 237 | Magnoliaceae | 53% | 1% | 0% | 0% | 22% | 26% | 0% |
| 238 | Malpighiaceae | 6% | 1% | 0% | 9% | 64% | 27% | 0% |
| 239 | Malvaceae | 19% | 14% | 1% | 28% | 30% | 16% | 0% |
| 240 | Marantaceae | 8% | 2% | 0% | 8% | 74% | 20% | 0% |
| 241 | Marcgraviaceae | 0% | 0% | 0% | 0% | 79% | 39% | 0% |
| 242 | Martyniaceae | 0% | 0% | 0% | 0% | 43% | 71% | 0% |
| 243 | Maundiaceae | 0% | 100% | 0% | 0% | 0% | 0% | 0% |
| 244 | Mayacaceae | 0% | 0% | 0% | 20% | 80% | 40% | 0% |
| 245 | Mazaceae | 91% | 11% | 2% | 0% | 0% | 0% | 0% |
| 246 | Melanthiaceae | 42% | 0% | 3% | 0% | 1% | 58% | 0% |
| 247 | Melastomataceae | 19% | 5% | 0% | 12% | 53% | 17% | 0% |
| 248 | Meliaceae | 32% | 20% | 0% | 28% | 21% | 11% | 0% |
| 249 | Menispermaceae | 34% | 11% | 0% | 24% | 29% | 10% | 0% |
| 250 | Menyanthaceae | 24% | 49% | 3% | 24% | 7% | 10% | 0% |
| 251 | Metteniusaceae | 14% | 6% | 0% | 28% | 43% | 20% | 0% |
| 252 | Microteaceae | 0% | 0% | 0% | 0% | 90% | 20% | 0% |
| 253 | Misodendraceae | 0% | 0% | 0% | 0% | 100% | 0% | 0% |
| 254 | Mitrastemonaceae | 50% | 50% | 0% | 0% | 50% | 50% | 0% |
| 255 | Molluginaceae | 11% | 7% | 2% | 76% | 12% | 11% | 0% |
| 256 | Monimiaceae | 7% | 48% | 0% | 23% | 22% | 4% | 0% |
| 257 | Montiaceae | 4% | 27% | 0% | 0% | 37% | 36% | 1% |
| 258 | Montiniaceae | 0% | 0% | 0% | 100% | 0% | 0% | 0% |
| 259 | Moraceae | 40% | 22% | 0% | 16% | 24% | 11% | 0% |
| 260 | Moringaceae | 23% | 0% | 0% | 85% | 0% | 0% | 0% |
| 261 | Muntingiaceae | 0% | 0% | 0% | 0% | 100% | 67% | 0% |
| 262 | Musaceae | 83% | 16% | 0% | 4% | 0% | 0% | 0% |
| 263 | Myodocarpaceae | 7% | 100% | 0% | 0% | 0% | 0% | 0% |
| 264 | Myricaceae | 16% | 4% | 4% | 39% | 16% | 33% | 0% |
| 265 | Myristicaceae | 47% | 28% | 0% | 5% | 20% | 6% | 0% |
| 266 | Myrothamnaceae | 0% | 0% | 0% | 100% | 0% | 0% | 0% |
| 267 | Myrtaceae | 14% | 48% | 0% | 4% | 24% | 12% | 0% |
| 268 | Nartheciaceae | 62% | 0% | 9% | 0% | 6% | 24% | 0% |
| 269 | Nelumbonaceae | 50% | 50% | 50% | 0% | 50% | 50% | 0% |
| 270 | Nepenthaceae | 91% | 9% | 0% | 2% | 0% | 0% | 0% |
| 271 | Neuradaceae | 25% | 0% | 0% | 88% | 0% | 0% | 0% |
| 272 | Nitrariaceae | 88% | 6% | 19% | 19% | 0% | 6% | 0% |
| 273 | Nothofagaceae | 0% | 71% | 0% | 0% | 29% | 0% | 0% |
| 274 | Nyctaginaceae | 7% | 8% | 0% | 11% | 40% | 46% | 0% |
| 275 | Nymphaeaceae | 26% | 22% | 7% | 13% | 29% | 29% | 0% |
| 276 | Nyssaceae | 86% | 3% | 0% | 0% | 0% | 14% | 0% |
| 277 | Ochnaceae | 4% | 1% | 0% | 23% | 67% | 9% | 0% |
| 278 | Olacaceae | 13% | 10% | 0% | 36% | 37% | 10% | 0% |
| 279 | Oleaceae | 52% | 9% | 2% | 24% | 5% | 12% | 0% |
| 280 | Onagraceae | 15% | 9% | 5% | 4% | 29% | 52% | 0% |
| 281 | Oncothecaceae | 0% | 100% | 0% | 0% | 0% | 0% | 0% |
| 282 | Opiliaceae | 32% | 21% | 0% | 32% | 13% | 16% | 0% |
| 283 | Orchidaceae | 26% | 16% | 1% | 9% | 40% | 14% | 0% |
| 284 | Orobanchaceae | 49% | 3% | 14% | 18% | 7% | 20% | 0% |
| 285 | Oxalidaceae | 8% | 3% | 0% | 37% | 45% | 11% | 0% |
| 286 | Paeoniaceae | 69% | 0% | 42% | 6% | 0% | 6% | 0% |
| 287 | Pandaceae | 41% | 6% | 0% | 59% | 0% | 0% | 0% |
| 288 | Pandanaceae | 35% | 47% | 0% | 20% | 0% | 0% | 0% |
| 289 | Papaveraceae | 82% | 0% | 10% | 9% | 1% | 11% | 0% |
| 290 | Paracryphiaceae | 6% | 97% | 0% | 0% | 0% | 0% | 0% |
| 291 | Passifloraceae | 4% | 1% | 0% | 19% | 64% | 20% | 0% |
| 292 | Paulowniaceae | 100% | 0% | 0% | 0% | 0% | 0% | 0% |
| 293 | Pedaliaceae | 6% | 4% | 0% | 92% | 0% | 0% | 0% |
| 294 | Penaeaceae | 0% | 0% | 0% | 100% | 0% | 0% | 0% |
| 295 | Pennantiaceae | 0% | 100% | 0% | 0% | 0% | 0% | 0% |
| 296 | Pentadiplandraceae | 0% | 0% | 0% | 100% | 0% | 0% | 0% |
| 297 | Pentaphragmataceae | 100% | 3% | 0% | 0% | 0% | 0% | 0% |
| 298 | Pentaphylacaceae | 52% | 10% | 0% | 1% | 25% | 13% | 0% |
| 299 | Penthoraceae | 100% | 0% | 0% | 0% | 0% | 50% | 0% |
| 300 | Peraceae | 8% | 0% | 0% | 53% | 24% | 18% | 0% |
| 301 | Peridiscaceae | 0% | 0% | 0% | 82% | 18% | 0% | 0% |
| 302 | Petenaeaceae | 0% | 0% | 0% | 0% | 0% | 100% | 0% |
| 303 | Petermanniaceae | 0% | 100% | 0% | 0% | 0% | 0% | 0% |
| 304 | Petiveriaceae | 0% | 5% | 0% | 0% | 91% | 36% | 0% |
| 305 | Petrosaviaceae | 100% | 0% | 0% | 0% | 0% | 0% | 0% |
| 306 | Phellinaceae | 0% | 100% | 0% | 0% | 0% | 0% | 0% |
| 307 | Philesiaceae | 0% | 0% | 0% | 0% | 100% | 0% | 0% |
| 308 | Philydraceae | 33% | 100% | 0% | 0% | 0% | 0% | 0% |
| 309 | Phrymaceae | 16% | 8% | 0% | 2% | 4% | 74% | 0% |
| 310 | Phyllanthaceae | 41% | 26% | 0% | 21% | 10% | 8% | 0% |
| 311 | Phyllonomaceae | 0% | 0% | 0% | 0% | 40% | 80% | 0% |
| 312 | Physenaceae | 0% | 0% | 0% | 100% | 0% | 0% | 0% |
| 313 | Phytolaccaceae | 24% | 3% | 0% | 12% | 48% | 27% | 0% |
| 314 | Picramniaceae | 0% | 0% | 0% | 0% | 70% | 43% | 0% |
| 315 | Picrodendraceae | 3% | 65% | 0% | 19% | 5% | 7% | 0% |
| 316 | Pinaceae | 46% | 0% | 9% | 3% | 0% | 47% | 0% |
| 317 | Piperaceae | 12% | 6% | 0% | 2% | 55% | 31% | 0% |
| 318 | Pittosporaceae | 26% | 69% | 0% | 7% | 0% | 0% | 0% |
| 319 | Plantaginaceae | 31% | 13% | 17% | 14% | 14% | 28% | 0% |
| 320 | Platanaceae | 20% | 0% | 10% | 0% | 0% | 80% | 0% |
| 321 | Plocospermataceae | 0% | 0% | 0% | 0% | 0% | 100% | 0% |
| 322 | Plumbaginaceae | 43% | 1% | 46% | 16% | 1% | 1% | 0% |
| 323 | Poaceae | 36% | 13% | 8% | 21% | 23% | 17% | 0% |
| 324 | Podocarpaceae | 32% | 48% | 0% | 7% | 15% | 8% | 0% |
| 325 | Podostemaceae | 32% | 1% | 0% | 30% | 36% | 4% | 0% |
| 326 | Polemoniaceae | 4% | 0% | 1% | 0% | 9% | 89% | 0% |
| 327 | Polygalaceae | 18% | 7% | 4% | 27% | 35% | 15% | 0% |
| 328 | Polygonaceae | 39% | 3% | 8% | 9% | 14% | 42% | 0% |
| 329 | Pontederiaceae | 15% | 9% | 2% | 11% | 60% | 49% | 0% |
| 330 | Portulacaceae | 14% | 11% | 7% | 34% | 38% | 20% | 0% |
| 331 | Posidoniaceae | 22% | 89% | 11% | 11% | 0% | 0% | 0% |
| 332 | Potamogetonaceae | 47% | 26% | 30% | 23% | 17% | 33% | 2% |
| 333 | Primulaceae | 52% | 13% | 4% | 8% | 14% | 14% | 0% |
| 334 | Proteaceae | 3% | 70% | 0% | 21% | 5% | 1% | 0% |
| 335 | Putranjivaceae | 48% | 8% | 0% | 38% | 4% | 6% | 0% |
| 336 | Quillajaceae | 0% | 0% | 0% | 0% | 100% | 0% | 0% |
| 337 | Rafflesiaceae | 100% | 0% | 0% | 0% | 0% | 0% | 0% |
| 338 | Ranunculaceae | 51% | 4% | 36% | 4% | 3% | 10% | 0% |
| 339 | Rapateaceae | 0% | 0% | 0% | 1% | 98% | 3% | 0% |
| 340 | Resedaceae | 48% | 0% | 23% | 38% | 0% | 15% | 0% |
| 341 | Restionaceae | 1% | 35% | 0% | 64% | 0% | 0% | 0% |
| 342 | Rhabdodendraceae | 0% | 0% | 0% | 0% | 100% | 0% | 0% |
| 343 | Rhamnaceae | 27% | 25% | 2% | 20% | 10% | 21% | 0% |
| 344 | Rhizophoraceae | 26% | 20% | 0% | 49% | 15% | 7% | 0% |
| 345 | Ripogonaceae | 0% | 100% | 0% | 0% | 0% | 0% | 0% |
| 346 | Roridulaceae | 0% | 0% | 0% | 100% | 0% | 0% | 0% |
| 347 | Rosaceae | 46% | 2% | 36% | 6% | 5% | 13% | 0% |
| 348 | Rousseaceae | 0% | 93% | 0% | 7% | 0% | 0% | 0% |
| 349 | Rubiaceae | 29% | 13% | 2% | 23% | 23% | 14% | 0% |
| 350 | Ruppiaceae | 45% | 36% | 27% | 36% | 27% | 27% | 9% |
| 351 | Rutaceae | 19% | 38% | 1% | 23% | 14% | 9% | 0% |
| 352 | Sabiaceae | 41% | 1% | 0% | 0% | 38% | 25% | 0% |
| 353 | Salicaceae | 41% | 11% | 6% | 18% | 14% | 20% | 0% |
| 354 | Salvadoraceae | 60% | 10% | 0% | 70% | 0% | 0% | 0% |
| 355 | Santalaceae | 14% | 13% | 3% | 32% | 27% | 18% | 0% |
| 356 | Sapindaceae | 20% | 23% | 1% | 18% | 32% | 15% | 0% |
| 357 | Sapotaceae | 25% | 20% | 0% | 24% | 25% | 11% | 0% |
| 358 | Sarcobataceae | 0% | 0% | 0% | 0% | 0% | 100% | 0% |
| 359 | Sarcolaenaceae | 0% | 0% | 0% | 100% | 0% | 0% | 0% |
| 360 | Sarraceniaceae | 0% | 0% | 0% | 0% | 60% | 40% | 0% |
| 361 | Saururaceae | 67% | 0% | 0% | 0% | 0% | 33% | 0% |
| 362 | Saxifragaceae | 66% | 0% | 17% | 3% | 1% | 22% | 0% |
| 363 | Scheuchzeriaceae | 100% | 0% | 100% | 0% | 0% | 100% | 0% |
| 364 | Schisandraceae | 91% | 0% | 0% | 0% | 0% | 9% | 0% |
| 365 | Schlegeliaceae | 0% | 0% | 0% | 0% | 57% | 59% | 0% |
| 366 | Schoepfiaceae | 12% | 0% | 0% | 0% | 41% | 56% | 0% |
| 367 | Sciadopityaceae | 100% | 0% | 0% | 0% | 0% | 0% | 0% |
| 368 | Scrophulariaceae | 31% | 14% | 7% | 45% | 4% | 3% | 0% |
| 369 | Setchellanthaceae | 0% | 0% | 0% | 0% | 0% | 100% | 0% |
| 370 | Simaroubaceae | 18% | 16% | 0% | 15% | 37% | 27% | 0% |
| 371 | Simmondsiaceae | 0% | 0% | 0% | 0% | 0% | 100% | 0% |
| 372 | Siparunaceae | 0% | 0% | 0% | 2% | 96% | 17% | 0% |
| 373 | Sladeniaceae | 67% | 0% | 0% | 33% | 0% | 0% | 0% |
| 374 | Smilacaceae | 56% | 10% | 1% | 2% | 21% | 19% | 0% |
| 375 | Solanaceae | 7% | 12% | 1% | 6% | 59% | 24% | 0% |
| 376 | Sphaerosepalaceae | 0% | 0% | 0% | 100% | 0% | 0% | 0% |
| 377 | Sphenocleaceae | 50% | 50% | 0% | 100% | 0% | 0% | 0% |
| 378 | Stachyuraceae | 100% | 0% | 0% | 0% | 0% | 0% | 0% |
| 379 | Staphyleaceae | 72% | 7% | 2% | 0% | 5% | 21% | 0% |
| 380 | Stegnospermataceae | 0% | 0% | 0% | 0% | 0% | 100% | 0% |
| 381 | Stemonaceae | 87% | 15% | 0% | 0% | 3% | 3% | 0% |
| 382 | Stemonuraceae | 68% | 27% | 0% | 5% | 3% | 1% | 0% |
| 383 | Stilbaceae | 8% | 0% | 0% | 100% | 0% | 0% | 0% |
| 384 | Strasburgeriaceae | 0% | 100% | 0% | 0% | 0% | 0% | 0% |
| 385 | Strelitziaceae | 0% | 0% | 0% | 92% | 8% | 0% | 0% |
| 386 | Stylidiaceae | 2% | 98% | 0% | 0% | 1% | 0% | 0% |
| 387 | Styracaceae | 47% | 1% | 1% | 0% | 37% | 18% | 0% |
| 388 | Surianaceae | 11% | 56% | 0% | 11% | 11% | 56% | 0% |
| 389 | Symplocaceae | 42% | 11% | 0% | 0% | 37% | 13% | 0% |
| 390 | Talinaceae | 4% | 0% | 0% | 61% | 21% | 29% | 0% |
| 391 | Tamaricaceae | 94% | 0% | 19% | 14% | 0% | 0% | 0% |
| 392 | Tapisciaceae | 33% | 0% | 0% | 0% | 50% | 33% | 0% |
| 393 | Taxaceae | 75% | 4% | 4% | 4% | 0% | 21% | 0% |
| 394 | Tecophilaeaceae | 0% | 0% | 0% | 63% | 33% | 4% | 0% |
| 395 | Tetracarpaeaceae | 0% | 100% | 0% | 0% | 0% | 0% | 0% |
| 396 | Tetrachondraceae | 0% | 33% | 0% | 0% | 67% | 33% | 0% |
| 397 | Tetramelaceae | 100% | 100% | 0% | 0% | 0% | 0% | 0% |
| 398 | Tetrameristaceae | 25% | 0% | 0% | 0% | 75% | 50% | 0% |
| 399 | Theaceae | 93% | 1% | 0% | 0% | 2% | 5% | 0% |
| 400 | Thomandersiaceae | 0% | 0% | 0% | 100% | 0% | 0% | 0% |
| 401 | Thurniaceae | 0% | 0% | 0% | 25% | 75% | 0% | 0% |
| 402 | Thymelaeaceae | 28% | 24% | 5% | 35% | 7% | 5% | 0% |
| 403 | Ticodendraceae | 0% | 0% | 0% | 0% | 0% | 100% | 0% |
| 404 | Tiganophytaceae | 0% | 0% | 0% | 100% | 0% | 0% | 0% |
| 405 | Tofieldiaceae | 41% | 0% | 7% | 0% | 34% | 28% | 0% |
| 406 | Torricelliaceae | 27% | 0% | 0% | 73% | 0% | 0% | 0% |
| 407 | Tovariaceae | 0% | 0% | 0% | 0% | 50% | 100% | 0% |
| 408 | Trigoniaceae | 3% | 0% | 0% | 3% | 94% | 6% | 0% |
| 409 | Trimeniaceae | 0% | 100% | 0% | 0% | 0% | 0% | 0% |
| 410 | Triuridaceae | 45% | 20% | 0% | 17% | 27% | 9% | 0% |
| 411 | Trochodendraceae | 100% | 0% | 0% | 0% | 0% | 0% | 0% |
| 412 | Tropaeolaceae | 0% | 0% | 0% | 0% | 100% | 3% | 0% |
| 413 | Typhaceae | 87% | 6% | 37% | 10% | 6% | 19% | 0% |
| 414 | Ulmaceae | 60% | 0% | 10% | 3% | 17% | 26% | 0% |
| 415 | Urticaceae | 47% | 15% | 1% | 8% | 18% | 19% | 0% |
| 416 | Vahliaceae | 60% | 0% | 0% | 100% | 0% | 0% | 0% |
| 417 | Velloziaceae | 1% | 0% | 0% | 18% | 81% | 0% | 0% |
| 418 | Verbenaceae | 2% | 0% | 0% | 10% | 65% | 30% | 0% |
| 419 | Violaceae | 28% | 8% | 15% | 18% | 24% | 18% | 0% |
| 420 | Vitaceae | 44% | 8% | 0% | 40% | 7% | 6% | 0% |
| 421 | Vochysiaceae | 0% | 0% | 0% | 1% | 97% | 5% | 0% |
| 422 | Welwitschiaceae | 0% | 0% | 0% | 100% | 0% | 0% | 0% |
| 423 | Winteraceae | 3% | 89% | 0% | 1% | 7% | 1% | 0% |
| 424 | Xeronemataceae | 0% | 100% | 0% | 0% | 0% | 0% | 0% |
| 425 | Xyridaceae | 5% | 7% | 0% | 17% | 64% | 11% | 0% |
| 426 | Zamiaceae | 0% | 18% | 0% | 25% | 13% | 44% | 0% |
| 427 | Zingiberaceae | 81% | 11% | 0% | 5% | 2% | 1% | 0% |
| 428 | Zosteraceae | 48% | 33% | 14% | 14% | 5% | 19% | 0% |
| 429 | Zygophyllaceae | 34% | 18% | 2% | 40% | 11% | 12% | 0% |

**Table S10. Distribution situation of the 18 distribution types in the north temperate zone, the tropical zone, the south temperate zone and seven continents**

The 0 and 1 represent non-distribution type center and distribution type center in the above regions, respectively.

| **Distribution type** | **North temperate zone** | **Tropical zone** | **South temperate zone** | **Asia** | **Oceania** | **Europe** | **Africa** | **South America** | **North America** | **Antarctica** |
| --- | --- | --- | --- | --- | --- | --- | --- | --- | --- | --- |
| I | 1 | 1 | 1 | 1 | 1 | 1 | 1 | 1 | 1 | 0 |
| II | 0 | 1 | 0 | 1 | 1 | 0 | 1 | 1 | 1 | 0 |
| III | 0 | 1 | 0 | 1 | 0 | 0 | 0 | 1 | 1 | 0 |
| IV | 0 | 1 | 0 | 1 | 1 | 0 | 1 | 0 | 0 | 0 |
| V | 0 | 1 | 0 | 1 | 1 | 0 | 0 | 0 | 0 | 0 |
| VI | 0 | 1 | 0 | 1 | 0 | 0 | 1 | 0 | 0 | 0 |
| VII | 0 | 1 | 0 | 0 | 0 | 0 | 1 | 1 | 0 | 0 |
| VIII | 0 | 1 | 0 | 1 | 0 | 0 | 0 | 0 | 0 | 0 |
| IX | 0 | 1 | 0 | 0 | 0 | 0 | 0 | 1 | 1 | 0 |
| X | 0 | 1 | 0 | 0 | 1 | 0 | 0 | 0 | 0 | 0 |
| XI | 0 | 1 | 0 | 0 | 0 | 0 | 1 | 0 | 0 | 0 |
| XII | 1 | 0 | 0 | 1 | 0 | 1 | 0 | 0 | 1 | 0 |
| XIII | 1 | 0 | 0 | 1 | 0 | 0 | 0 | 0 | 1 | 0 |
| XIV | 1 | 0 | 1 | 1 | 0 | 1 | 1 | 0 | 1 | 0 |
| XV | 1 | 0 | 0 | 1 | 0 | 0 | 0 | 0 | 0 | 0 |
| XVI | 1 | 0 | 0 | 0 | 0 | 0 | 0 | 0 | 1 | 0 |
| XVII | 0 | 0 | 1 | 0 | 0 | 0 | 0 | 0 | 0 | 0 |
| XVIII | 1 | 0 | 1 | 1 | 0 | 1 | 0 | 1 | 1 | 0 |

**REFERENCES**

**Alcantara, S., Ree, R.H., and Mello-Silva, R.** (2018). Accelerated diversification and functional trait evolution in Velloziaceae reveal new insights into the origins of the campos rupestres' exceptional floristic richness. Ann. Bot. **122:** 165–180.

**Appelhans, M.S., Keßler, P.J.A., Smets, E., Razafimandimbison, S.G., and Janssens, S.B.** (2012). Age and historical biogeography of the pantropically distributed Spathelioideae (Rutaceae, Sapindales). J. Biogeogr. **39:** 1235–1250.

**Bansal, M., Morley, R.J., Nagaraju, S.K., Dutta, S., Mishra, A.K., Selveraj, J., Kumar, S., Niyolia, D., Harish, S.M., Abdelrahim, O.B., Hasan, S.E., Ramesh, B.R., Dayanandan, S., Morley, H.P., Ashton, P.S., and Prasad, V.** (2022). Southeast Asian Dipterocarp origin and diversification driven by Africa-India floristic interchange. Science **375:** 455–460.

**Bardon, L., Sothers, C., Prance, G.T., Malé , P.J.G., Xi, Z.X., Davis, C.C., Murienne, J., Garcia-Villacorta, R., Coissac, E., Lavergne, S., and Chave, J.** (2016). Unraveling the biogeographical history of Chrysobalanaceae from plastid genomes. Am. J. Bot. **103:** 1089–1102.

**Berger, B.A., Kriebel, R., Spalink, D., and Sytsma, K.J.** (2016). Divergence times, historical biogeography, and shifts in speciation rates of Myrtales. Mol. Phylogenet. Evol. **95:** 116–136.

**Birch, J.L., and Keeley, S.C.** (2013). Dispersal pathways across the Pacific: the historical biogeography of *Astelia* s.l. (Asteliaceae, Asparagales). J. Biogeogr. **40:** 1914–1927.

**Birch, J.L., and Kocyan, A.** (2021). Biogeography of the monocotyledon astelioid clade (Asparagales): A history of long-distance dispersal and diversification with emerging habitats. Mol. Phylogenet. Evol. **163:** 107203.

**Biswal, D.K., Debnath, M., Konhar, R., Yanthan, S., and Tandon, P.** (2018). Phylogeny and biogeography of carnivorous plant family Nepenthaceae with reference to the Indian pitcher plant *Nepenthes khasiana* reveals an Indian subcontinent origin of *Nepenthes* colonization in South East Asia during the Miocene epoch. Front. Ecol. Evol. **6:** 108.

**Bitencourt, C., Nürk, N.M., Rapini, A., Fishbein, M., Simoes, A.O., Middleton, D.J., Meve, U., Endress, M.E., and Liede-Schumann, S.** (2021). Evolution of dispersal, habit, and pollination in Africa pushed Apocynaceae diversification after the Eocene-Oligocene climate transition. Front. Ecol. Evol. **9:** 719741.

**Bremer, K.R., and Janssen, T.** (2006). Gondwanan origin of major monocot groups inferred from dispersal–vicariance analysis. Aliso **22:** 22–27.

**Brown, M.J.M., Walker, B.E., Black, N., Govaerts, R.H.A., Ondo, I., Turner, R., and Lughadha, E.N.** (2023). RWCVP: a companion R package for the World Checklist of Vascular Plants. New Phytol. **240:** 1355–1365.

**Brown, R.W.** (1962). Paleocene flora of the Rocky Mountains and Great Plains. U.S. Geol. Surv. Prof. Pap. **375:** 1–119.

**Buerki, S., Forest, F., Alvarez, N., Nylander, J.A.A., Arrigo, N., and Sanmartín, I.** (2011). An evaluation of new parsimony-based versus parametric inference methods in biogeography: a case study using the globally distributed plant family Sapindaceae. J. Biogeogr. **38:** 531–550.

**Buerki, S., Manning, J.C., and Forest, F.** (2013). Spatio-temporal history of the disjunct family Tecophilaeaceae: a tale involving the colonization of three Mediterranean-type ecosystems. Ann. Bot. **111:** 361–373.

**Cai, L.M., Xi, Z.X., Peterson, K., Rushworth, C., Beaulieu, J., and Davis, C.C.** (2016). Phylogeny of Elatinaceae and the tropical Gondwanan origin of the Centroplacaceae (Malpighiaceae, Elatinaceae) clade. PLoS One **11:** e0161881.

**Cao, Y.N., Comes, H.P., Sakaguchi, S., Chen, L.Y., and Qiu, Y.X.** (2016). Evolution of East Asia's Arcto-Tertiary relict *Euptelea* (Eupteleaceae) shaped by Late Neogene vicariance and Quaternary climate change. BMC Evol. Biol. **16:** 66.

**Carvalho, F.A., and Renner, S.S.** (2012). A dated phylogeny of the papaya family (Caricaceae) reveals the crop's closest relatives and the family's biogeographic history. Mol. Phylogenet. Evol. **65:** 46–53.

**Chacón, J., de Assis, M.C., Meerow, A.W., and Renner, S.S.** (2012). From East Gondwana to Central America: historical biogeography of the Alstroemeriaceae. J. Biogeogr. **39:** 1806–1818.

**Chacón, J., and Renner, S.S.** (2014). Assessing model sensitivity in ancestral area reconstruction using Lagrange: a case study using the Colchicaceae family. J. Biogeogr. **41:** 1414–1427.

**Chandler, G.T., Plunkett, G.M., Pinney, S.M., Cayzer, L.W., and Gemmill, C.E.C.** (2007). Molecular and morphological agreement in Pittosporaceae: phylogenetic analysis with nuclear ITS and plastidtrnL–trnF sequence data. Aust. Syst. Bot. **20:** 390–401.

**Chen, L.Y., Chen, J.M., Gituru, R.W., Temam, T.D., and Wang, Q.F.** (2012a). Generic phylogeny and historical biogeography of Alismataceae, inferred from multiple DNA sequences. Mol. Phylogenet. Evol. **63:** 407–416.

**Chen, L.Y., Chen, J.M., Gituru, R.W., and Wang, Q.F.** (2012b). Generic phylogeny, historical biogeography and character evolution of the cosmopolitan aquatic plant family Hydrocharitaceae. BMC Evol. Biol. **12:** 30.

**Chen, L.Y., Chen, J.M., Gituru, R.W., and Wang, Q.F.** (2013). Eurasian origin of Alismatidae inferred from statistical dispersal-vicariance analysis. Mol. Phylogenet. Evol. **67:** 38–42.

**Chen, L.Y., Grimm, G.W., Wang, Q.F., and Renner, S.S.** (2015). A phylogeny and biogeographic analysis for the Cape-Pondweed family Aponogetonaceae (Alismatales). Mol. Phylogenet. Evol. **82:** 111–117.

**Chen, L.Y., Zhao, S.Y., Mao, K.S., Les, D.H., Wang, Q.F., and Moody, M.L.** (2014). Historical biogeography of Haloragaceae: An out-of-Australia hypothesis with multiple intercontinental dispersals. Mol. Phylogenet. Evol. **78:** 87–95.

**Chen, Z.D., Manchester, S.R., and Sun, H.Y.** (1999). Phylogeny and evolution of the Betulaceae as inferred from DNA sequences, morphology, and paleobotany. Am. J. Bot. **86:** 1168–1181.

**Christenhusz, M.J.M., Fay, M.F., and Chase, M.W.** (2017). Plants of the world: An illustrated encyclopedia of vascular plant families. Richmond, Royal Botanic Gardens.

**Clayton, J.W., Soltis, P.S., and Soltis, D.E.** (2009). Recent long-distance dispersal overshadows ancient biogeographical patterns in a pantropical angiosperm family (Simaroubaceae, Sapindales). Syst. Biol. **58:** 395–410.

**Coiro, M., Allio, R., Mazet, N., Seyfullah, L.J., and Condamine, F.L.** (2023). Reconciling fossils with phylogenies reveals the origin and macroevolutionary processes explaining the global cycad biodiversity. New Phytol. **240:** 1616–1635.

**Crowl, A.A., Miles, N.W., Visger, C.J., Hansen, K., Ayers, T., Haberle, R., and Cellinese, N.** (2016). A global perspective on Campanulaceae: Biogeographic, genomic, and floral evolution. Am. J. Bot. **103:** 233–245.

**Davis, C.C., Bell, C.D., Mathews, S., and Donoghue, M.J.** (2002). Laurasian migration explains Gondwanan disjunctions: Evidence from Malpighiaceae. Proc. Natl. Acad. Sci. U.S.A. **99:** 6833–6837.

**Deng, J.B., Drew, B.T., Mavrodiev, E.V., Gitzendanner, M.A., Soltis, P.S., and Soltis, D.E.** (2015). Phylogeny, divergence times, and historical biogeography of the angiosperm family Saxifragaceae. Mol. Phylogenet. Evol. **83:** 86–98.

**Deng, T.** (2015). Origin and diversification of important character taxa in the East Asian flora. Kunming: Yunnan University.

**Donoghue, M.J.** (2008). A phylogenetic perspective on the distribution of plant diversity. Proc. Natl. Acad. Sci. U.S.A. **105:** 11549–11555.

**Dupin, J., Matzke, N.J., Särkinen, T., Knapp, S., Olmstead, R.G., Bohs, L., and Smith, S.D.** (2017). Bayesian estimation of the global biogeographical history of the Solanaceae. J. Biogeogr. **44:** 887–899.

**Ebersbach, J., Muellner-Riehl, A.N., Michalak, I., Tkach, N., Hoffmann, M.H., Röser, M., Sun, H., and Favre, A.** (2017). In and out of the Qinghai-Tibet Plateau: divergence time estimation and historical biogeography of the large arctic-alpine genus *Saxifraga* L. J. Biogeogr. **44:** 900–910.

**Ellison, A.M., Butler, E.D., Hicks, E.J., Naczi, R.F., Calie, P.J., Bell, C.D., and Davis, C.C.** (2012). Phylogeny and biogeography of the carnivorous plant family Sarraceniaceae. PLoS One **7:** e39291.

**Farris, D.W., Jaramillo, C., Bayona, G., Restrepo-Moreno, S.A., Montes, C., Cardona, A., Mora, A., Speakman, R.J., Glascock, M.D., and Valencia, V.** (2011). Fracturing of the Panamanian Isthmus during initial collision with South America. Geology **39:** 1007–1010.

**Feng, Y., Comes, H.P., and Qiu, Y.X.** (2020). Phylogenomic insights into the temporal-spatial divergence history, evolution of leaf habit and hybridization in *Stachyurus* (Stachyuraceae). Mol. Phylogenet. Evol. **150:** 106878.

**Fishbein, M., Livshultz, T., Straub, S.C.K., Sitnoes, A.O., Boutte, J., McDonnell, A., and Foote, A.** (2018). Evolution on the backbone: Apocynaceae phylogenomics and new perspectives on growth forms, flowers, and fruits. Am. J. Bot. **105:** 495–513.

**Franzke, A., German, D., Al-Shehbaz, I.A., and Mummenhoff, K.** (2009). *Arabidopsis* family ties: molecular phylogeny and age estimates in Brassicaceae. Taxon **58:** 425–437.

**Fritsch, P.W., Manchester, S.R., Stone, R.D., Cruz, B.C., and Almeda, F.** (2015). Northern Hemisphere origins of the amphi-Pacific tropical plant family Symplocaceae. J. Biogeogr. **42:** 891–901.

**Frost, L., Santamaria-Aguilar, D.A., Singletary, D., and Lagomarsino, L.P.** (2022). Neotropical niche evolution of *Otoba* trees in the context of global biogeography of the nutmeg family. J. Biogeogr. **49:** 156–170.

**Fu, C.N., Mo, Z.Q., Yang, J.B., Ge, X.J., Li, D.Z., Xiang, Q.J., and Gao, L.M.** (2019). Plastid phylogenomics and biogeographic analysis support a trans-Tethyan origin and rapid early radiation of Cornales in the Mid-Cretaceous. Mol. Phylogenet. Evol. **140:** 106601.

**Fuller, D.Q., and Hickey, L.J.** (2005). Systematics and leaf architecture of the Gunneraceae. Bot. Rev. **71:** 295–353.

**Gallaher, T.J., Peterson, P.M., Soreng, R.J., Zuloaga, F.O., Li, D.Z., Clark, L.G., Tyrrell, C.D., Welker, C.A.D., Kellogg, E.A., and Teisher, J.K.** (2022). Grasses through space and time: An overview of the biogeographical and macroevolutionary history of Poaceae. J. Syst. Evol. **60:** 522–569.

**Gaynor, M.L., Fu, C.N., Gao, L.M., Lu, L.M., Soltis, D.E., and Soltis, P.S.** (2020). Biogeography and ecological niche evolution in Diapensiaceae inferred from phylogenetic analysis. J. Syst. Evol. **58:** 646–662.

**Givnish, T.J., Barfuss, M.H.J., Van Ee, B., Riina, R., Schulte, K., Horres, R., Gonsiska, P.A., Jabaily, R.S., Crayn, D.M., Smith, J.A.C., Winter, K., Brown, G.K., Evans, T.M., Holst, B.K., Luther, H., Till, W., Zizka, G., Berry, P.E., and Sytsma, K.J.** (2011). Phylogeny, adaptive radiation, and historical biogeography in Bromeliaceae: insights from an eight-locus plastid phylogeny. Am. J. Bot. **98:** 872–895.

**Givnish, T.J., Spalink, D., Ames, M., Lyon, S.P., Hunter, S.J., Zuluaga, A., Doucette, A., Caro, G.G., McDaniel, J., Clements, M.A., Arroyo, M.T.K., Endara, L., Kriebel, R., Williams, N.H., and Cameron, K.M.** (2016a). Orchid historical biogeography, diversification, Antarctica and the paradox of orchid dispersal. J. Biogeogr. **43:** 1905–1916.

**Givnish, T.J., Zuluaga, A., Marques, I., Lam, V.K.Y., Gomez, M.S., Iles, W.J.D., Ames, M., Spalink, D., Moeller, J.R., Briggs, B.G., Lyon, S.P., Stevenson, D.W., Zomlefer, W., and Graham, S.W.** (2016b). Phylogenomics and historical biogeography of the monocot order Liliales: out of Australia and through Antarctica. Cladistics **32:** 581–605.

**Goncalves, D.J.P., Shimizu, G.H., Ortiz, E.M., Jansen, R.K., and Simpson, B.B.** (2020). Historical biogeography of Vochysiaceae reveals an unexpected perspective of plant evolution in the Neotropics. Am. J. Bot. **107:** 1004–1020.

**Grímsson, F., Grimm, G.W., and Zetter, R.** (2017). Tiny pollen grains: First evidence of Saururaceae from the Late Cretaceous of western North America. PeerJ **5:** e3434.

**Grimsson, F., Zetter, R., Halbritter, H., and Grimm, G.W.** (2014). *Aponogeton* pollen from the Cretaceous and Paleogene of North America and West Greenland: Implications for the origin and palaeobiogeography of the genus. Rev. Palaeobot. Palynol. **200:** 161–187.

**Guo, S.X.** (2000). Evolution, palaeobiogeography and palaeoecology of Eucommiaceae. Paleobotanist. **49:** 65–83.

**Herbert, J.** (2004). Systematics and biogeography of Myricaceae. Scotland: University of St. Andrews.

**Herman, A.B.** (2013). Albian-Paleocene Flora of the North Pacific: Systematic Composition, Palaeofloristics and Phytostratigraphy. Stratigr. Geol. Correl. **21:** 689–747.

**Hernández-Hernández, T., Colorado, W.B., and Sosa, V.** (2013). Molecular evidence for the origin and evolutionary history of the rare American desert monotypic family Setchellanthaceae. Org. Divers. Evol. **13:** 485–496.

**Herrera, F., Manchester, S.R., Vélez-Juarbe, J., and Jaramillo, C.** (2014). Phytogeographic History of the Humiriaceae (Part 2). Int. J. Plant Sci. **175:** 828–840.

**Hickey, L.J., and Peterson, R.K.** (1978). Zingiberopsis, a fossil genus of the ginger family from Late Cretaceous to early Eocene sediments of Western Interior North America. Can. J. Bot. **56:** 1136–1152.

**Huang, X.H., Deng, T., Moore, M.J., Wang, H.C., Li, Z.M., Lin, N., Yusupov, Z., Tojibaev, K.S., Wang, Y.H., and Sun, H.** (2019). Tropical Asian Origin, boreotropical migration and long-distance dispersal in Nettles (Urticeae, Urticaceae). Mol. Phylogenet. Evol. **137:** 190–199.

**Ickert-Bond, S.M., Rydin, C., and Renner, S.S.** (2009). A fossil-calibrated relaxed clock for *Ephedra* indicates an Oligocene age for the divergence of Asian and New World clades and Miocene dispersal into South America. J. Syst. Evol. **47:** 444–456.

**Iles, W.J., Lee, C., Sokoloff, D.D., Remizowa, M.V., Yadav, S.R., Barrett, M.D., Barrett, R.L., Macfarlane, T.D., Rudall, P.J., and Graham, S.W.** (2014). Reconstructing the age and historical biogeography of the ancient flowering-plant family Hydatellaceae (Nymphaeales). BMC Evol. Biol. **14:** 102.

**Janssen, T., and Bremer, K.** (2004). The age of major monocot groups inferred from 800+ rbcl sequences. Bot. J. Linn. Soc. **146:** 385–398.

**Janssens, S.B., Knox, E.B., Huysmans, S., Smets, E.F., and Merckx, V.S.** (2009). Rapid radiation of *Impatiens* (Balsaminaceae) during Pliocene and Pleistocene: result of a global climate change. Mol. Phylogenet. Evol. **52:** 806–824.

**Janssens, S.B., Vandelook, F., De Langhe, E., Verstraete, B., Smets, E., Vandenhouwe, I., and Swennen, R.** (2016). Evolutionary dynamics and biogeography of Musaceae reveal a correlation between the diversification of the banana family and the geological and climatic history of Southeast Asia. New Phytol. **210:** 1453–1465.

**Jin, J.J., Yang, M.Q., Fritsch, P.W., van Velzen, R., Li, D.Z., and Yi, T.S.** (2020). Born migrators: Historical biogeography of the cosmopolitan family Cannabaceae. J. Syst. Evol. **58:** 461–473.

**Klaus, K.V., and Matzke, N.J.** (2020). Statistical comparison of trait-dependent biogeographical models indicates that Podocarpaceae dispersal is influenced by both seed cone traits and geographical distance. Syst. Biol. **69:** 61–75.

**Kress, W.J., and Specht, C.D.** (2006). The evolutionary and biogeographic origin and diversification of the tropical monocot order Zingiberales. Aliso **22:** 621–632.

**Lai, Y.J., Gandolfo, M.A., Crepet, W.L., and Nixon, K.C.** (2021). *Paleoaltingia* gen. nov., a new genus of Altingiaceae from the Late Cretaceous of New Jersey. Am. J. Bot. **108:** 461–471.

**Li, Y., Smith, T., Svetlana, P., Yang, J., Jin, J.H., and Li, C.S.** (2014). Paleobiogeography of the lotus plant (Nelumbonaceae: Nelumbo) and its bearing on the paleoclimatic changes. Palaeogeogr. Palaeoclimatol. Palaeoecol. **399:** 284–293.

**Li, Z.Z., Lehtonen, S., Martins, K., Wang, Q.F., and Chen, J.M.** (2022). Complete genus-level plastid phylogenomics of Alismataceae with revisited historical biogeography. Mol. Phylogenet. Evol. **166:** 107334.

**Liu, B., Le, C.T., Barrett, R.L., Nickrent, D.L., Chen, Z.D., Lu, L.M., and Vidal-Russell, R.** (2018). Historical biogeography of Loranthaceae (Santalales): Diversification agrees with emergence of tropical forests and radiation of songbirds. Mol. Phylogenet. Evol. **124:** 199–212.

**Liu, J., Lindstrom, A.J., Gong, Y.Q., Dong, S.S., Liu, Y.S., Zhang, S.Z., and Gong, X.** (2024). Eco-evolutionary evidence for the global diversity pattern of *Cycas* (Cycadaceae). J. Integr. Plant Biol. **66:** 1170–1191.

**Liu, J., Lindstrom, A.J., Marler, T.E., and Gong, X.** (2022). Not that young: combining plastid phylogenomic, plate tectonic and fossil evidence indicates a Palaeogene diversification of Cycadaceae. Ann. Bot. **129:** 217–230.

**Liu, J., Lindstrom, A.J., Nagalingum, N.S., Wiens, J.J., and Gong, X.** (2021). Testing the causes of richness patterns in the paleotropics: time and diversification in cycads (Cycadaceae). Ecography **44:** 1606–1618.

**Luebert, F., Couvreur, T.L.P., Gottschling, M., Hilger, H.H., Miller, J.S., and Weigend, M.** (2017). Historical biogeography of Boraginales: West Gondwanan vicariance followed by long-distance dispersal? J. Biogeogr. **44:** 158–169.

**Macqueen, J.** (1967). Some methods for classification and analysis of multivariate observations. In *Proceedings of the fifth* *Berkeley symposium on mathematical statistics and probability, vol 1: Statistics*. Cam L.M.L., Neyman J., eds, (Berkeley: University of California Press), pp. 281–297.

**Mao, K.S., Milne, R.I., Zhang, L.B., Peng, Y.L., Liu, J.Q., Thomas, P., Mill, R.R., and Renner, S.S.** (2012). Distribution of living Cupressaceae reflects the breakup of Pangea. Proc. Natl. Acad. Sci. U.S.A. **109:** 7793–7798.

**Martin-Rodriguez, I., Vargas, P., Ojeda, F., and Fernandez-Mazuecos, M.** (2020). An enigmatic carnivorous plant: ancient divergence of Drosophyllaceae but recent differentiation of *Drosophyllum lusitanicum* across the Strait of Gibraltar. Syst. Biodivers. **18:** 525–537.

**Marx, H.E., O'Leary, N., Yuan, Y.W., Lu-Irving, P., Tank, D.C., Mulgura, M.E., and Olmstead, R.G.** (2010). A molecular phylogeny and classification of Verbenaceae. Am. J. Bot. **97:** 1647–1663.

**Maurin, K.J.L., and Smissen, R.D.** (2022). A dated phylogeny of Argophyllaceae (Asterales) is consistent with spread by long-distance dispersal. N. Z. J. Botan. **60:** 27–44.

**Maurin, O., Anest, A., Forest, F., Turner, I., Barrett, R.L., Cowan, R.C., Wang, L.J., Tomlinson, K.W., and Charles-Dominique, T.** (2023). Drift in the tropics: Phylogenetics and biogeographical patterns in Combretaceae. Global Ecol. Biogeogr. **32:** 1790–1802.

**Mello-Silva, R., Santos, D.Y., Salatino, M.L., Motta, L.B., Cattai, M.B., Sasaki, D., Lovo, J., Pita, P.B., Rocini, C., Rodrigues, C.D., Zarrei, M., and Chase, M.W.** (2011). Five vicariant genera from Gondwana: the Velloziaceae as shown by molecules and morphology. Ann. Bot. **108:** 87–102.

**Menegoz, K., Villarroel, A.E., and Lavandero, N.** (2024). Phylogeny of Berberidopsidales based on nuclear and chloroplast loci, with the description of a new species of *Berberidopsis* endemic to Central Chile. Taxon **73:** 800–817.

**Mennes, C.B., Lam, V.K.Y., Rudall, P.J., Lyon, S.P., Graham, S.W., Smets, E.F., Merckx, V.S.F.T., and Ebach, M.** (2015). Ancient Gondwana break-up explains the distribution of the mycoheterotrophic family Corsiaceae (Liliales). J. Biogeogr. **42:** 1123–1136.

**Merckx, V., Chatrou, L.W., Lemaire, B., Sainge, M.N., Huysmans, S., and Smets, E.F.** (2008). Diversification of myco-heterotrophic angiosperms: Evidence from Burmanniaceae. BMC Evol. Biol. **8:** 178.

**Montes, C., Cardona, A., Jaramillo, C., Pardo, A., Silva, J.C., Valencia, V., Ayala, C., Perez-Angel, L.C., Rodriguez-Parra, L.A., Ramirez, V., and Niño, H.** (2015). Middle Miocene closure of the Central American Seaway. Science **348:** 226–229.

**Muellner, A.N., Vassiliades, D.D., and Renner, S.S.** (2007). Placing Biebersteiniaceae, a herbaceous clade of Sapindales, in a temporal and geographic context. Plant Syst. Evol. **266:** 233–252.

**Müller, S., Salomo, K., Salazar, J., Naumann, J., Jaramillo, M.A., Neinhuis, C., Feild, T.S., and Wanke, S.** (2015). Intercontinental long-distance dispersal of Canellaceae from the New to the Old World revealed by a nuclear single copy gene and chloroplast loci. Mol. Phylogenet. Evol. **84:** 205–219.

**Nauheimer, L., Metzler, D., and Renner, S.S.** (2012). Global history of the ancient monocot family Araceae inferred with models accounting for past continental positions and previous ranges based on fossils. New Phytol. **195:** 938–950.

**Nie, Z.L., Wen, J., Azuma, H., Qiu, Y.L., Sun, H., Meng, Y., Sun, W.B., and Zimmer, E.A.** (2008). Phylogenetic and biogeographic complexity of Magnoliaceae in the Northern Hemisphere inferred from three nuclear data sets. Mol. Phylogenet. Evol. **48:** 1027–1040.

**Ocampo, G., and Columbus, J.T.** (2010). Molecular phylogenetics of suborder Cactineae (Caryophyllales), including insights into photosynthetic diversification and historical biogeography. Am. J. Bot. **97:** 1827–1847.

**Olmstead, R.G.** (2013). Phylogeny and biogeography in Solanaceae, Verbenaceae and Bignoniaceae: a comparison of continental and intercontinental diversification patterns. Bot. J. Linn. Soc. **171:** 80–102.

**Olmstead, R.G., Zjhra, M.L., Lohmann, L.G., Grose, S.O., and Eckert, A.J.** (2009). A molecular phylogeny and classification of Bignoniaceae. Am. J. Bot. **96:** 1731–1743.

**Onstein, R.E., Kissling, W.D., Chatrou, L.W., Couvreur, T.L.P., Morlon, H., and Sauquet, H.** (2019). Which frugivory‐related traits facilitated historical long‐distance dispersal in the custard apple family (Annonaceae)? J. Biogeogr. **46:** 1874–1888.

**Peng, H.W., Xiang, K.L., Erst, A.S., Lian, L., Ortiz, R.D.C., Jabbour, F., Chen, Z.D., and Wang, W.** (2023). A complete genus-level phylogeny reveals the Cretaceous biogeographic diversification of the poppy family. Mol. Phylogenet. Evol. **181:** 107712.

**Pillon, Y., Hopkins, H.C.F., Maurin, O., Epitawalage, N., Bradford, J., Rogers, Z.S., Baker, W.J., and Forest, F.** (2021). Phylogenomics and biogeography of Cunoniaceae (Oxalidales) with complete generic sampling and taxonomic realignments. Am. J. Bot. **108:** 1181–1200.

**Prince, L.M., and Kress, W.J.** (2006). Phylogeny and biogeography of the prayer plant family: Getting to the root problem in Marantaceae. Aliso **22:** 645–659.

**Qi, X.S., Chen, C., Comes, H.P., Sakaguchi, S., Liu, Y.H., Tanaka, N., Sakio, H., and Qiu, Y.X.** (2012). Molecular data and ecological niche modelling reveal a highly dynamic evolutionary history of the East Asian Tertiary relict *Cercidiphyllum* (Cercidiphyllaceae). New Phytol. **196:** 617–630.

**Qi, Z.C., Li, P., Wu, J.J., Gamisch, A., Yang, T., Zhao, Y.P., Xu, W.Q., Chen, S.C., Cameron, K.M., Qiu, Y.X., and Fu, C.X.** (2023). Climatic niche evolution in Smilacaceae (Liliales) drives patterns of species diversification and richness between the Old and New World. J. Syst. Evol. **61:** 733–747.

**Renner, S.S., Barreda, V.D., Telleria, M.C., Palazzesi, L., and Schuster, T.M.** (2020). Early evolution of Coriariaceae (Cucurbitales) in light of a new early Campanian (ca. 82 Mya) pollen record from Antarctica. Taxon **69:** 87–99.

**Renner, S.S., Clausing, G., and Meyer, K.** (2001). Historical biogeography of Melastomataceae: the roles of Tertiary migration and long-distance dispersal. Am. J. Bot. **88:** 1290–1300.

**Renner, S.S., Strijk, J.S., Strasberg, D., and Thébaud, C.** (2010). Biogeography of the Monimiaceae (Laurales): A role for East Gondwana and long-distance dispersal, but not West Gondwana. J. Biogeogr. **37:** 1227–1238.

**Roalson, E.H., and Roberts, W.R.** (2016). Distinct processes drive diversification in different clades of Gesneriaceae. Syst. Biol. **65:** 662–684.

**Rose, J.P., Xiang, C.L., Sytsma, K.J., and Drew, B.T.** (2022). A timeframe for mint evolution: towards a better understanding of trait evolution and historical biogeography in Lamiaceae. Bot. J. Linn. Soc. **200:** 15–38.

**Rutschmann, F., Eriksson, T., Schonenberger, J., and Conti, E.** (2004). Did Crypteroniaceae really disperse out of india? molecular dating evidence from rbcl, ndhf, and rpl16 intron sequences. Int. J. Plant Sci. **165:** S69–S83.

**Sanmartin, I., and Ronquist, F.** (2004). Southern Hemisphere biogeography inferred by event-based models: Plant versus animal patterns. Syst. Biol. **53:** 216–243.

**Särkinen, T., Bohs, L., Olmstead, R.G., and Knapp, S.** (2013). A phylogenetic framework for evolutionary study of the nightshades (Solanaceae): a dated 1000-tip tree. BMC Evol. Biol. **13:** 214.

**Schaefer, H., Heibl, C., and Renner, S.S.** (2009). Gourds afloat: a dated phylogeny reveals an Asian origin of the gourd family (Cucurbitaceae) and numerous oversea dispersal events. Proceedings of the Royal Society B: Biological Sciences **276:** 843–851.

**Schneider, J.V., Jungcurt, T., Cardoso, D., Amorim, A.M., Paule, J., and Zizka, G.** (2022). Predominantly eastward long-distance dispersal in pantropical Ochnaceae inferred from ancestral range estimation and phylogenomics. Front. Ecol. Evol. **10:** 813336.

**Schuster, T.M., Setaro, S.D., and Kron, K.A.** (2013). Age estimates for the buckwheat family Polygonaceae based on sequence data calibrated by fossils and with a focus on the amphi-Pacific Muehlenbeckia. PLoS One **8:** e61261.

**Scotese, C.R.** (2001). Atlas of Earth history, volume 1, paleogeography. Arlington, Paleomap project.

**Shi, G.L., Zhou, Z.Y., and Xie, Z.M.** (2010). A new *Cephalotaxus* and associated epiphyllous fungi from the Oligocene of Guangxi, South China. Rev. Palaeobot. Palynol. **161:** 179–195.

**Smith, A.G., Smith, D.G., and Funnell, B.M.** (2004). Atlas of Cenozoic and Mesozoic Coastlines. Cambridge, Cambridge University Press.

**Smith, S.A., and Brown, J.W.** (2018). Constructing a broadly inclusive seed plant phylogeny. Am. J. Bot. **105:** 302–314.

**Snead, R.G.** (1969). Microfloral diagnosis of the Creaceous-Tertiary boundary, Central Alberta. Res. Coun. Alberta Bull. **25:** 1–148.

**Spalink, D., Drew, B.T., Pace, M.C., Zaborsky, J.G., Starr, J.R., Cameron, K.M., Givnish, T.J., and Sytsma, K.J.** (2016). Biogeography of the cosmopolitan sedges (Cyperaceae) and the area-richness correlation in plants. J. Biogeogr. **43:** 1893–1904.

**Specht, C.D.** (2006). Gondwanan vicariance or dispersal in the tropics? the biogeographic history of the tropical monocot family Costaceae (Zingiberales). Aliso **22:** 631–642.

**Sun, Y.X., Moore, M.J., Meng, A.P., Soltis, P.S., Soltis, D.E., Li, J.Q., and Wang, H.C.** (2013). Complete plastid genome sequencing of Trochodendraceae reveals a significant expansion of the inverted repeat and suggests a Paleogene divergence between the two extant species. PLoS One **8:** e60429.

**Thomas, D.C., Chatrou, L.W., Stull, G.W., Johnson, D.M., Harris, D.J., Thongpairoj, U.S., and Saunders, R.M.K.** (2015). The historical origins of palaeotropical intercontinental disjunctions in the pantropical flowering plant family Annonaceae. Perspect. Plant Ecol. Evol. Syst. **17:** 1–16.

**Thorne, R.F.** (1972). Major disjunctions in the geographic ranges of seed plants. Q. Rev. Biol. **47:** 365–411.

**Thornhill, A.H., Ho, S.Y.W., Külheim, C., and Crisp, M.D.** (2015). Interpreting the modern distribution of Myrtaceae using a dated molecular phylogeny. Mol. Phylogenet. Evol. **93:** 29–43.

**Tiffney, B.H., and Manchester, S.R.** (2001). The use of geological and paleontological evidence in evaluating plant phylogeographic hypotheses in the Northern Hemisphere tertiary. Int. J. Plant Sci. **162:** S3–S17.

**Trovó, M., Andrade, M.J.G.D., Sano, P.T., Ribeiro, P.L., and Berg, C.V.D.** (2013). Molecular phylogenetics and biogeography of Neotropical Paepalanthoideae with emphasis on Brazilian *Paepalanthus* (Eriocaulaceae). Bot. J. Linn. Soc. **171:** 225–243.

**Villaverde, T., Larridon, I., Shah, T.R., Fowler, R.M., Chau, J.H., Olmstead, R.G., and Sanmartín, I.** (2023). Phylogenomics sheds new light on the drivers behind a long-lasting systematic riddle: the figwort family Scrophulariaceae. New Phytol. **240:** 1601–1615.

**Vinnersten, A., and Bremer, K.** (2001). Age and biogeography of major clades in Liliales. Am. J. Bot. **88:** 1695–1703.

**Wagstaff, S.J., and Dawson, M.I.** (2000). Classification, origin, and patterns of diversification of *Corynocarpus* (Corynocarpaceae) inferred from dna sequences. Syst. Bot. **25:** 134–149.

**Wang, Q., Manchester, S.R., Gregor, H.J., Shen, S., and Li, Z.Y.** (2013). Fruits of *Koelreuteria* (Sapindaceae) from the Cenozoic throughout the northern hemisphere: Their ecological, evolutionary, and biogeographic implications. Am. J. Bot. **100:** 422–449.

**Wang, W., Ortiz, R.D.C., Jacques, F.M., Xiang, X.G., Li, H.L., Lin, L., Li, R.Q., Liu, Y., Soltis, P.S., Soltis, D.E., and Chen, Z.D.** (2012). Menispermaceae and the diversification of tropical rainforests near the Cretaceous-Paleogene boundary. New Phytol. **195:** 470–478.

**Wang, W., Xiang, X.G., Xiang, K.L., Ortiz, R.D., Jabbour, F., and Chen, Z.D.** (2020). A dated phylogeny of Lardizabalaceae reveals an unusual long-distance dispersal across the Pacific Ocean and the rapid rise of East Asian subtropical evergreen broadleaved forests in the late Miocene. Cladistics **36:** 447–457.

**Wolfe, A.D., Randle, C.P., Liang, L., and Steiner, K.E.** (2005). Phylogeny and biogeography of Orobanchaceae. Folia Geobot. **40:** 115–134.

**Worth, J.R.P., Sakaguchi, S., Tanaka, N., Yamasaki, M., and Isagi, Y.** (2013). Northern richness and southern poverty: contrasting genetic footprints of glacial refugia in the relictual tree *Sciadopitys verticillata* (Coniferales: Sciadopityaceae). Biol. J. Linn. Soc. **108:** 263–277.

**Wu, Z.Y.** (2003). Revision of the areal-types of the world families of seed plants. Acta Bot. Yunnan. **25:** 535–538.

**Wu, Z.Y., Liu, J., Provan, J., Wang, H., Chen, C.J., Cadotte, M.W., Luo, Y.H., Amorim, B.S., Li, D.Z., and Milne, R.I.** (2018). Testing Darwin's transoceanic dispersal hypothesis for the inland nettle family (Urticaceae). Ecol. Lett. **21:** 1515–1529.

**Wu, Z.Y., Lu, A.M., Tang, Y.C., Chen, Z.D., and Li, D.Z.** (2003a). The families and genera of angiosperms in China: A comprehensive analysis. Beijing, Science Press.

**Wu, Z.Y., Zhou, Z.K., Li, D.Z., Peng, H., and Sun, H.** (2003b). The areal-types of the world families of seed plants. Acta Bot. Yunnan. **25:** 245–257.

**Wu, Z.Y., Zhou, Z.K., Sun, H., Li, D.Z., and Peng, H.** (2006). The areal-types of seed plants and their origin and differentiation. Kunming, Yunnan Science & Technology Press.

**Xue, J.H., Dong, W.P., Cheng, T., and Zhou, S.L.** (2012). Nelumbonaceae: Systematic position and species diversification revealed by the complete chloroplast genome. J. Syst. Evol. **50:** 477–487.

**Yan, Y.J., Davis, C.C., Dimitrov, D., Wang, Z.H., Rahbek, C., and Borregaard, M.K.** (2021). Phytogeographic history of the tea family inferred through high-resolution phylogeny and fossils. Syst. Biol. **70:** 1256–1271.

**Yang, T., Lu, L.M., Wang, W., Li, J.H., Manchester, S.R., Wen, J., and Chen, Z.D.** (2018). Boreotropical range expansion and long-distance dispersal explain two amphi-Pacific tropical disjunctions in Sabiaceae. Mol. Phylogenet. Evol. **124:** 181–191.

**Yu, X.Q., Gao, L.M., Soltis, D.E., Soltis, P.S., Yang, J.B., Fang, L., Yang, S.X., and Li, D.Z.** (2017). Insights into the historical assembly of East Asian subtropical evergreen broadleaved forests revealed by the temporal history of the tea family. New Phytol. **215:** 1235–1248.

**Yuan, C.H., and Yang, H.T.** (2019). Research on K-Value Selection Method of K-Means Clustering Algorithm. J **2:** 226–235.

**Zhang, L.B., Simmons, M.P., and Renner, S.S.** (2007). A phylogeny of Anisophylleaceae based on six nuclear and plastid loci: Ancient disjunctions and recent dispersal between South America, Africa, and Asia. Mol. Phylogenet. Evol. **44:** 1057–1067.

**Zhang, Q.Y., Deng, M., Bouchenak-Khelladi, Y., Zhou, Z.K., Hu, G.W., and Xing, Y.W.** (2022a). The diversification of the northern temperate woody flora - A case study of the Elm family (Ulmaceae) based on phylogenomic and paleobotanical evidence. J. Syst. Evol. **60:** 728–746.

**Zhang, Q.Y., Ree, R.H., Salamin, N., Xing, Y.W., and Silvestro, D.** (2022b). Fossil-informed models reveal a boreotropical origin and divergent evolutionary trajectories in the walnut family (Juglandaceae). Syst. Biol. **71:** 242–258.

**Zhang, X., Sun, Y.X., Landis, J.B., Zhang, J.W., Yang, L.S., Lin, N., Zhang, H.J., Guo, R., Li, L.J., Zhang, Y.H., Deng, T., Sun, H., and Wang, H.C.** (2020). Genomic insights into adaptation to heterogeneous environments for the ancient relictual *Circaeaster agrestis* (Circaeasteraceae, Ranunculales). New Phytol. **228:** 285–301.

**Zhang, Z.S., Ramstein, G., Schuster, M., Li, C., Contoux, C., and Yan, Q.** (2014). Aridification of the Sahara desert caused by Tethys Sea shrinkage during the Late Miocene. Nature **513:** 401–404.

**Zhao, Y.P., Fan, G.Y., Yin, P.P., Sun, S., Li, N., Hong, X.N., Hu, G., Zhang, H., Zhang, F.M., Han, J.D., Hao, Y.J., Xu, Q.W., Yang, X.W., Xia, W.J., Chen, W.B., Lin, H.Y., Zhang, R., Chen, J., Zheng, X.M., Lee, S.M.Y., Lee, J., Uehara, K., Wang, J.A., Yang, H.M., Fu, C.X., Liu, X., Xu, X., and Ge, S.** (2019). Resequencing 545 ginkgo genomes across the world reveals the evolutionary history of the living fossil. Nat. Commun. **10:** 4201.

**Zhou, S.L., Xu, C., Liu, J., Yu, Y., Wu, P., Cheng, T., and Hong, D.Y.** (2021). Out of the Pan-Himalaya: Evolutionary history of the Paeoniaceae revealed by phylogenomics. J. Syst. Evol. **59:** 1170–1182.

**Zhou, Z.K., and Momohara, A.** (2005). Fossil history of some endemic seed plants of East Asia and its phytogeographical significance. Acta Bot. Yunnan. **27:** 449–470.

**Zhu, Y.P., Wen, J., Zhang, Z.Y., and Chen, Z.D.** (2006). Evolutionary relationships and diversification of Stachyuraceae based on sequences of four chloroplast markers and the nuclear ribosomal its region. Taxon **55:** 931–940.
